# Tetramine Aspect Ratio and Flexibility Determine Framework Symmetry for Zn_8_L_6_ Self‐Assembled Structures

**DOI:** 10.1002/anie.202217987

**Published:** 2023-02-01

**Authors:** Jack A. Davies, Andrew Tarzia, Tanya K. Ronson, Florian Auras, Kim E. Jelfs, Jonathan R. Nitschke

**Affiliations:** ^1^ Yusuf Hamied Department of Chemistry University of Cambridge Lensfield Road Cambridge CB2 1EW UK; ^2^ Department of Chemistry Molecular Sciences Research Hub Imperial College London White City Campus Wood Lane London W12 0BZ UK; ^3^ Department of Synthetic Materials and Functional Devices Max-Planck Institute of Microstructure Physics Weinberg 2 06120 Halle Germany

**Keywords:** Metal-Organic Cages, Self-Assembly, Stereochemistry, Supramolecular Chemistry

## Abstract

We derive design principles for the assembly of rectangular tetramines into Zn_8_L_6_ pseudo‐cubic coordination cages. Because of the rectangular, as opposed to square, geometry of the ligand panels, and the possibility of either Δ or Λ handedness of each metal center at the eight corners of the pseudo‐cube, many different cage diastereomers are possible. Each of the six tetra‐aniline subcomponents investigated in this work assembled with zinc(II) and 2‐formylpyridine in acetonitrile into a single Zn_8_L_6_ pseudo‐cube diastereomer, however. Each product corresponded to one of four diastereomeric configurations, with *T*, *T*_h_, *S*_6_ or *D*_3_ symmetry. The preferred diastereomer for a given tetra‐aniline subcomponent was shown to be dependent on its aspect ratio and conformational flexibility. Analysis of computationally modeled individual faces or whole pseudo‐cubes provided insight as to why the observed diastereomers were favored.

## Graphical abstract

Geometric design rules for the assembly of metal‐organic capsules were derived. The preferred diastereomeric configurations of Zn_8_L_6_ pseudo‐cubes, with *T*, *T*_h_, *S*_6_ and *D*_3_ symmetry, are shown to depend upon the aspect ratio and conformational flexibility of their constituent rectangular subunits. Computational methods allowed further design principles to be deciphered.

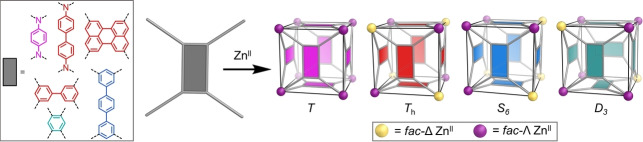

## Introduction

Metal‐organic coordination cages have been utilized for an array of applications, including catalysis,[^1^, ^2^, ^3^, ^4^, ^5^, ^6^, ^7^, ^8^, ^9^, ^10^, ^11^, ^12^] enantioselective recognition and separation,[^13^, ^14^, ^15^] and the stabilization of reactive molecules.[^16^, ^17^, ^18^, ^19^, ^20^, ^21^] The ability of a cage to be used for an application depends on its interior cavity structure in two ways. First, a high degree of enclosure is desirable to ensure that the environment provided by the interior cavity is distinct from that of the bulk solvent. Second, the size, shape and electronic properties of the cage cavity influence the efficiency and selectivity of guest binding, and the substrate binding scope.^[22]^ Thus, to expand the range of cage applications, new generations of these capsules, providing cavities with different shapes, sizes and electronic properties, are needed.

M_8_L_6_ pseudo‐cubic coordination cages, which assemble from fourfold‐symmetric *tetrakis*(bidentate) ligands and metal ions with an octahedral coordination geometry, have been shown to enclose volumes in the range of 1340–3300 Å^3^, allowing the cages to bind a wide range of guests.[^23^, ^24^, ^25^, ^26^] Furthermore, cages prepared from tetratopic subcomponents have been shown to exhibit higher stability than structures containing di‐ or tri‐topic subunits, due to the greater degree of cooperativity that holds them together.^[27]^

Subcomponents with fourfold symmetry can form square tetratopic ligands that become the faces of cubic architectures. Examples of fourfold‐symmetric subcomponent cores include porphyrins,[^23^, ^24^, ^25^, ^26^] dimolybdenum(II) “paddlewheels”,^[28]^ and metal centers with square‐planar[^29^, ^30^, ^31^] or octahedral^[32]^ coordination geometries.

Rectangular *tetrakis*(monodentate) ligands have been employed to prepare open‐ended barrel‐like structures[^33^, ^34^, ^35^, ^36^, ^37^, ^38^, ^39^, ^40^, ^41^, ^42^] and heteroleptic tetragonal prisms,[^43^, ^44^, ^45^, ^46^, ^47^, ^48^] as well as structures with gyrobifastigium,[^49^, ^50^] triangular orthobicupola[^51^, ^52^] and square orthobicupola^[53]^ geometries. The few reported pseudo‐cube architectures formed from rigid rectangular subunits[^54^, ^55^, ^56^, ^57^, ^58^, ^59^, ^60^, ^61^] have found diverse applications, ranging from novel photophysical properties^[60]^ to cascade catalysis.^[54]^ Foundational work by Duan and co‐workers used flexible *tetrakis*(tridentate) ligands in combination with Ce(NO_3_)_3_ to assemble an array of M_8_L_6_ species with different geometries, including a pseudo‐cube.[^62^, ^63^, ^64^]

**Figure 1:**
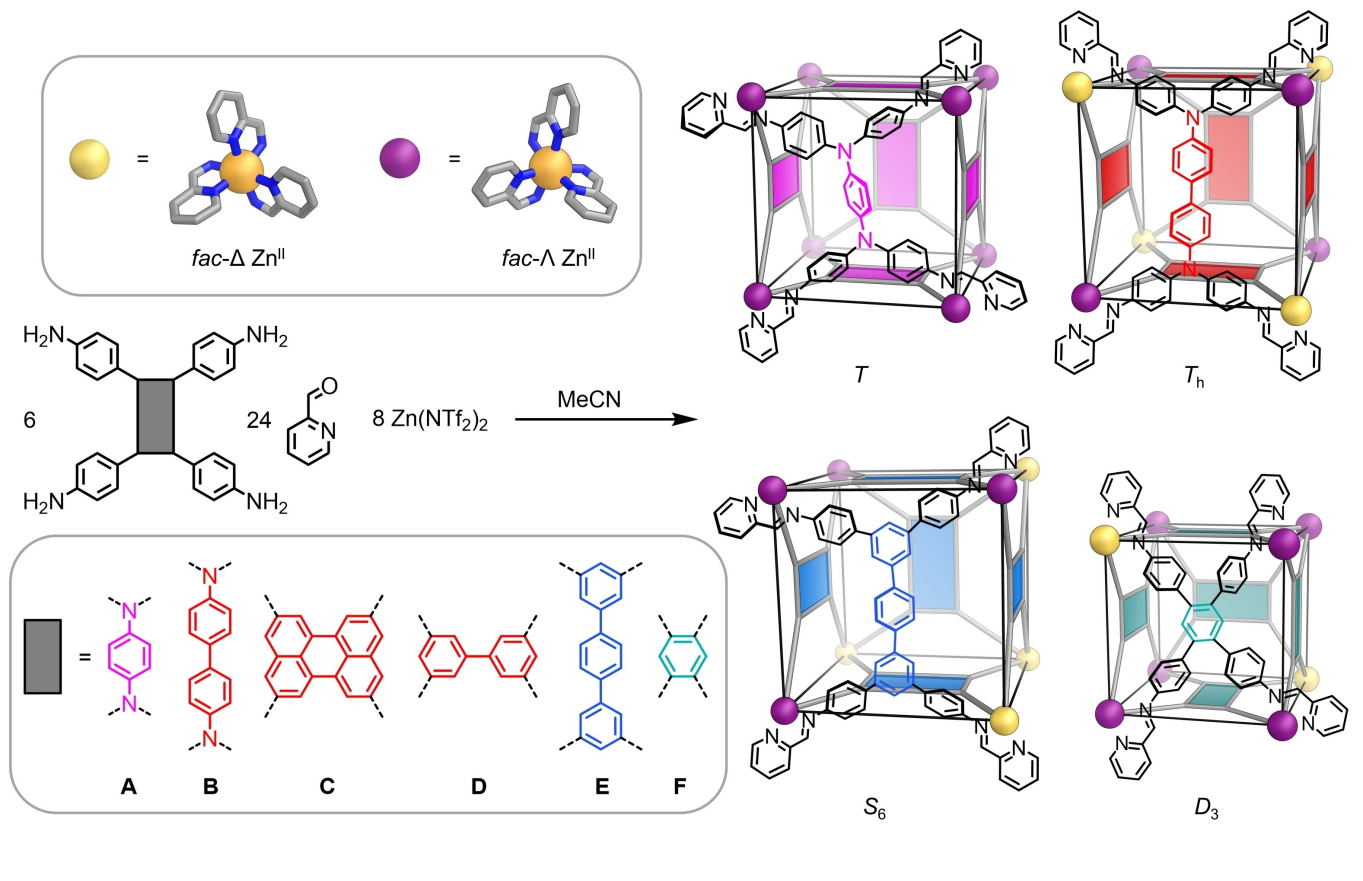
Subcomponent self‐assembly of Zn_8_L_6_ pseudo‐cubic metal‐organic cages from rectangular tetra‐anilines. The handedness of metal centers and the relative orientations of the rectangular panels determine the point symmetry of each structure.

Here we explore the formation of M_8_L_6_ pseudo‐cubic assemblies from rigid, rectangular tetra‐aniline subcomponents **A**–**F**, 2‐formylpyridine and zinc(II) *bis*(trifluoromethanesulfonyl)imide (triflimide, ^−^NTf_2_) in acetonitrile (Figure 1). Although the aspect ratios of some of these panels deviate significantly from a square geometry, all still assemble into pseudo‐cubic coordination cages, but with distinct diastereomeric configurations. Each of the products was characterized in solution by NMR spectroscopy and electrospray ionization mass spectrometry (ESI‐MS), and in the solid‐state by single‐crystal X‐ray diffraction.^[65]^

By considering the stereochemistry of the metal‐organic pseudo‐cubes, the preference of each tetra‐aniline subcomponent for a particular M_8_L_6_ diastereomer could be rationalized, allowing us to establish design rules for the formation of the pseudo‐cubes with lower symmetry. We also applied a computational workflow to judge the relative stabilities of possible diastereomers for pseudo‐cubes **1**–**6** to determine whether self‐assembly outcome prediction was viable, and to uncover design rules.

## Results and Discussion

### Synthesis and Characterization of Metal‐Organic Cages

Tetramine subcomponents **A**–**F** were either commercially available or synthesized using procedures detailed in Supporting Information Section 2. As detailed in Supporting Information Section 3, the reaction of tetramine **A**, **B**, **C**, **D**, **E** or **F** with 2‐formylpyridine and zinc(II) triflimide gave Zn_8_L_6_ pseudo‐cubes **1**, **2**, **3**, **4**,^[65]^ **5** or **6**, respectively. The [Zn_8_L_6_]^16+^ composition of each of the structures was confirmed by ESI‐MS (Figures S23, S24, S36, S37, S49, S50, S63, S64, S78 and S79), and all structures gave ^1^H diffusion‐ordered spectroscopy (DOSY) NMR spectra (Figures S21, S34, S47, S61 and S76) consistent with the formation of a single species in solution.

X‐ray quality crystals for each of the new products were obtained as detailed in Supporting Information Section 4. The solid‐state structures were determined by single crystal X‐ray diffraction using synchrotron radiation.^[66]^ Each crystal structure revealed a [Zn_8_L_6_]^16+^ assembly with pseudo‐cubic geometry. The eight *fac*‐Zn^II^ centers describe the vertices of a pseudo‐cube, and six ligands, formed from the condensation of a tetramine in the series **A**–**F** (Figure 1) with four equivalents of 2‐formylpyridine, panel the faces.^[67]^

Diastereomeric configurations were observed to differ between **1**–**6**, however. Different diastereomers of metal‐organic cages may arise as a result of the differing stereochemistry of *tris*(chelated) metal centers with an octahedral coordination geometry.[^68^, ^69^, ^70^, ^71^] Differing rotational configurations,^[72]^ or helicities,^[73]^ of capping tritopic ligands are also reported to give rise to distinct isomers, and the helical twists of individual helicate subunits^[74]^ or the relative orientations of ligand panels[^75^, ^76^] yield different diastereomers. In this study, different isomers have distinct point symmetries that emerge from the relative orientations of their panels and the metal ion stereochemistry, as detailed below.

**Figure 2:**
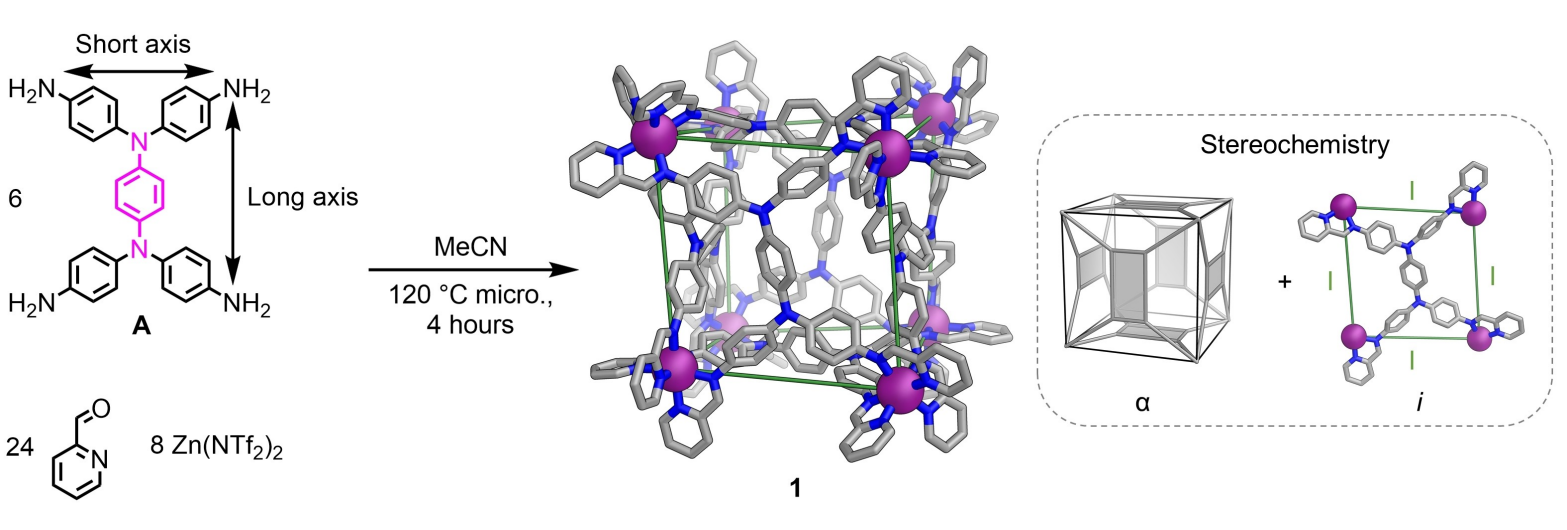
Subcomponent self‐assembly of pseudo‐cube **1**, with idealized *T* point symmetry, from **A**, 2‐formylpyridine and zinc(II) triflimide. A relative arrangement of the six rectangular subunits α was observed, meaning that the long axis of each **A** residue is oriented perpendicular to the long axes of the **A** residues paneling the four adjacent faces and parallel to the long axis of the residue paneling the opposite face. Each of the six face panels adopts facial configuration *i* (inset, right). Disorder, anions, hydrogen atoms and solvent molecules are omitted from the crystal structure of **1** for clarity. C ‐ gray, N ‐ blue, *fac*‐Λ Zn^II^ ‐ purple.

The crystal structure of **1** matches well with the structure reported by Duan and co‐workers as this work was being finished.^[59]^ Each **A** residue is oriented perpendicular to the **A** residues paneling the four adjacent faces, but parallel to the tetra‐aniline **A** residue paneling the opposite face (Figure 2), a configuration we denote “α”. When arrangement α is adopted, the short axis of each **A** residue meets the long axis of another residue at each of the twelve edges of the pseudo‐cube.^[77]^

All eight Zn^II^ centers have identical handedness, thus all six ligands adopt the facial configuration defined as *i*, shown in Figure 2 (inset, right). The ^1^H NMR spectrum of **1** (Figures S11 and S13) indicated the presence of two magnetically inequivalent ligand arms (Figure S12). Thus, both solid‐state and solution data were consistent with the formation of a pseudo‐cube with idealized *T* point symmetry. The two enantiomers of **1** co‐crystallized as a racemate.

**Figure 3:**
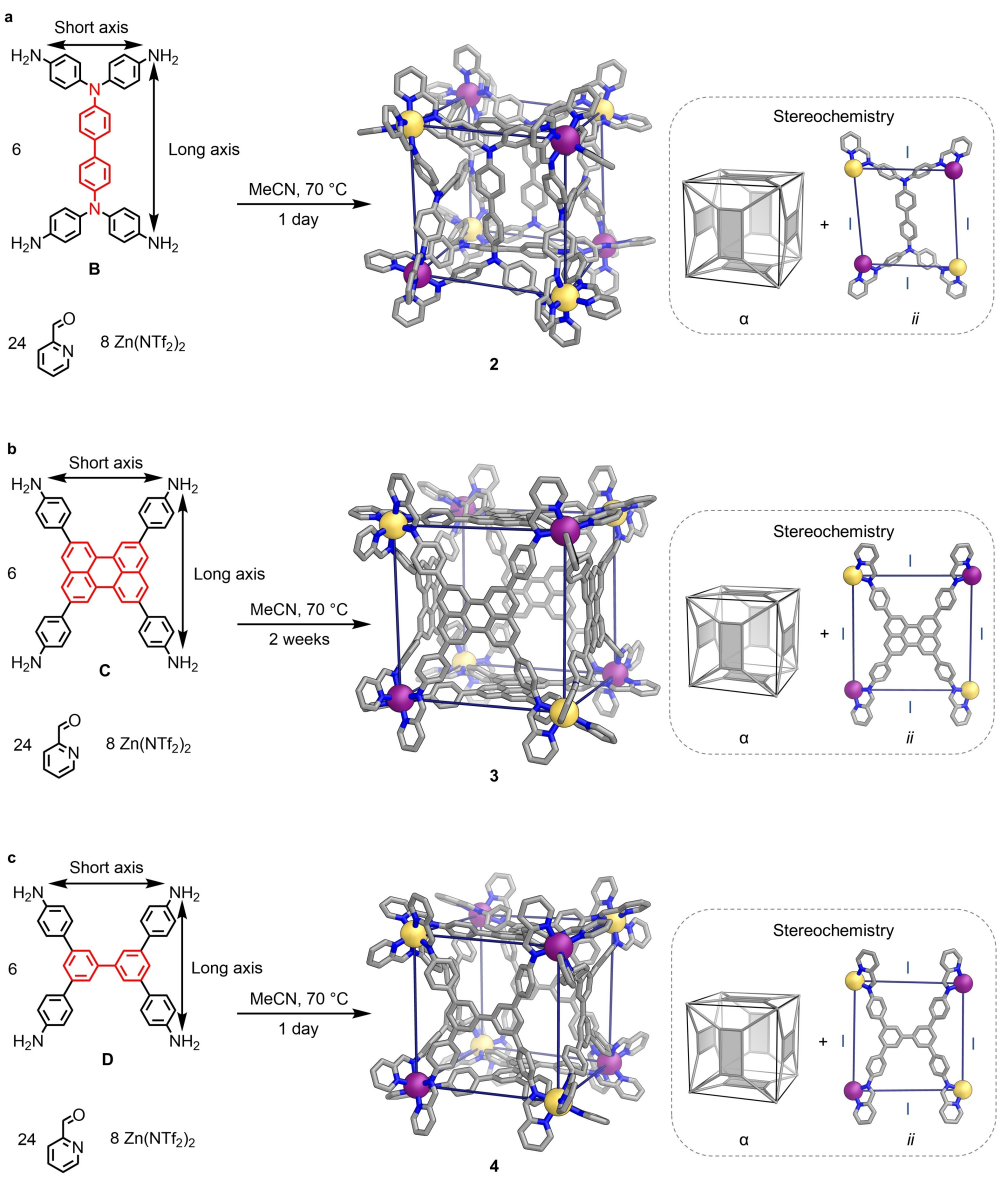
Subcomponent self‐assembly of pseudo‐cubes **2**–**4** with idealized *T*_h_ point symmetry, from the reaction of **B**, **C** or **D** ^65^ with 2‐formylpyridine and zinc(II) triflimide in acetonitrile. Each structure has an α relative orientation of the six rectangular panels (insets, left), and each face adopts configuration *ii* (insets, right). Disorder, anions, hydrogen atoms and solvent molecules are omitted from the crystal structures of **2**–**4** for clarity. C ‐ gray, N ‐ blue, *fac*‐Λ Zn^II^ ‐ purple, *fac*‐Δ Zn^II^ ‐ yellow.

The α arrangement of the six rectangular panels was also observed in the crystal structures of **2**, **3** and **4** ^65^ (Figure 3). In contrast to **1**, these structures each contain four Δ Zn^II^ centers, and four Λ Zn^II^ centers; all the nearest neighbors of a Zn^II^ center with Δ handedness have Λ handedness, and vice versa. The ^1^H NMR spectra of **2** (Figure S27), **3** (Figure S40), and **4** ^65^ indicated that all ligand arms within each structure are magnetically equivalent (Figures S26 and S39), consistent with the observation that all six ligands in each crystal structure adopt facial configuration *ii* (Figure 3), resulting in a framework with idealized *T*_h_ symmetry.

**Figure 4:**
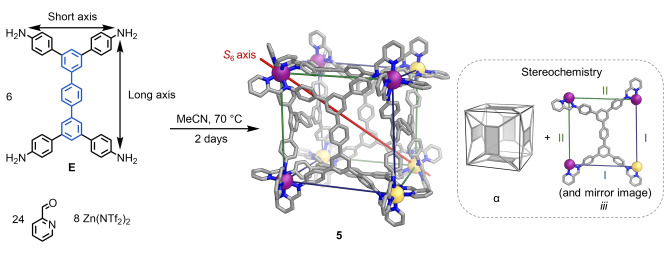
Subcomponent self‐assembly of pseudo‐cube **5**, from the reaction of **E** with 2‐formylpyridine and zinc(II) triflimide in acetonitrile. The *S*_6_ axis is shown to help visualize the idealized *S*_6_ symmetry of **5**. The different edge types within **5** are identified using different colors, and are labelled I and II, respectively, on the cut‐out of a single face (inset, right). Disorder, anions, hydrogen atoms and solvent molecules are omitted from the crystal structure of **5** for clarity. C ‐ gray, N ‐ blue, *fac*‐Λ Zn^II^ ‐ purple, *fac*‐Δ Zn^II^ ‐ yellow.

Structure **5** also contains rectangular panels in an α arrangement, and equal numbers of Zn^II^ centers with Δ and Λ handedness. However, the distribution of metal stereocenters within **5** gives rise to idealized *S*_6_ point symmetry, in contrast with the *T*_h_‐symmetric frameworks of **2**–**4**. Along the body diagonal defining the *S*_6_ axis, the eight metal centers can be separated into two groups of four, related to each other by that rotoinversion axis (Figures 1 and 4). This pattern of metal handedness is similar to the *S*_6_‐symmetric M_8_L_12_ structures reported by Ward and co‐workers.^[78]^ The ^1^H NMR spectrum of **5** (Figures S51 and S53) indicated the presence of four magnetically unique ligand arms in the structure, consistent with the solid‐state structure (Figure S52) in which all six ligands adopt facial configuration *iii*.

**Figure 5:**
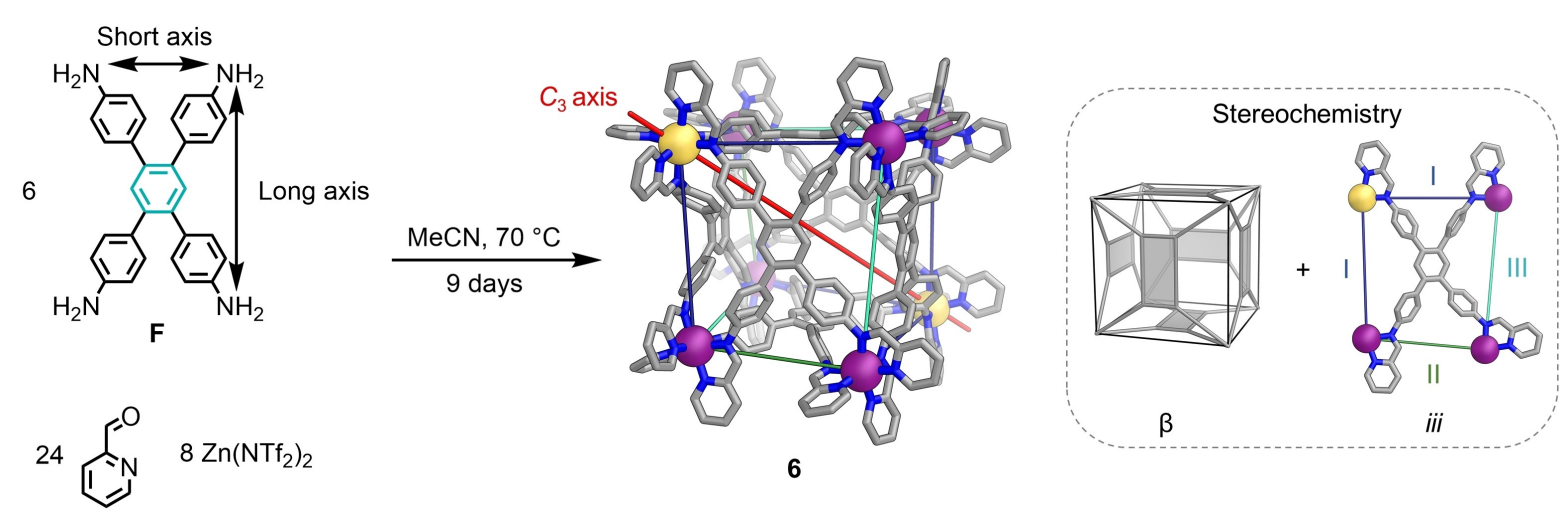
Subcomponent self‐assembly of pseudo‐cube **6**, from the reaction of **F** with 2‐formylpyridine and zinc(II) triflimide in acetonitrile. A *β* relative arrangement of the six rectangular subunits (inset, left) was observed, contrasting with the α configuration of the other pseudo‐cubes discussed herein. The non‐crystallographic *C*_3_ axis is drawn to help visualize the idealized *D*_3_ symmetry of **6**. The different edge types within **6** are identified using different colors, and are labelled I, II and III, respectively, on the cut‐out of a single face (inset, right). Disorder, anions, hydrogen atoms and solvent molecules are omitted from the crystal structure of **6** for clarity. C ‐ gray, N ‐ blue, *fac*‐Λ Zn^II^ ‐ purple, *fac*‐Δ Zn^II^ ‐ yellow.

The six rectangular panels forming the faces of a pseudo‐cube only deviate from an α configuration in the case of **6**. In **6**, the long axis of each residue of **F** is oriented perpendicular to the long axis of the moiety paneling the opposite face, perpendicular to the long axes of the residues on two of its adjacent faces, and parallel to the long axes of **F** residues paneling the remaining two adjacent faces (Figure 5). In this arrangement, which we denote “β”, the short axis of one tetra‐aniline residue meets the long axis of another residue along six of the twelve edges of the pseudo‐cube. The short axes of the tetra‐aniline residues match together at three of the six remaining edges, while the long axes of two residues meet at the other three edges. Zn^II^ centers with Δ and Λ handedness are present within **6**, but not in a one‐to‐one ratio. Each pair of antipodal Zn^II^ centers have the same handedness; three pairs have one handedness, and the fourth pair has the opposite handedness (Figures 1 and 5).

Pseudo‐cube **6** thus has idealized *D*_3_ symmetry, which emerges from the chiral β framework adopted by its panels (Figure 5). Compared with the α configuration, the β framework of **6** has fewer edges where long and short tetra‐aniline residue axes mismatch. Only two corners in **6** have a mismatch between tetra‐aniline axes at all three of the edges that converge there. Zn^II^ centers with minority handedness, Δ in the enantiomer shown in Figures 1 and 5, reside at these two corners.

The ^1^H NMR spectrum of **6** (Figures S65 and S67) indicated the presence of four magnetically distinct ligand arms. This observation is consistent with the solid‐state structure of **6** (Figure S66), in which all six ligands adopt facial configuration *iii*. Both enantiomers of **6** co‐crystallized as a racemate (Figure S80).

Examining the Zn^II^⋅⋅⋅Zn^II^ separations in the crystal structures of pseudo‐cubes **1**–**6** revealed very little variation in adjacent Zn^II^⋅⋅⋅Zn^II^ distances, except in the case of **6**. Pseudo‐cubes **1**–**4** contain Zn^II^⋅⋅⋅Zn^II^ edges of a single type (Figures 2 and 3), with little variation in absolute distances (Table S1). Two distinct types of adjacent Zn^II^⋅⋅⋅Zn^II^ separation are observed in **5**, depending on whether the pair of Zn^II^ centers spanning a given edge have the same or opposite handedness (Figure 4). The distances for each type of edge are similar (15.5 Å and 16.0 Å), however. The three edge types in **6** have different mean Zn^II^⋅⋅⋅Zn^II^ distances (Table S1); groups of four Zn^II^ centers thus form trapezoidal faces. Substantial deviations of Zn^II^⋅⋅⋅Zn^II^⋅⋅⋅Zn^II^ angles from 90° are observed for structures **1**, **2**, **5** and **6** in the solid‐state (Table S2).

The internal cavity volumes for the crystal structures of **1**–**6** were calculated to be 400 Å^3^, 424 Å^3^, 1770 Å^3^, 942 Å^3^, 1600 Å^3^, 421 Å^3^, respectively (Figure S82), using MoloVol.^[79]^ The volume enclosed by a Zn_8_L_6_ pseudo‐cubic cage with a given mean Zn^II^⋅⋅⋅Zn^II^ separation is thus much larger than that enclosed by an M_4_L_4_ tetrahedral cage with a similar mean M^II^⋅⋅⋅M^II^ distance (Figure S83), as expected from simple geometrical considerations.

Elongation of a rectangular subcomponent results in the expansion of the resulting pseudo‐cube volume, with cavity volume tracking the cube of the average Zn^II^⋅⋅⋅Zn^II^ distance between adjacent pseudo‐cube vertices (Figure S84). For example, tetra‐anilines **D**–**F** are each terminated by 1,3‐di(4‐aminophenyl)benzene units, but they have different spacers between these termini. The identity of the spacer impacts the resulting Zn^II^⋅⋅⋅Zn^II^ separations (Table S1), and thus the interior cavity volumes (Figure S82).

### Factors Driving Single Isomer Formation

In their work considering the stereoisomers of organic pseudo‐cubic cages formed from tetra‐phenylethylene (TPE)‐based panels, Cao and co‐workers show that eight distinct relative orientations of the six rectangular panels forming the faces of a pseudo‐cube are possible, two of which are enantiomers.^[80]^ Combining these eight orientations with the Δ/Λ stereochemistry of each of the eight metal centers gives an upper limit of 8×2^8^=2048 possible isomers, many of which are identical or enantiomers, however. It is thus remarkable that each of the tetra‐aniline subcomponents **A**–**F** produces only a single Zn_8_L_6_ pseudo‐cube diastereomer from this large range of possibilities. We sought to understand the design rules underpinning this exceptional selectivity.

Cubic metal‐organic structures, with idealized *O* symmetry, may form from ligands with *C*_4_ axes of symmetry, which map onto the square faces of a cube.[^23^, ^24^, ^25^, ^26^] A facial configuration analogous to *i* (Figure 2) thus results in the same metal‐metal distance along each side of the square ligand panels. For ligands formed from rectangular subcomponents **A**–**F**, facial configuration *i* would be expected to result in two distinct metal‐metal separations, if the ligand is not constrained within a pseudo‐cube framework.

**Figure 6:**
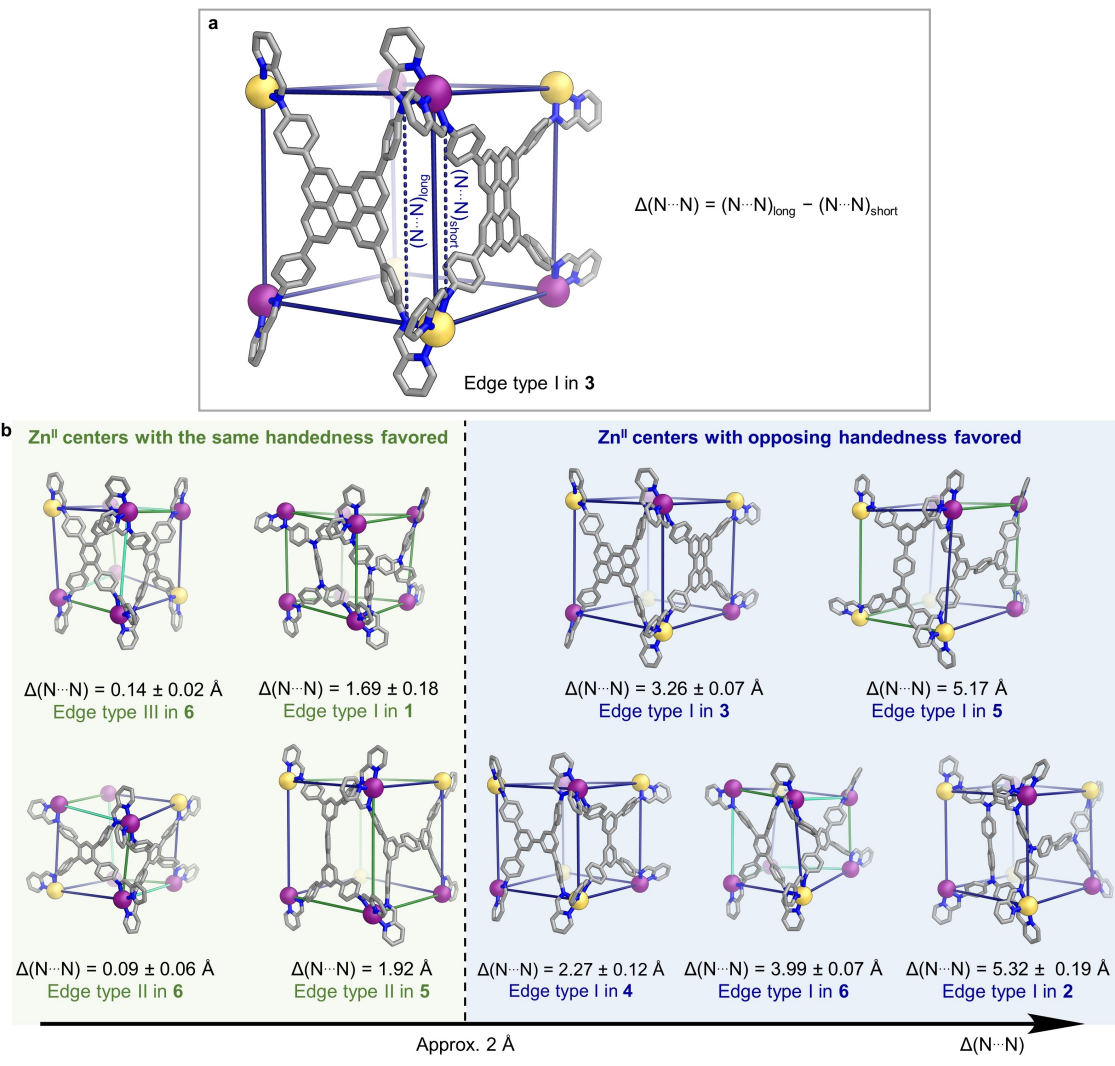
a) Representative example, **3**, showing the calculation of Δ(N⋅⋅⋅N) between the two tetra‐aniline residue sides meeting at a given edge in the Zn_8_L_6_ pseudo‐cubes. b) Analysis of the crystal structures of **1**–**6** shows a “phase boundary” of Δ(N⋅⋅⋅N)≈2 Å, below which edges containing pairs of Zn^II^ centers with the same handedness are observed, and Zn^II^⋅⋅⋅Zn^II^ pairs with opposite handedness above.

In all the pseudo‐cube structures **1**–**6**, there are edges where the short axis of one tetra‐aniline residue meets the long axis of another. At some of these edges, the two Zn^II^ centers have the same handedness. However, edges where they have opposite stereochemical configurations are also present. We thus hypothesize that in some cases, selectively inverting the handedness of a Zn^II^ center provides a better match of ideal Zn^II^⋅⋅⋅Zn^II^ distances along the two ligand axes meeting at the edge, allowing the edge to form with minimal strain (Figure 6a).

We investigated the impact of the geometric constraints imposed on the system by the tetra‐aniline upon the preference for forming edges spanned by two Zn^II^ centers with the same or opposite handedness. The preference for Zn^II^ centers with the same or opposite handedness to form a pseudo‐cube edge was quantified with respect to the difference in the distances between imine‐condensed aniline N atoms (N⋅⋅⋅N separation) along the sides of the tetra‐aniline residues meeting at that edge. In each case, we calculated the mean difference in the N⋅⋅⋅N separations (Δ(N⋅⋅⋅N)) along the sides of the two tetra‐aniline residues meeting at the same pseudo‐cube edge from N⋅⋅⋅N distances in the crystal structures of **1**–**6**. A representative example of this comparison is shown in Figure 6a.

This analysis revealed that for Δ(N⋅⋅⋅N)<2 Å, the pseudo‐cube edge forms from a pair of Zn^II^ centers with the same handedness. However, for Δ(N⋅⋅⋅N)>2 Å, the pseudo‐cube edge forms from two Zn^II^ centers with opposite handedness (Figure 6b). The opposing stereochemical configurations of the Zn^II^ centers enable strain to be dissipated by placing the short‐axis nitrogen atoms between the two Zn^II^ centers, and the long‐axis nitrogens on the outside of, or approximately in line with, the Zn^II^⋅⋅⋅Zn^II^ axis, as shown in Figure 6a.

For the high‐symmetry panel configuration α (Figures 2, 3 and 4), the strain associated with tetra‐aniline axis mismatches distributes evenly throughout the structure. While arrangement β (Figure 5) reduces the number of edges having short‐long axis mismatches, the reduced symmetry of this framework may result in localized regions of higher strain. For **6** we attribute the preference for β over α to the relatively large aspect ratio and high conformational rigidity of tetra‐aniline **F**. These features may disfavor the formation of edges at which the axes of the tetra‐aniline residues mismatch, even if the handednesses of the Zn^II^ centers act to reduce the effective difference in ideal Zn^II^⋅⋅⋅Zn^II^ separations along the different ligand lengths meeting at the edges. Although other relative arrangements of ligand panels could further reduce the number of mismatched edges, the resulting reduced framework symmetry would likely localize high strain at the remaining mismatched edges.

As tetra‐anilines **A**–**E** are anticipated to exhibit more flexibility than **F**, they may bend to dissipate some of the strain associated with the sharing of pseudo‐cube edges by ligand lengths with different ideal Zn^II^⋅⋅⋅Zn^II^ separations. We infer this bending allows the high‐symmetry relative arrangement α to be adopted.

Pseudo‐cube **5** provides an example of how the bending and flexing of a tetramine residue impacts upon the favored diastereomeric configuration. When its constituent tetra‐aniline **E** adopts a rectangular conformation, the N⋅⋅⋅N distances for its short and long axes are distinct. However, the conformations adopted by **E** residues in **5** deviate substantially from rectangular geometries. The central terphenyl cores bend notably (Figure 4), causing the four aniline‐derived N atoms in a ligand panel of **5** to describe a trapezoid, with four distinct N⋅⋅⋅N distances.

Two of the four possible short‐long combinations are observed to form selectively at the edges of **5**. In one combination, Δ(N⋅⋅⋅N) is 1.92 Å, whereas for the more extreme short‐long distance pairing, Δ(N⋅⋅⋅N) is 5.17 Å. Adoption of facial configuration *iii*, and thus a framework with *S*_6_ symmetry, enables the formation of edges by Zn^II^ centers having the same handedness when Δ(N⋅⋅⋅N) is 1.92 Å, but opposite handedness when Δ(N⋅⋅⋅N) is 5.17 Å. Therefore, we infer that the ability of **E** residues to bend is key to the emergence of an *S*_6_‐symmetric framework. As noted above, despite containing two different edge types, the structure deviates minimally from that of a regular cube, with Δ(Zn^II^⋅⋅⋅Zn^II^)=0.5 Å between its two crystallographically inequivalent edges.

The degree of conformational flexibility of a given tetra‐aniline subcomponent thus impacts upon the stereochemistry of the Zn_8_L_6_ pseudo‐cubic structure that self‐assembles. However, given that conformational flexibility manifests in different ways, it is challenging to predict how the flexibility of a subcomponent will affect the relative stability of different stereochemical configurations of the pseudo‐cube. Meeting this challenge would allow for the stereochemical configuration of a pseudo‐cube to be predicted based simply upon tetra‐aniline structure. We approached this problem computationally, as described below.

### Computational Analysis of Zn_8_L_6_ Structures

We used our Python software, the supramolecular toolkit, *stk*,[^81^, ^82^] and low‐cost optimization protocols using the UFF4MOF[^83^, ^84^, ^85^] force field in GULP[^86^, ^87^] and the semiempirical tight‐binding method GFN2‐xTB[^88^, ^89^] to construct the models herein, as detailed in Supporting Information Sections 9–15.

First, a ligand‐based geometric approach was established for assessing the relative stabilities of different diastereomers. We assessed the geometric feasibility of the seven possible edge types (Figures S92 and S93) by comparing ideal Zn^II^⋅⋅⋅Zn^II^ distances along the ligand lengths meeting at an edge (Supporting Information Section 11). The Zn^II^⋅⋅⋅Zn^II^ distances were obtained from models of different possible face configurations for each tetra‐aniline **A**–**F** (Figure S94). We infer that a small difference in ideal Zn^II^⋅⋅⋅Zn^II^ distances is likely to result in an edge with little strain, as compared with edges for which the mismatch is large.

This approach is computationally less costly than constructing full cage structures given that each cage contains more than 600 atoms and 8 metals. Models of the isolated faces do not consider the strain that results from being forced into a pseudo‐cube structure. Therefore, a discrepancy exists between the ligand conformation in the lowest‐energy pseudo‐cube configuration, and the conformation predicted by the face model (Figure S91). This method was thus not suitable for accurately predicting the single pseudo‐cube diastereomer favored by a given tetra‐aniline. However, our approach allowed general geometric principles to be deciphered. Four edge types were predicted to be geometrically feasible, while three others appeared geometrically unfavorable (Supporting Information Section 11.2).

The Δ(N⋅⋅⋅N) boundary for a switch in preference from an edge type formed by two Zn^II^ centers with the same handedness to one formed by a pair of Zn^II^ centers with opposite handedness determined via this method is in good agreement with the boundary observed experimentally (Figures 6b and S95). Our analysis of isolated cage face models also elucidated a link between the angles between aniline arms within a tetra‐aniline subcomponent, and the Δ(N⋅⋅⋅N) distance at the “phase boundary” shown in Figure 6. When the idealized angles between subcomponent arms are 60° and 120° along the short and long axes, respectively, which is the case for tetra‐anilines **C**, **D** and **F** (Figure S96), the “phase boundary” is estimated to shrink to Δ(N⋅⋅⋅N)≈1 Å (Figure S97). When these values are reversed—120° along the short axis and 60° along the long axis—as with **A**, **B** and **E**, the “phase boundary” grows to Δ(N⋅⋅⋅N)≈3 Å. Our experimental data are consistent with these boundary values (Figure S98).

Diastereomers were considered disfavored if they contained one or more geometrically unfavorable edge types. Thus, only seven diastereomers were predicted to be favored using this ligand‐based analysis of edge types, out of the fourteen we analyzed (Table S4). The assignment of favored and disfavored diastereomers is consistent with experimental observations for structures **1**–**6**, as well as other reported Zn_8_L_6_ pseudo‐cubes (Figure S99).[^54^, ^59^, ^60^]

A second computational approach attempted to improve upon a limitation of the above strategy. In this approach, the requirement for pre‐selection of the relative arrangement of rectangular panels was removed. For each of the tetra‐anilines **A**–**F** (Figure 1), we built models of the fourteen diastereomers considered above (Figures S86 and S87). The size of this data set prompted us to use a low‐cost method. Our approach (code is freely available at https://github.com/andrewtarzia/sca_cage_assembler) produced good structural correspondence with the experimental X‐ray data: Figures S103, S104 and S105 show good overlap between experimental and calculated structures, cage structural parameters, and Zn^II^⋅⋅⋅Zn^II^ distances. By examining the degree of deviation from a perfect cube (Figure S108) and relative strain (Figure S109) for different diastereomers, design rules may be deciphered.

None of the seven diastereomers that were predicted to be geometrically unfavorable using the ligand‐based approach were predicted to be the most stable diastereomer for any tetra‐aniline **A**–**F** when comparing ligand strain energies of the cage models (Supporting Information Sections 15.2.1 and 15.3.3). Although the energy differences between the most stable structures are small in some cases, ligand strain thus appears to be a useful gauge for assessing the relative stability of possible diastereomeric configurations of a given pseudo‐cube (Figure S109). For all tetra‐anilines, excepting **D** for which pseudo‐cube **4** is a kinetic product,^[65]^ the diastereomer observed was among those calculated to have the lowest strain.

Categorical prediction of which diastereomer will form for a given tetra‐aniline was not possible when modeling the cages, because, as noted above, multiple diastereomers are predicted to have similar stabilities using GFN2‐xTB calculated ligand strain. Our method nonetheless allows identification of a subset of low energy possibilities. The automated, low‐cost nature of the optimization process of the cage structures means that it can become stuck in local minima, as illustrated in Supporting Information Section 15.3.4. The impact of discrepancies that arise due to this on the trends identified and conclusions drawn were minimal, however.

Of the four diastereomers observed to be favored experimentally in this work, *D*_3_‐symmetric **6**, formed from tetra‐aniline **F** (Figure 5), deviates most from a regular cube, based on the positions of the Zn^II^ cations (Table S1 and Figure S108). Due to their high‐symmetry relative arrangement of rectangular panels, the other three diastereomers retain structures more closely resembling regular cubes, even for the most elongated ligands. The three additional diastereomers that were not observed experimentally in this work, but which were shown to be geometrically favorable using the ligand‐ and cage‐based strategies, deviate from high‐symmetry relative arrangements of rectangular panels. Their structures are thus expected to deviate from regular cubic structures to a greater extent than structures with the observed *T*‐, *T*_h_‐ and *S*_6_‐symmetric diastereomeric configurations (Figure S108).

## Conclusion

We have demonstrated that rectangular tetra‐aniline subcomponents, with differing aspect ratios and conformational flexibilities, self‐assemble into Zn_8_L_6_ pseudo‐cubic architectures. The diastereomer favored by a given tetra‐aniline subcomponent could be rationalized in light of these two parameters, enabling empirical design rules for the formation of M_8_L_6_ pseudo‐cubic coordination cages to be derived. Two distinct modes of computational examination provided insights into which diastereomers are most stable in each case, and which are disfavored. These predictions are consistent with our experimentally observed results. Furthermore, we find that examining this array of cage structures leads to design rules regarding the anisotropy of the internal cavities of the cages. In future work, specific diastereomers, with different symmetries, may be targeted to form structures with anisotropic internal volumes, which will be explored for the binding of lower‐symmetry guests. This automated computational approach for enumerating cage structures that self‐assemble from subcomponents is generalizable and open‐sourced, and we hope it may be useful to future endeavors in complex cage design.

Our method could also enable the preparation of endohedrally‐functionalized cages,[^4^, ^56^, ^90^, ^91^, ^92^, ^93^, ^94^] whose inwardly‐directed functional groups are arrayed in specific ways around central cavities. The control of framework geometry and symmetry using our method could lead to a specifically oriented functional‐group array, which may enable the binding and potentially transformation of specific guests within novel cages. Other functional groups might also be designed to respond to different stimuli,[^95^, ^96^, ^97^, ^98^, ^99^] potentially changing cage shape and enabling guest uptake or exchange to be driven.[^100^, ^101^]

We also aim to extend our methods in the future beyond pseudo‐cubes, for example, to the design of heteroleptic assemblies formed from more than one different type of ligand.[^102^, ^103^, ^104^]

## Conflict of interest

The authors declare no conflict of interest.

1

## Supporting information

As a service to our authors and readers, this journal provides supporting information supplied by the authors. Such materials are peer reviewed and may be re‐organized for online delivery, but are not copy‐edited or typeset. Technical support issues arising from supporting information (other than missing files) should be addressed to the authors.

Supporting Information

Supporting Information

Supporting Information

Supporting Information

Supporting Information

Supporting Information

Supporting Information

Supporting Information

Supporting Information

Supporting Information

Supporting Information

## Data Availability

The python code for performing the computational study is available at https://github.com/andrewtarzia/sca_cage_assembler and input and output structures are available at https://zenodo.org/record/7328029.
