# Supplementary material for: Tetramine Aspect Ratio and Flexibility Determine Framework Symmetry for Zn8L6 Self‐Assembled Structures
Source: Angew Chem Int Ed Engl. 2023 Feb 1;62(10):e202217987. doi: 10.1002/anie.202217987 (PMC10946785; doi:10.1002/anie.202217987)
Supplement: Supplementary file 1 — Supporting Information [file ANIE-62-0-s011.pdf]

Angewandte Chemie
Eine Zeitschrift der Gesellschaft Deutscher Chemiker

Supporting Information

Tetramine Aspect Ratio and Flexibility Determine Framework Symmetry for Zn_8L_6 Self-Assembled Structures

J. A. Davies, A. Tarzia, T. K. Ronson, F. Auras, K. E. Jelfs, J. R. Nitschke**

Supporting Information for:

Tetramine aspect ratio and flexibility determine framework symmetry for Zn_8L_6 self-assembled structures

Jack A. Davies[1], Andrew Tarzia[2], Tanya K. Ronson[1], Florian Auras[3], Kim E. Jelfs[2]* and Jonathan R. Nitschke[1]*

[1]Yusuf Hamied Department of Chemistry, University of Cambridge, Lensfield Road, Cambridge CB2 1EW, United Kingdom

[2]Department of Chemistry, Molecular Sciences Research Hub, Imperial College London, White City Campus, Wood Lane, London W12 0BZ, United Kingdom

[3]Department of Synthetic Materials and Functional Devices, Max-Planck Institute of Microstructure Physics, Weinberg 2, 06120 Halle, Germany

Table of Contents

1 General Information S3

2 Synthesis and characterization of tetra-aniline subcomponents S3

2.1 Subcomponent **B** S4

2.2 Subcomponent **E** S6

2.3 Subcomponent **F** S8

2.3.1 Synthesis and characterization of precursor **S3** S9

2.3.2 Synthesis and characterization of subcomponent **F** S10

3 Synthesis and characterization of Zn_8L_6 pseudo-cubic metal-organic cages S12

3.1 Synthesis and characterization of **1** S12

3.2 Synthesis and characterization of **2** S24

3.3 Synthesis and characterization of **3** S33

3.4 Synthesis and characterization of **5** S43

3.5 Synthesis and characterization of **6** S54

4 X-ray crystallography S66

5 $Zn^{II}\cdots Zn^{II}$ distance and $Zn^{II}\cdots Zn^{II}\cdots Zn^{II}$ angle measurements S73

6 Volume calculations S74

7 Software versions used for computational studies S76

8 Diastereomers to be evaluated geometrically using computed models S76

9 Tetratopic panel model construction S80

10 Metal complex model construction S81

11 Ligand-based geometric approach for assessing relative stability of diastereomers S81

11.1 Face model construction S82

11.2 Geometric feasibility of edge types S83

12 Deconstruction of subcomponent self-assembly S91

13 Library definitions S91

14 Density functional theory calculations S92

15 Cage-based modelling and geometric analysis S92

15.1 Cage construction S93

15.2 Methods for cage analysis S94

15.2.1 Ligand strain S94

15.2.2 Deviation from a perfect cube S95

15.2.3 Porosity S95

15.3 Cage analysis S95

15.3.1 X-ray structure analysis S96

15.3.2 Evaluating semiempirical and DFT relative energetics for diastereomer prediction S98

15.3.3 Evaluating structure and strain across different diastereomers S101

15.3.4 Structural output and limitations S103

16 References S105

1 General Information

Unless otherwise stated, all starting materials were sourced from commercial suppliers and used without further purification. Celite refers to AW Standard Super-Cel® NF supplied by Sigma-Aldrich. Self-assembly reactions were conducted in either MeCN-d_3 or distilled acetonitrile. For the subcomponent self-assembly of pseudo-cube **1**, a CEM Discover microwave reactor was used. For purification, acetonitrile solutions of the metal-organic cages were filtered through glass fibre. Size-exclusion chromatography was performed using Bio-Beads™ S-X3 Support, purchased from Bio-Rad Laboratories.

NMR spectra were recorded using the following NMR spectrometers: Bruker 400 MHz Avance III HD Smart Probe (routine ^{1}H NMR, ^{13}C NMR, ^{19}F NMR and ^{1}H DOSY), 500 MHz DCH Cryoprobe (High resolution ^{1}H, ^{13}C and 2D NMR experiments). Chemical shifts (δ) were reported in parts per million (ppm) for ^{1}H, ^{13}C and ^{19}F NMR spectra. Chemical shifts were referenced using the residual CD_3CN solvent signal (^{1}H = 1.94 ppm, ^{13}C = 118.26 ppm) or DMSO-d_6 solvent signal (^{1}H = 2.50 ppm, ^{13}C = 39.52 ppm). A C_6F_6 internal standard was added for ^{19}F NMR spectra, with the signal referenced at −164.38 ppm.[1] Coupling constants (J) were reported in Hz to 1 decimal place. Signal multiplicity in ^{1}H and ^{13}C NMR spectra was described using the following abbreviations: singlet (s), doublet (d), triplet (t), doublet of doublets (dd), triplet of doublets (td), doublet of doublet of doublets (ddd), multiplet (m), broad (br) and apparent (app.).

^{1}H DOSY NMR experiments were conducted on a Bruker 400 MHz Avance III HD Smart Probe spectrometer. Maximum gradient strength was 5.35 G/cm A. The standard Bruker pulse program, ledbpgp2s,[2] employing a stimulated echo and longitudinal eddy-current delay (LED) using bipolar gradient pulses for diffusion using 2 spoil gradients, was utilized. A gradient ramp of 5% to 90% was used. d20 was set to 0.1 s and p30 was optimized for each species, p30 = 1350 μs for **1**·$(NTf_2)_{16}$ and **2**·$(NTf_2)_{16}$, 1400 μs for **3**·$(NTf_2)_{16}$ and **5**·$(NTf_2)_{16}$, and 1300 μs for **6**·$(NTf_2)_{16}$.

Edited HSQC sequence results in cross-peaks for CH/CH_3 and CH_2 having opposite phases.

Low resolution electrospray ionization mass spectra (ESI–LRMS), for acetonitrile solutions of metal-organic cages, were recorded on a Micromass Quattro LC instrument (cone voltage 10 or 20 V; capillary voltage 3.8 kV; desolvation temperature 313 K; source block temperature 313 K). The sample was infused from a Harvard Syringe Pump at a rate of 10 μL per minute. High resolution electrospray ionization mass spectra (ESI–HRMS), for both cage complexes and organic compounds, were recorded on a Waters Synapt G2-Si instrument.

2 Synthesis and characterization of tetra-aniline subcomponents

Subcomponent **A** (*N,N,N',N'*-Tetrakis(4-aminophenyl)-1,4-phenylenediamine) was purchased from Carbosynth and used without further purification. Reported procedures were followed for the synthesis of subcomponent **C**.[3]

2.1 Subcomponent B

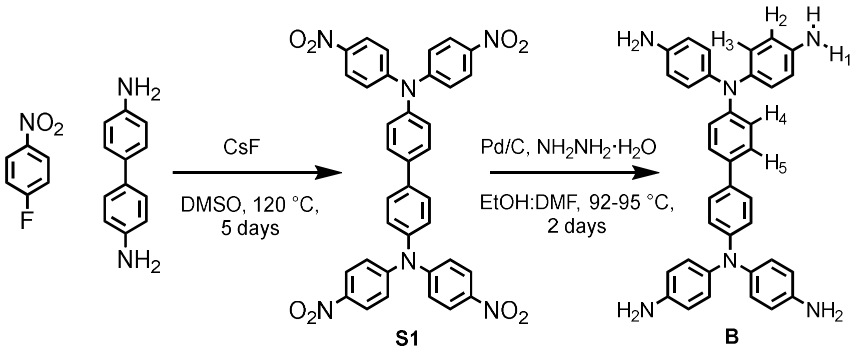

An oven-dried Schlenk flask was charged with benzidine (0.40 g, 2.2 mmol, 1.0 equiv), cesium fluoride (1.98 g, 13.0 mmol, 5.9 equiv), dried dimethyl sulfoxide (22 mL) and 4-fluoronitrobenzene (1.5 mL, 14.1 mmol, 6.4 equiv). The mixture was degassed by performing four freeze-pump-thaw cycles and the reaction mixture was heated under a nitrogen atmosphere at 120 °C for 5 days. The orange suspension was allowed to cool to room temperature and water (400 mL) was added. After 24 hours, the solid was collected by vacuum filtration and washed with water (x 4), and copious diethyl ether. The crude precursor **S1** was obtained as an orange solid and used in the following step without further purification.

A mixture of **S1** (1.00 g, 1.5 mmol, 1 equiv), ethanol (80 mL), *N,N*-Dimethylformamide (DMF) (10 mL) and hydrazine monohydrate (5 mL, 100 mmol, 67 equiv) was degassed by bubbling nitrogen for 1 hour. To the reaction mixture, 10 wt% Pd/C (370 mg) was added, the mixture was degassed by bubbling nitrogen for 1 hour, and heated at 92 °C under a nitrogen atmosphere for 24 hours. A solution of hydrazine monohydrate (3 mL, 62 mmol, 41 equiv) in DMF (5 mL) was degassed by bubbling nitrogen for 45 mins and added to the reaction mixture at room temperature. The reaction mixture was heated at 95 °C under a nitrogen atmosphere for 17 hours. The reaction mixture was allowed to cool to room temperature and water (1-2 L) added. The resulting solid was collected via decanting the supernatant, and centrifugation of the supernatant. The solid was washed with copious water, and DMF was added to the solid. The DMF mixture was passed through a celite plug and the DMF was removed *in vacuo*. The solid was washed with diethyl ether and water several times. The product **B** was obtained as a very dark green solid (0.66 g, 1.20 mmol, 80%).

^{1}H NMR (400 MHz, DMSO-d_6, 298 K) δ 7.26 (d, J = 8.8 Hz, 4H), 6.81 (d, J = 8.5 Hz, 8H), 6.63 (d, J = 8.7 Hz, 4H), 6.53 (d, J = 8.6 Hz, 8H), 4.96 (s, 8H).

^{13}C NMR (101 MHz, DMSO-d_6, 298 K) δ 148.0, 145.4, 136.0, 129.9, 127.2, 126.0, 117.2, 114.8.

ESI-HRMS Found *m/z* = 548.2676 $[M]^+$, $C_{36}H_{32}N_6$ requires m/z = 548.2688.

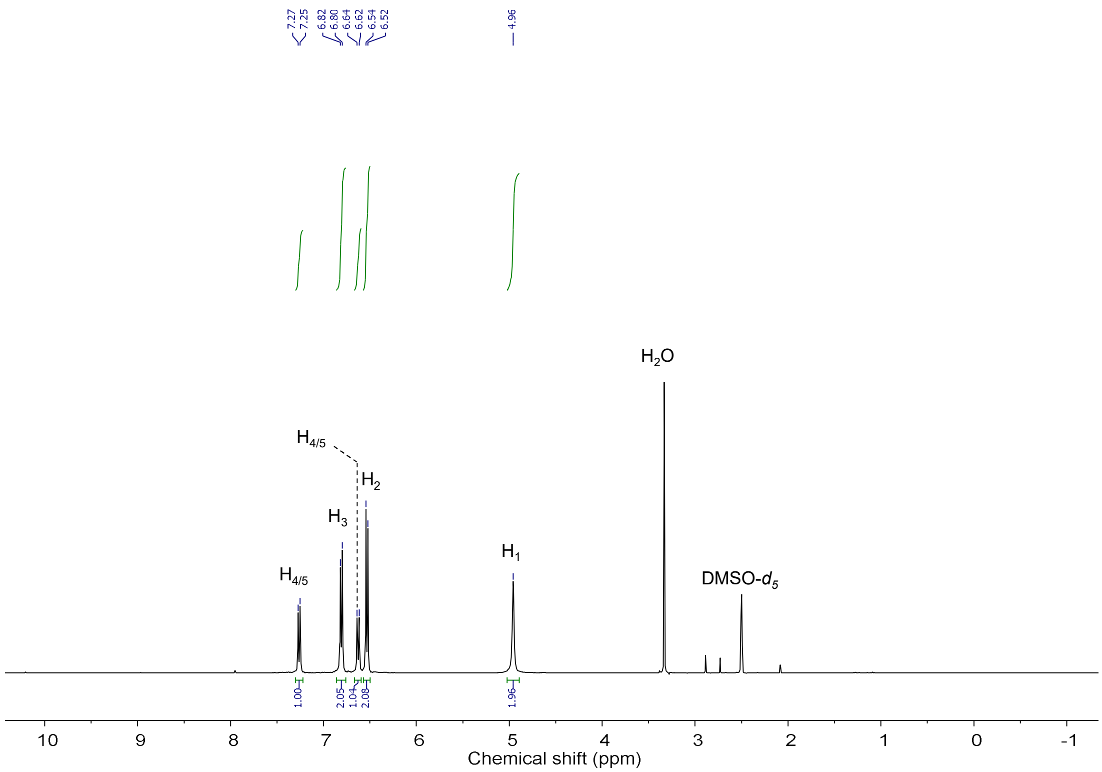

Figure S1. ^{1}H NMR spectrum (400 MHz, DMSO-d_6, 298 K) of **B**.

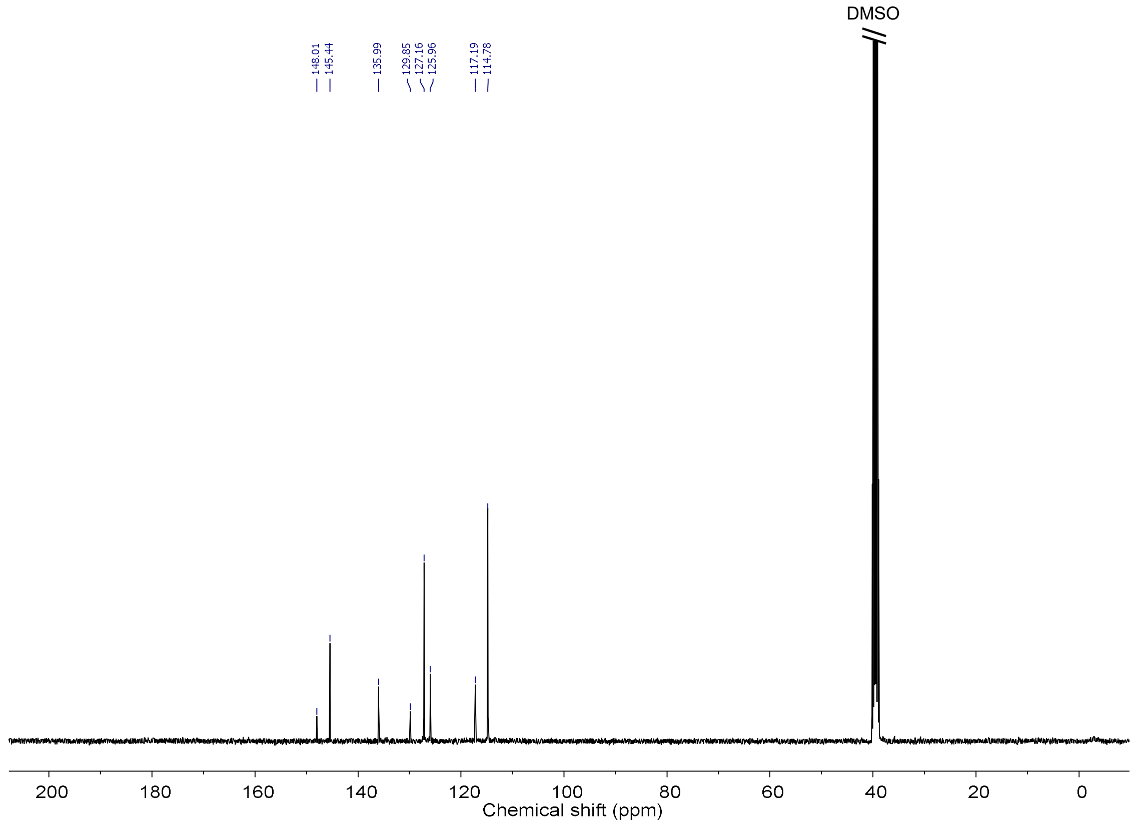

Figure S2. ^{13}C NMR spectrum (101 MHz, DMSO-d_6, 298 K) of **B**.

2.2 Subcomponent E

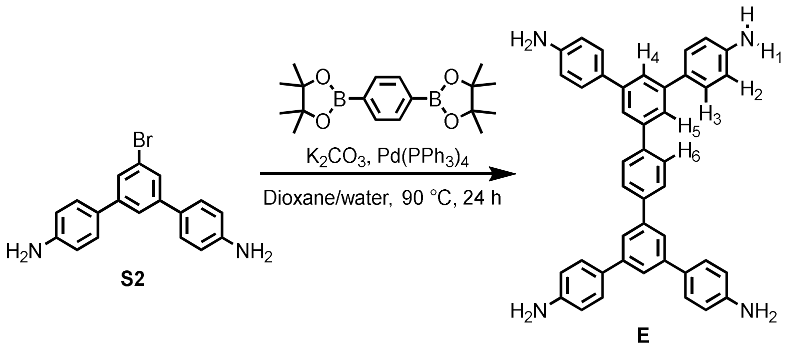

Precursor **S2** was synthesized according to a reported procedure.[4]

To **S2** (1.00 g, 2.9 mmol, 2.2 equiv) and 1,4-bezenediboronic acid bis(pinacol) ester (0.44 g, 1.3 mmol, 1.0 equiv) in 1,4-dioxane (28 mL), potassium carbonate (0.76 g, 5.5 mmol, 4.2 equiv) in water (5 mL) was added. The mixture was degassed by bubbling nitrogen for approximately 1 hour under stirring. *Tetrakis*(triphenylposphine)palladium(0) (0.17 g, 0.15 mmol) was added and the mixture was further degassed by bubbling nitrogen for approximately 1 hour under stirring. The reaction mixture was heated at 90 °C under a nitrogen atmosphere for 24 hours. The reaction mixture was allowed to cool to room temperature and the solvent was removed *in vacuo*. Ethyl acetate was added and the mixture was passed through a celite plug. The organic layer was washed with water (x 2), brine (x 1) and water (x 3), dried over anhydrous magnesium sulfate and the solvent was removed *in vacuo*. The solid was washed with diethyl ether (×2), hexane (×2) and diethyl ether (×2), and recrystallized from acetone/hexane. The solid was collected via filtration and washed with hexane to give product **E** as a pale yellow solid (0.34 g, 570 μmol, 44%).

^{1}H NMR (400 MHz, DMSO-d_6, 300 K) δ 7.91 (s, 4H), 7.68 (s, 4H), 7.66 (s, 2H), 7.54 (d, J = 8.1 Hz, 8H), 6.70 (d, J = 8.1 Hz, 8H), 5.25 (s, 8H).

^{13}C NMR (126 MHz, DMSO-d_6, 298 K) δ 148.5, 141.9, 140.8, 139.9, 127.7, 127.6, 127.5, 122.0, 121.5, 114.2.

ESI-HRMS Found m/z = 594.2789 [M]$^+$, $C_{42}H_{34}N_4$ requires m/z = 594.2783.

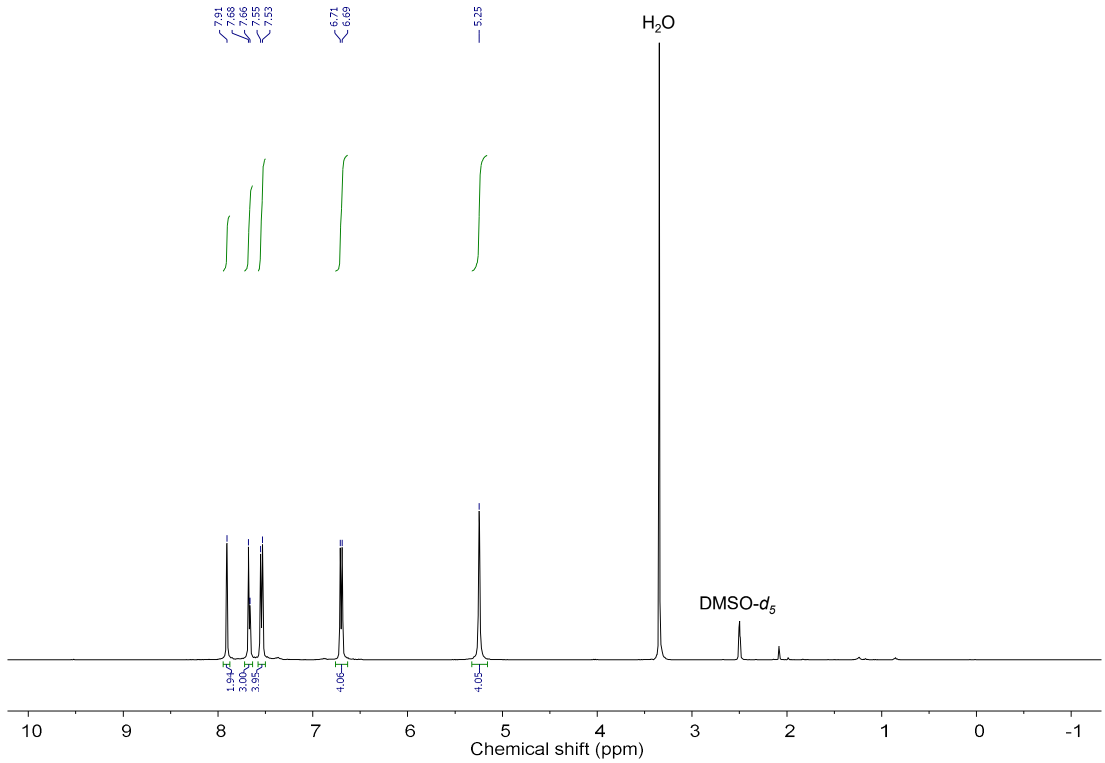

Figure S3. ^{1}H NMR spectrum (400 MHz, DMSO-d_6, 300 K) of **E**.

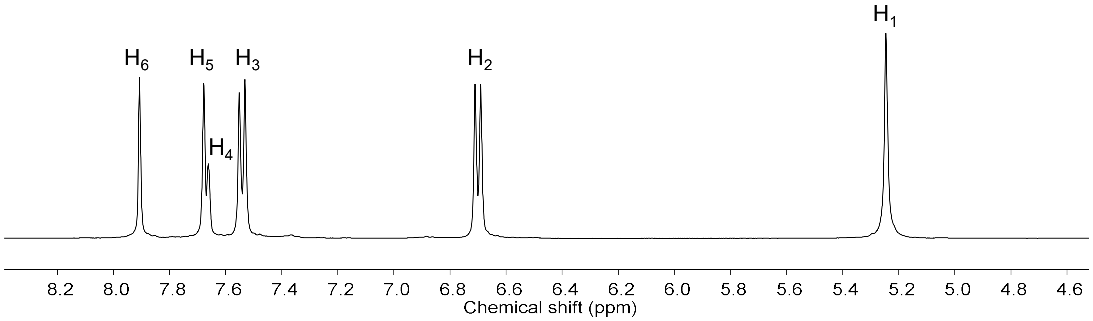

Figure S4. Aromatic region of the ^{1}H NMR spectrum (400 MHz, DMSO-d_6, 300 K) of **E**.

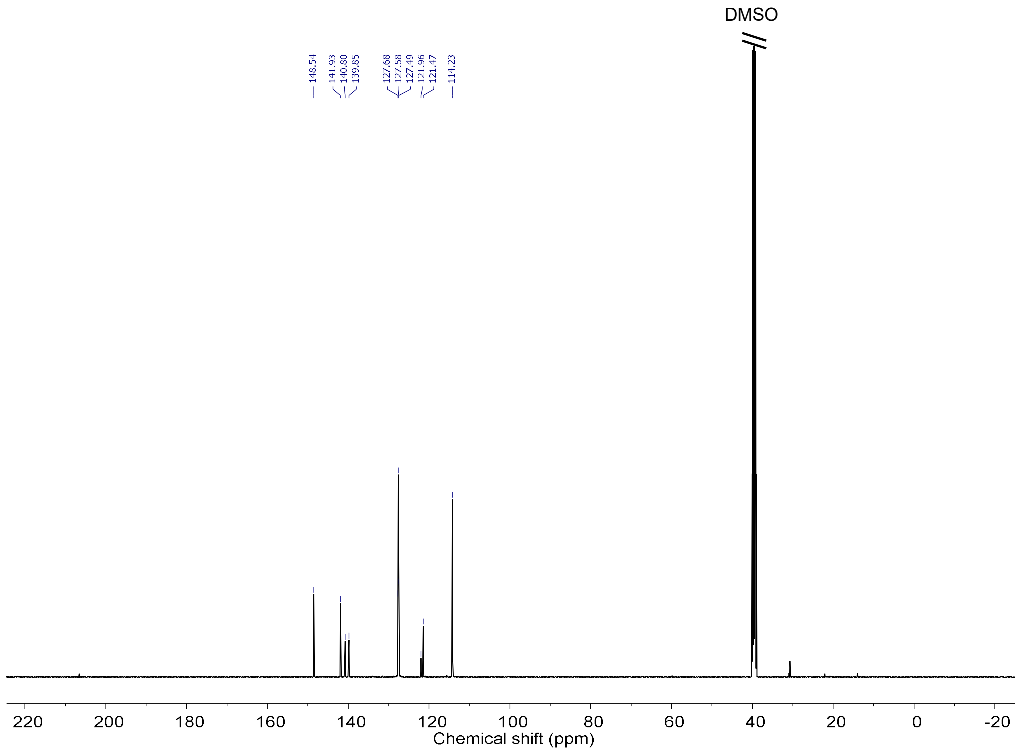

Figure S5. ^{13}C NMR spectrum (126 MHz, DMSO-d_6, 298 K) of **E**.

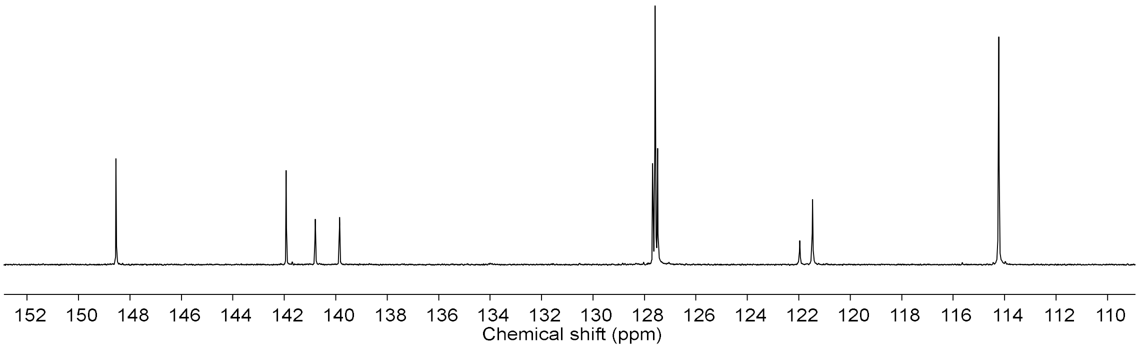

Figure S6. Aromatic region of the ^{13}C NMR spectrum (126 MHz, DMSO-d_6, 298 K) of **E**.

2.3 Subcomponent F

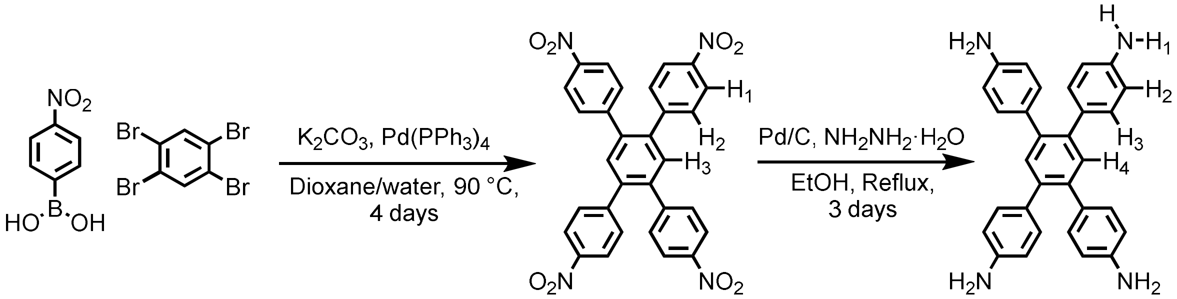

2.3.1 Synthesis and characterization of precursor S3

To 1,2,4,5-tetrabromobenzene (1.12 g, 2.8 mmol, 1.0 equiv) and 4-nitrophenylboronic acid (2.06 g, 12.3 mmol, 4.4 equiv) in dioxane (65 mL), potassium carbonate (1.90 g, 13.7 mmol, 4.9 equiv) in water (15 mL) was added. The mixture was degassed by bubbling nitrogen for 1 hour under stirring. *Tetrakis*(triphenylphosphine)palladium(0) (0.70 g, 0.61 mmol) was added and the mixture was further degassed by bubbling nitrogen for 1 hour under stirring. The reaction mixture was heated at 90 °C under a nitrogen atmosphere for 4 days. The reaction mixture was allowed to cool to room temperature and the solvent removed *in vacuo*. *N,N*-Dimethylformamide (DMF) was added and the mixture was passed through a Celite plug. DMF was removed *in vacuo* and the solid was washed with diethyl ether, water and then copious amounts of diethyl ether. The product **S3** was obtained as an orange/brown solid, and used without further purification (1.63 g, quant.).

^{1}H NMR (500 MHz, DMSO-d_6, 298 K) δ 8.18 (d, *J* = 8.9 Hz, 8H), 7.74 (s, 2H), 7.56 (d, *J* = 8.9 Hz, 8H).

^{13}C NMR (126 MHz, DMSO-d_6, 298 K) δ 146.7, 146.0, 138.5, 133.0, 131.2, 123.6.

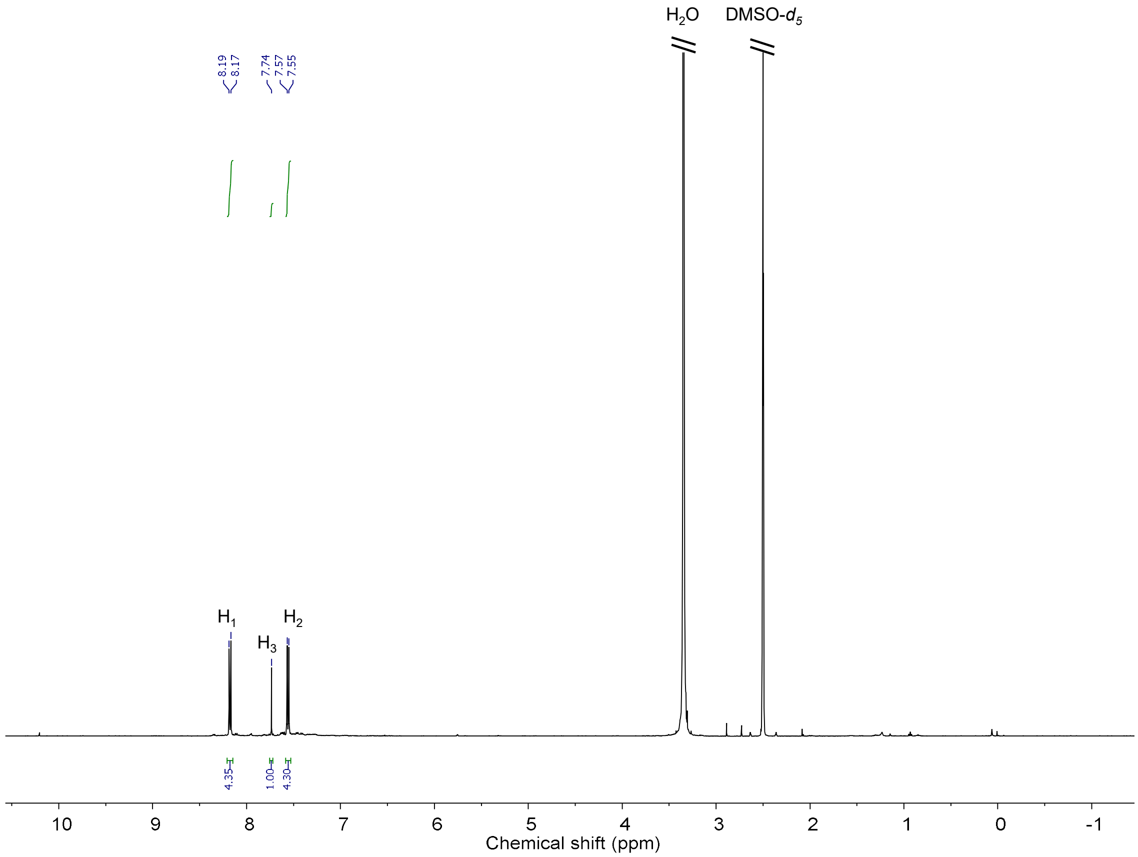

Figure S7. ^{1}H NMR spectrum (500 MHz, DMSO-d_6, 298 K) of **S3**.

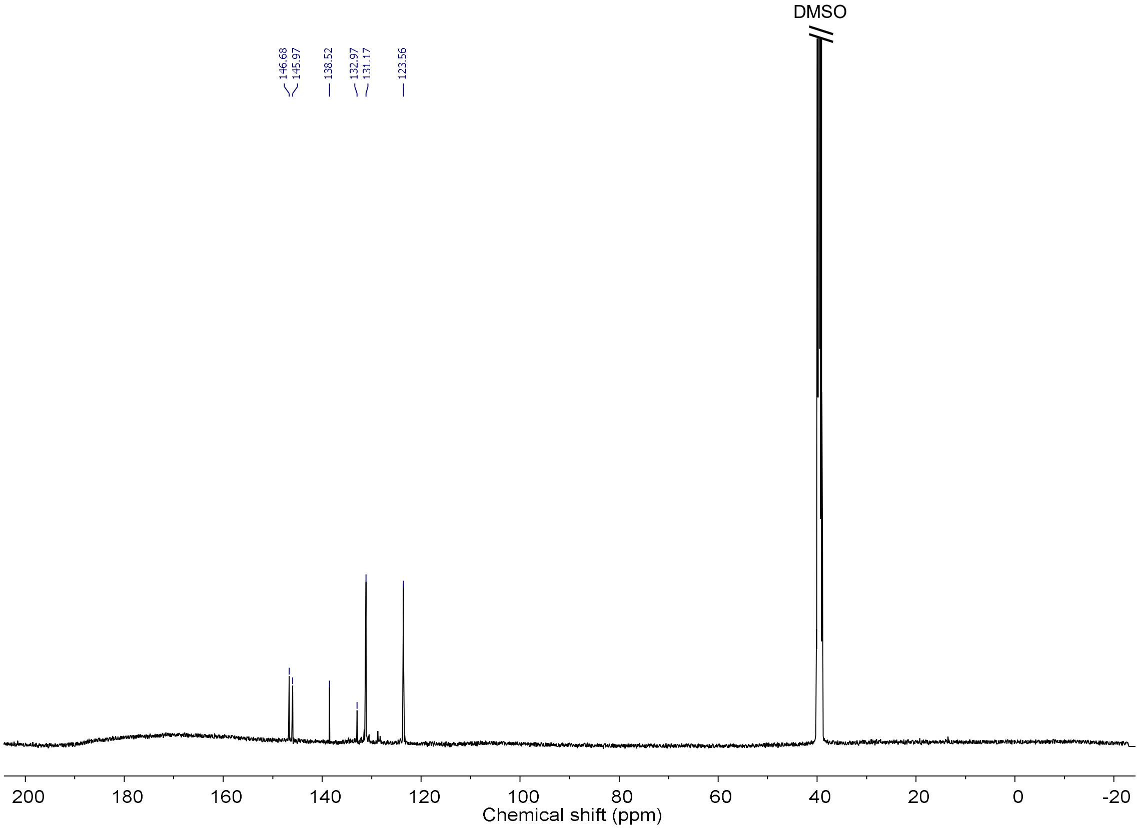

Figure S8. ^{13}C NMR spectrum (126 MHz, DMSO-d_6, 298 K) of **S3.**

2.3.2 Synthesis and characterization of subcomponent F

A mixture of **S3** (205 mg, 360 µmol, 1 equiv), ethanol (22 mL) and hydrazine monohydrate (1 mL, 21 mmol, 58 equiv) was degassed by bubbling nitrogen for 30 minutes under stirring. To the reaction mixture, 10 wt% Pd/C (79 mg) was added, the mixture was degassed by bubbling nitrogen for 30 minutes under stirring and then heated under reflux for 71 hours. The reaction mixture was allowed to cool to room temperature, passed through a celite plug, and the celite plug was washed with copious water. The celite plug was washed with *N,N*-Dimethylformamide (DMF) to retrieve the product. To the DMF eluate, water was added to induce precipitation. The precipitate was collected by vacuum filtration and washed with ethanol to give **F** as a pale yellow precipitate (79 mg, 180 µmol, 50%).

^{1}H NMR (500 MHz, DMSO-d_6, 298 K) δ 7.10 (s, 2H), 6.83 (d, J = 8.5 Hz, 8H), 6.43 (d, J = 8.5 Hz, 8H), 4.98 (s, 8H).

^{13}C NMR (101 MHz, DMSO-d_6, 298 K) δ 147.0, 138.0, 131.9, 129.9, 128.8, 113.6.

ESI-HRMS Found m/z = 442.2147 [M]$^+$, $C_{30}H_{26}N_4$ requires m/z = 442.2157.

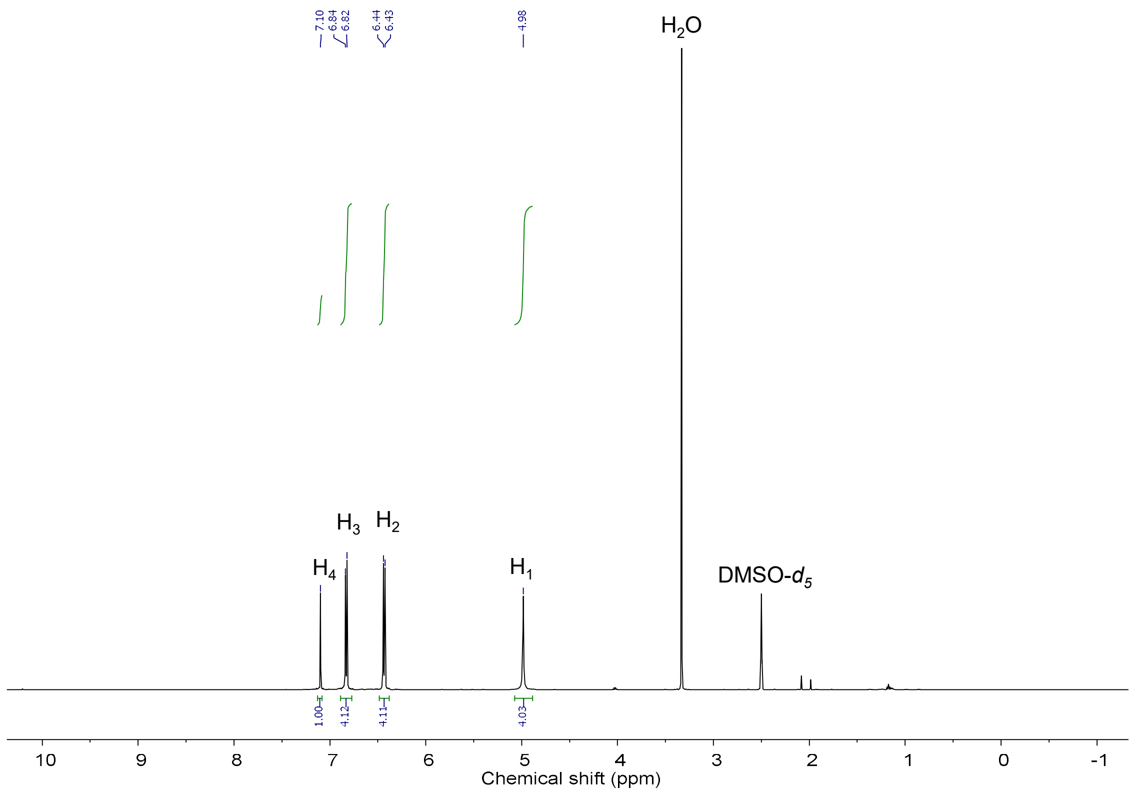

Figure S9. ^{1}H NMR spectrum (500 MHz, DMSO-d_6, 298 K) of **F**.

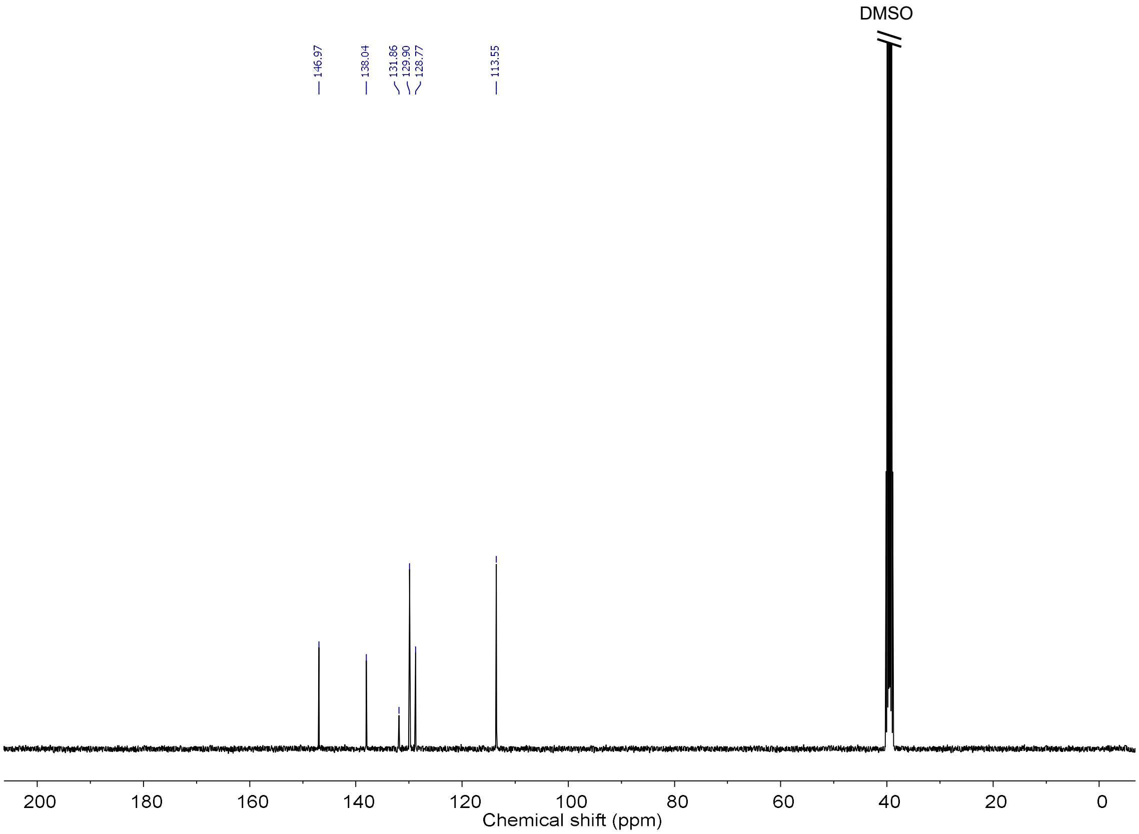

Figure S10. ^{13}C NMR spectrum (101 MHz, DMSO-d_6, 298 K) of **F**.

3 Synthesis and characterization of Zn_8L_6 pseudo-cubic metal-organic cages

4·$(NTf_2)_{16}$ was synthesized following a previously reported procedure.[5]

3.1 Synthesis and characterization of 1

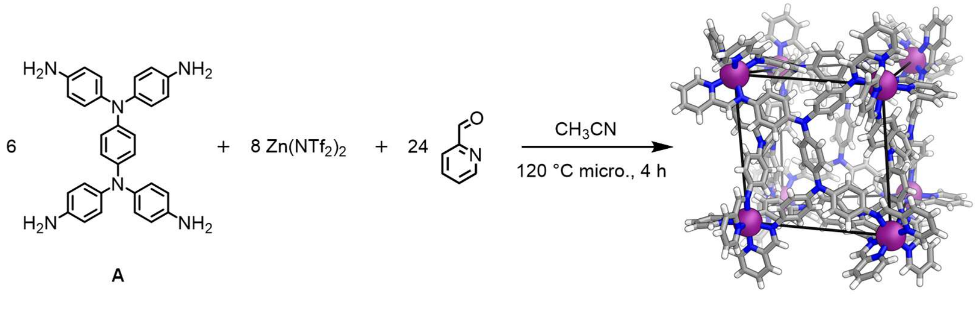

1

Scheme S1. Subcomponent self-assembly of **1**·$(NTf_2)_{16}$.

To a mixture of subcomponent **A** (5.1 mg, 11 µmol, 1.0 equiv) and $Zn(NTf_2)_2$ (9.4 mg, 15 µmol, 1.4 equiv) in distilled acetonitrile (2.0 mL), 2-formylpyridine (4.4 µL, 46 µmol, 4.2 equiv) was added. The reaction mixture was heated at 120 °C for 4 hours in a microwave reactor. The reaction mixture was filtered through a glass fibre plug and concentrated to a small volume using a stream of nitrogen. The

crude was purified by size-exclusion chromatography (acetonitrile:dichloromethane = 1:1). The solvent was removed using a stream of nitrogen to obtain **1**·$(NTf_2)_{16}$ as a very dark orange solid (12.0 mg, 1.2 µmol, 65%).

^{1}H NMR (500 MHz, CD_3CN, 298 K) δ 8.63 (s, 12H), 8.61 (s, 12H), 8.38–8.33 (m, 24H), 8.17 (d, *J* = 7.9 Hz, 12H), 8.15 (d, *J* = 7.9 Hz, 12H), 7.81 (d, *J* = 4.8 Hz, 12H), 7.79–7.74 (m, 24H), 7.66 (d, *J* = 4.9 Hz, 12H), 7.06 (s, 24H), 6.92 (d, *J* = 8.8 Hz, 24H), 6.81 (d, *J* = 8.8 Hz, 24 H), 6.77 (d, *J* = 8.8 Hz, 24H), 6.47 (d, *J* = 8.8 Hz, 24H).

^{13}C NMR (126 MHz, CD_3CN, 298 K) δ 162.7, 162.4, 149.9, 149.8, 148.8, 148.0, 147.4 (2 x ^{13}C), 143.3, 143.3, 142.9, 142.6, 142.1, 131.6, 131.5, 131.3, 131.3, 127.1, 126.2, 124.5, 124.3, 123.5, 120.8 (q, *J* = 321 Hz, NTf_2).

^{19}F NMR (376 MHz, CD_3CN, 298 K) δ −79.84.

ESI-LRMS ([**1**$(NTf_2)_{16}$] = $C_{324}H_{240}N_{60}Zn_8(C_2F_6NO_4S_2)_{16}$) *m/z* = 717.9 [**1**$(NTf_2)_6$]$^{10+}$ (calc. 717.8), 828.8 [**1**$(NTf_2)_7$]$^{9+}$ (calc. 828.7), 967.5 [**1**$(NTf_2)_8$]$^{8+}$ (calc. 967.3), 1145.7 [**1**$(NTf_2)_9$]$^{7+}$ (calc. 1145.5), 1383.4 [**1**$(NTf_2)_{10}$]$^{6+}$ (calc. 1383.1), 1716.4 [**1**$(NTf_2)_{11}$]$^{5+}$ (calc. 1715.7).

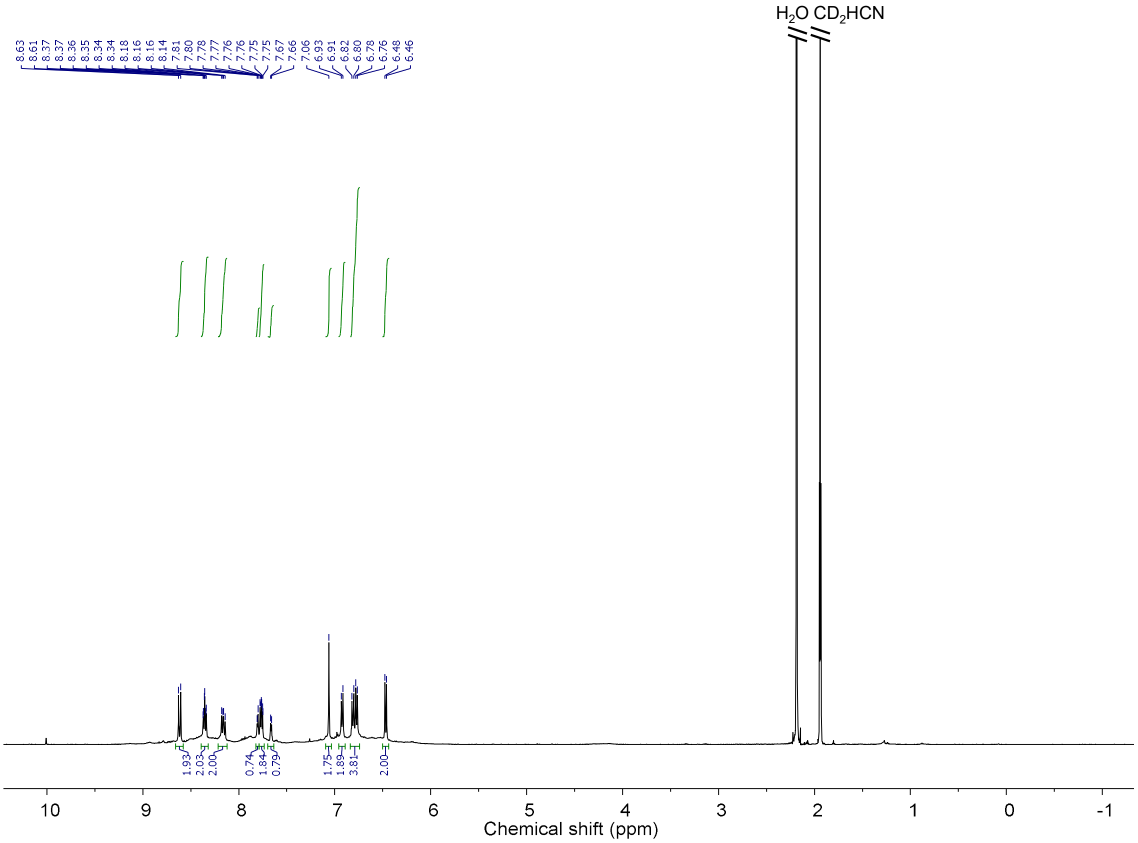

Figure S11. ^{1}H NMR spectrum (500 MHz, CD_3CN, 298 K) of **1**·$(NTf_2)_{16}$.

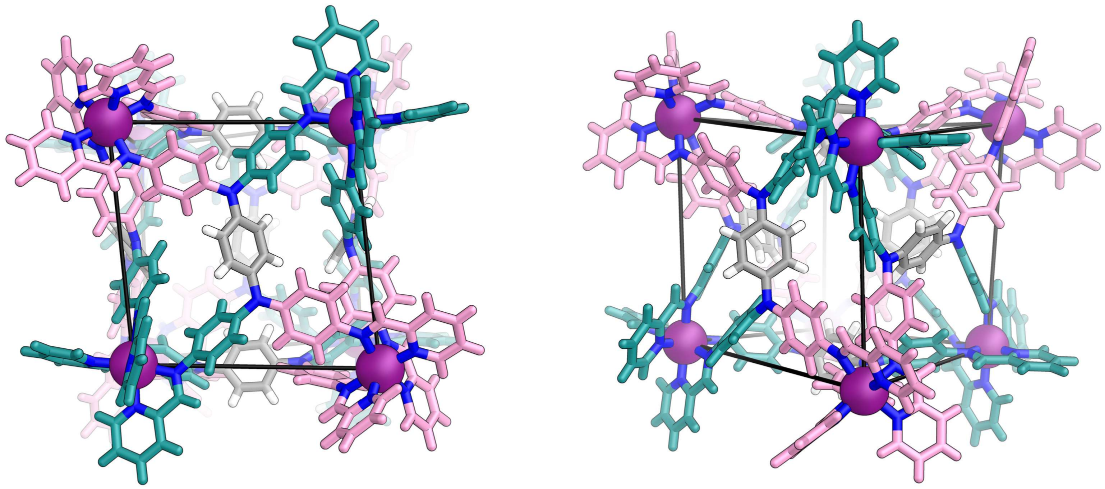

Figure S12. Views of the crystal structure of **1** with the two magnetically distinct ligand arms colored differently. (This is assuming idealized *T* point symmetry, not all ligand arms colored the same are crystallographically equivalent).

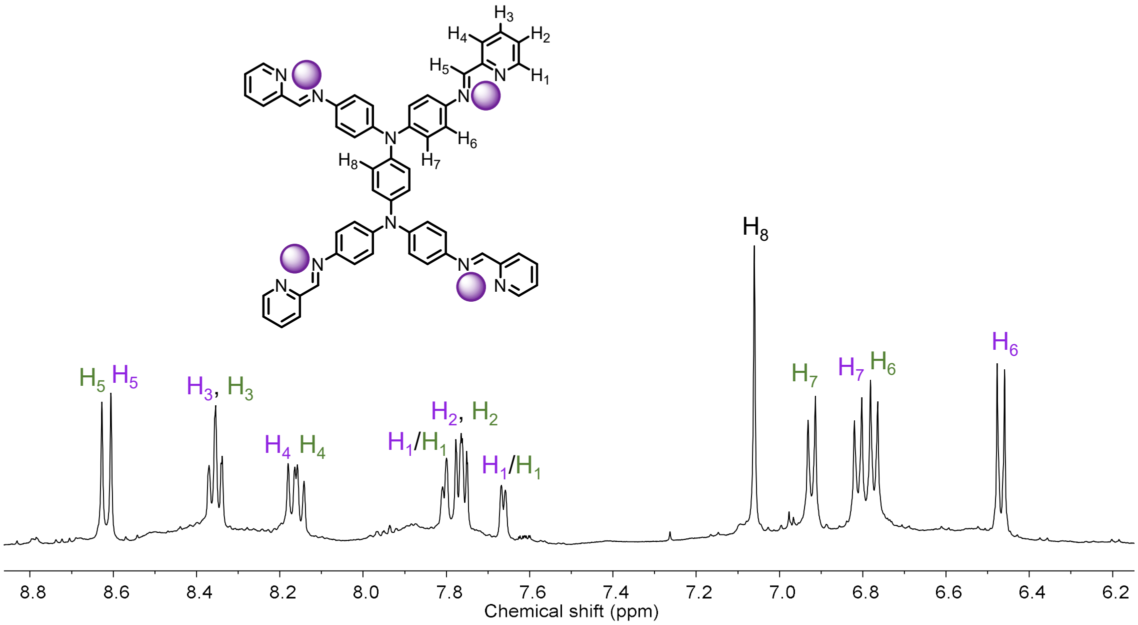

Figure S13. Aromatic region of the ^{1}H NMR spectrum (500 MHz, CD_3CN, 298 K) of **1**·$(NTf_2)_{16}$, with assignment of signals. The signals for each unique ligand arm could be identified and are labeled with different colors; however, each set could not be conclusively assigned to a specific arm in the structure. The absence of the splitting of the H_8 signal was attributed to rapid rotation of the central phenyl ring of **A** residues on the NMR timescale. This rotation results in rapid exchange between the two proton environments, giving a single averaged signal.

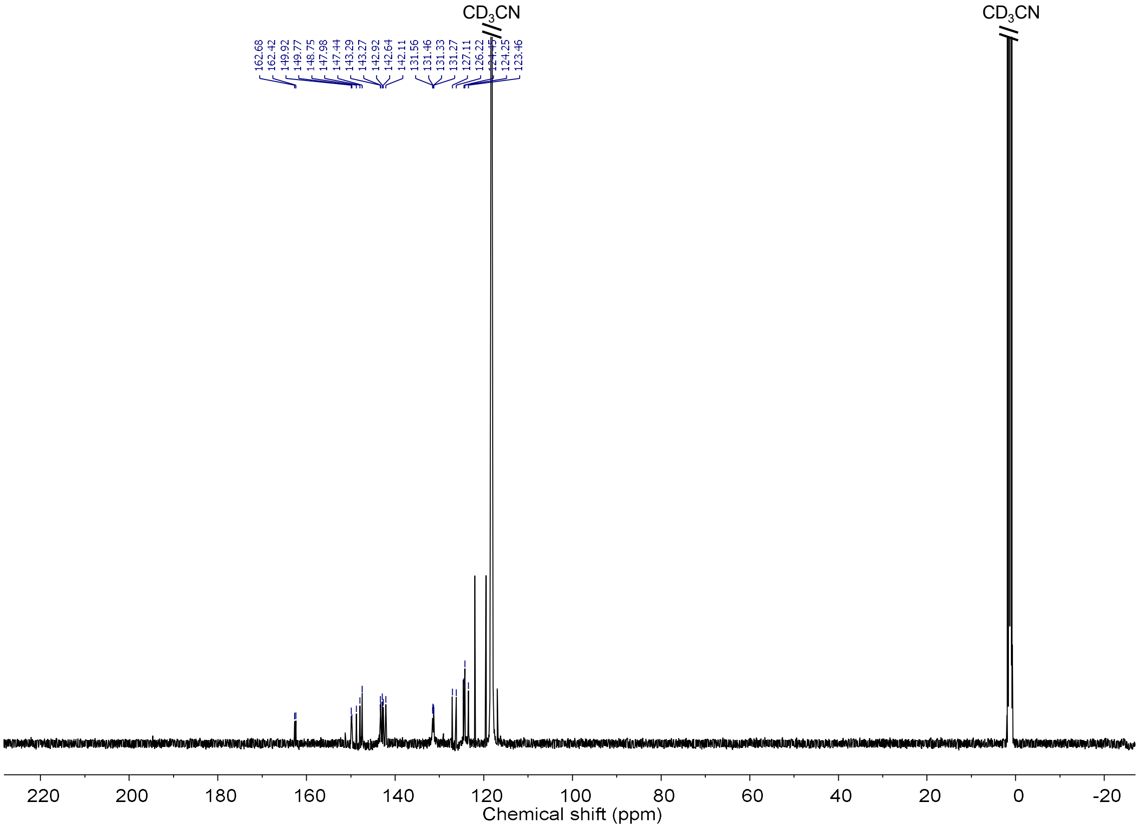

Figure S14. ^{13}C NMR spectrum (126 MHz, CD_3CN, 298 K) of **1**·$(NTf_2)_{16}$.

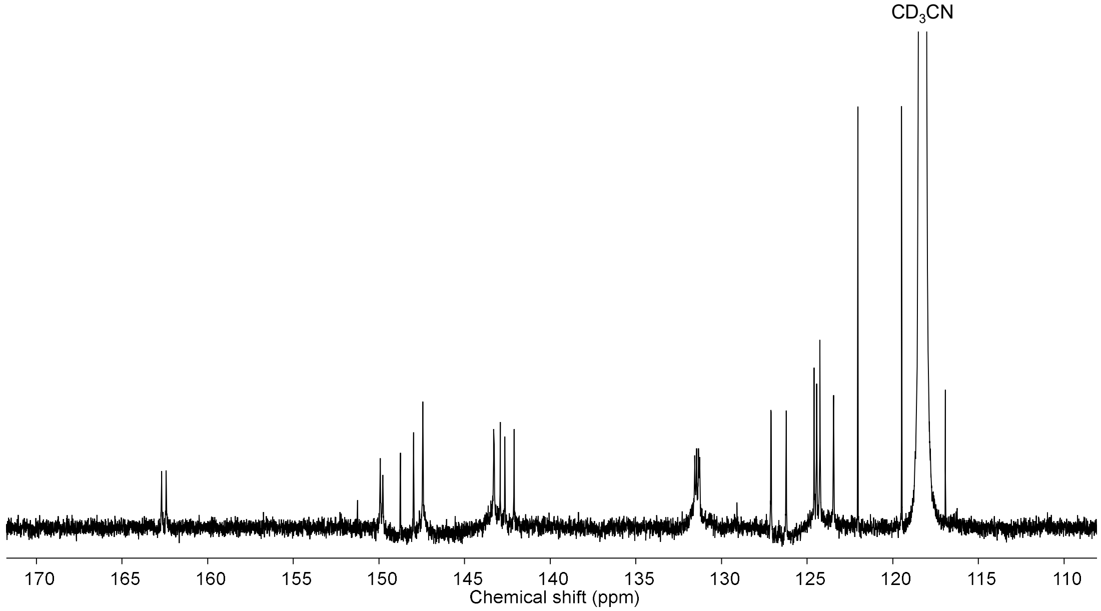

Figure S15. Aromatic region of the ^{13}C NMR spectrum (126 MHz, CD_3CN, 298 K) of **1**·$(NTf_2)_{16}$.

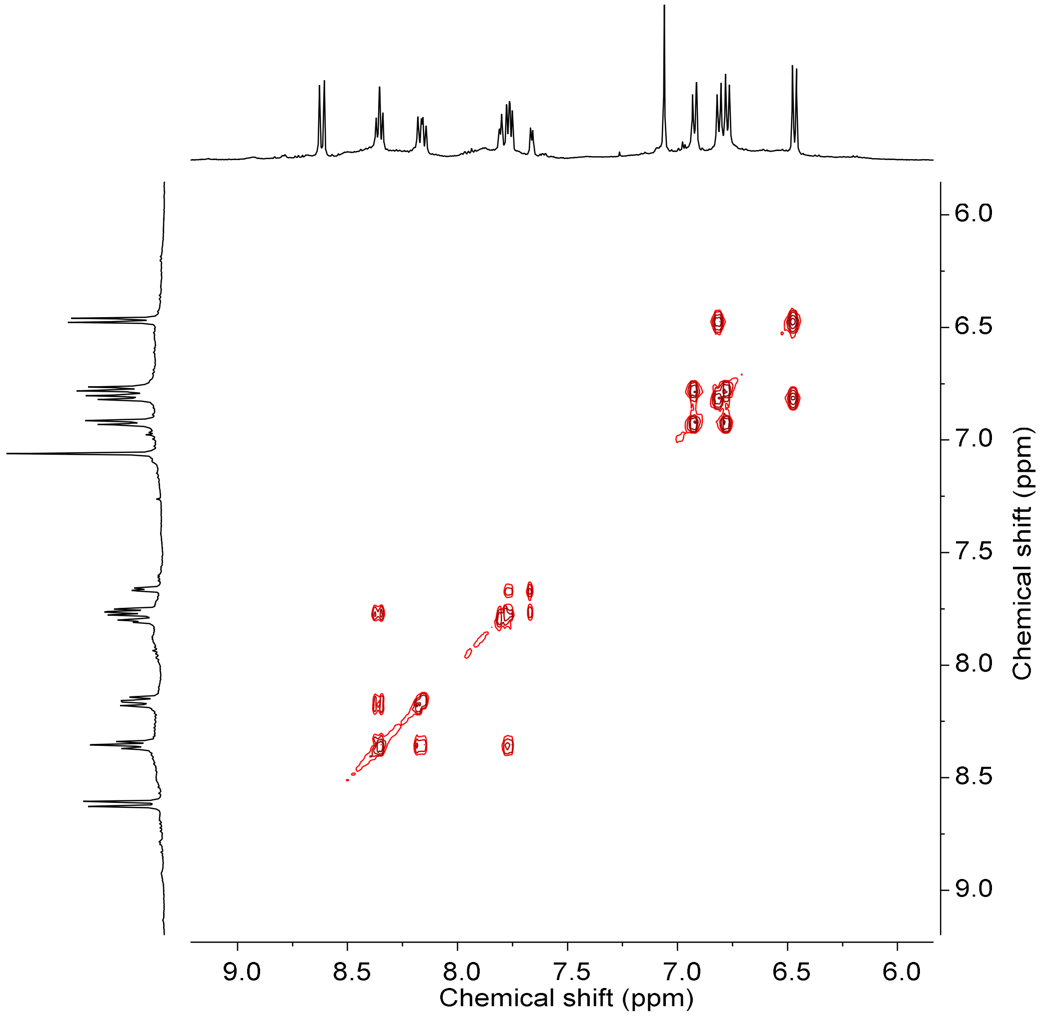

Figure S16. Aromatic region of the ^1H-^1H DQF-COSY spectrum (500 MHz, CD_3CN, 298 K) of **1**·$(NTf_2)_{16}$.

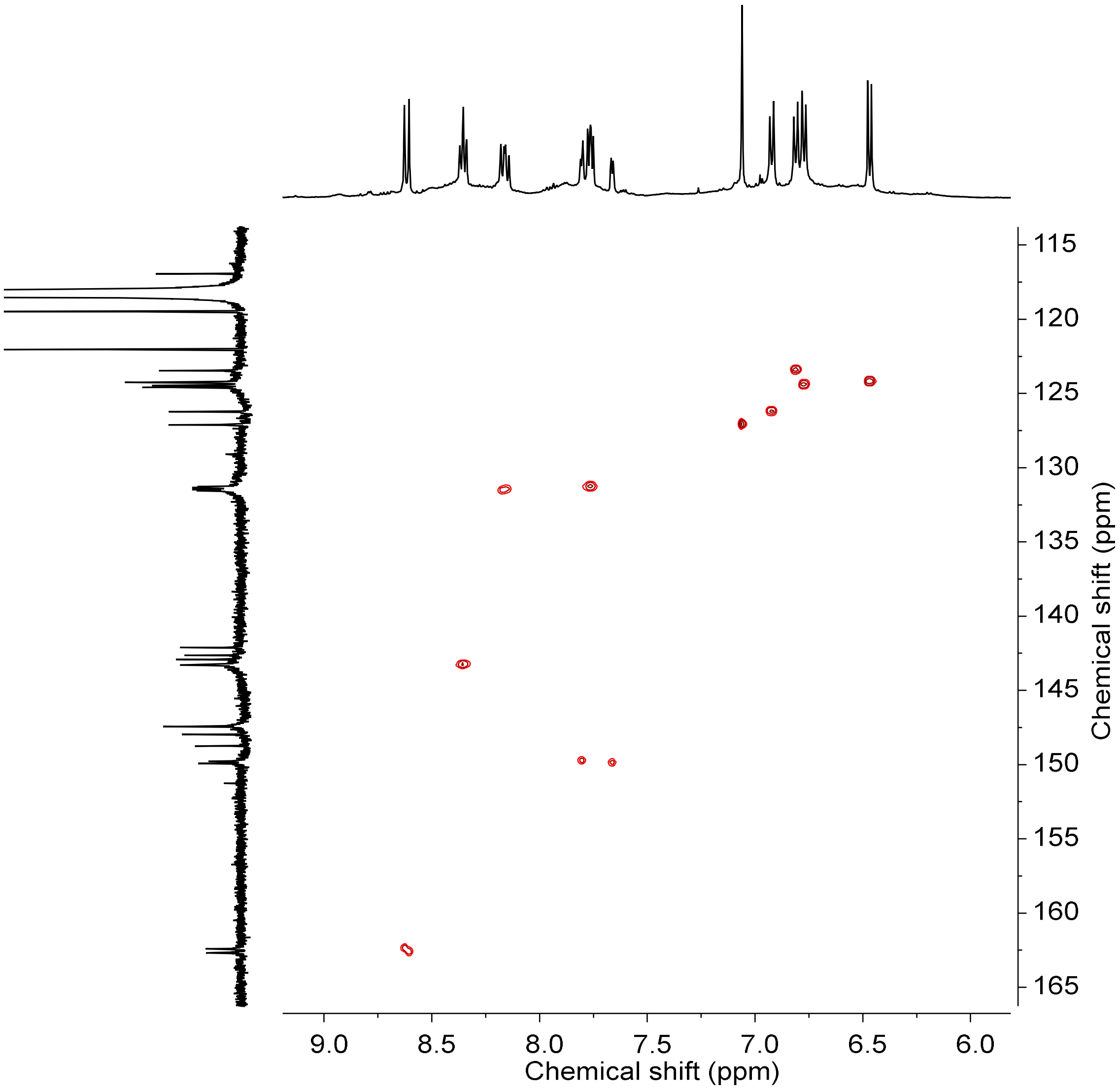

Figure S17. Aromatic region of the ^{1}H-^{13}C edited HSQC spectrum (500 MHz, CD_3CN, 298 K) of **1**·$(NTf_2)_{16}$.

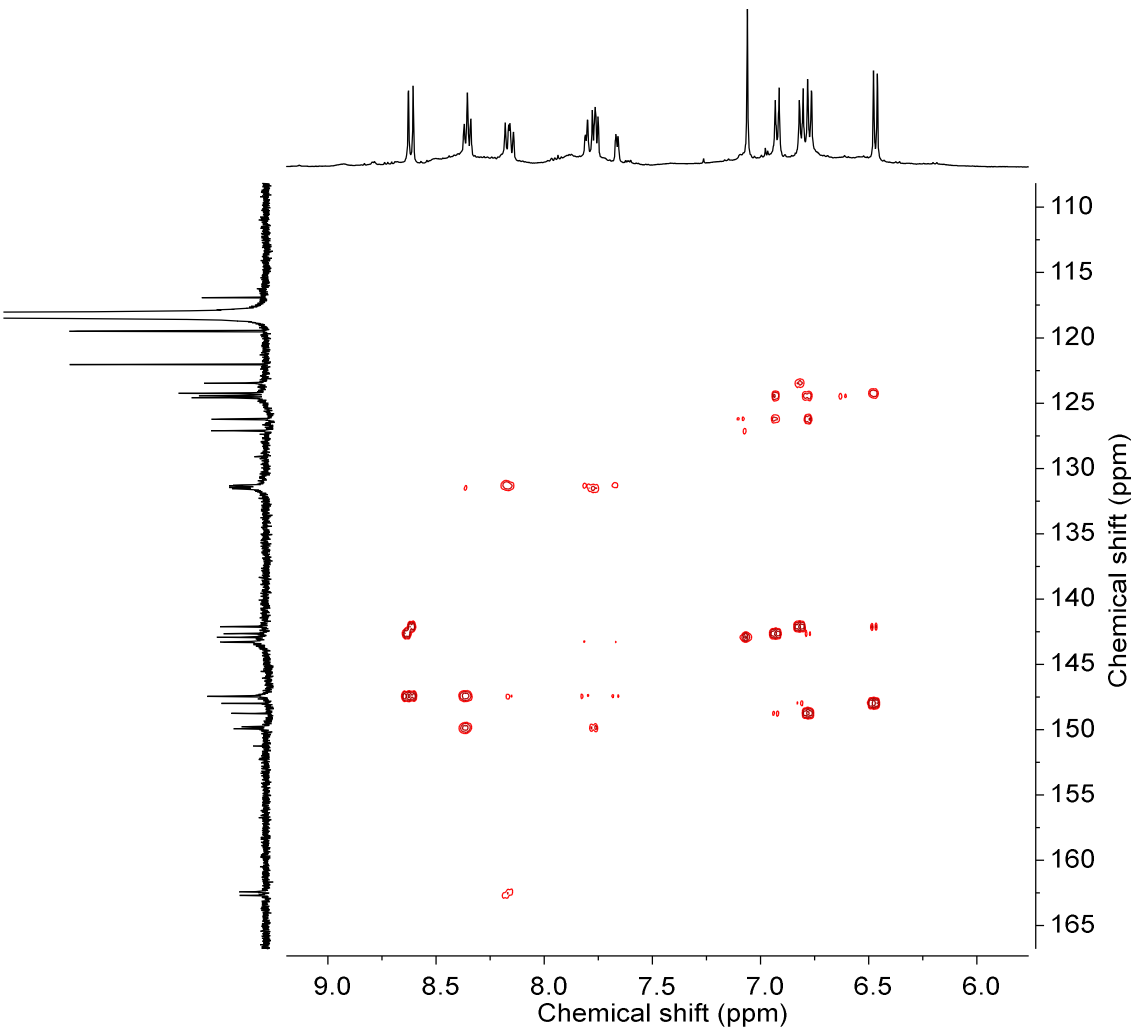

Figure S18. Aromatic region of the ^{1}H-^{13}C HMBC spectrum (500 MHz, CD_3CN, 298 K) of **1**·$(NTf_2)_{16}$.

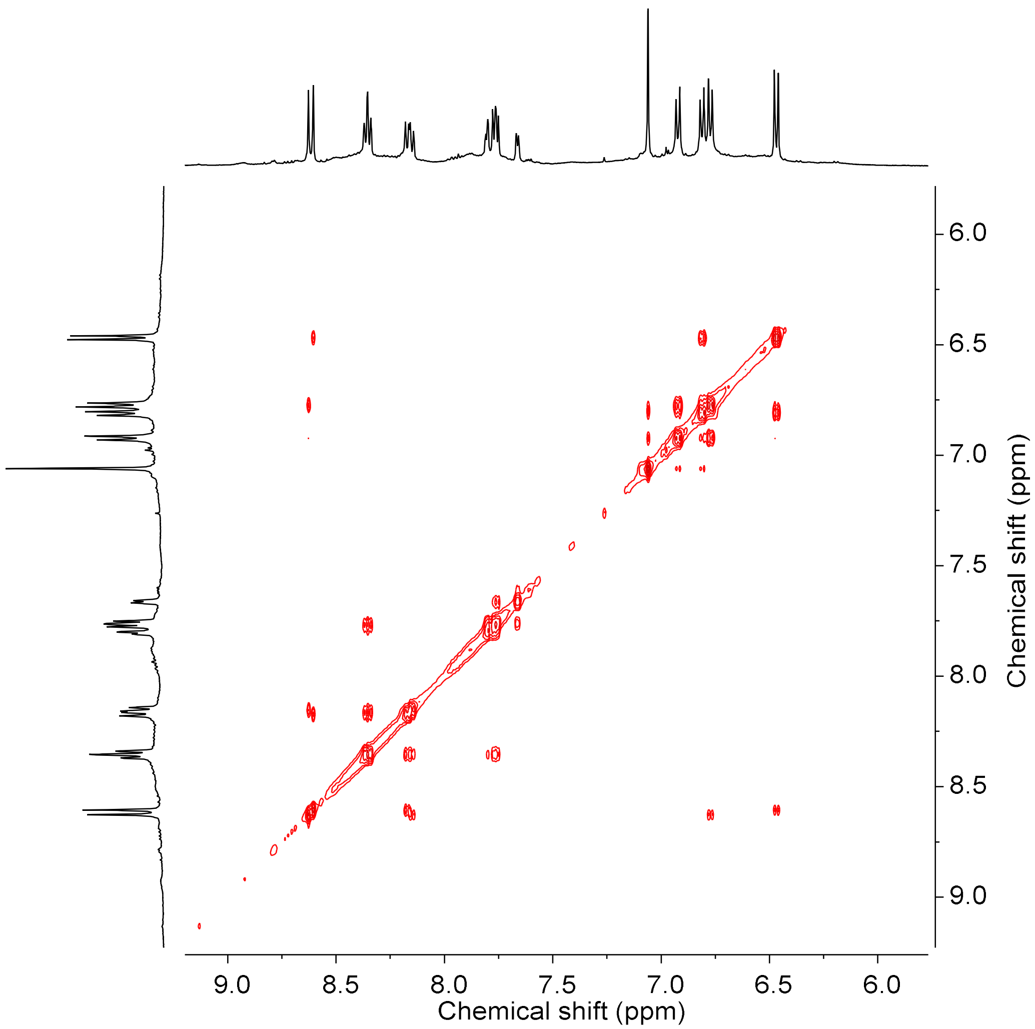

Figure S19. Aromatic region of the ^{1}H-^{1}H NOESY spectrum (500 MHz, CD_3CN, 298 K) of **1**·$(NTf_2)_{16}$.

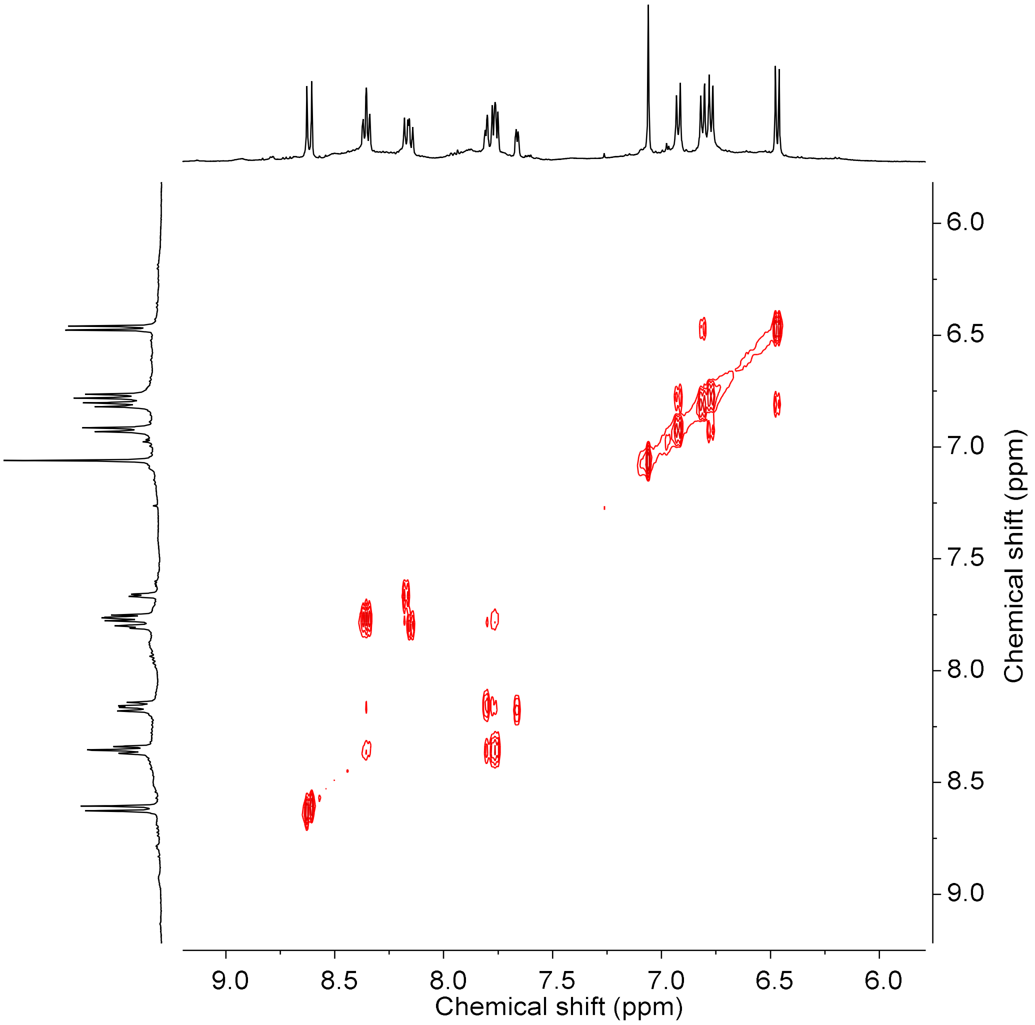

Figure S20. Aromatic region of the ^1H-^1H TOCSY spectrum (500 MHz, CD_3CN, 298 K) of **1**·$(NTf_2)_{16}$.

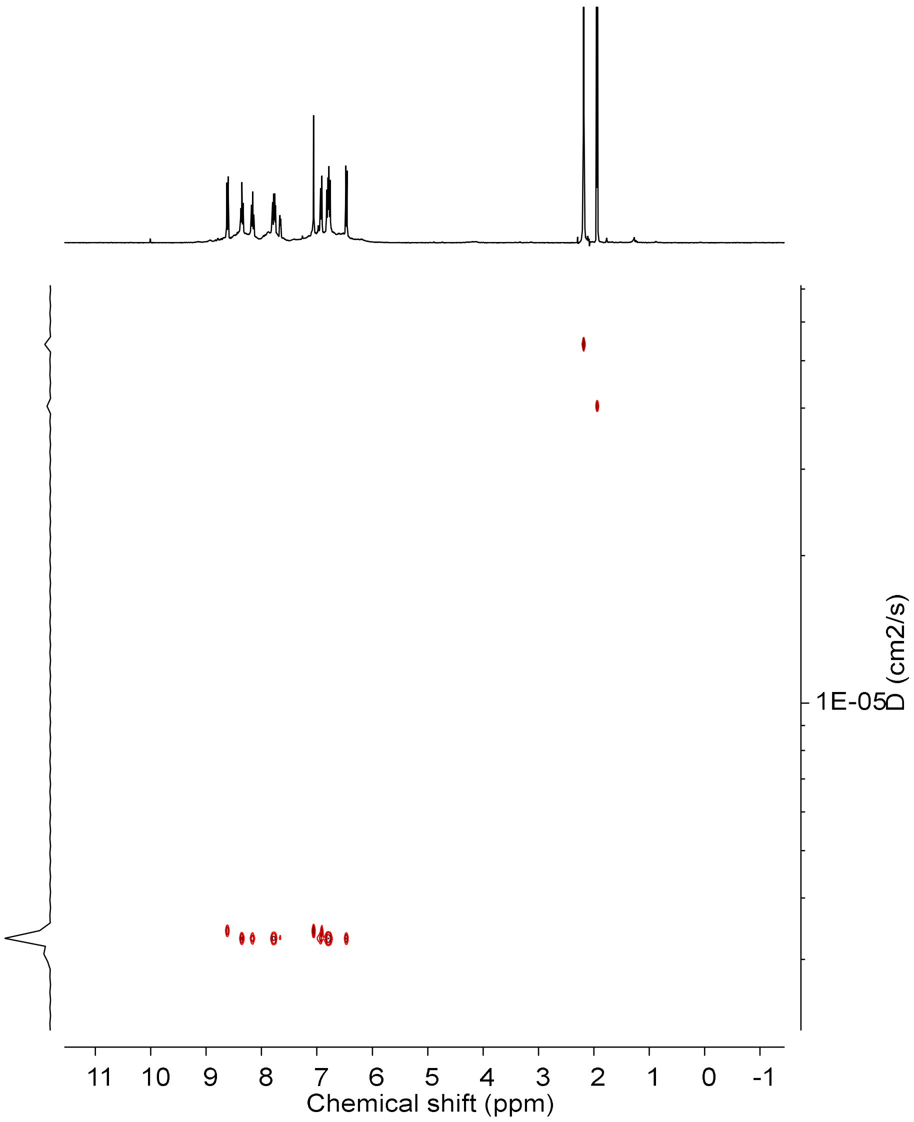

Figures S21. ^{1}H DOSY spectrum (400 MHz, CD_3CN, 298 K) of **1**·$(NTf_2)_{16}$.

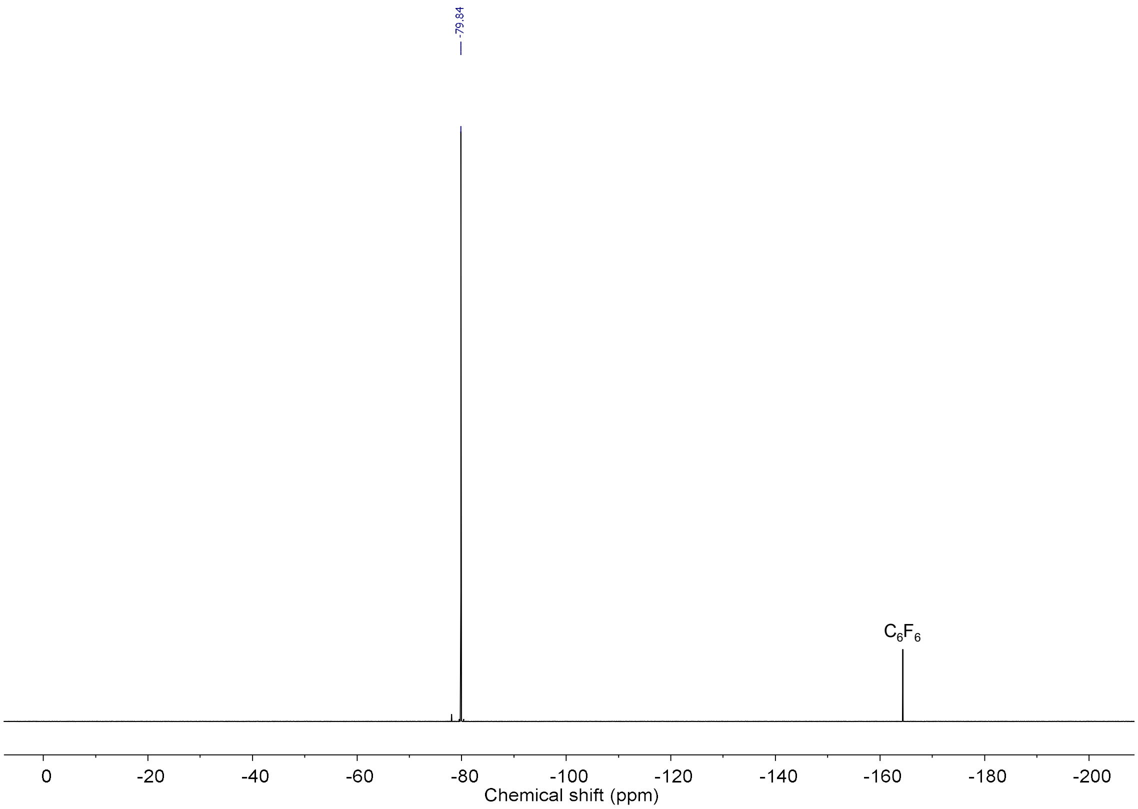

Figure S22. ^{19}F NMR spectrum (376 MHz, CD_3CN, 298 K) of **1**·$(NTf_2)_{16}$.

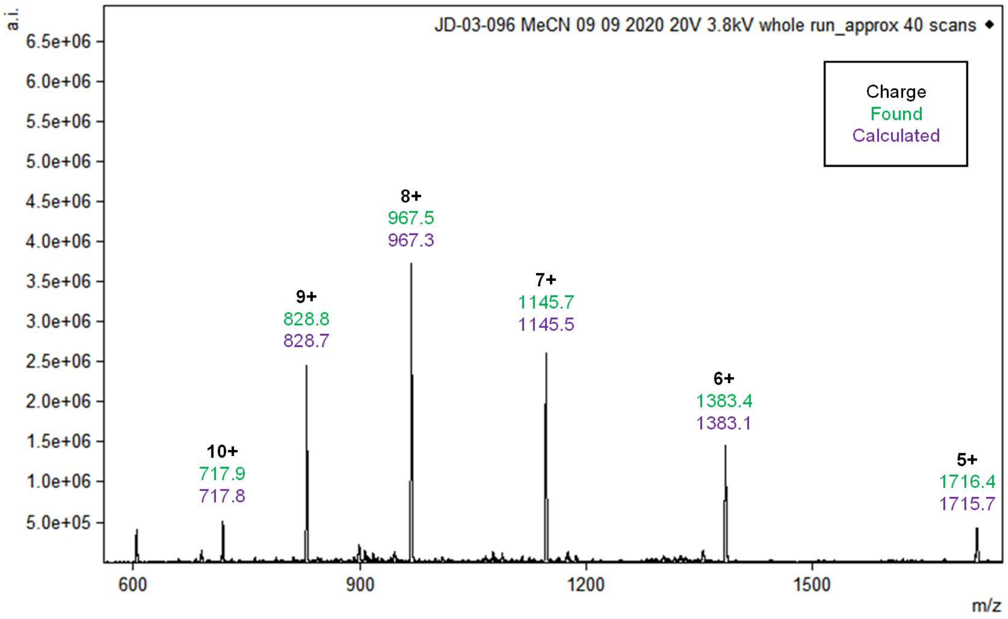

Figure S23. Low resolution ESI-mass spectrum for **1**·$(NTf_2)_{16}$.

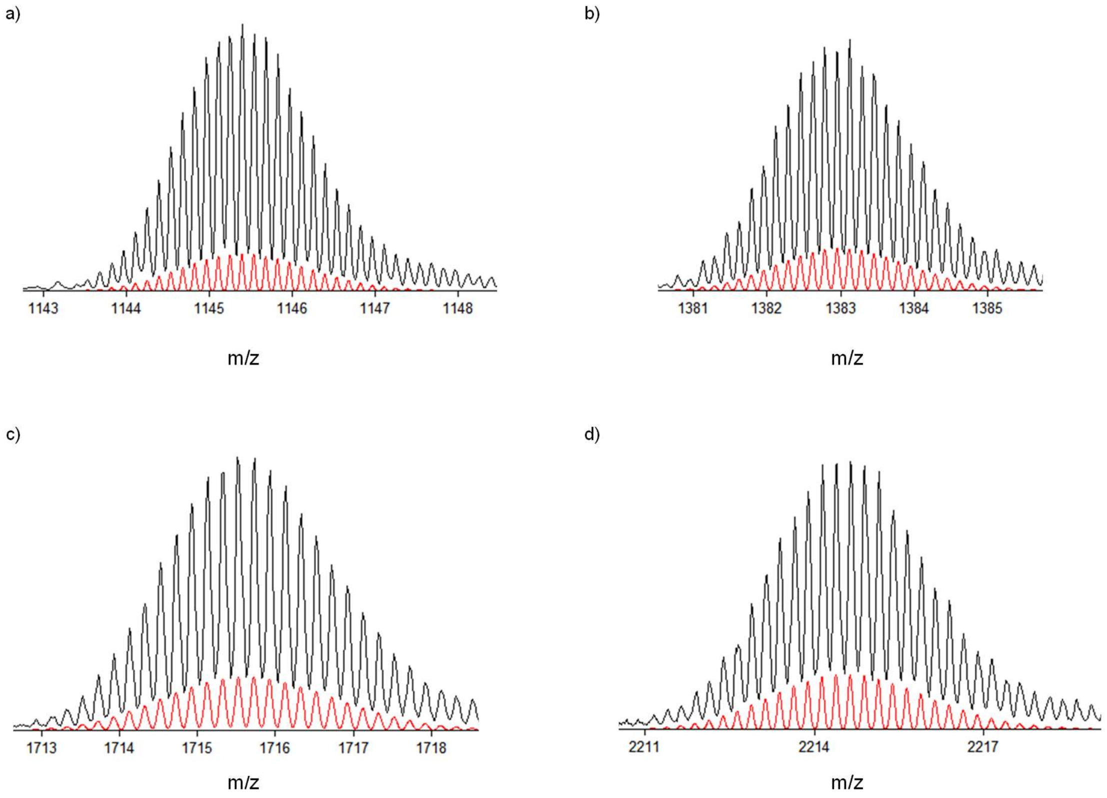

Figure S24. Signals from the high resolution ESI-mass spectrum for **1**·$(NTf_2)_{16}$. Experimental (black) and calculated (red) signals for a) $[\mathbf{1}(NTf_2)_9]^{7+}$ b) $[\mathbf{1}(NTf_2)_{10}]^{6+}$ c) $[\mathbf{1}(NTf_2)_{11}]^{5+}$ d) $[\mathbf{1}(NTf_2)_{12}]^{4+}$.

3.2 Synthesis and characterization of 2

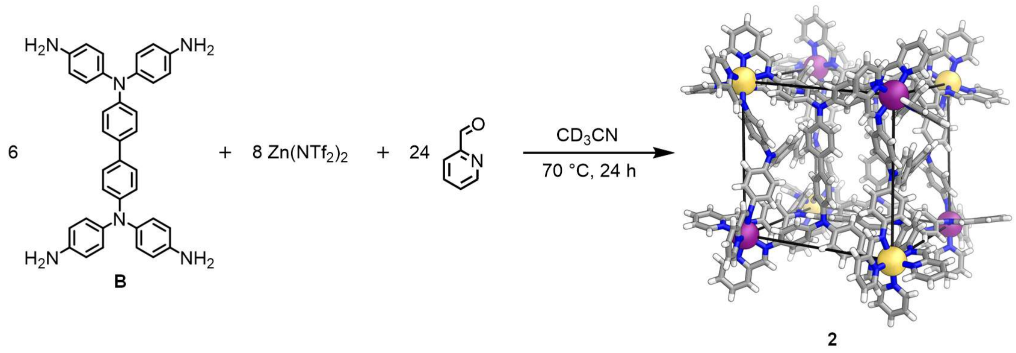

Scheme S2. Subcomponent self-assembly of **2**·$(NTf_2)_{16}$.

To a Schlenk flask, subcomponent **B** (5.0 mg, 9.1 μmol, 1.0 equiv), $Zn(NTf_2)_2$ (8.3 mg, 13.3 μmol, 1.5 equiv) and CD_3CN (0.6 mL) were added. 2-formylpyridine (3.7 μL, 38.9 μmol, 4.3 equiv) was added, the mixture was degassed by conducting four freeze-pump-thaw cycles and the reaction mixture was heated at 70 °C under nitrogen for 24 hours. The reaction mixture was allowed to cool to room temperature, filtered through a glass fibre plug, concentrated to a small volume using a stream of nitrogen, and diethyl ether (ca. 15 mL) was added. The precipitate was collected using centrifugation and washed with diethyl ether (3 × 15 mL). The product **2**·$(NTf_2)_{16}$ was obtained as a very dark red/orange solid (14.5 mg, 1.4 μmol, 92%).

^{1}H NMR (500 MHz, CD_3CN, 298 K) δ 8.70 (s, 24H), 8.37 (app. td, J = 7.8, 1.5 Hz, 24H), 8.19 (d, J = 7.8 Hz, 24H), 7.78 (ddd, J = 7.8, 5.2, 1.1 Hz, 24H), 7.64 (d, J = 5.0 Hz, 24H), 7.42 (d, J = 8.7 Hz, 24H), 6.94 (d, J = 8.8 Hz, 48H), 6.82 (d, J = 8.7 Hz, 24H), 6.74 (d, J = 8.8 Hz, 48H).

^{13}C NMR (126 MHz, CD_3CN, 298 K) δ 163.7, 149.9, 147.7, 147.4, 147.3, 143.8, 143.4, 134.8, 131.8, 131.6, 128.0, 127.2, 125.2, 123.0, 120.8 (q, J = 321 Hz, NTf_2).

^{19}F NMR (376 MHz, CD_3CN, 298 K) δ −79.92.

ESI-LRMS ([**2**$(NTf_2)_{16}$] = $C_{360}H_{264}N_{60}Zn_8(C_2F_6NO_4S_2)_{16}$) *m/z* = 589.5 [**2**$(NTf_2)_4$]$^{12+}$ (calc. 589.5), 668.8 [**2**$(NTf_2)_5$]$^{11+}$ (calc. 668.6), 763.6 [**2**$(NTf_2)_6$]$^{10+}$ (calc. 763.4), 879.6 [**2**$(NTf_2)_7$]$^{9+}$ (calc. 879.4), 1024.6 [**2**$(NTf_2)_8$]$^{8+}$ (calc. 1024.3), 1210.9 [**2**$(NTf_2)_9$]$^{7+}$ (calc. 1210.7), 1459.7 [**2**$(NTf_2)_{10}$]$^{6+}$ (calc. 1459.2).

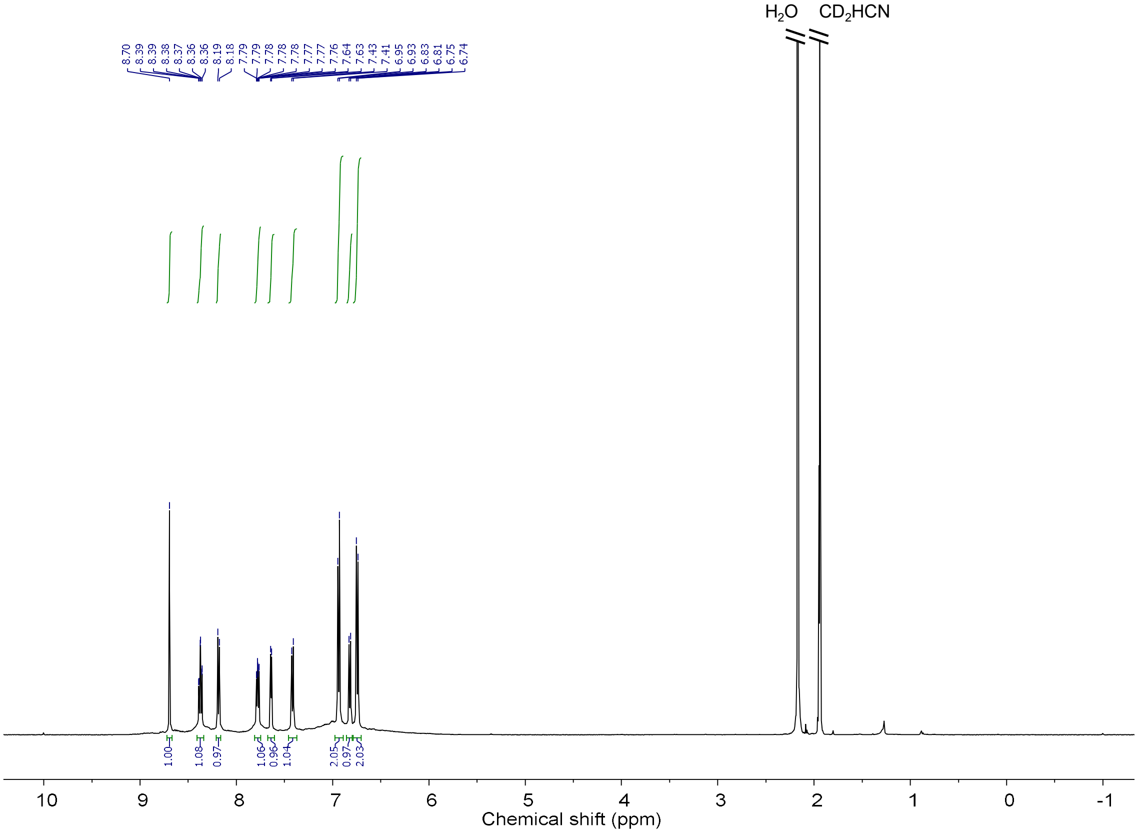

Figure S25. ^{1}H NMR spectrum (500 MHz, CD_3CN, 298 K) of **2**·$(NTf_2)_{16}$.

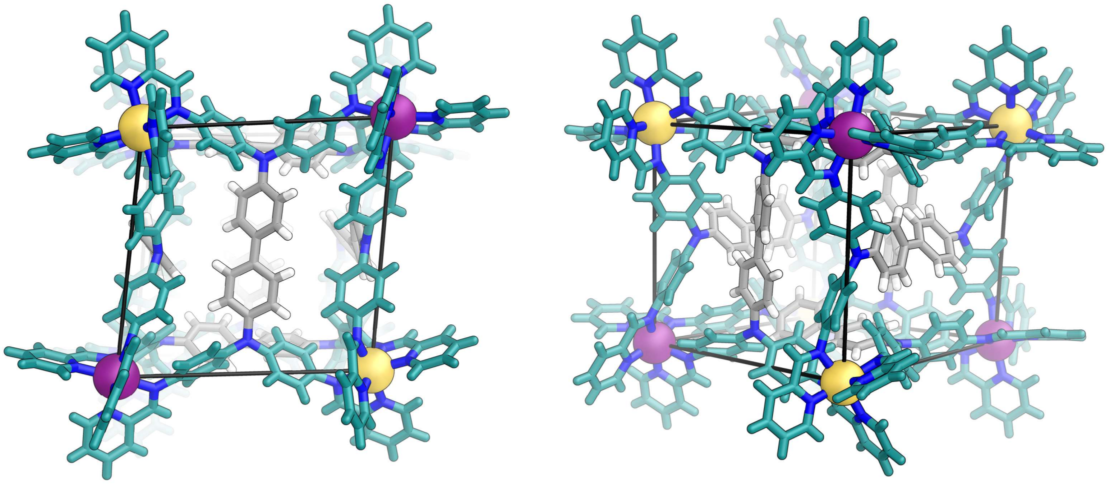

Figure S26. Views of the crystal structure of **2** illustrating the magnetic equivalence of all ligand arms. (This is assuming idealized T_h point symmetry, not all ligand arms are crystallographically equivalent).

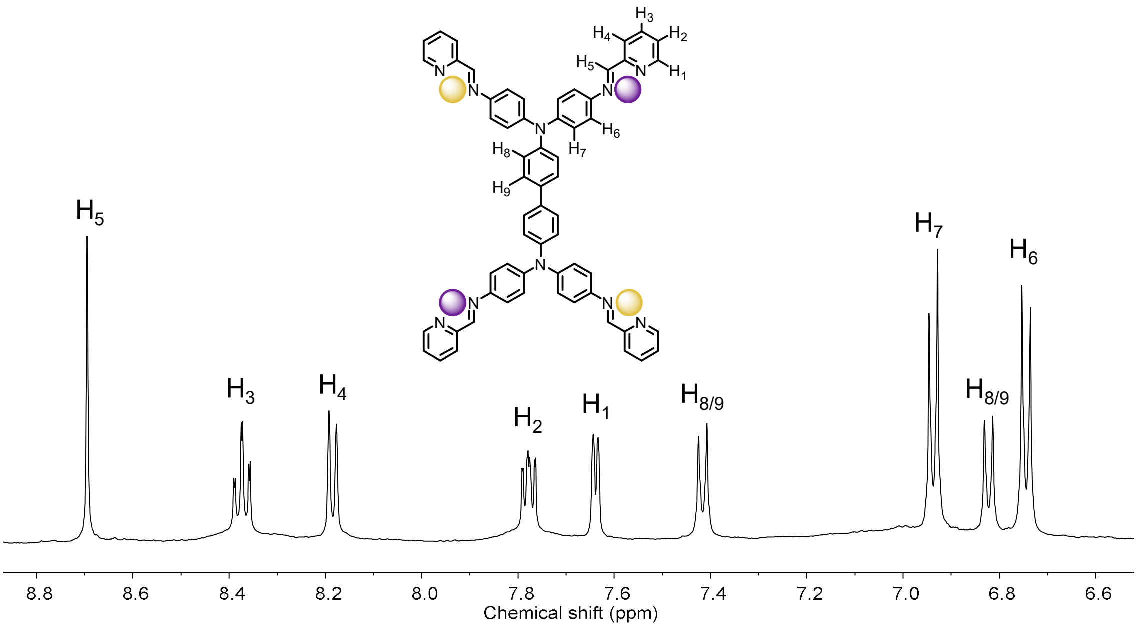

Figure S27. Aromatic region of the ^{1}H NMR spectrum (500 MHz, CD_3CN, 298 K) of **2**·$(NTf_2)_{16}$, with assignment of signals.

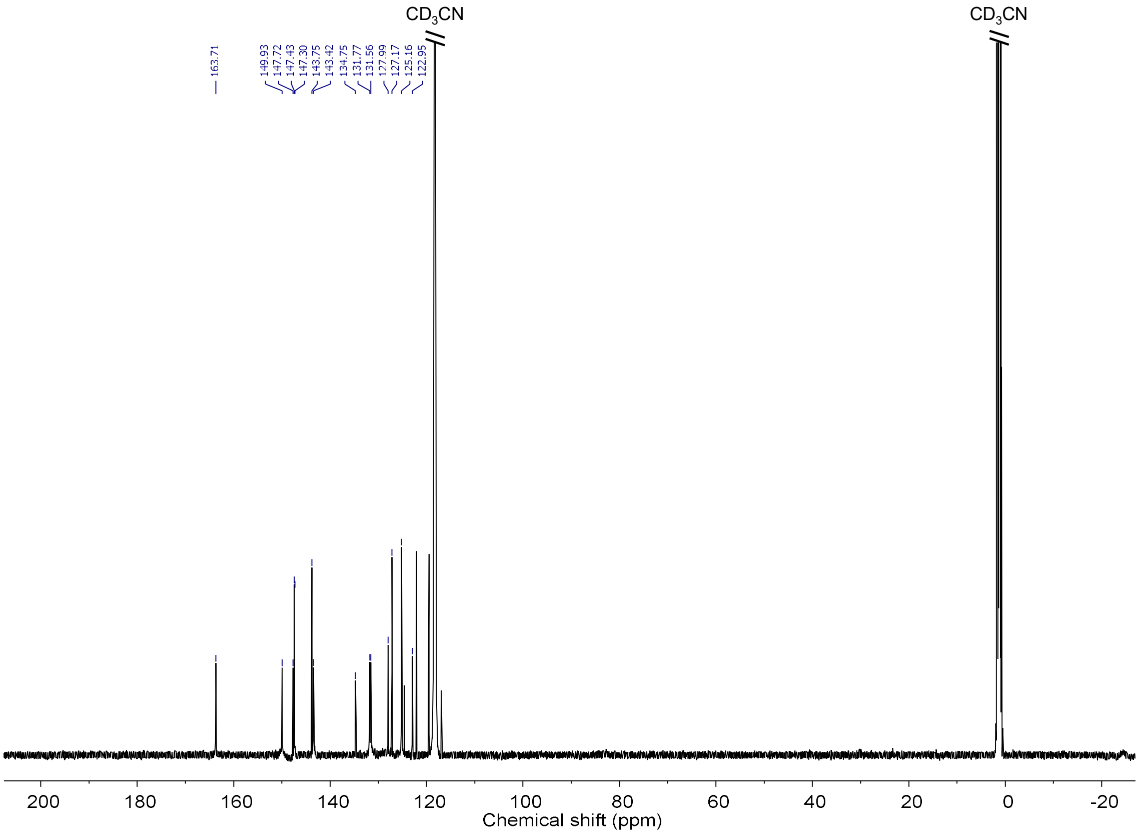

Figure S28. ^{13}C NMR spectrum (126 MHz, CD_3CN, 298 K) of **2**·$(NTf_2)_{16}$.

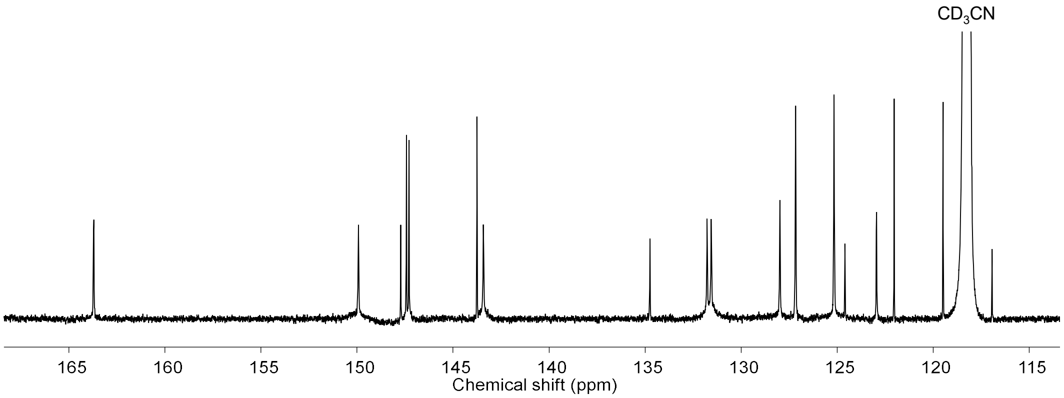

Figure S29. Aromatic region of the ^{13}C NMR spectrum (126 MHz, CD_3CN, 298 K) of **2**·$(NTf_2)_{16}$.

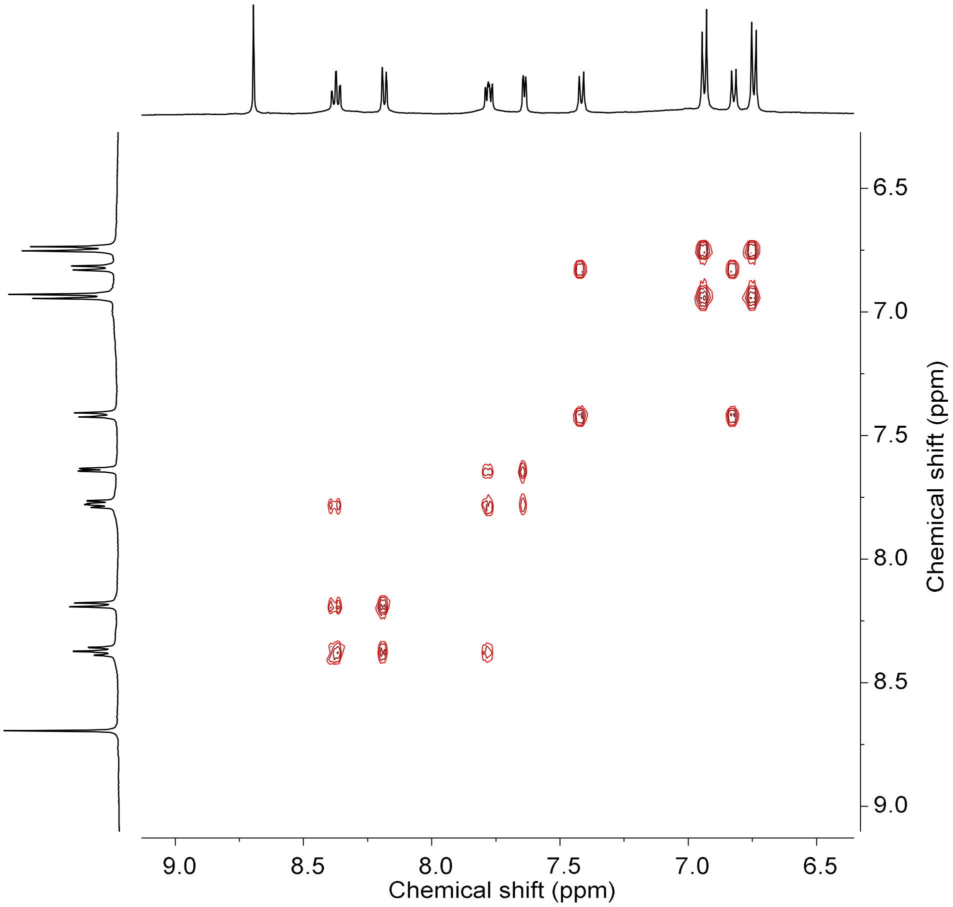

Figure S30. Aromatic region of the ^{1}H-^{1}H DQF-COSY spectrum (500 MHz, CD_3CN, 298 K) of **2**·$(NTf_2)_{16}$.

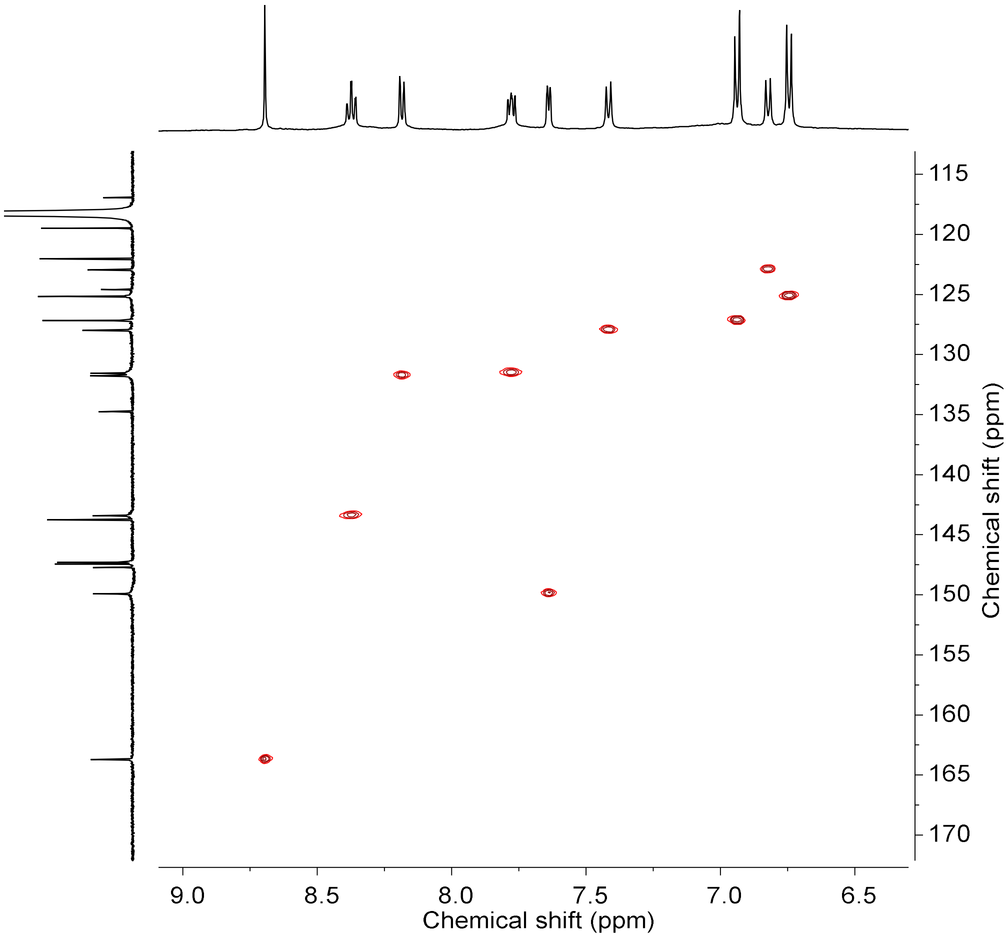

Figure S31. Aromatic region of the ^{1}H-^{13}C edited HSQC spectrum (500 MHz, CD_3CN, 298 K) of **2**·$(NTf_2)_{16}$.

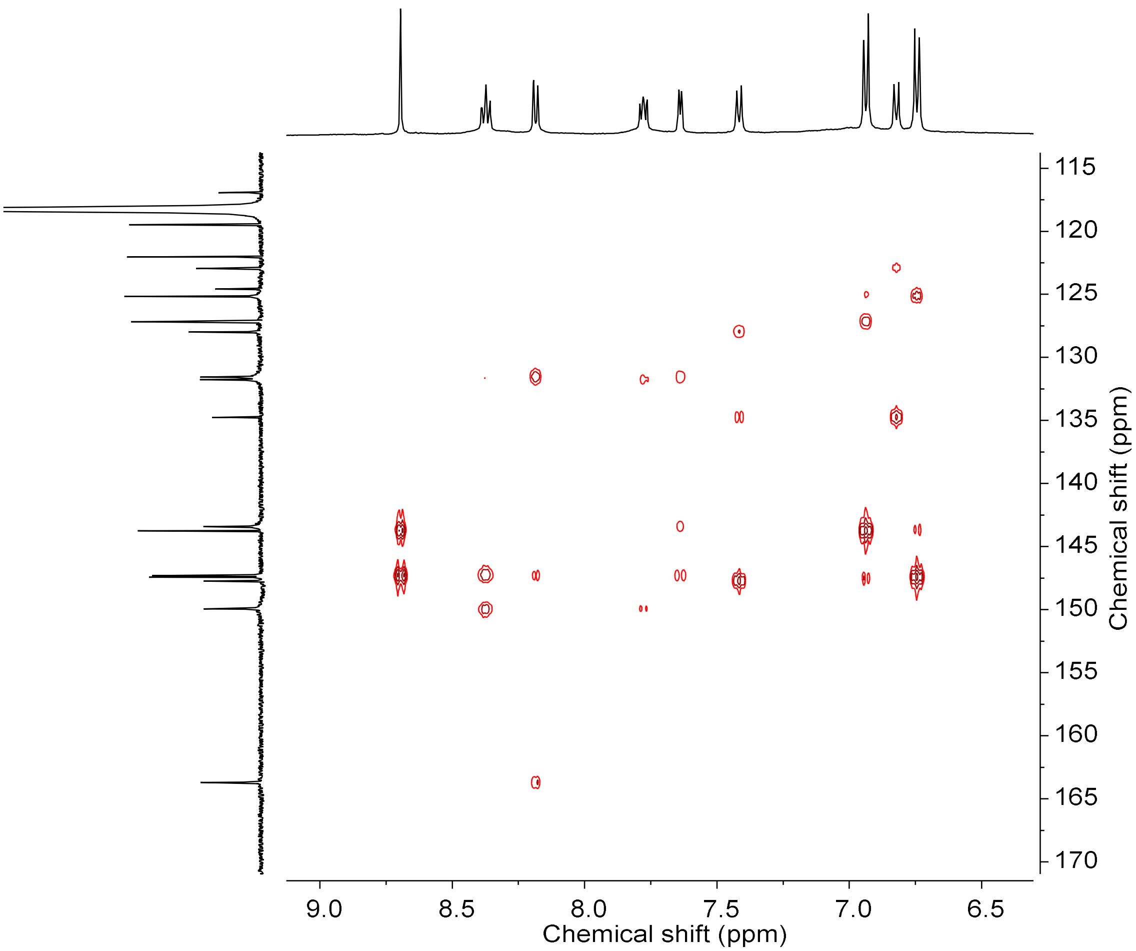

Figure S32. Aromatic region of the ^1H-^{13}C HMBC spectrum (500 MHz, CD_3CN, 298 K) of **2**·$(NTf_2)_{16}$.

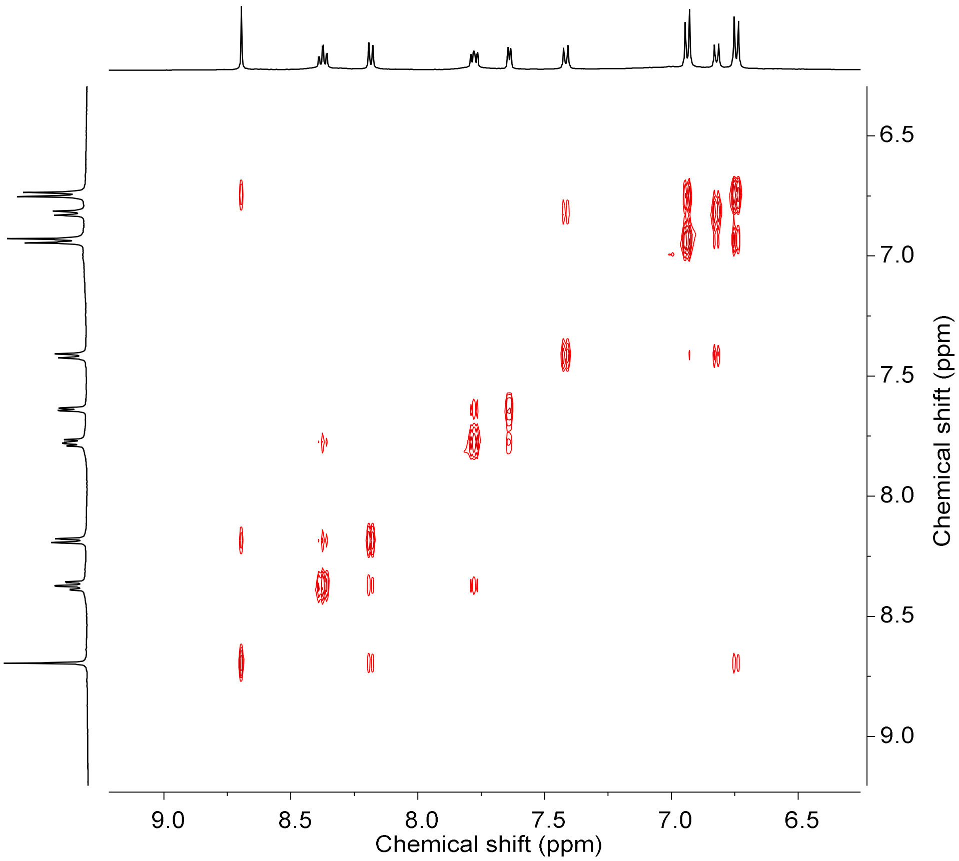

Figure S33. Aromatic region of the ^1H-^1H NOESY spectrum (500 MHz, CD_3CN, 298 K) of **2**·$(NTf_2)_{16}$.

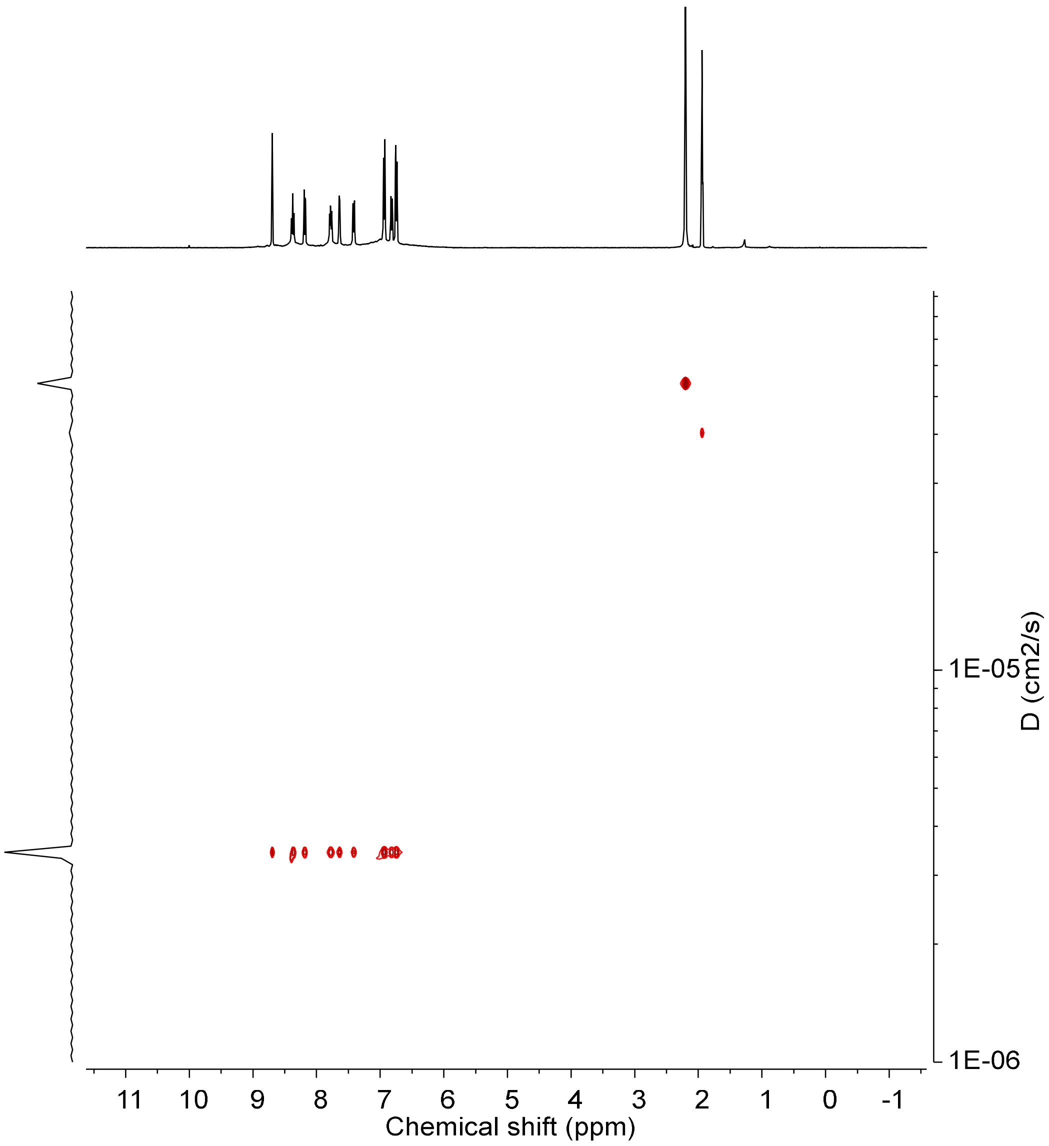

Figure S34. ^{1}H DOSY spectrum (400 MHz, CD_3CN, 298 K) of **2**·$(NTf_2)_{16}$.

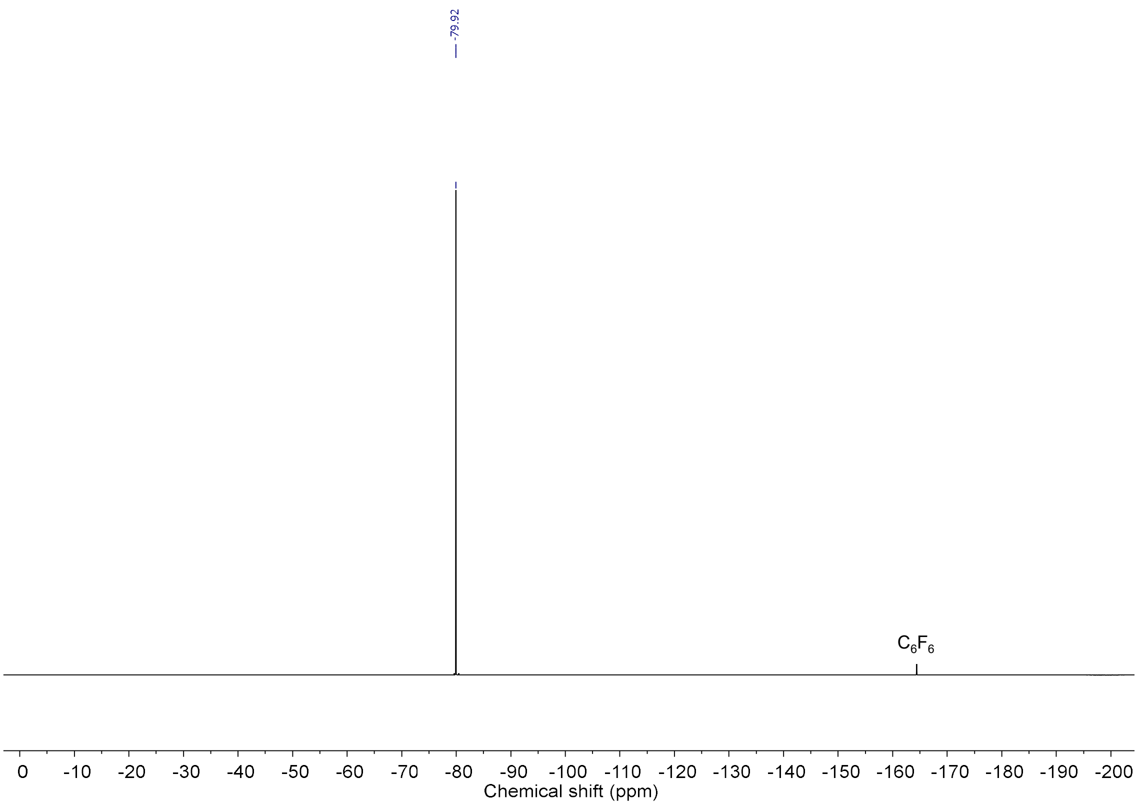

Figure S35. ^{19}F NMR spectrum (376 MHz, CD_3CN, 298 K) of **2**·$(NTf_2)_{16}$.

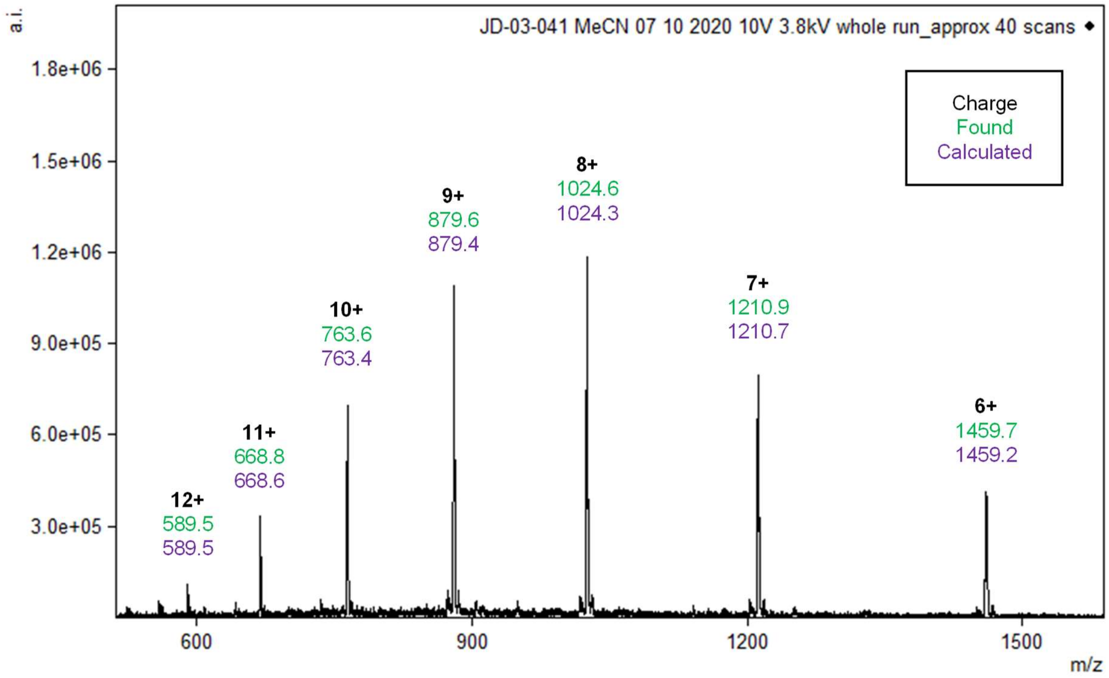

Figure S36. Low resolution ESI-mass spectrum for **2**·$(NTf_2)_{16}$.

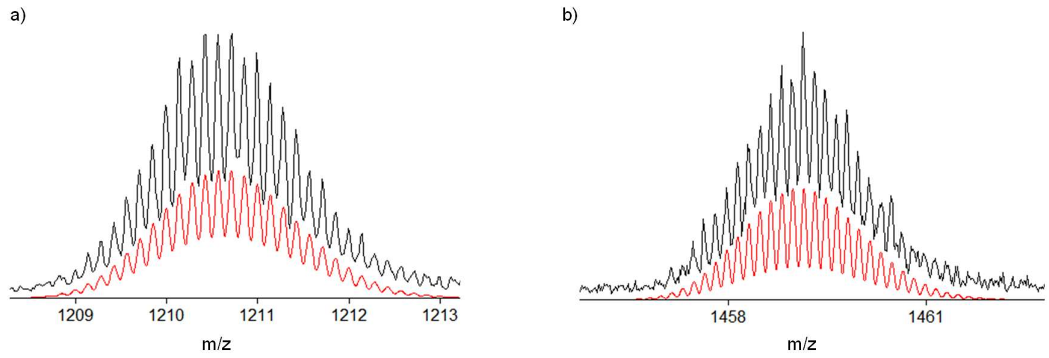

Figure S37. Signals from the high resolution ESI-mass spectrum for **2**·$(NTf_2)_{16}$. Experimental (black) and calculated (red) peaks for a) [**2**$(NTf_2)_9]^{7+}$ b) [**2**$(NTf_2)_{10}]^{6+}$.

3.3 Synthesis and characterization of 3

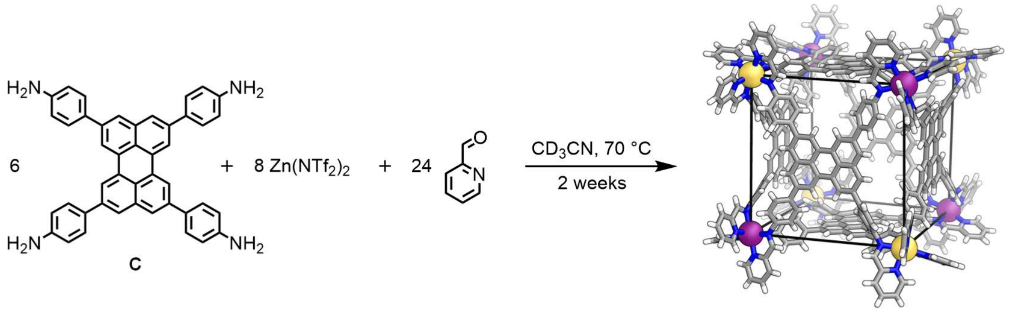

Scheme S3. Subcomponent self-assembly **3**·$(NTf_2)_{16}$.

To a mixture of tetra-aniline **C** (4.8 mg, 7.8 µmol, 1.0 equiv) and $Zn(NTf_2)_2$ (7.9 mg, 12.6 µmol, 1.6 equiv) in CD_3CN (0.6 mL), 2-formylpyridine (3.1 µL, 32.6 µmol, 4.2 equiv) was added. Four freeze-pump-thaw cycles were conducted, and the reaction mixture was heated at 70 °C under nitrogen for 2 weeks. The reaction mixture was allowed to cool to room temperature, filtered through a glass fibre plug, concentrated to a small volume using a stream of nitrogen, and diethyl ether was added. The precipitate was collected using centrifugation and washed with diethyl ether (×4). **3**·$(NTf_2)_{16}$ was obtained as a dark orange/brown solid (14.2 mg, quant.).

^{1}H NMR (500 MHz, CD_3CN, 298 K) δ 8.78 (s, 24H), 8.53 (app. td, *J* = 7.8, 1.5 Hz , 24H), 8.39 (d, *J* = 7.8 Hz, 24H), 8.35 (s, 24H), 8.16 (br d, *J* = 1.1 Hz, 24H), 8.07 (d, *J* = 5.0 Hz, 24H), 7.95 (ddd, *J* = 7.8, 5.1, 1.1 Hz, 24H), 7.69 (d, *J* = 8.5 Hz, 48H), 6.49 (d, *J* = 8.5 Hz, 48H).

^{13}C NMR (126 MHz, CD_3CN, 298 K) δ 165.6, 150.6, 147.9, 147.4, 143.7, 141.8, 139.9, 136.3, 132.0, 131.8, 131.7, 129.6, 127.9, 127.8, 122.7, 121.6, 120.9 (q, *J* = 321 Hz, NTf_2).

^{19}F NMR (376 MHz, CD_3CN, 298 K) δ −79.87.

ESI-LRMS ([**3**]$(NTf_2)_{16}$] = $C_{408}H_{264}N_{48}Zn_8(C_2F_6NO_4S_2)_{16}$) *m/z* = 442.9 [**3**$(NTf_2)$]$^{15+}$ (calc. 442.8), 494.5 [**3**$(NTf_2)_2$]$^{14+}$ (calc. 494.4), 554.1 [**3**$(NTf_2)_3$]$^{13+}$ (calc. 554.0), 623.6 [**3**$(NTf_2)_4$]$^{12+}$ (calc. 623.5), 705.8 [**3**$(NTf_2)_5$]$^{11+}$ (calc. 705.7), 804.4 [**3**$(NTf_2)_6$]$^{10+}$ (calc. 804.3), 924.9 [**3**$(NTf_2)_7$]$^{9+}$ (calc. 924.8), 1075.6 [**3**$(NTf_2)_8$]$^{8+}$ (calc. 1075.4), 1269.2 [**3**$(NTf_2)_9$]$^{7+}$ (calc. 1269.0), 1527.5 [**3**$(NTf_2)_{10}$]$^{6+}$ (calc. 1527.2).

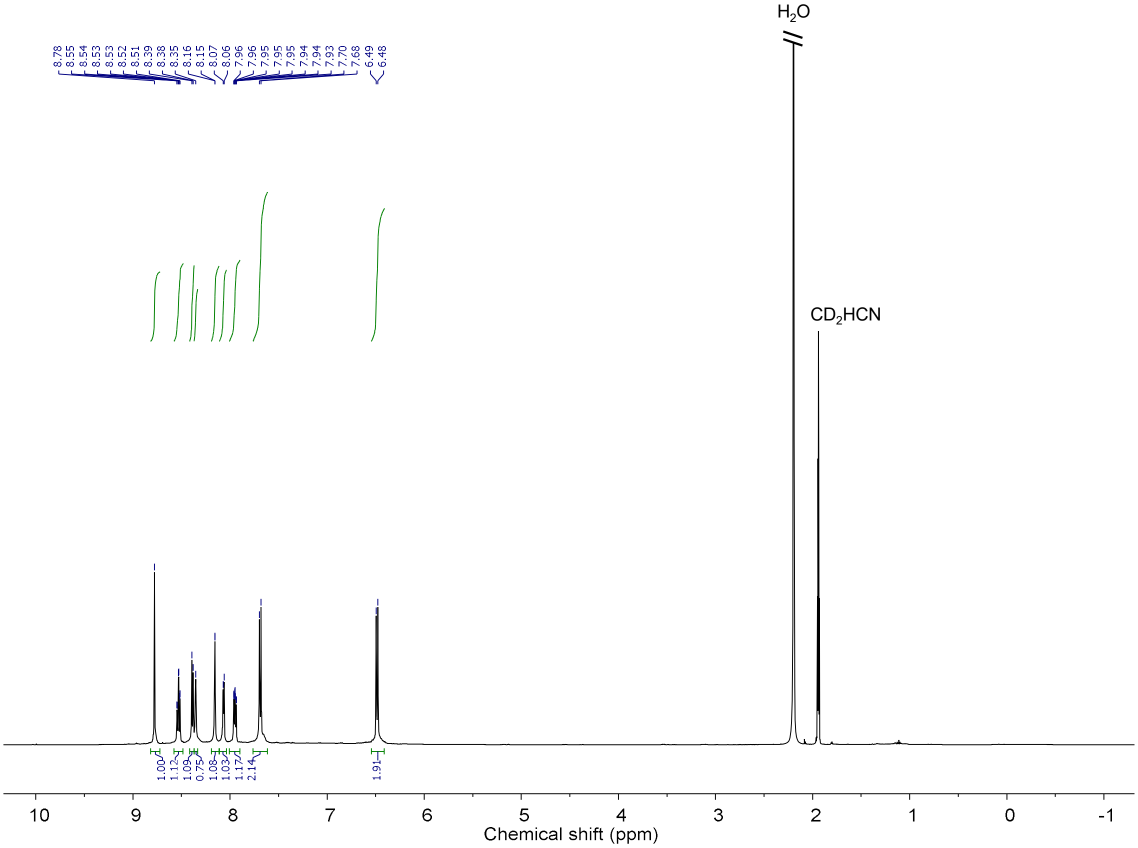

Figure S38. ^1H NMR spectrum (500 MHz, CD_3CN, 298 K) of **3**·$(NTf_2)_{16}$.

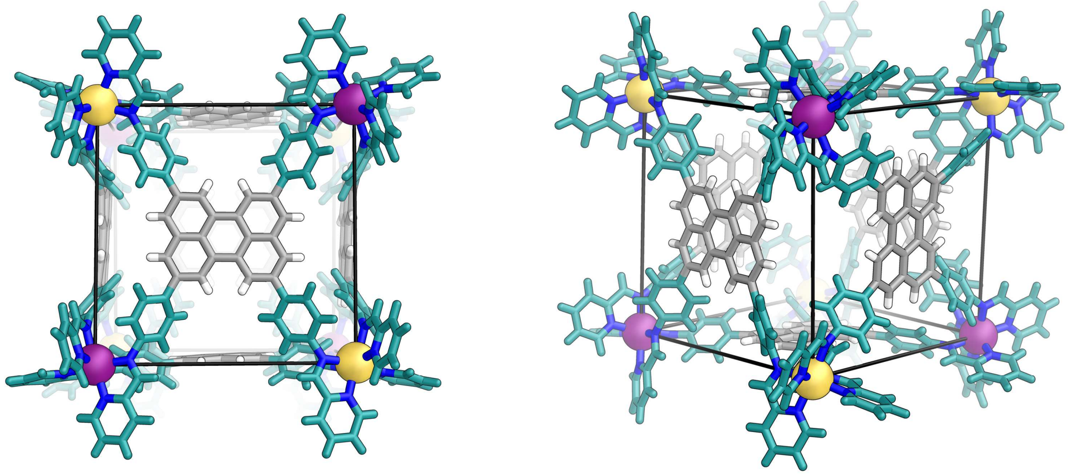

Figure S39. Views of the crystal structure of **3** illustrating the magnetic equivalence of all ligand arms. (This is assuming idealized T_h point symmetry, not all ligand arms are crystallographically equivalent).

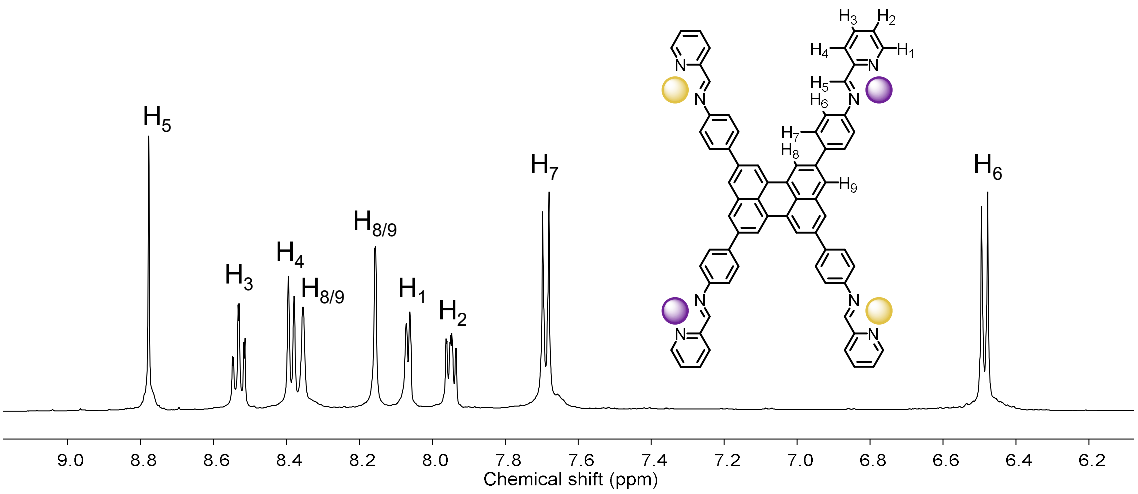

Figure S40. Aromatic region of the ^{1}H NMR spectrum (500 MHz, CD_3CN, 298 K) of **3**·$(NTf_2)_{16}$, with assignment of signals.

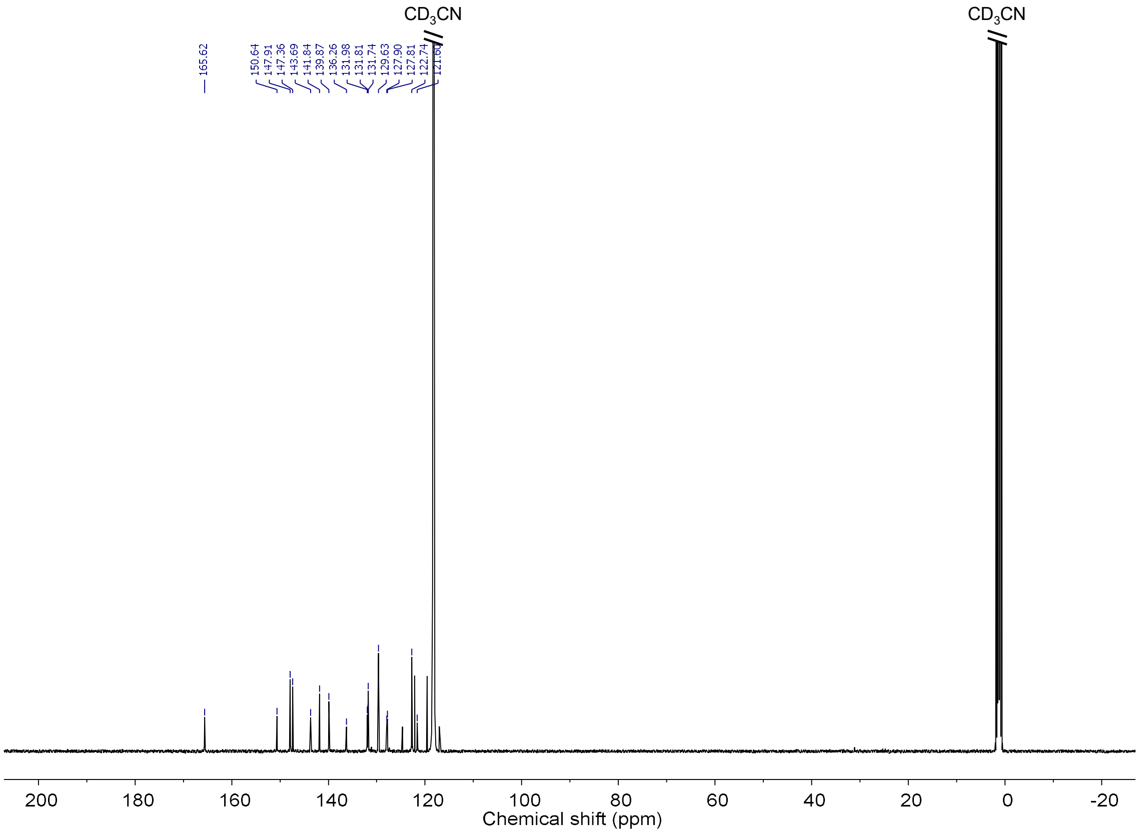

Figure S41. ^{13}C NMR spectrum (126 MHz, CD_3CN, 298 K) of **3**·$(NTf_2)_{16}$.

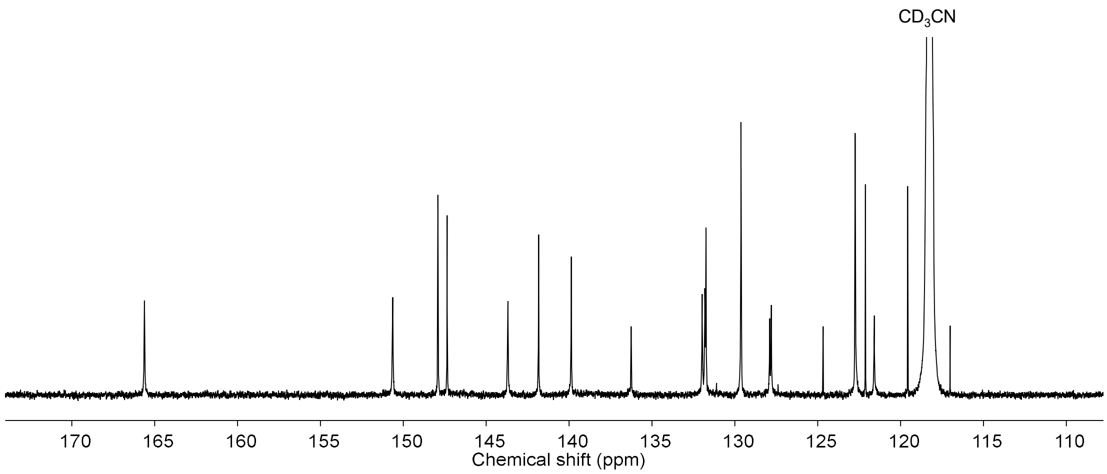

Figure S42. Aromatic region of the ^{13}C NMR spectrum (126 MHz, CD_3CN, 298 K) of **3**·$(NTf_2)_{16}$.

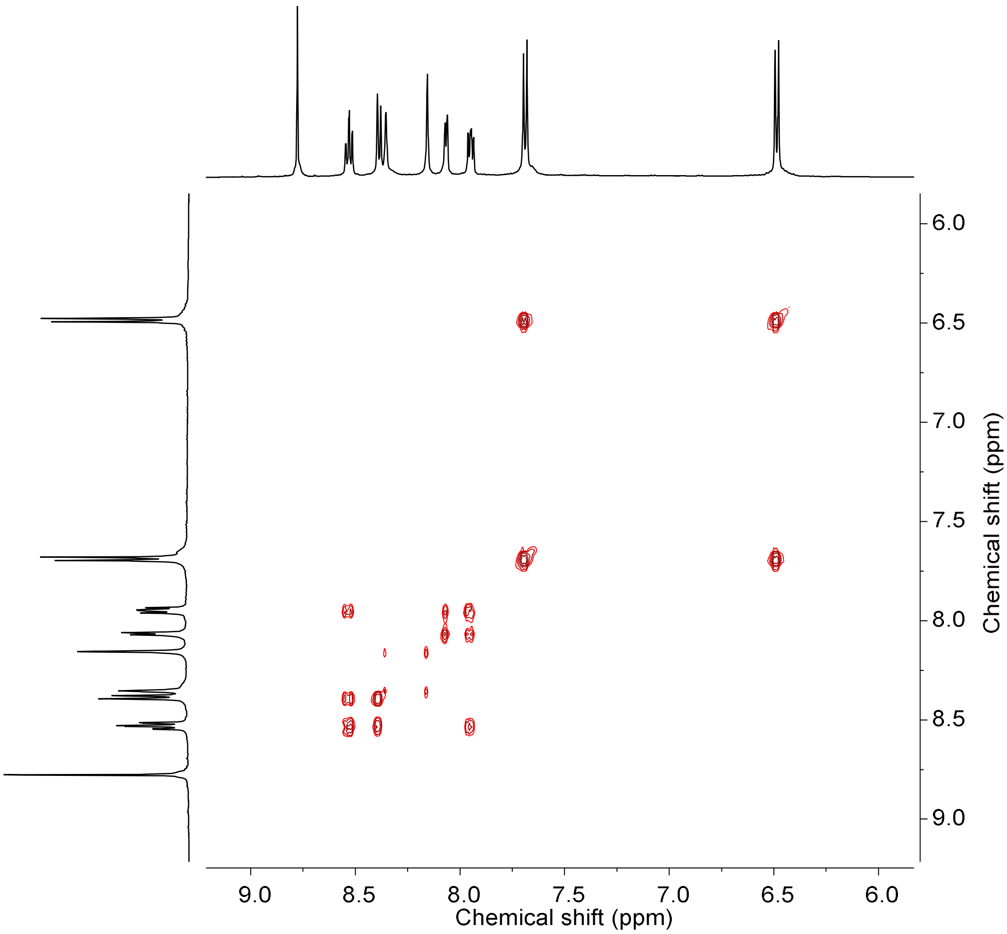

Figure S43. Aromatic region of the ^{1}H-^{1}H DQF-COSY spectrum (500 MHz, CD_3CN, 298 K) of **3**·$(NTf_2)_{16}$.

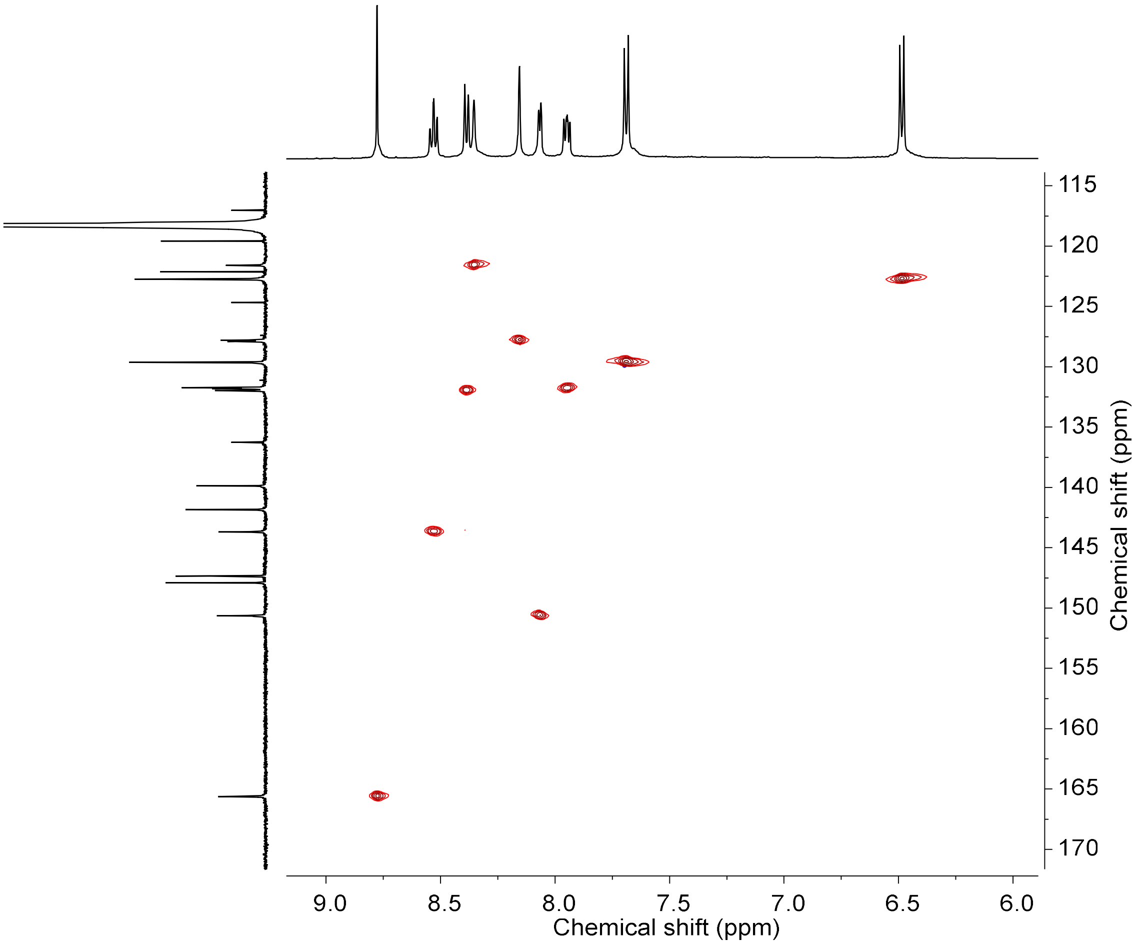

Figure S44. Aromatic region of the ^{1}H-^{13}C edited HSQC spectrum (500 MHz, CD_3CN, 298 K) of **3**·$(NTf_2)_{16}$.

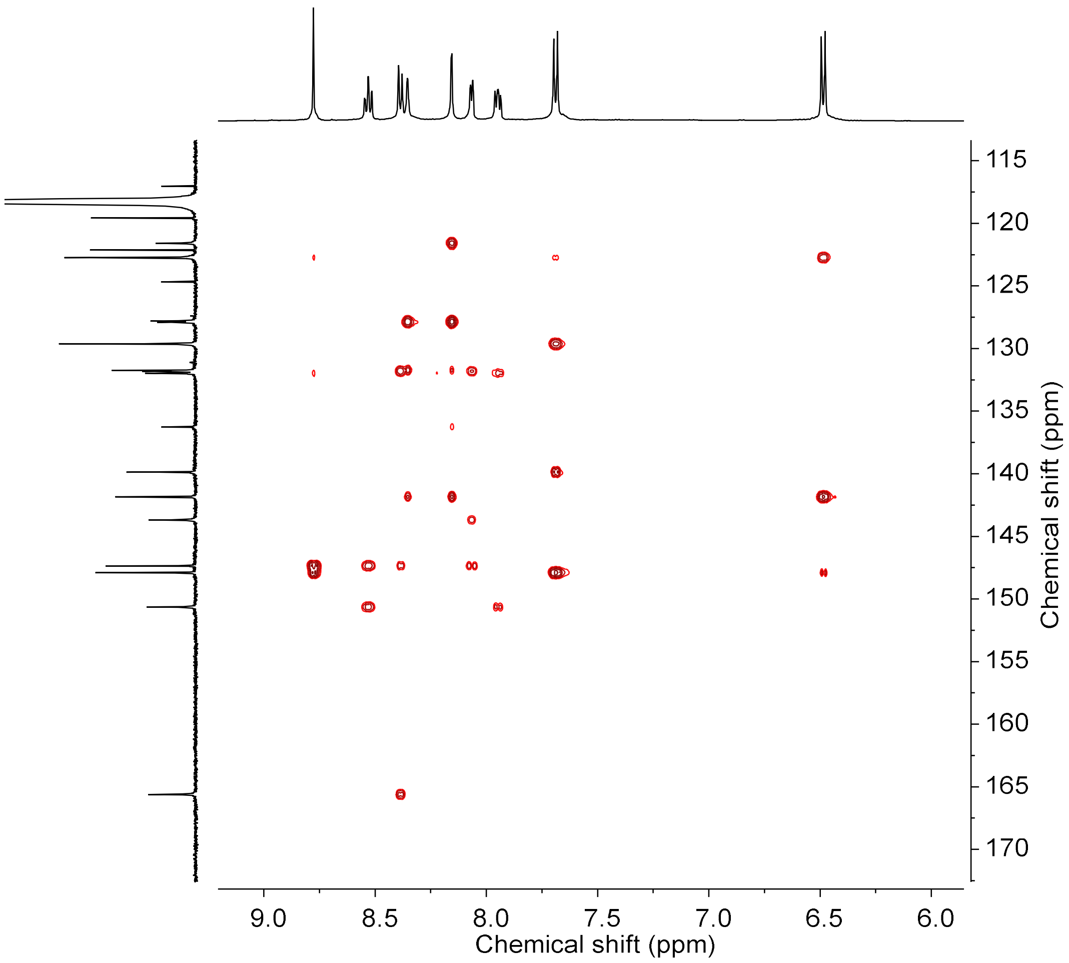

Figure S45. Aromatic region of the ^{1}H-^{13}C HMBC spectrum (500 MHz, CD_3CN, 298K) of **3**·$(NTf_2)_{16}$.

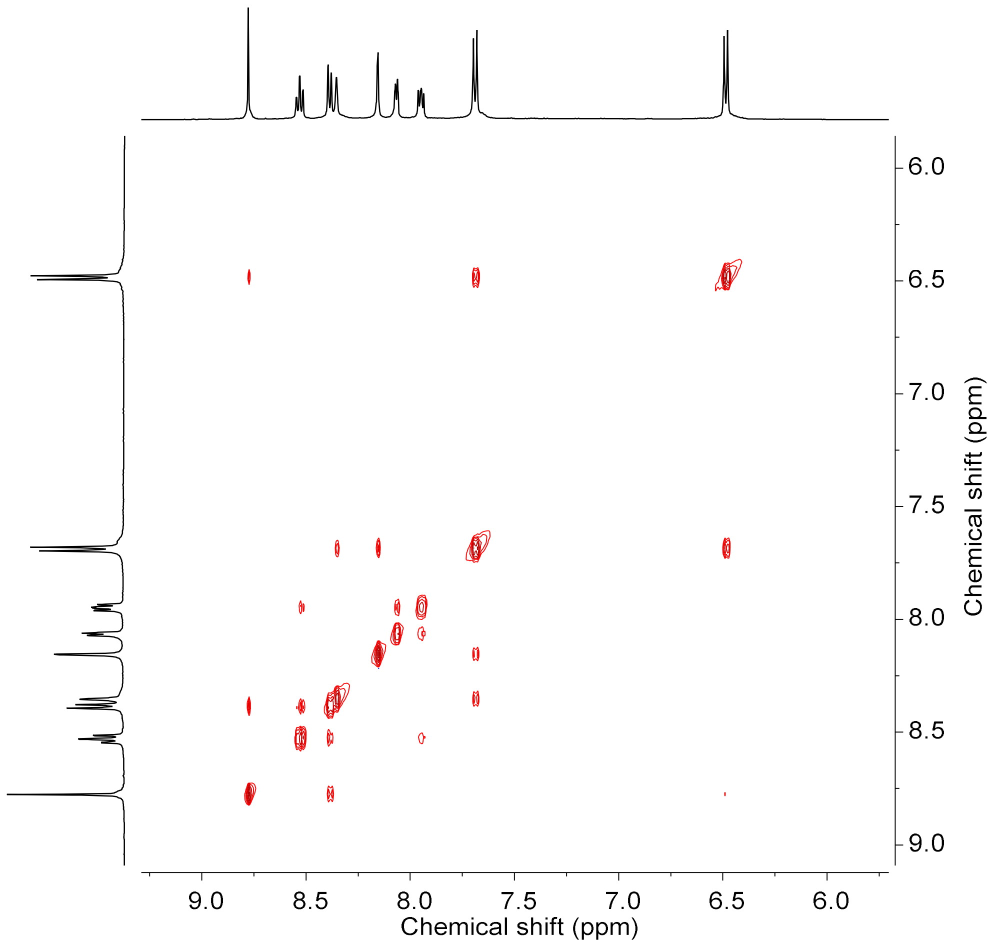

Figure S46. Aromatic region of the ^1H-^1H NOESY spectrum (500 MHz, CD_3CN, 298 K) of **3**·$(NTf_2)_{16}$.

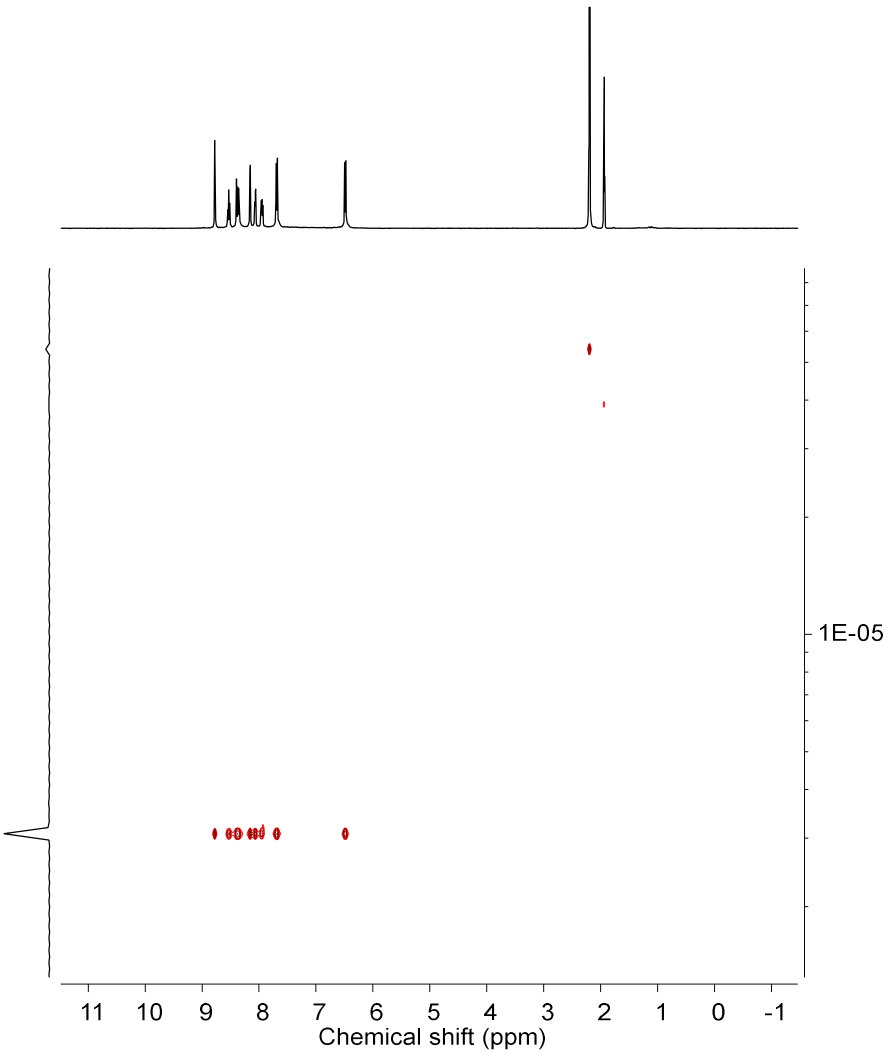

Figures S47. ^{1}H DOSY spectrum (400 MHz, CD_3CN, 298 K) of **3**·$(NTf_2)_{16}$.

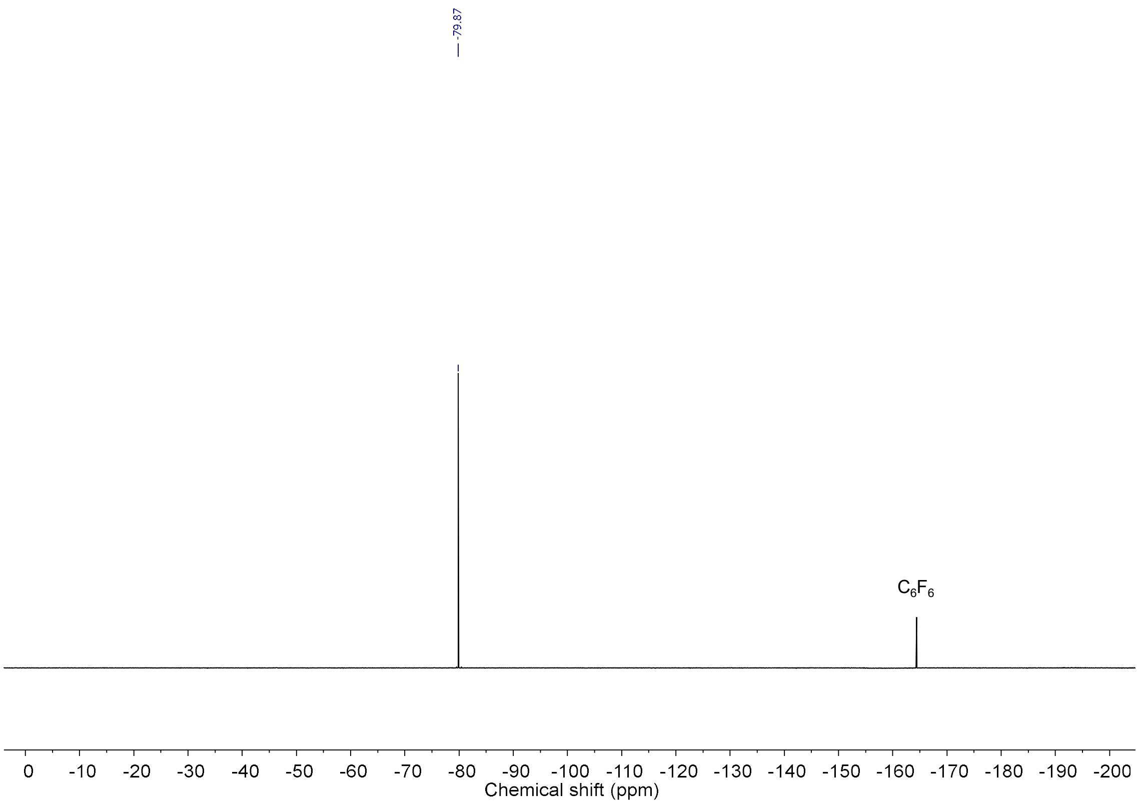

Figure S48. ^{19}F NMR spectrum (376 MHz, CD_3CN, 298 K) of **3**·$(NTf_2)_{16}$.

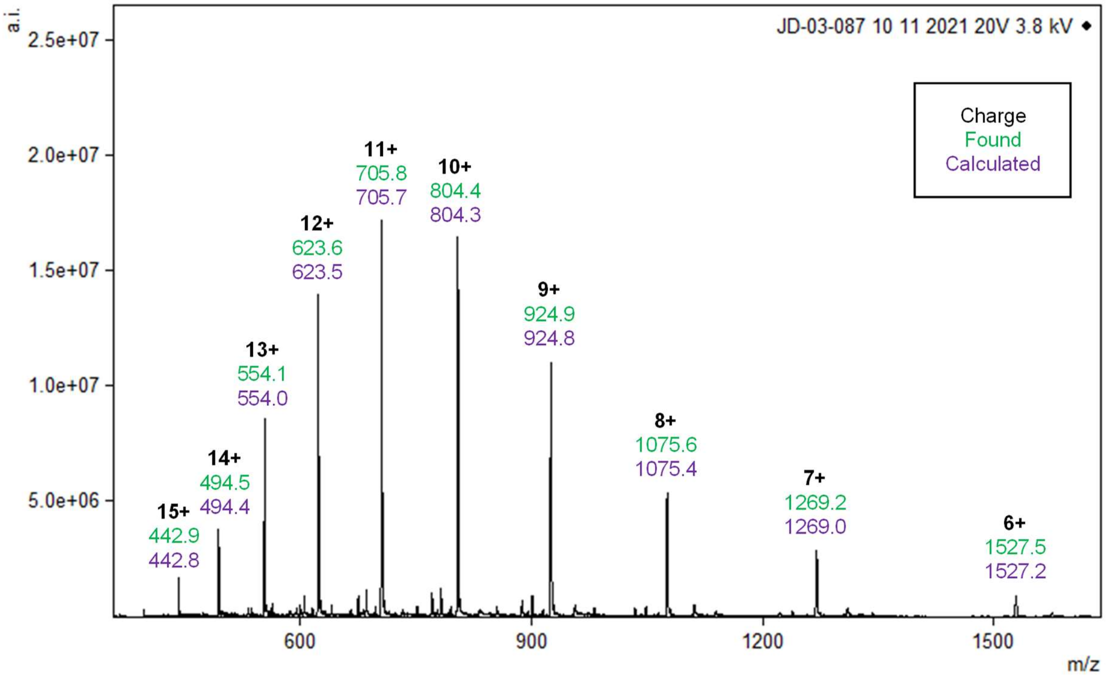

Figure S49. Low resolution ESI-mass spectrum for **3**·$(NTf_2)_{16}$.

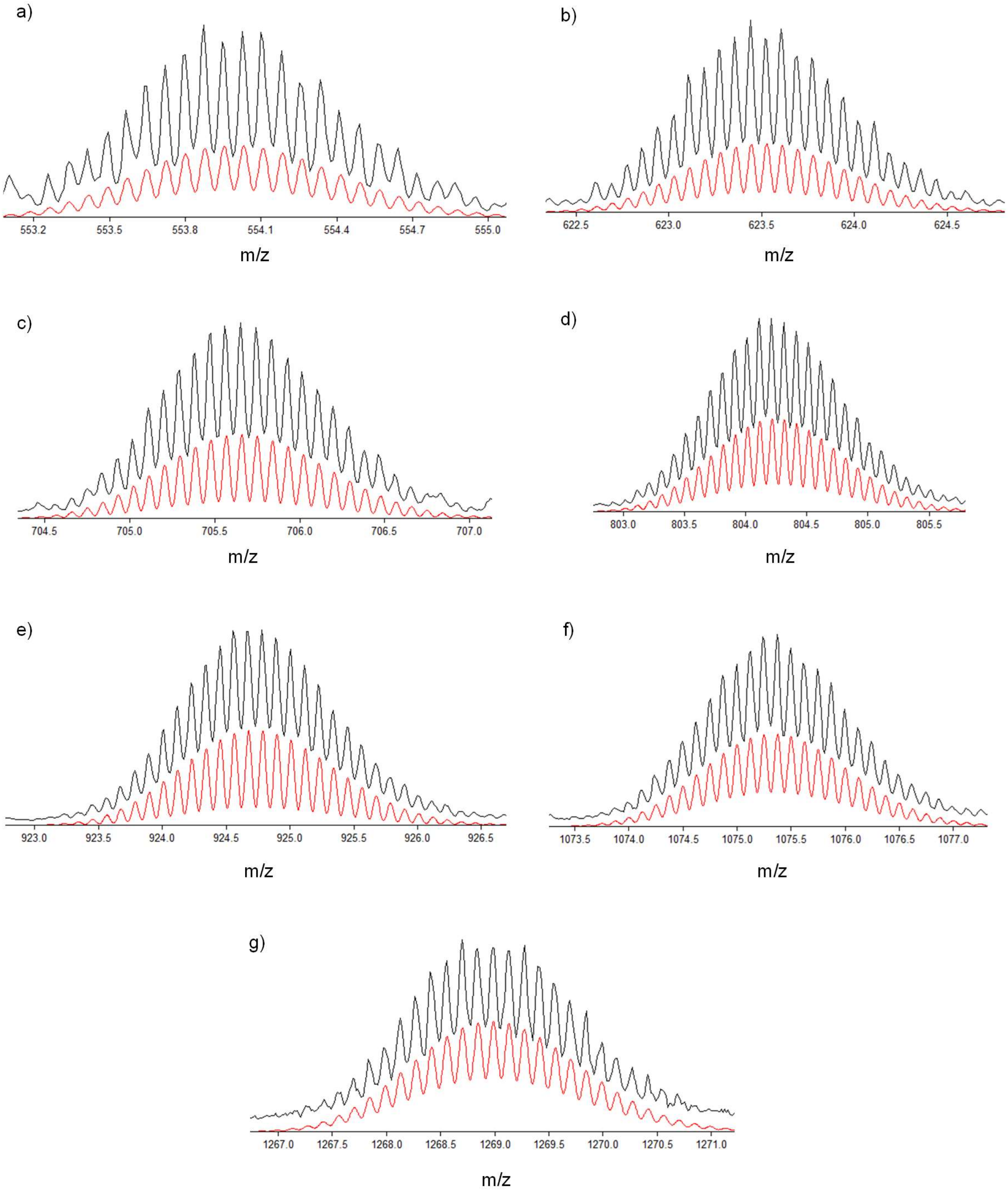

Figure S50. Signals from the high resolution ESI-mass spectrum for **3**·$(NTf_2)_{16}$. Experimental (black) and calculated (red) peaks for a) $[\mathbf{3}(NTf_2)_3]^{13+}$ b) $[\mathbf{3}(NTf_2)_4]^{12+}$ c)$[\mathbf{3}(NTf_2)_5]^{11+}$ d) $[\mathbf{3}(NTf_2)_6]^{10+}$ e) $[\mathbf{3}(NTf_2)_7]^{9+}$ f) $[\mathbf{3}(NTf_2)_8]^{8+}$ g) $[\mathbf{3}(NTf_2)_9]^{7+}$.

3.4 Synthesis and characterization of 5

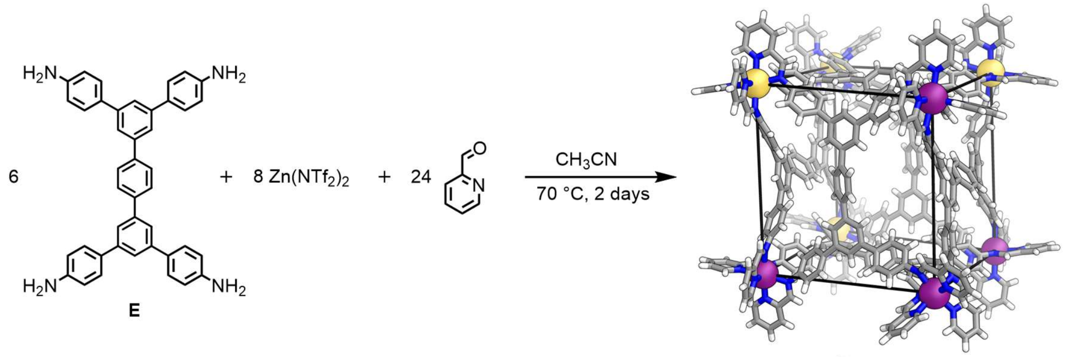

5

Scheme S4. Subcomponent self-assembly of **5**·$(NTf_2)_{16}$.

To a reaction vial, subcomponent **E** (10.0 mg, 16.8 μmol, 1.0 equiv), $Zn(NTf_2)_2$ (16.7 mg, 26.7 μmol, 1.6 equiv) and distilled acetonitrile (2.5 mL) were added. 2-formylpyridine (6.8 μL, 71.5 μmol, 4.3 equiv) was added and the reaction mixture was heated at 70 °C for 44 hours. The reaction mixture was allowed to cool to room temperature, concentrated to a small volume using a stream of nitrogen, and diethyl ether (ca. 15 mL) was added. The precipitate was collected using centrifugation and washed with diethyl ether (4 × 15 mL). The product **5**·$(NTf_2)_{16}$ was obtained as a yellow solid (25.1 mg, 2.3 μmol, 82%).

^{1}H NMR (500 MHz, CD_3CN, 298 K) δ 8.98 (s, 6H), 8.92 (s, 6H), 8.59 (s, 6H), 8.54 (td, *J* = 7.7, 1.5 Hz, 6H), 8.52 (s, 6H), 8.46 (td, *J* = 7.8, 1.5 Hz, 6H), 8.42 (td, *J* = 7.7, 1.5 Hz, 6H), 8.39–8.30 (m, 24H), 8.17 (d, *J* = 5.0 Hz, 6H), 8.06 (d, *J* = 7.7 Hz, 6H), 7.98 (ddd, *J* = 7.9, 5.1, 1.0 Hz, 6H), 7.88–7.59 (m, 132H), 7.56 (d, *J* = 8.6 Hz, 12H), 7.35 (d, *J* = 8.7 Hz, 12H), 7.14 (d, *J* = 8.4 Hz, 12H), 6.83 (d, *J* = 8.6 Hz, 12H), 5.94 (d, *J* = 8.4 Hz, 12H).

^{13}C NMR (126 MHz, CD_3CN, 298 K) δ 165.8, 164.8, 164.7, 164.6, 150.7, 150.3, 150.2, 149.5, 148.3, 147.4, 147.3, 147.3, 147.2, 147.2, 147.1, 147.1, 143.8, 143.7, 143.5, 143.4, 143.3, 143.0, 142.9, 142.7, 142.6, 142.6, 141.9, 141.7, 140.9, 140.7, 140.7, 132.3, 132.0, 131.9, 131.7, 131.6, 130.2, 130.0, 129.9, 129.2, 129.1, 128.8, 128.0, 127.9, 127.9, 126.2, 125.3, 124.4, 124.2, 124.1, 123.6, 122.0, 120.8 (q, *J* = 321 Hz, NTf_2). (Some of the expected peaks could not be located, likely due to signal overlap and potentially also reduced intensity, from the limit in sample concentration and the fourfold desymmetrization of the ligand in the cage structure).

^{19}F NMR (376 MHz, CD_3CN, 298 K) δ −79.97.

ESI-LRMS ([**5**$(NTf_2)_{16}$] = $C_{396}H_{276}N_{48}Zn_8(C_2F_6NO_4S_2)_{16}$) *m/z* = 485.1 [**5**$(NTf_2)_2$]$^{14+}$ (calc. 485.0), 544.0 [**5**$(NTf_2)_3$]$^{13+}$ (calc. 543.9), 612.6 [**5**$(NTf_2)_4$]$^{12+}$ (calc. 612.5), 693.8 [**5**$(NTf_2)_5$]$^{11+}$ (calc. 693.7), 791.2 [**5**$(NTf_2)_6$]$^{10+}$ (calc. 791.1), 910.3 [**5**$(NTf_2)_7$]$^{9+}$ (calc. 910.1), 1059.1 [**5**$(NTf_2)_8$]$^{8+}$ (calc. 1058.9), 1250.4 [**5**$(NTf_2)_9$]$^{7+}$ (calc. 1250.2), 1505.6 [**5**$(NTf_2)_{10}$]$^{6+}$ (calc. 1505.2).

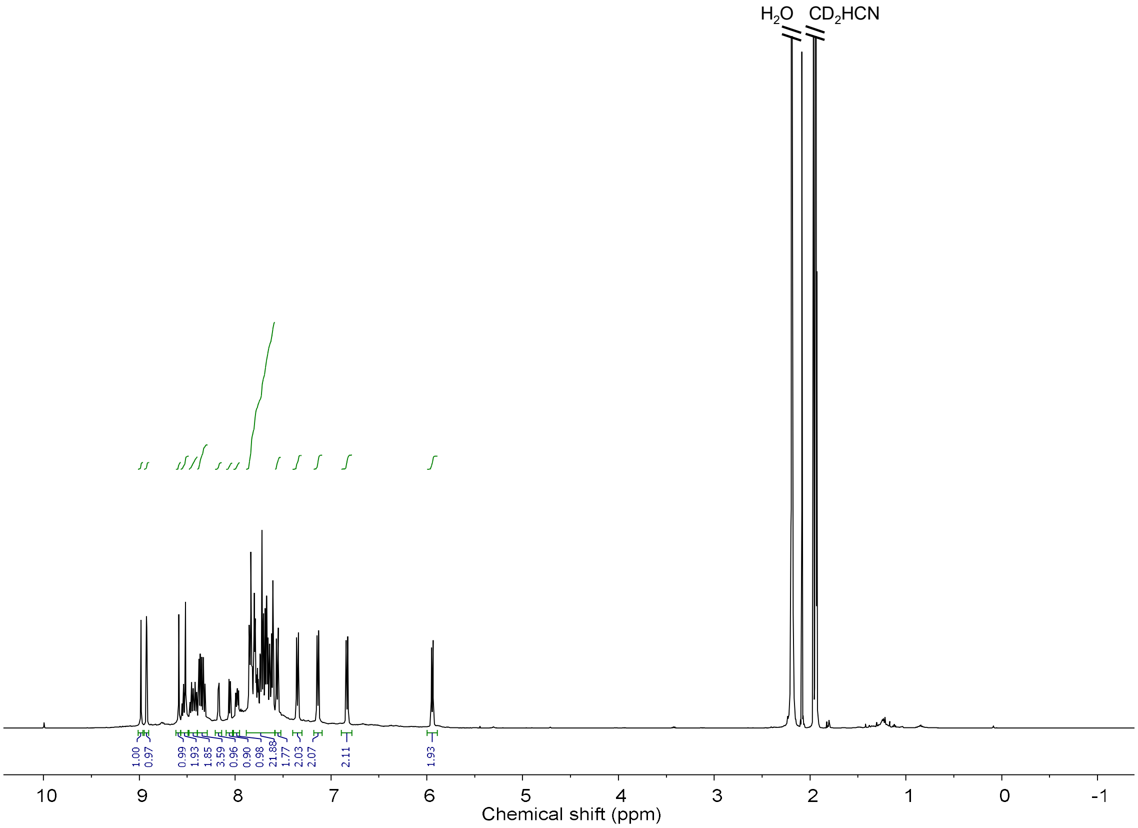

Figure S51. ^{1}H NMR spectrum (500 MHz, CD_3CN, 298 K) of **5**·$(NTf_2)_{16}$.

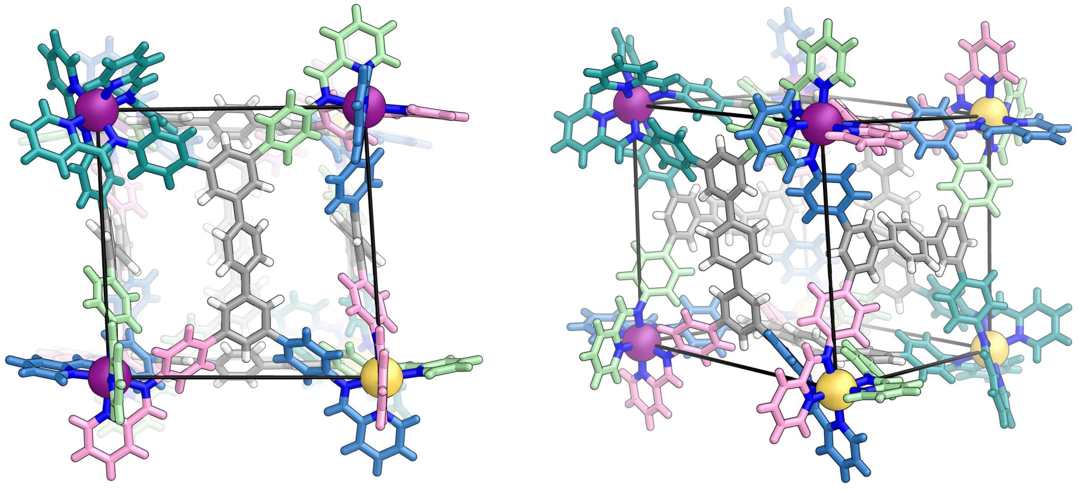

Figure S52. Views of the crystal structure of **5** with the four magnetically distinct ligand arms colored differently, corresponding to idealized S_6 point symmetry.

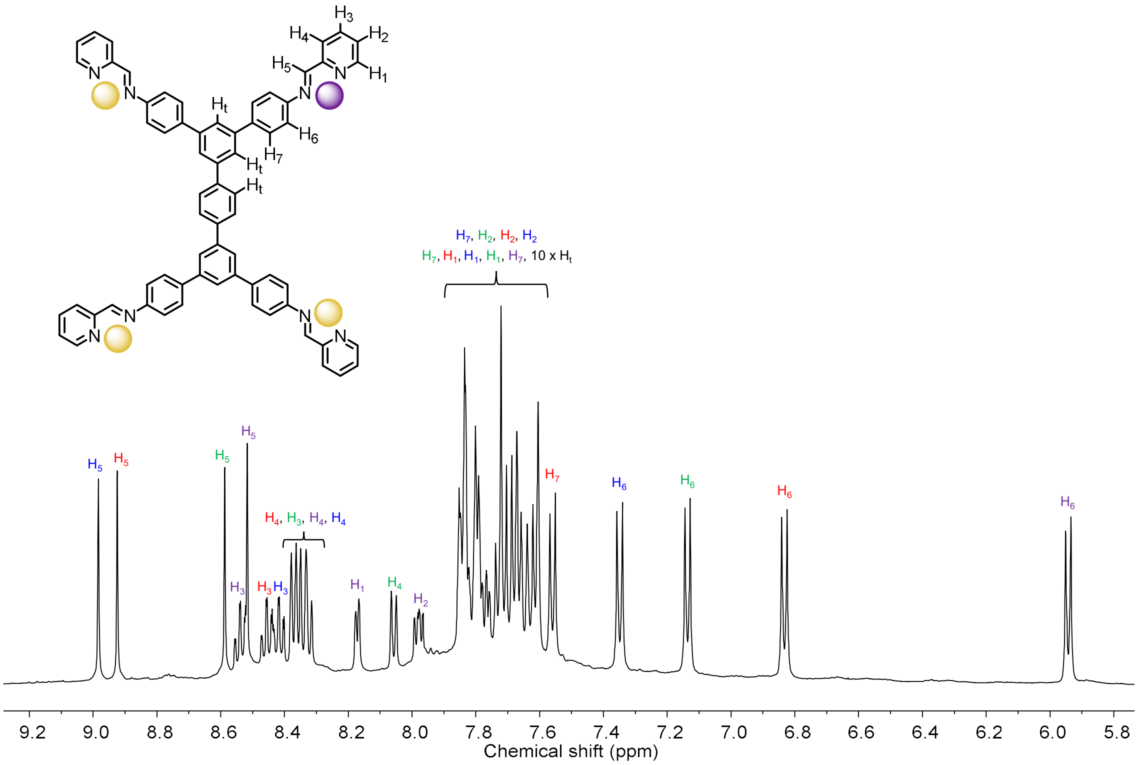

Figure S53. Aromatic region of the ^{1}H NMR spectrum (500 MHz, CD_3CN, 298 K) of **5**·$(NTf_2)_{16}$, with assignment of signals. The signals for each unique ligand arm could be identified and are labeled with different colors; however, each set could not be conclusively assigned to a specific arm in the structure. Each of the ten inequivalent protons on the central terphenyl core of the ligand are labeled H_t.

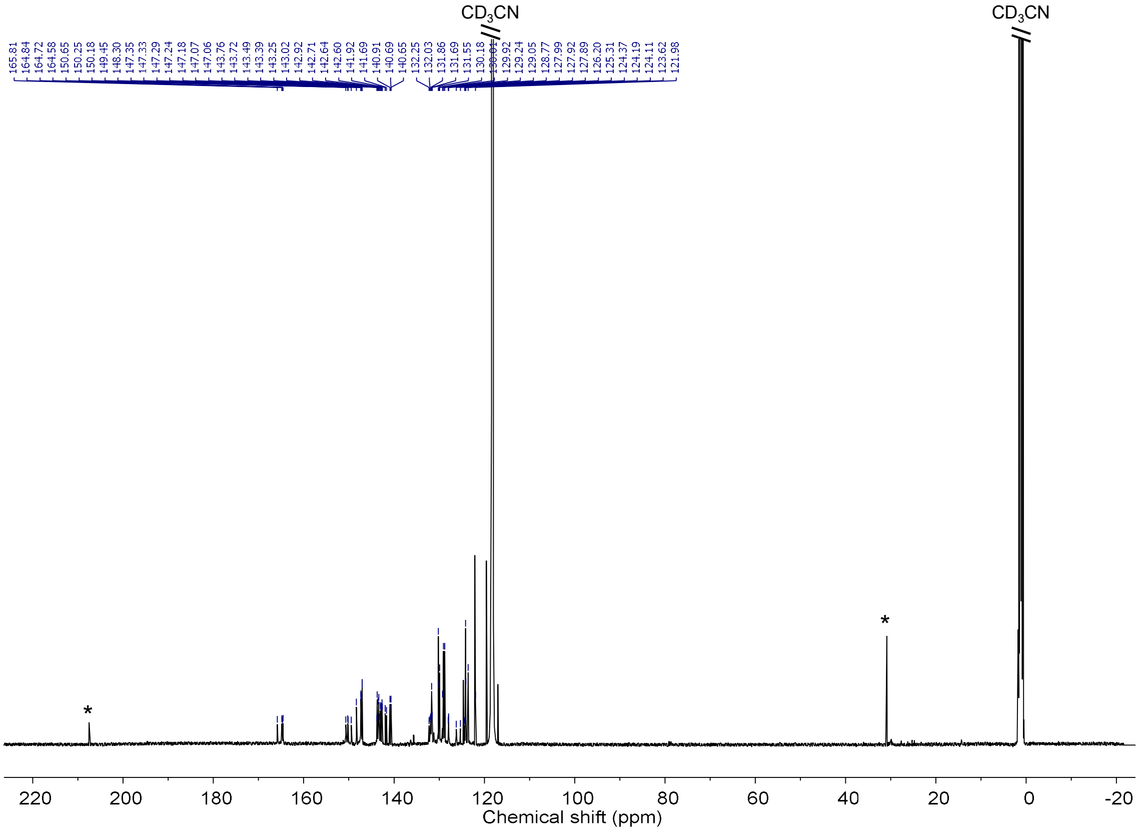

Figure S54. ^{13}C NMR spectrum (126 MHz, CD_3CN, 298 K) of **5**·$(NTf_2)_{16}$. (*indicates signals for the ^{13}C environments in acetone, present as an impurity).

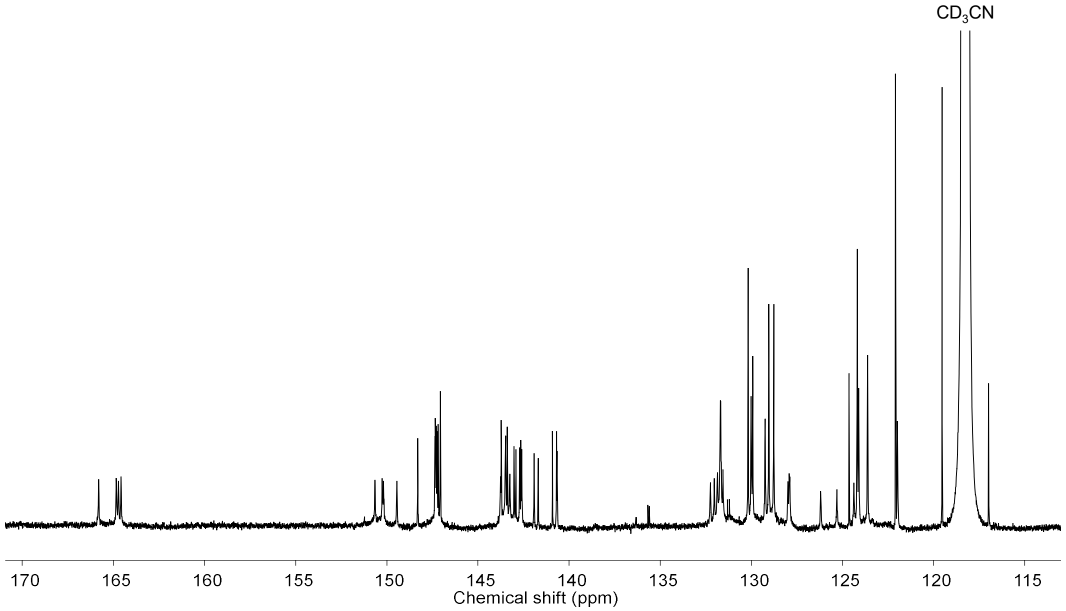

Figure S55. Aromatic region of the ^{13}C NMR spectrum (126 MHz, CD_3CN, 298 K) of **5**·$(NTf_2)_{16}$.

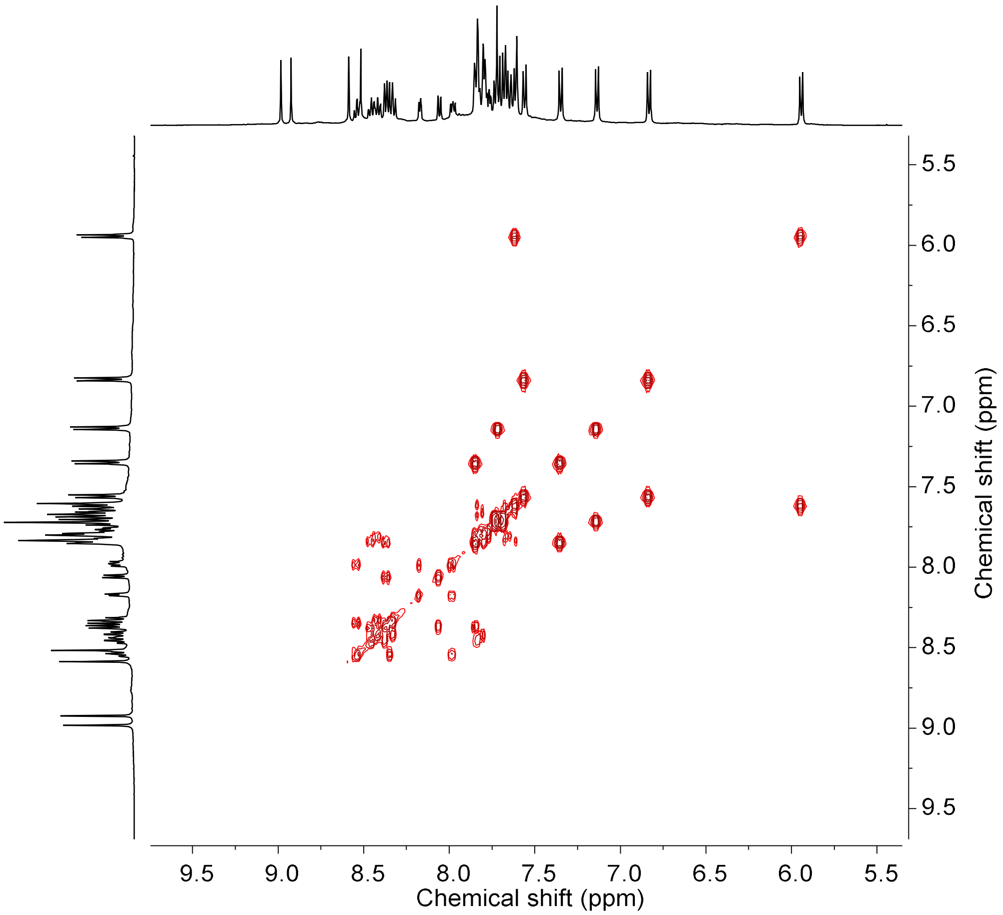

Figure S56. Aromatic region of the ^1H-^1H DQF-COSY spectrum (500 MHz, CD_3CN, 298 K) of **5**·$(NTf_2)_{16}$.

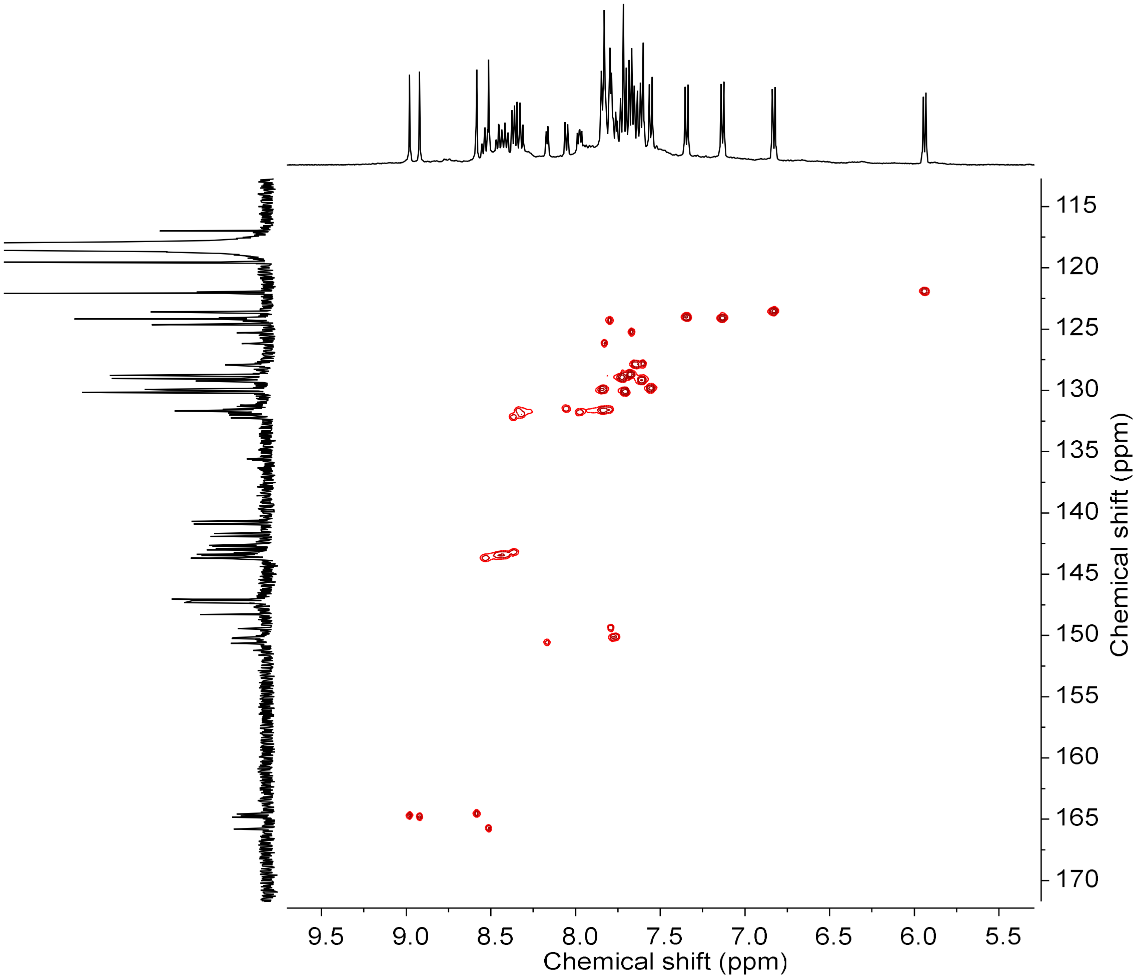

Figure S57. Aromatic region of the ^{1}H-^{13}C edited HSQC spectrum (500 MHz, CD_3CN, 298 K) of **5**·$(NTf_2)_{16}$.

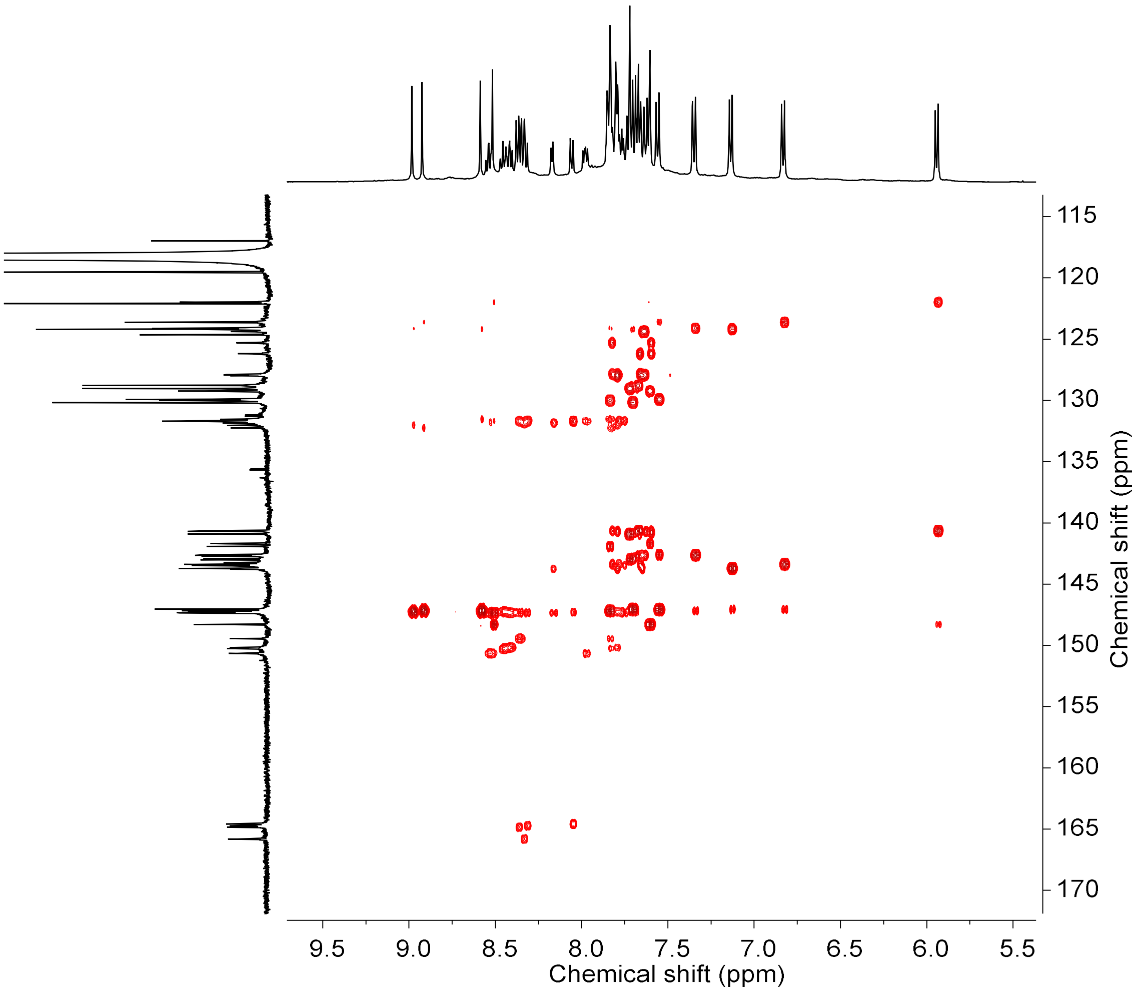

Figure S58. Aromatic region of the ^{1}H-^{13}C HMBC spectrum (500 MHz, CD_3CN, 298 K) of **5**·$(NTf_2)_{16}$.

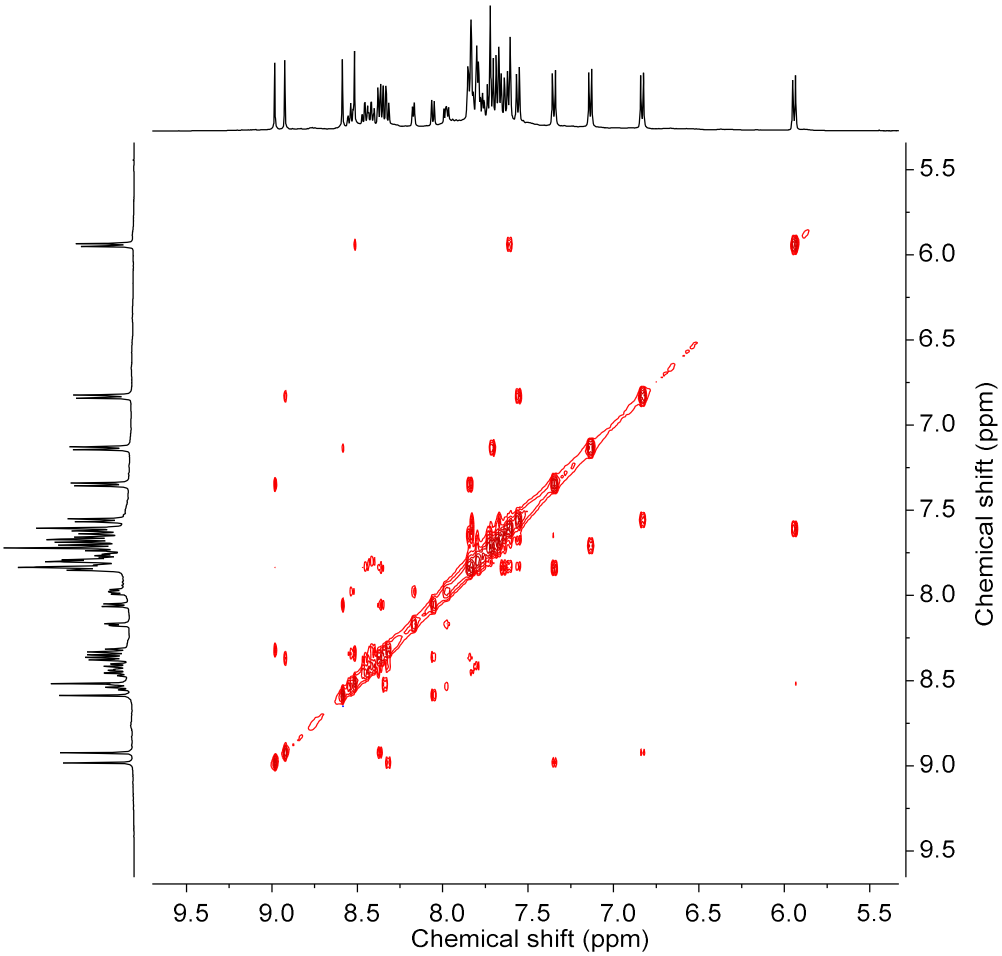

Figure S59. Aromatic region of the ^1H-^1H NOESY spectrum (500 MHz, CD_3CN, 298 K) of **5**·$(NTf_2)_{16}$.

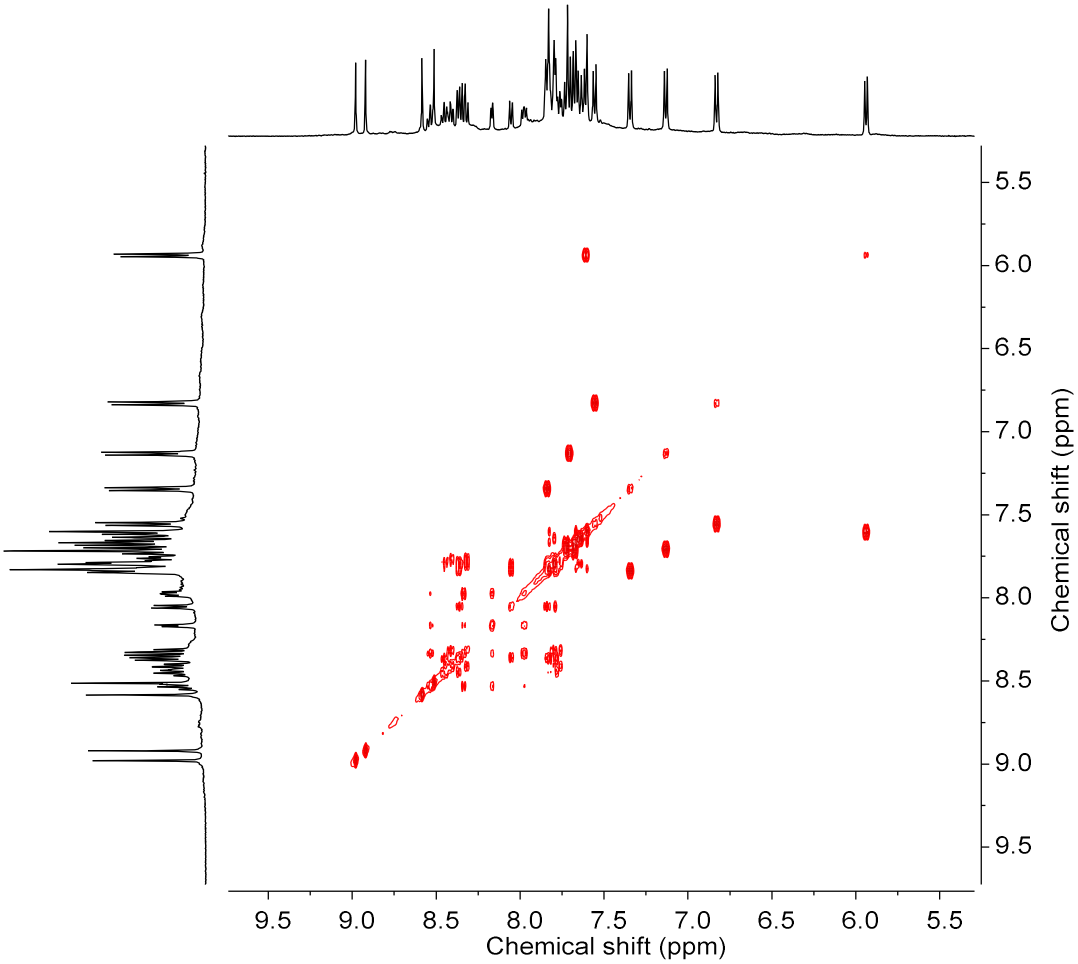

Figure S60. Aromatic region of the ^{1}H-^{1}H TOCSY spectrum (500 MHz, CD_3CN, 298 K) of **5**·$(NTf_2)_{16}$.

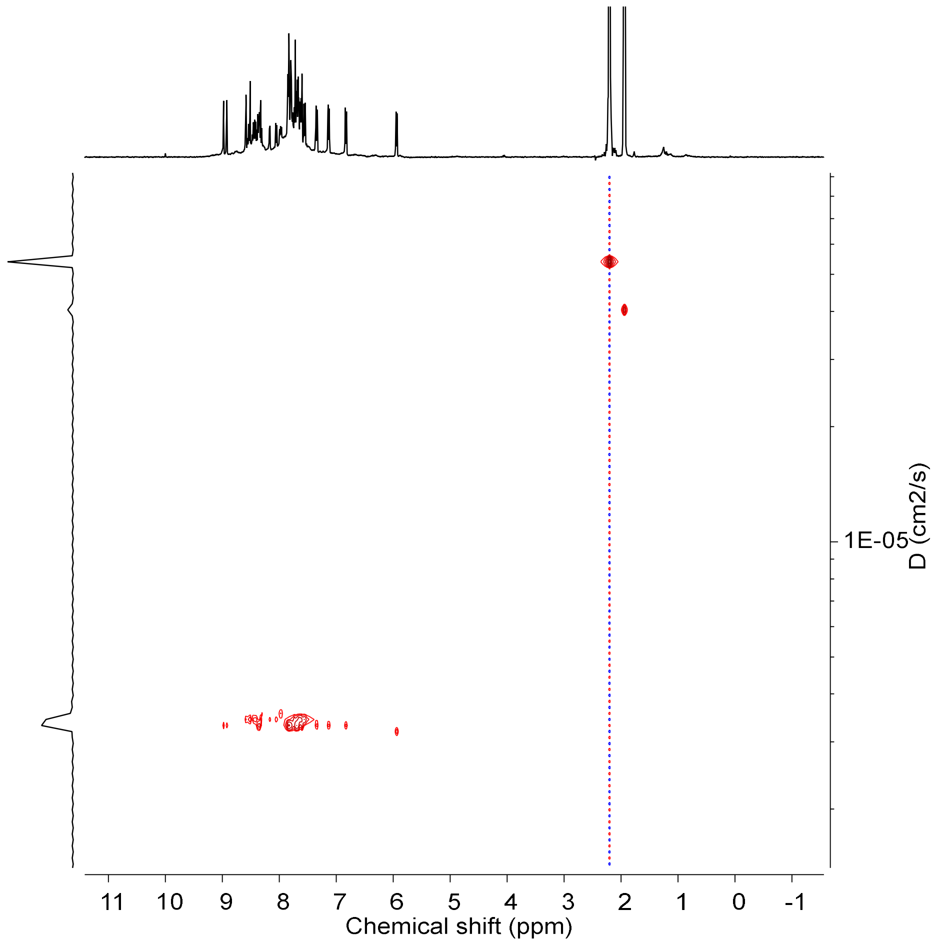

Figure S61. ^{1}H DOSY spectrum (400 MHz, CD_3CN, 298 K) of **5**·$(NTf_2)_{16}$.

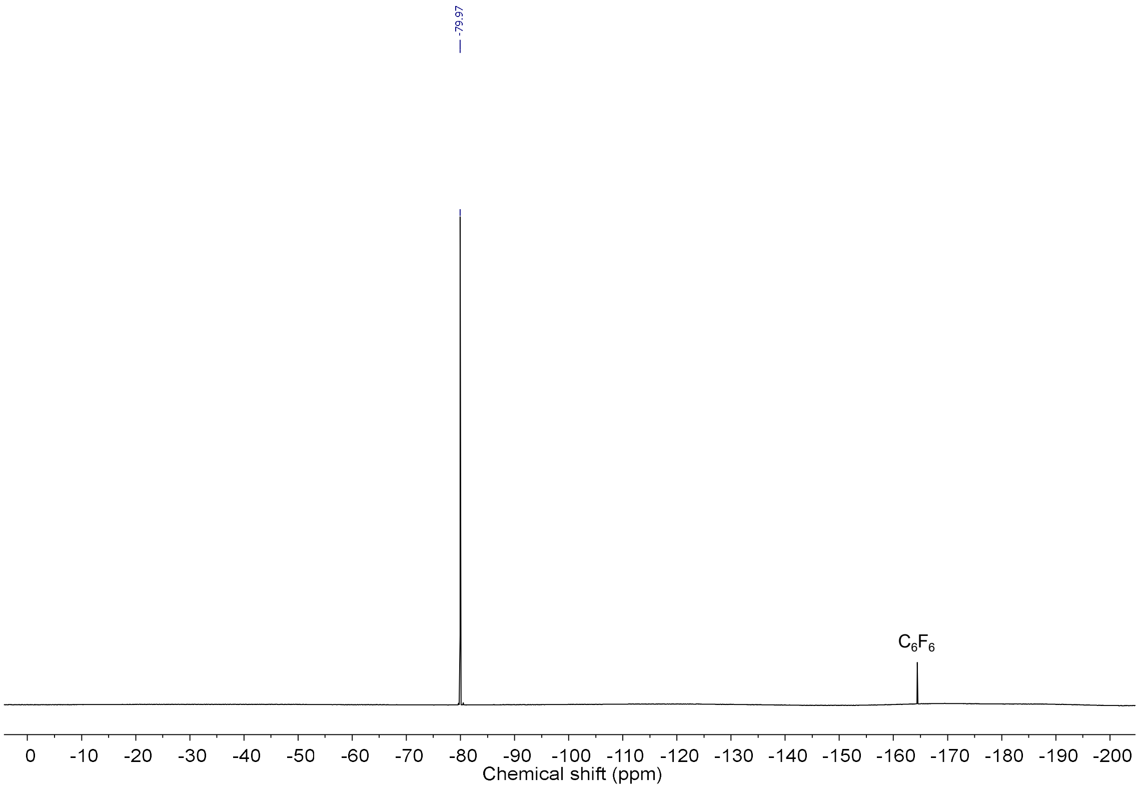

Figure S62. ^{19}F NMR spectrum (376 MHz, CD_3CN, 298 K) of **5**·$(NTf_2)_{16}$.

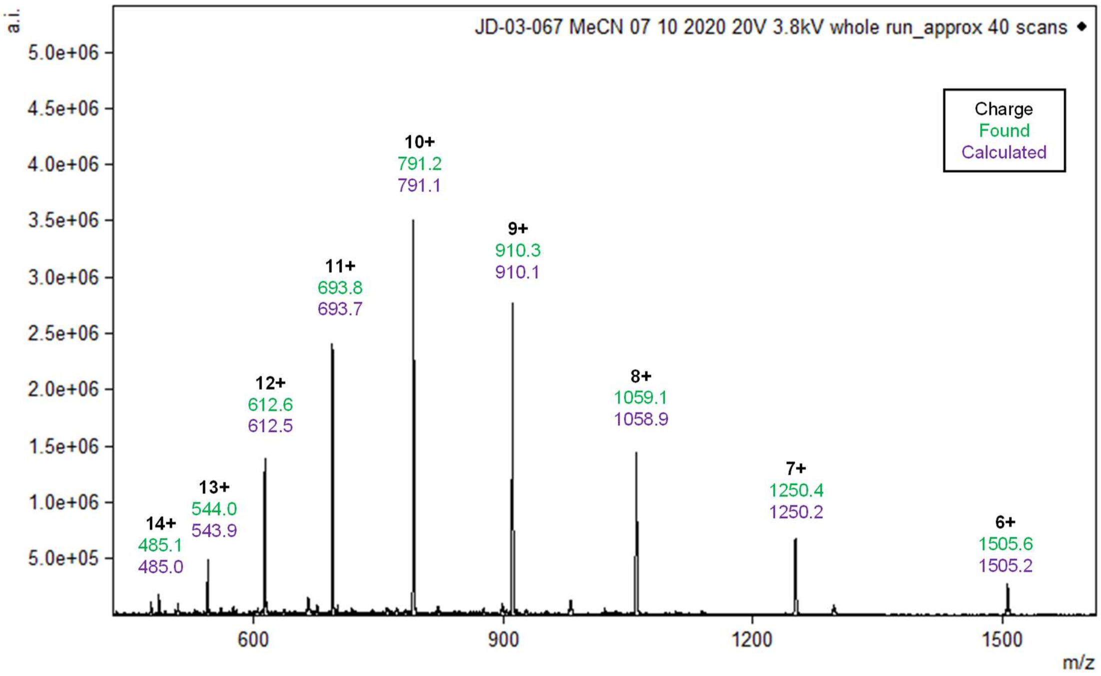

Figure S63. Low resolution ESI-mass spectrum for **5**·$(NTf_2)_{16}$.

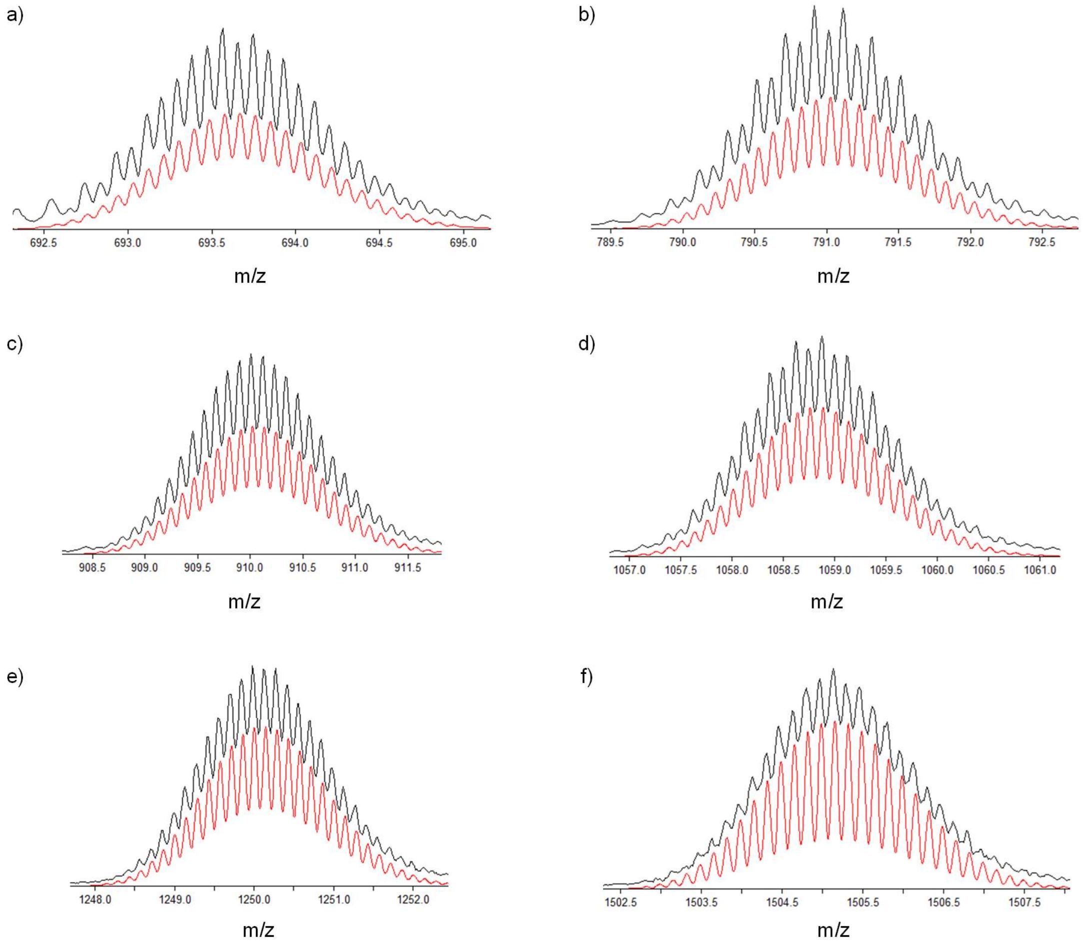

Figure S64. Signals from the high resolution ESI-mass spectrum for **5**·$(NTf_2)_{16}$. Experimental (black) and calculated (red) signals for a) [**5**$(NTf_2)_5]^{11+}$ b) [**5**$(NTf_2)_6]^{10+}$ c) [**5**$(NTf_2)_7]^{9+}$ d) [**5**$(NTf_2)_8]^{8+}$ e) [**5**$(NTf_2)_9]^{7+}$ f) [**5**$(NTf_2)_{10}]^{6+}$.

3.5 Synthesis and characterization of 6

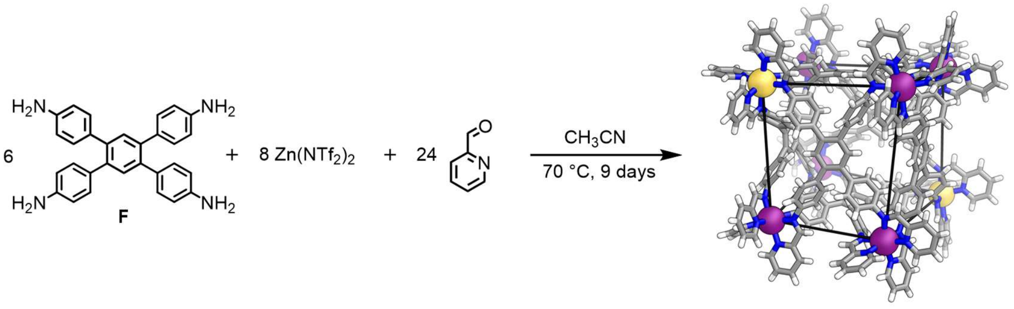

6

Scheme S5. Subcomponent self-assembly of **6**·$(NTf_2)_{16}$.

To a Schlenk flask, subcomponent **F** (5.0 mg, 11 µmol, 1.0 equiv), $Zn(NTf_2)_2$ (10.5 mg, 17 µmol, 1.5 equiv) and distilled acetonitrile (2.5 mL) were added. 2-formylpyridine (4.6 µL, 48 µmol, 4.4 equiv)

was added, the mixture was degassed by conducting four freeze-pump-thaw cycles, and heated at 70 °C under a nitrogen atmosphere for 9 days. The reaction mixture was allowed to cool to room temperature, filtered through a glass fibre plug, concentrated to a small volume using a stream of nitrogen, and diethyl ether (ca. 8 mL) was added. The precipitate was collected using centrifugation and washed with diethyl ether (4 × 8 mL). The product **6**·$(NTf_2)_{16}$ was obtained as a yellow solid (16.7 mg, 1.7 µmol, 93%).

^{1}H NMR (500 MHz, CD_3CN, 298 K) δ 8.58–8.53 (m, 18H), 8.51–8.46 (m, 12H), 8.43 (d, *J* = 7.8 Hz, 6H), 8.39–8.36 (m, 12H), 8.29 (d, *J* = 7.7 Hz, 6H), 8.23 (td, *J* = 7.4, 2.3 Hz, 6H), 8.13 (d, *J* = 5.1 Hz, 6H), 8.09–8.06 (m, 12H), 8.01–7.96 (m, 12H), 7.93–7.87 (m, 18H), 7.78–7.73 (m, 12H), 7.36 (s, 6H), 7.32–7.25 (m, 18H), 7.02 (d, *J* = 8.4 Hz, 12H), 5.98 (d, *J* = 8.7 Hz, 12H), 5.71 (d, *J* = 8.2 Hz, 12H). 48H could not be identified due to the intermediate rate of rotation of some of the phenyl rings on the NMR timescale, Figure S68 addresses this.

^{13}C NMR (126 MHz, CD_3CN, 298 K) δ 166.1, 165.2, 164.8, 164.3, 150.7, 150.6, 150.6, 150.5, 149.8, 147.7, 147.5, 147.4, 147.2, 147.2, 147.1, 146.7, 146.5, 143.8, 143.7, 143.5, 143.2, 142.1, 142.0, 141.4, 141.3, 140.8, 139.9, 139.2, 138.5, 135.0, 132.5, 132.1, 131.9, 131.9, 131.8, 131.7, 131.7, 131.6, 131.3, 122.7, 121.4, 120.8 (q, *J* = 321 Hz). (Some of the expected peaks are not detected, likely due to signal overlap and dynamic processes occurring at room temperature).

^{19}F NMR (376 MHz, CD_3CN, 298 K) δ −79.39, −79.90.

ESI-LRMS ([**6**$(NTf_2)_{16}$] = $C_{324}H_{228}N_{48}Zn_8(C_2F_6NO_4S_2)_{16}$) *m/z* = 610.8 [**6**$(NTf_2)_5]^{11+}$ (calc. 610.7), 699.8 [**6**$(NTf_2)_6]^{10+}$ (calc. 699.8), 808.7 [**6**$(NTf_2)_7]^{9+}$ (calc. 808.6), 944.8 [**6**$(NTf_2)_8]^{8+}$ (calc. 944.7), 1119.8 [**6**$(NTf_2)_9]^{7+}$ (calc. 1119.7), 1353.1 [**6**$(NTf_2)_{10}]^{6+}$ (calc. 1353.0), 1680.0 [**6**$(NTf_2)_{11}]^{5+}$ (calc. 1679.7).

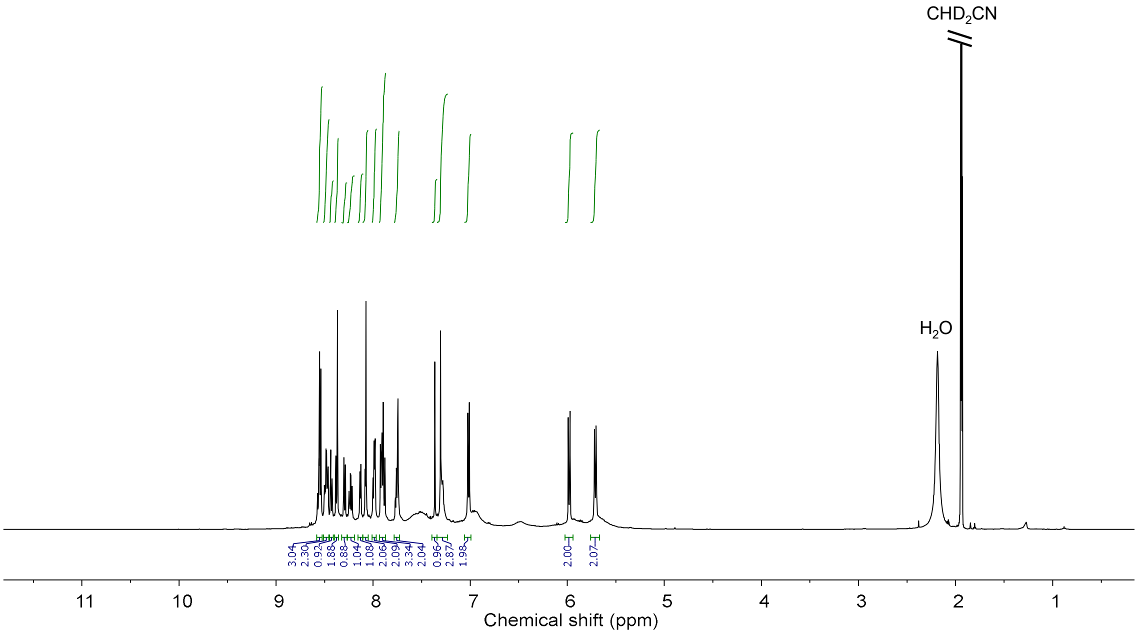

Figure S65. ^{1}H NMR spectrum (500 MHz, CD_3CN, 298 K) of **6**·$(NTf_2)_{16}$.

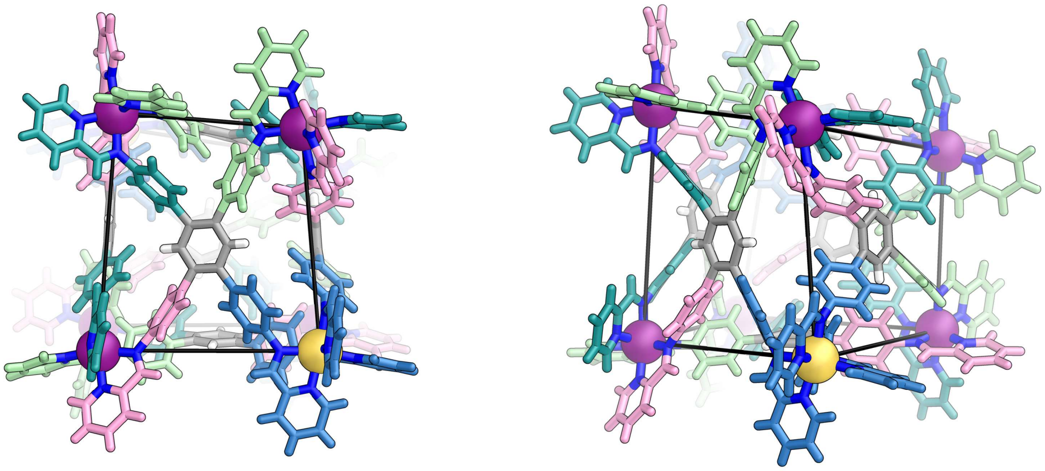

Figure S66. Views of the crystal structure of **6** with the four magnetically distinct ligand arms colored differently, in idealized D_3 point symmetry.

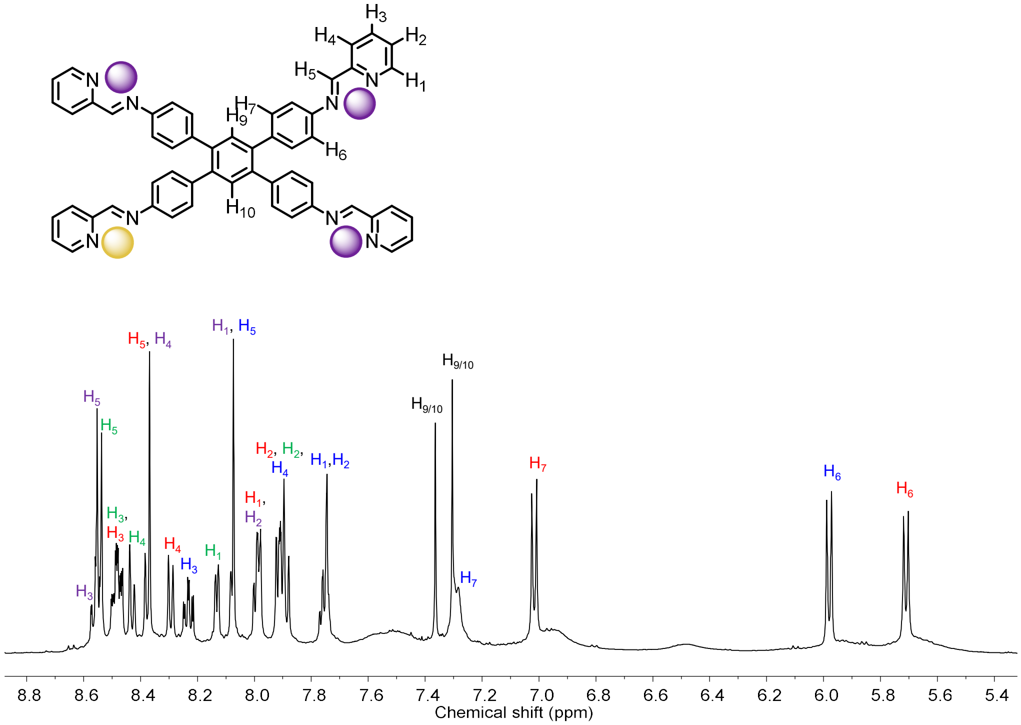

Figure S67. Aromatic region of the ^{1}H NMR spectrum (500 MHz, CD_3CN, 298 K) of **6**·$(NTf_2)_{16}$, with assignment of signals. The signals for each unique ligand arm could be identified (with the exception of two H_6 and two H_7 proton environments, see below) and are labeled with different colors; however, each set could not be conclusively assigned to a specific arm in the structure. The broadening of the signal for H_7 is attributed to the intermediate rate of rotation on the NMR timescale of the phenyl ring that this proton is attached to.

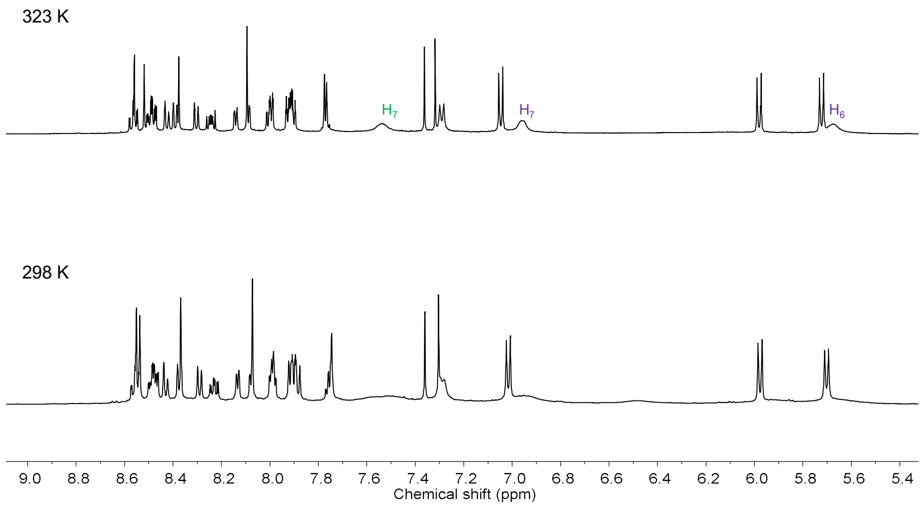

Figure S68. Aromatic region of the ^{1}H NMR spectra (500 MHz, CD_3CN) of **6**·$(NTf_2)_{16}$ at 298 K and 323 K. At 298 K, several signals for H_6 and H_7 proton environments are broad, and cannot be identified. At increased temperature H_6, H_7 and H_7 can be located. The signal for H_6 remained challenging to locate and we infer that the signal for this proton environment remains very broad even at increased temperature. Decreasing the temperature at which the ^{1}H NMR spectrum was recorded did not help identify the signal for H_6. The broadening of the signals for the H_6 and H_7 proton environments is attributed to the intermediate rate of rotation on the NMR timescale of the phenyl rings that these protons are attached to.

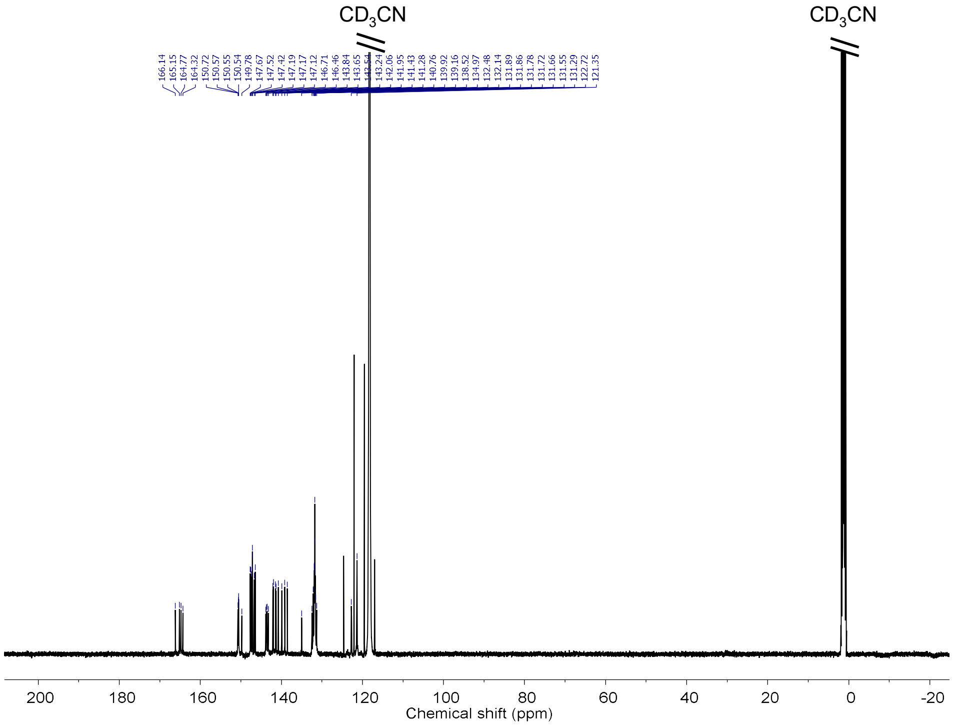

Figure S69. ^{13}C NMR spectrum (126 MHz, CD_3CN, 298 K) of **6**·$(NTf_2)_{16}$.

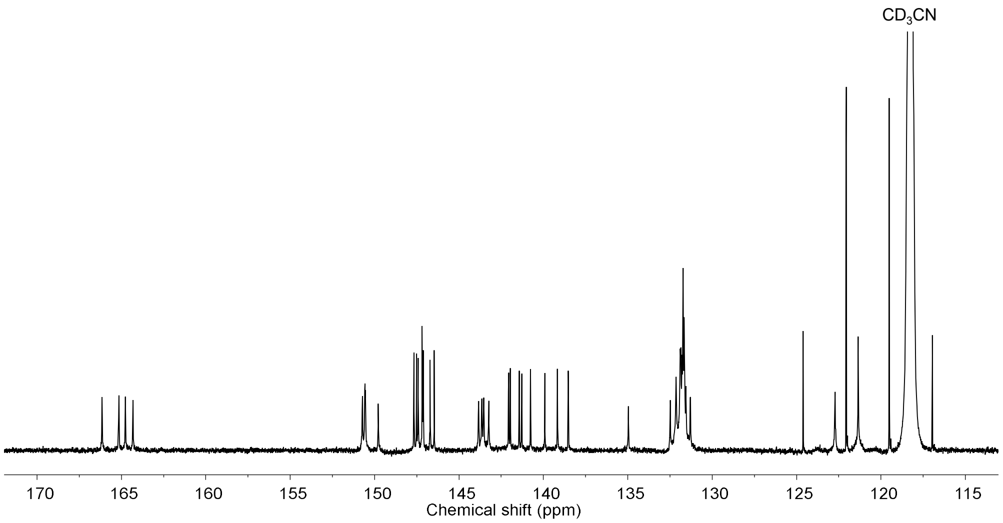

Figure S70. Aromatic region of the ^{13}C NMR spectrum (126 MHz, CD_3CN, 298 K) of **6**·$(NTf_2)_{16}$.

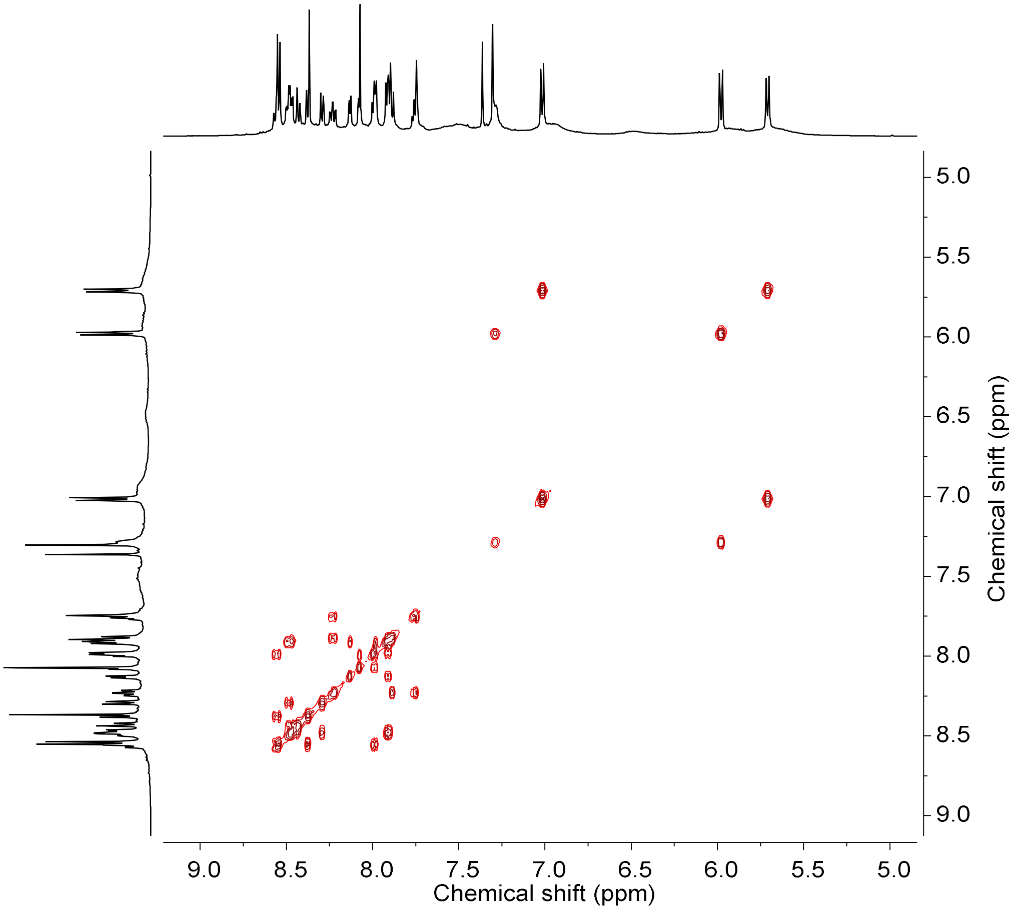

Figure S71. Aromatic region of the ^1H-^1H DQF-COSY spectrum (500 MHz, CD_3CN, 298 K) of **6**·$(NTf_2)_{16}$.

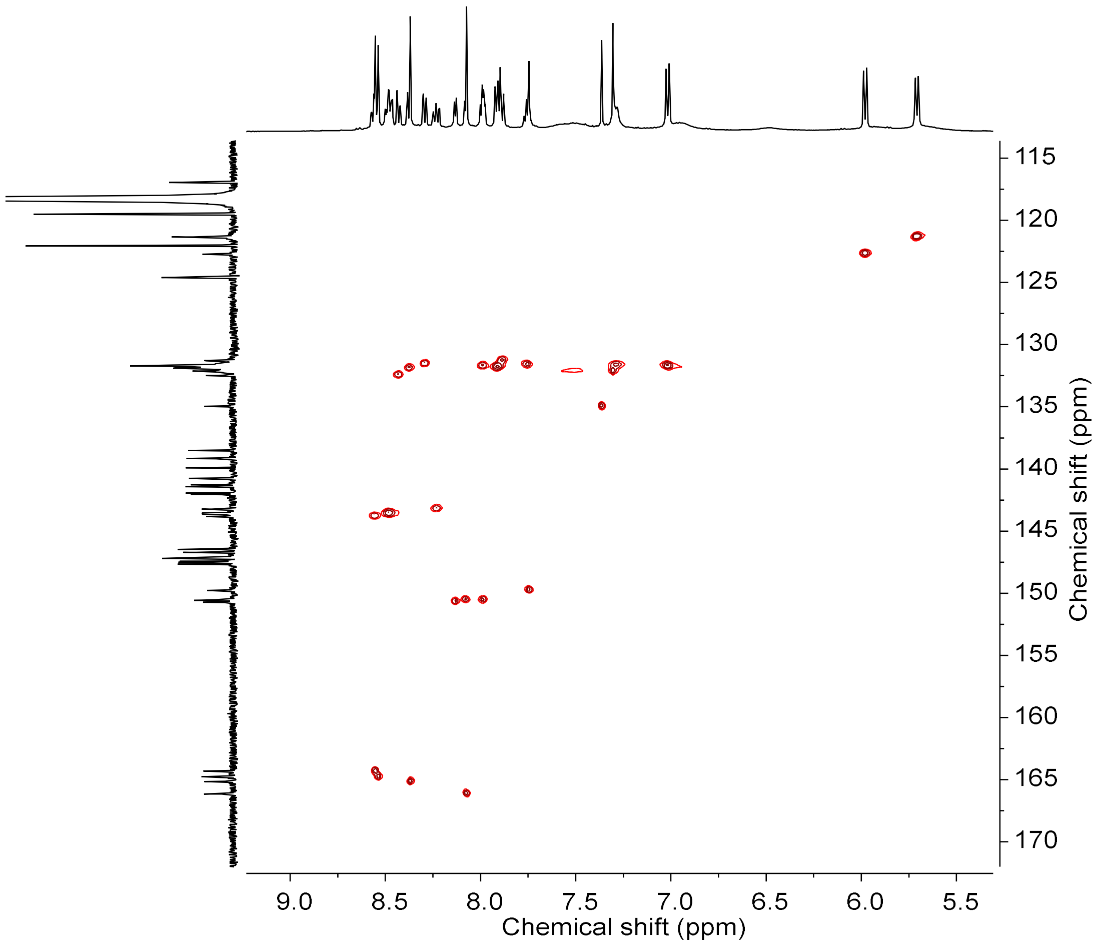

Figure S72. Aromatic region of the ^1H-^{13}C edited HSQC spectrum (500 MHz, CD_3CN, 298 K) of **6**·$(NTf_2)_{16}$.

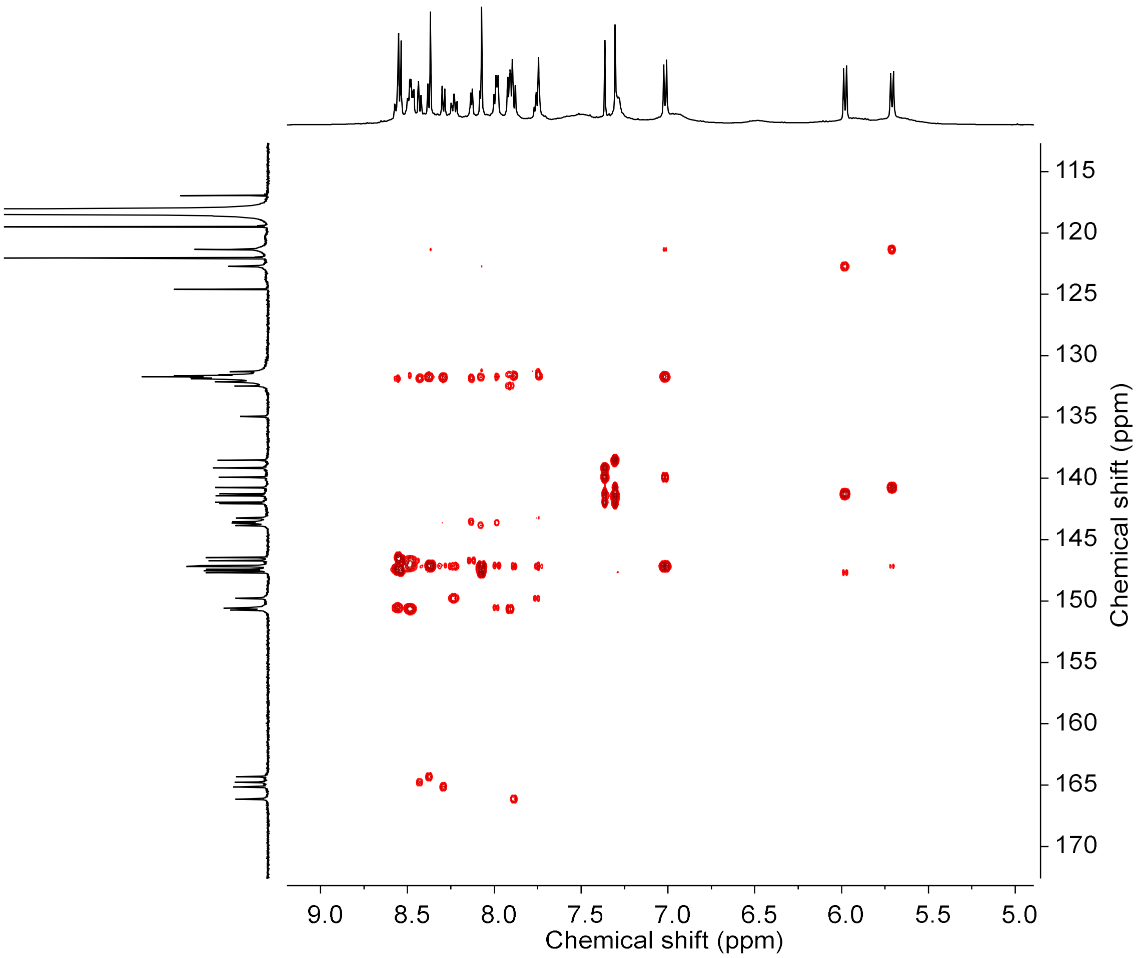

Figure S73. Aromatic region of the ^{1}H-^{13}C HMBC spectrum (500 MHz, CD_3CN, 298 K) of **6**·$(NTf_2)_{16}$.

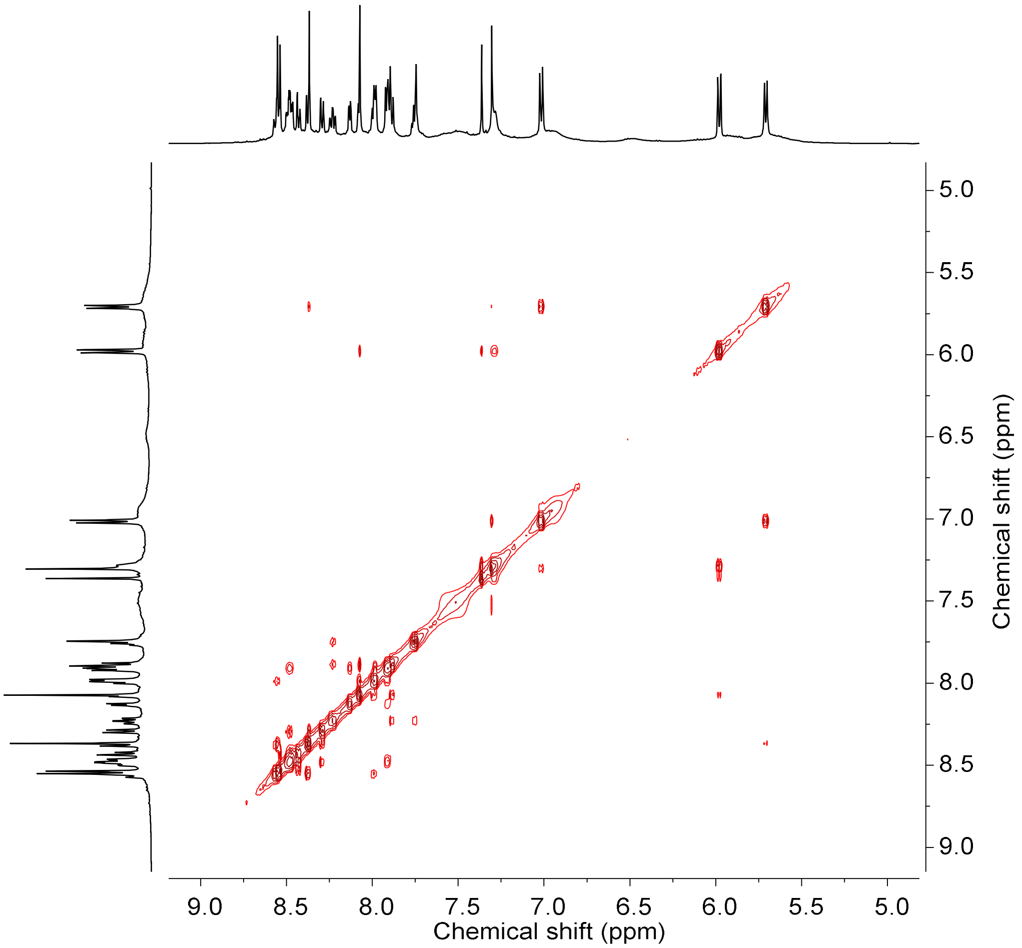

Figure S74. Aromatic region of the ^{1}H-^{1}H NOESY spectrum (500 MHz, CD_3CN, 298 K) of **6**·$(NTf_2)_{16}$.

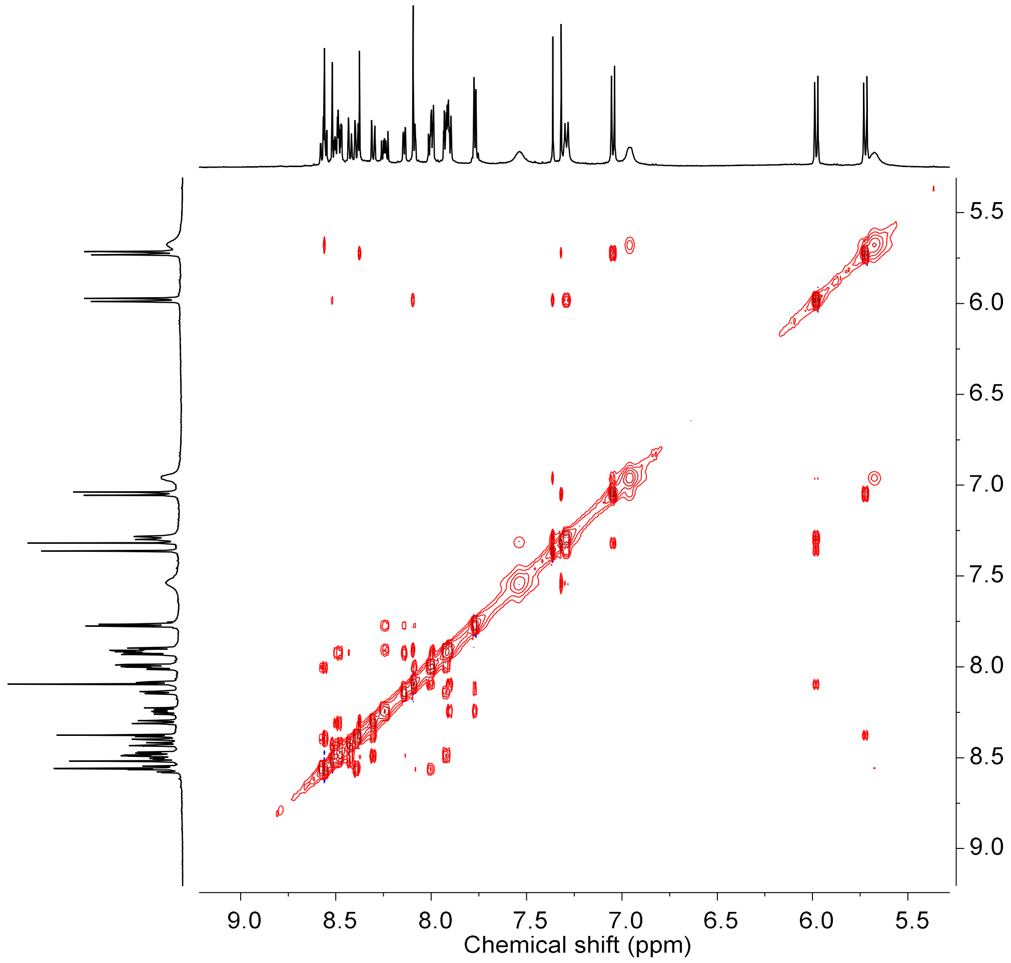

Figure S75. Aromatic region of the ^{1}H-^{1}H NOESY spectrum (500 MHz, CD_3CN, 323 K) of **6**·$(NTf_2)_{16}$.

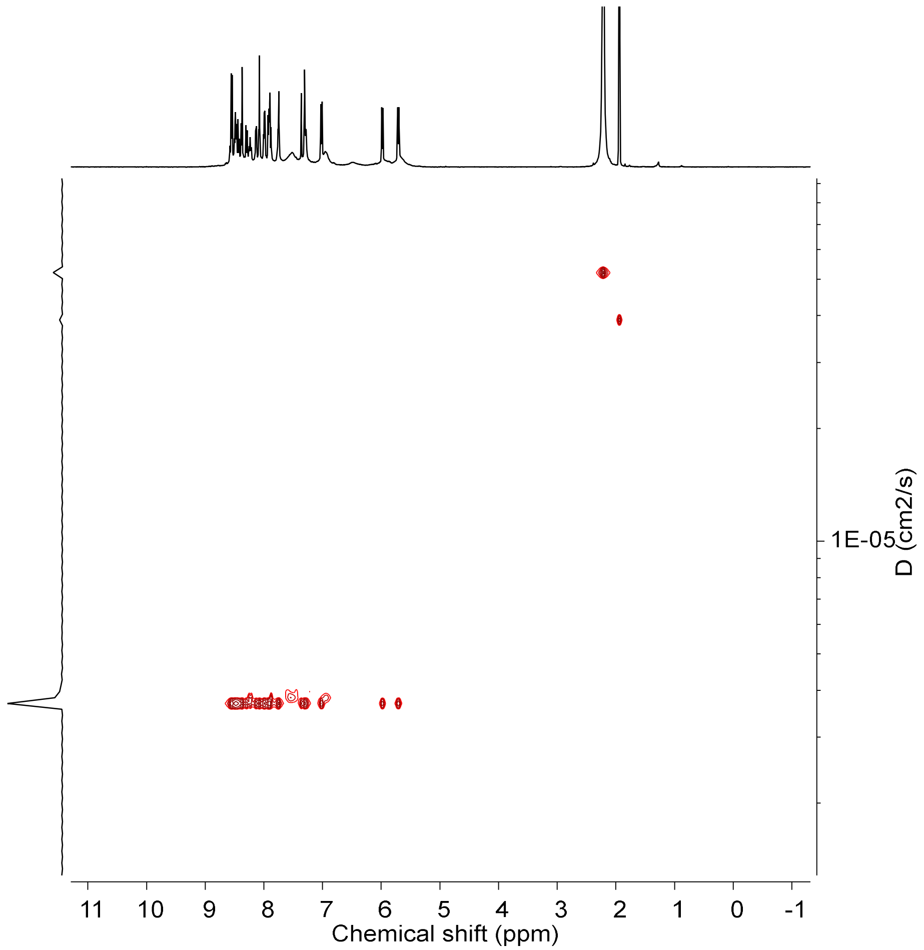

Figure S76. ^{1}H DOSY spectrum (400 MHz, CD_3CN, 298 K) of **6**·$(NTf_2)_{16}$.

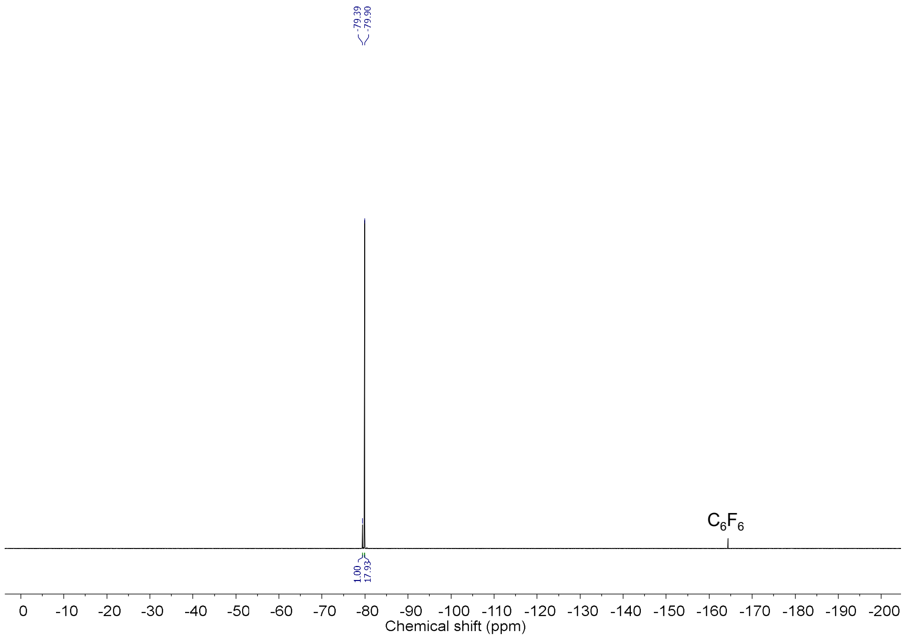

Figure S77. ^{19}F NMR spectrum (376 MHz, CD_3CN, 298 K) of **6**·$(NTf_2)_{16}$. The two peaks at δ = −79.39 and −79.90 ppm are attributed to bound and unbound NTf_2^-, respectively.

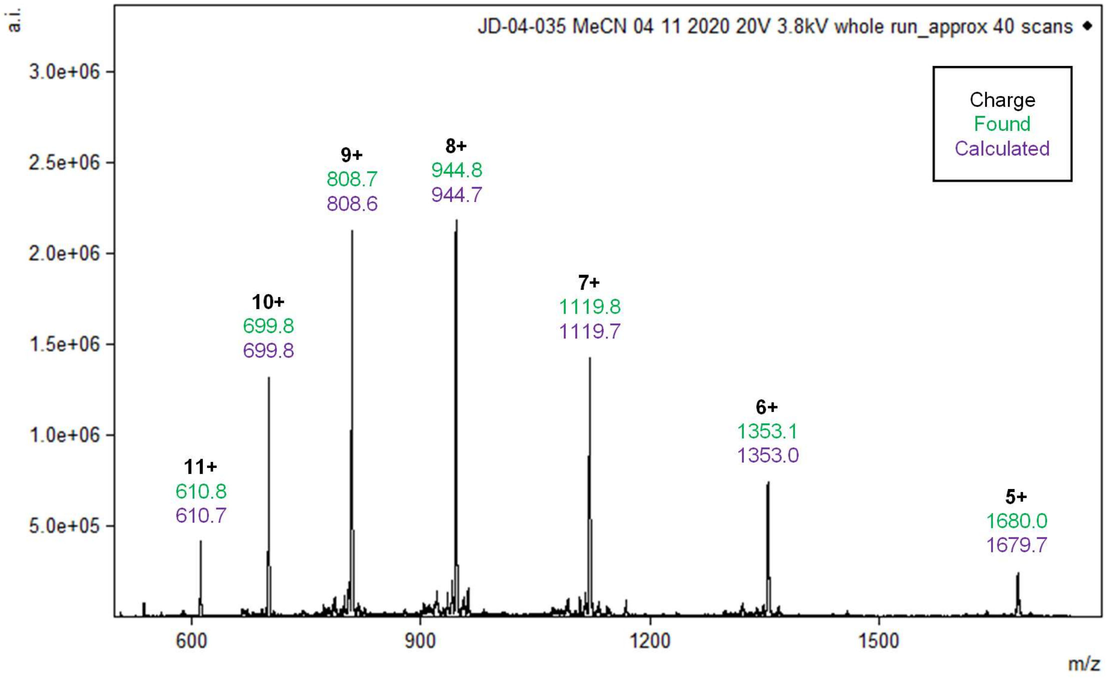

Figure S78. Low resolution ESI-mass spectrum for **6**·$(NTf_2)_{16}$.

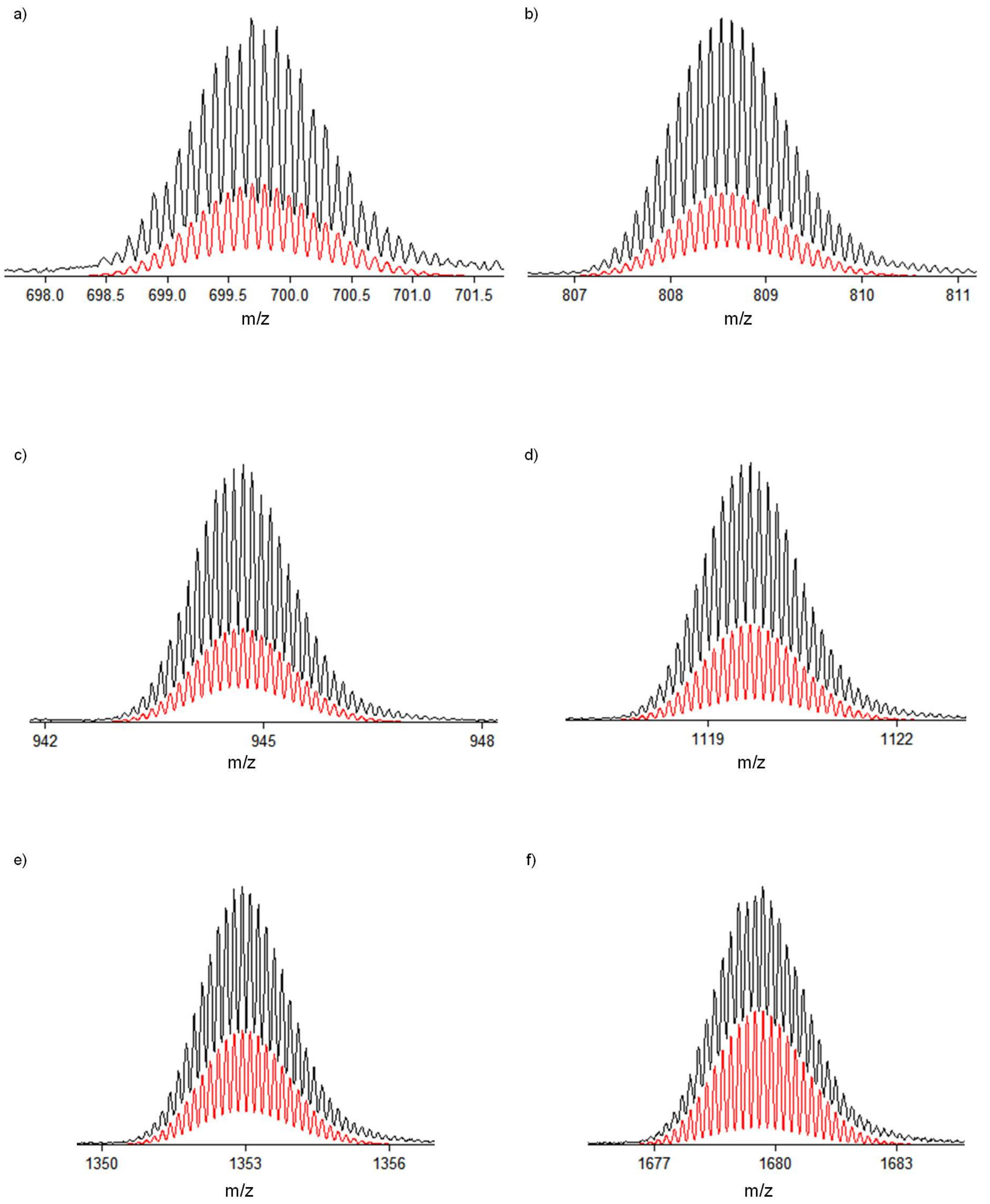

Figure S79. Signals from the high resolution ESI-mass spectrum for **6**·$(NTf_2)_{16}$. Experimental (black) and calculated (red) signals for a) [**6**$(NTf_2)_6]^{10+}$ b) [**6**$(NTf_2)_7]^{9+}$ c) [**6**$(NTf_2)_8]^{8+}$ d) [**6**$(NTf_2)_9]^{7+}$ e) [**6**$(NTf_2)_{10}]^{6+}$ f) [**6**$(NTf_2)_{11}]^{5+}$.

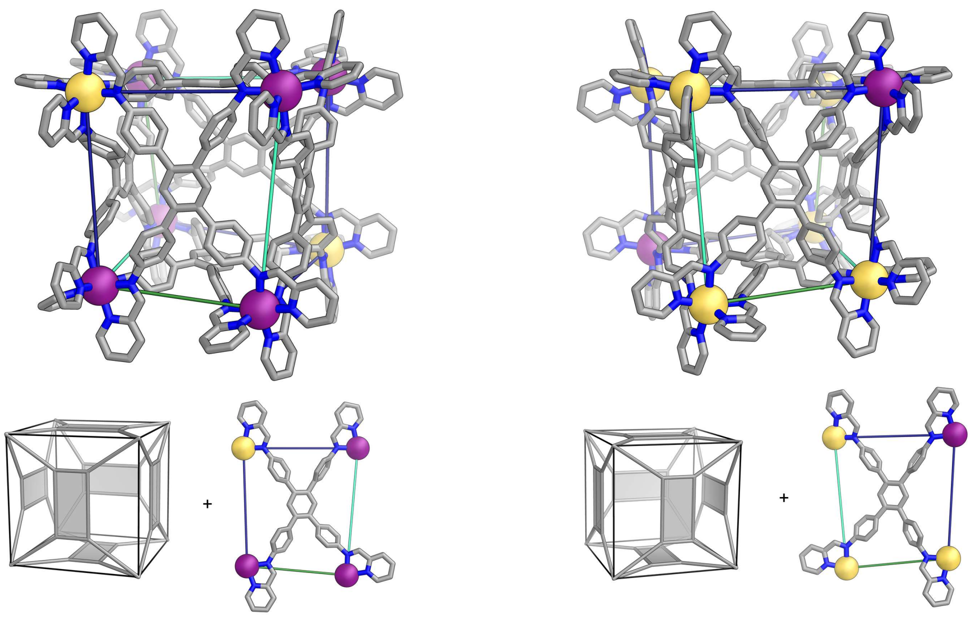

Figure S80. The two enantiomers of **6** present in the crystal structure.

4 X-ray crystallography

Data were collected at Beamline I19 of Diamond Light Source employing silicon double crystal monochromated synchrotron radiation (0.6889 Å) with ω and ψ scans at 100(2) K.[6] Data integration and reduction were undertaken with Xia2.[7-9] Subsequent computations were carried out using the WinGX-32 graphical user interface.[10] Multi-scan empirical absorption corrections were applied to the data using the AIMLESS[11] tool in the CCP4 suite.[12] The structures were solved by direct methods using SHELXT[13] then refined and extended with SHELXL.[14] In general, non-hydrogen atoms with occupancies greater than 0.5 were refined anisotropically. Carbon-bound hydrogen atoms were included in idealized positions and refined using a riding model. Disorder was modelled using standard crystallographic methods including constraints, restraints and rigid bodies where necessary. Crystallographic data along with specific details pertaining to the refinement follow. Crystallographic data have been deposited with the CCDC (2179381–2179385).

1·16BF$_4$·6.5CH$_3$CN [+ solvent]

Formula $C_{337}H_{259.50}B_{16}F_{64}N_{66.50}Zn_8$, *M* 7152.52, Triclinic, space group P -1 (#2), *a* 20.8181(6), *b* 22.8600(8), *c* 42.6867(14) Å, α 90.355(3), β 97.922(3), γ 95.178(3)°, *V* 20035.1(11) Å^3, D_c 1.186 g cm^{-3}, *Z* 2, crystal size 0.200 by 0.100 by 0.050 mm, color red brown, habit block, temperature 100(2) Kelvin, λ(synchrotron) 0.6889 Å, μ(synchrotron) 0.510 mm^{-1}, *T*(Analytical)$_{min,max}$ 0.992936602510474, 1.0, 2θ_{max} 40.30, *hkl* range -20 20, -22 22, -42 42, *N* 170976, N_{ind}

41924(R_{merge} 0.0768), N_{obs} 17539(I > 2σ(I)), N_{var} 4539, residuals* $R1(F)$ 0.1278, $wR2(F^2)$ 0.3788, GoF(all) 1.124, $\Delta\rho_{min,max}$ -0.695, 2.232 e$^-$ Å^{-3}.

*$R1 = \Sigma||F_o| - |F_c||/\Sigma|F_o|$ for $F_o > 2\sigma(F_o)$; $wR2 = (\Sigma w(F_o^2 - F_c^2)^2/\Sigma(wF_c^2)^2)^{1/2}$ all reflections

$w=1/[\sigma^2(F_o^2)+(0.2000P)^2]$ where $P=(F_o^2+2F_c^2)/3$

Specific refinement details:

The crystals of **1**·$16BF_4$·6.5MeCN [+ solvent] were grown by diffusion of diethyl ether into an acetonitrile solution of **1**·$16NTf_2$ containing excess ($^nBu_4N$)·BF_4. The crystals employed immediately lost solvent after removal from the mother liquor and rapid handling prior to flash cooling in liquid nitrogen was required to collect data. Despite these measures and the use of synchrotron radiation few reflections at greater than 1.0 Å resolution were observed and the data were trimmed accordingly. Furthermore there was a significant drop-off in diffraction intensity after around 1.3 Å resolution resulting in a low ratio of observed/unique reflections. Nevertheless, the quality of the data is far more than sufficient to establish the connectivity of the structure. The asymmetric unit was found to contain one complete Zn_8L_6 assembly and associated counterions and solvent molecules.

In order to obtain a reasonable model for the organic parts of the structure the GRADE program[15] was thus employed, using the GRADE Web Server,[16] to generate a full set of bond distance and angle restraints (DFIX, DANG, FLAT). Due to the thermal motion and less than ideal resolution, thermal parameter restraints (SIMU, RIGU) were applied to all atoms except for zinc to facilitate anisotropic refinement. Several ligand sections were modelled as disordered over two locations with only the major occupancy parts refined anisotropically. The strongest remaining electron density peaks are close to the zinc atoms and may indicate minor unresolved disorder or may possibly arise from absorption effects.

The anions within the structure show evidence of substantial disorder. Five of the located BF_4^- anions were modelled as disordered over two or three locations. The BF_4^- anions were restrained to be approximately tetrahedral and most low occupancy anions were modelled with isotropic thermal parameters. The occupancies of all located anions were allowed to freely refine which resulted in a discrepancy of ca. 4.5 anions per Zn_8L_6 assembly. Bond length restraints were also applied to the acetonitrile solvent molecules which were mostly refined with partial occupancy and isotropic thermal parameters. The hydrogen atoms of some acetonitrile molecules could not be located in the electron density map and were therefore not included in the model.

Further reflecting the solvent loss and poor diffraction properties there is a significant amount of void volume in the lattice containing smeared electron density from disordered solvent and the remaining anions. Consequently the SQUEEZE[17] function of PLATON[18] was employed to remove the contribution of the electron density associated with these remaining anions and further highly disordered solvent, which gave a potential solvent accessible void of 4278 Å^3 per unit cell (a total of approximately 906

electrons). Diffuse solvent molecules could not be assigned to acetonitrile or diethyl ether and were therefore not included in the formula. Consequently, the molecular weight and density given above are underestimated.

CheckCIF gives one A and two B level alerts. These alerts result from the limited resolution of the data and the poor diffraction properties.

2·16ReO$_4$·7MeCN·13.333C$_6$H$_6$ [+ solvent]

Formula $C_{454}H_{365}N_{67}O_{64}Re_{16}Zn_8$, *M* 11285.27, Trigonal, space group R -3 (#148), *a* 33.94390(6), *b* 33.94390(6), *c* 32.52560(12) Å, γ 120°, *V* 32454.83(16) Å^3, D_c 1.732 g cm^{-3}, *Z* 3, crystal size 0.003 by 0.002 by 0.002 mm, color red brown, habit block, temperature 100(2) Kelvin, λ(Synchrotron) 0.6889 Å, μ(Synchrotron) 4.550 mm^{-1}, T(Analytical)$_{min,max}$ 0.9777026183527046, 1.0, $2\theta_{max}$ 64.00, *hkl* range -52 42, -51 51, -38 50, *N* 154585, N_{ind} 27468(R_{merge} 0.0453), N_{obs} 17741(I > 2σ(I)), N_{var} 1006, residuals* $R1(F)$ 0.0536, $wR2(F^2)$ 0.1794, GoF(all) 1.017, $\Delta\rho_{min,max}$ -0.943, 2.600 e$^-$ Å^{-3}.

*$R1 = \Sigma||F_o| - |F_c||/\Sigma|F_o|$ for $F_o > 2\sigma(F_o)$; $wR2 = (\Sigma w(F_o^2 - F_c^2)^2/\Sigma(wF_c^2)^2)^{1/2}$ all reflections

$w=1/[\sigma^2(F_o^2)+(0.1154P)^2]$ where $P=(F_o^2+2F_c^2)/3$

Specific refinement details:

The crystals of **2**·16ReO$_4$·7MeCN·13.333C$_6$H$_6$ [+ solvent] were grown by diffusion of benzene into an acetonitrile solution of **2**·16NTf$_2$ containing excess (^{n}Bu$_4$N)·ReO$_4$. The crystals employed immediately lost solvent after removal from the mother liquor. However rapid handling prior to flash cooling in liquid nitrogen enabled the collection of high resolution data using synchrotron radiation. The asymmetric unit was found to contain one sixth of a Zn$_8$L$_6$ assembly (i.e. one complete organic ligand) and associated counterions and solvent molecules. Thermal parameter restraints (SIMU, RIGU) were applied to all atoms except for zinc and rhenium.

The anions within the structure also show evidence of substantial disorder. All three located perrhenate anions were modelled as disordered over two or three locations. Substantial bond length and thermal parameter restraints were applied to facilitate a reasonable refinement of the disordered anions and most low occupancy oxygen atoms were modelled with isotropic thermal parameters. One perrhenate per Zn$_8$L$_6$ assembly (corresponding to only one sixth of a perrhenate per asymmetric unit) remains unaccounted for and no reasonable model could be obtained for it despite many attempts using restraints or rigid groups. Consequently the SQUEEZE[17] function of PLATON[18] was employed to remove the contribution of the electron density associated with this remaining highly disordered anion (and possibly a small amount of highly disordered solvent) which gave a potential solvent accessible void of 789 Å^3 per unit cell (a total of approximately 348 electrons).

CheckCIF gives one A and one B level alert, both resulting from electron density peaks close to the rhenium atoms which either arise from absorption effects or minor unresolved disorder.

3·16AsF$_6$·7MeCN [+ solvent]

Formula $C_{422}H_{285}As_{16}F_{96}N_{55}Zn_8$, *M* 9671.71, Triclinic, space group P-1 (#2), *a* 24.8925(4), *b* 25.7465(4), *c* 29.1725(5) Å, α 70.1250(10), β 70.0550(10), γ 79.2200(10)°, *V* 16474.8(5) Å^3, D_c 0.975 g cm^{-3}, *Z* 1, crystal size 0.050 by 0.040 by 0.020 mm, color orange, habit block, temperature 100(2) Kelvin, λ(Synchrotron) 0.6889 Å, μ(Synchrotron) 1.056 mm^{-1}, T(Analytical)$_{min,max}$ 0.9876220929407328, 1.0, $2\theta_{max}$ 45.00, *hkl* range -27 27, -28 28, -30 32, *N* 132741, N_{ind} 46290(R_{merge} 0.0611), N_{obs} 26460(I > 2σ(I)), N_{var} 2724, residuals* $R1(F)$ 0.1065, $wR2(F^2)$ 0.3446, GoF(all) 1.158, $\Delta\rho_{min,max}$ -0.741, 0.789 e$^-$ Å^{-3}.

*$R1 = \Sigma||F_o| - |F_c||/\Sigma|F_o|$ for $F_o > 2\sigma(F_o)$; $wR2 = (\Sigma w(F_o^2 - F_c^2)^2/\Sigma(wF_c^2)^2)^{1/2}$ all reflections

$w=1/[\sigma^2(F_o^2)+(0.2000P)^2]$ where $P=(F_o^2+2F_c^2)/3$

Specific refinement details:

The crystals of **3**·16AsF$_6$·7MeCN [+ solvent] were grown by diffusion of diisopropyl ether into an acetonitrile solution of **3**·16NTf$_2$ containing excess KAsF$_6$. The crystals employed immediately lost solvent after removal from the mother liquor and rapid handling prior to flash cooling in liquid nitrogen was required to collect data. Despite these measures and the use of synchrotron radiation few reflections at greater than 0.9 Å resolution were observed. The asymmetric unit was found to contain one half of a Zn_8L_6 assembly and associated counterions and solvent molecules.

Due to the limited resolution, bond lengths and angles within pairs of chemically identical organic ligands were restrained to be similar to each other and thermal parameter restraints (SIMU, RIGU) were applied to all atoms except for zinc and arsenic. Four of the ligand phenyl rings were modelled as disordered over two locations with bond length and angle restraints applied to achieve a reasonable model.

The anions within the structure also show evidence of disorder. Three hexafluoroarsenate anions were modelled as disordered over two locations and the occupancies of all located anions were freely refined. Substantial bond length and thermal parameter restraints were applied to facilitate a reasonable refinement of the disordered hexafluoroarsenate anions and solvent molecules, and most low occupancy disordered groups were modelled with isotropic thermal parameters.

Further reflecting the solvent loss and poor diffraction properties there is a significant amount of void volume in the lattice containing smeared electron density from disordered solvent and 5.8 anions per Zn_8L_6 assembly (assigned to hexafluoroarsenate in the formula). Consequently the SQUEEZE[17] function of PLATON[18] was employed to remove the contribution of the electron density associated with these remaining anions and further highly disordered solvent, which gave a potential solvent accessible void of 7722 Å^3 per unit cell (a total of approximately 2306 electrons). Diffuse solvent molecules could not be assigned to acetonitrile or diisopropyl ether and were therefore not included in the formula. Consequently, the molecular weight and density given above are underestimated.

CheckCIF gives two B level alerts, one resulting from the limited resolution of the data and one for reflections which may have been blocked by the beamstop.

5·16ClO$_4$·3MeCN·2H$_2$O [+ solvent]

Formula $C_{402}H_{289}Cl_{16}N_{51}O_{66}Zn_8$, *M* 7979.98, Trigonal, space group R -3 c (#167), 27.3930(3), *b* 27.3930(3), *c* 125.954(3) Å, γ 120°, *V* 81851(3) Å^3, D_c 0.971 g cm^{-3}, *Z* 9, crystal size 0.050 by 0.040 by 0.030 mm, color colorless, habit block, temperature 100(2) Kelvin, λ(Synchrotron) 0.6889 Å, μ(Synchrotron) 0.441 mm^{-1}, T(Analytical)$_{min,max}$ 0.9840774334282736, 1.0, $2\theta_{max}$ 40.30, *hkl* range -27 27, -27 27, -125 125, *N* 84603, N_{ind} 9546(R_{merge} 0.0653), N_{obs} 5368(I > 2σ(I)), N_{var} 1010, residuals* $R1(F)$ 0.1182, $wR2(F^2)$ 0.3740, GoF(all) 1.191, $\Delta\rho_{min,max}$ -0.372, 0.680 e$^-$ Å^{-3}.

*$R1 = \Sigma||F_o| - |F_c||/\Sigma|F_o|$ for $F_o > 2\sigma(F_o)$; $wR2 = (\Sigma w(F_o^2 - F_c^2)^2/\Sigma(wF_c^2)^2)^{1/2}$ all reflections

$w=1/[\sigma^2(F_o^2)+(0.2000P)^2+150.0000P]$ where $P=(F_o^2+2F_c^2)/3$

Specific refinement details:

The crystals of **5**·16ClO$_4$·3MeCN·2H$_2$O [+ solvent] were grown by diffusion of diisopropyl ether into an acetonitrile solution of **5**·16NTf$_2$ containing excess (^{n}Bu$_4$N)·ClO$_4$. The crystals employed immediately lost solvent after removal from the mother liquor and rapid handling prior to flash cooling in liquid nitrogen was required to collect data. Despite these measures and the use of synchrotron radiation few reflections at greater than 1.0 Å resolution were observed. Nevertheless, the quality of the data is far more than sufficient to establish the connectivity of the structure. The asymmetric unit was found to contain one sixth of a Zn_8L_6 assembly (i.e. one complete organic ligand) and associated counterions and solvent molecules.

Several sections of the central core of the ligand were modelled as disordered over two locations with only the major occupancy parts refined anisotropically. In order to obtain a reasonable model for the organic parts of the structure the GRADE program[15] was thus employed, using the GRADE Web Server,[16] to generate a full set of bond distance and angle restraints (DFIX, DANG, FLAT). Due to the

thermal motion and less than ideal resolution, thermal parameter restraints (SIMU, RIGU) were applied to all atoms except for zinc to facilitate anisotropic refinement.

The anions within the structure also show evidence of disorder. One perchlorate anion was modelled as disordered over two locations and the occupancies of all located anions were freely refined.

Further reflecting the solvent loss and poor diffraction properties there is a significant amount of void volume in the lattice containing smeared electron density from disordered solvent. Consequently the SQUEEZE[17] function of PLATON[18] was employed to remove the contribution of the electron density associated with the highly disordered solvent, which gave a potential solvent accessible void of 26094 $Å^3$ per unit cell (a total of approximately 6650 electrons). Diffuse solvent molecules could not be assigned to acetonitrile or diisopropyl ether and were therefore not included in the formula. Consequently, the molecular weight and density given above are underestimated.

CheckCIF gives one A and four B level alerts. These alerts (both A and B level) result from the limited resolution, poor diffraction properties (high wR2) and thermal motion and/or unresolved disorder of the anions (large average Ueq values).

6·16OTf·4MeCN [+ solvent]

Formula $C_{348}H_{240}F_{48}N_{52}O_{48}S_{16}Zn_8$, *M* 7865.83, Monoclinic, space group P 21/c (#14), *a* 25.9759(4), *b* 48.2777(6), *c* 36.8773(4) Å, β 110.3310(10), *V* 43365.1(10) $Å^3$, D_c 1.205 g cm^{-3}, *Z* 4, crystal size 0.050 by 0.040 by 0.040 mm, color pale yellow, habit block, temperature 100(2) Kelvin, λ(synchrotron) 0.6889 Å, μ(synchrotron) 0.546 mm^{-1}, T(Analytical)$_{min,max}$ 0.9908809809073816, 1.0, $2\theta_{max}$ 36.50, *hkl* range -23 23, -43 43, -33 33, *N* 109452, N_{ind} 33662(R_{merge} 0.0595), N_{obs} 10495(I > 2σ(I)), N_{var} 4230, residuals* $R1(F)$ 0.1208, $wR2(F^2)$ 0.3444, GoF(all) 0.999, $\Delta\rho_{min,max}$ -0.449, 0.902 e^- $Å^{-3}$.

*$R1 = \Sigma||F_o| - |F_c||/\Sigma|F_o|$ for $F_o > 2\sigma(F_o)$; $wR2 = (\Sigma w(F_o^2 - F_c^2)^2/\Sigma(wF_c^2)^2)^{1/2}$ all reflections

$w=1/[\sigma^2(F_o^2)+(0.1700P)^2]$ where $P=(F_o^2+2F_c^2)/3$

Specific refinement details:

The crystals of **6**·16OTf·4MeCN [+ solvent] were grown by diffusion of ethyl acetate into an acetonitrile solution of **6**·$16NTf_2$ containing excess ($^nBu_4N$)·OTf. The crystals employed immediately lost solvent after removal from the mother liquor and rapid handling prior to flash cooling in liquid nitrogen was required to collect data. Despite these measures and the use of synchrotron radiation few reflections at greater than 1.1 Å resolution were observed and the data were trimmed accordingly. Furthermore, there was a significant drop-off in diffraction intensity after around 1.5 Å resolution resulting in a low ratio of observed/unique reflections. Nevertheless, the quality of the data is far more than sufficient to establish the connectivity of the structure. The asymmetric unit was found to contain one complete Zn_8L_6

assembly and associated counterions and solvent molecules. Due to the limited resolution, bond lengths and angles within pairs of chemically identical organic ligands were restrained to be similar to each other. Additional DFIX and DANG restraints were applied to some pyridyl rings displaying a high degree of thermal motion and one phenyl ring was modelled as disordered over two locations. Thermal parameter restraints (SIMU, RIGU) were applied to all atoms except for zinc. However, even with these restraints, some thermal parameters remain higher than ideal.

The anions within the structure show evidence of substantial disorder. Five of the located triflate anions were modelled as disordered over two or three locations. Substantial bond length and thermal parameter restraints were applied to facilitate stable refinement of the disordered anions and most low occupancy anions were modelled with isotropic thermal parameters. The occupancies of all located anions were allowed to freely refine which resulted in a discrepancy of 7.15 anions per Zn_8L_6 assembly. Most of the remaining electron density peaks (following the use of SQUEEZE, see below) are close to the triflate anions suggesting further disorder which could not be resolved due to the limited resolution of the data. The hydrogen atoms of one acetonitrile molecule could not be located in the electron density map and were therefore not included in the model.

Further reflecting the solvent loss and poor diffraction properties there is a significant amount of void volume in the lattice containing smeared electron density from disordered solvent and the remaining anions. Consequently the SQUEEZE[17] function of PLATON[18] was employed to remove the contribution of the electron density associated with these remaining anions and further highly disordered solvent, which gave a potential solvent accessible void of 12108 $Å^3$ per unit cell (a total of approximately 3054 electrons). Diffuse solvent molecules could not be assigned to acetonitrile or ethyl acetate and were therefore not included in the formula. Consequently, the molecular weight and density given above are underestimated.

CheckCIF gives one A and five B level alerts. These alerts mostly result from the limited resolution of the data with one alert also resulting from a short contact between pyridyl rings with a high degree of thermal motion.

5 $Zn^{II}\cdots Zn^{II}$ distance and $Zn^{II}\cdots Zn^{II}\cdots Zn^{II}$ angle measurements

Table S1. $Zn^{II}\cdots Zn^{II}$ distances for each edge type in pseudo-cubes **1**–**6** (edge types are shown in manuscript Figures 2–5).

Cage	Edge type	$Zn^{II}\cdots Zn^{II}$ distance (Å)
**1**	I	12.6 ± 0.2
**2**	I	13.7 ± 0.2
**3**	I	15.6 ± 0.1
**4**	I	14.2 ± 0.2
**5**	I	16.0
	II	15.5
**6**	I	12.3 ± 0.1
	II	10.4 ± 0.1
	III	13.4 ± 0.1

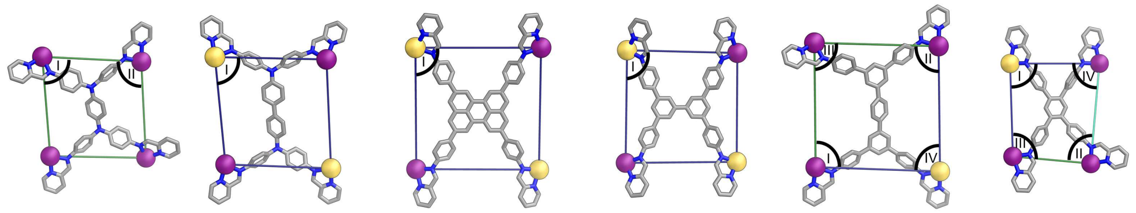

1 2 3 4 5 6

Figure S81. Different corner types in pseudo-cubes **1**–**6**.

Table S2. $Zn^{II}\cdots Zn^{II}\cdots Zn^{II}$ angles for each corner type in pseudo-cubes **1**–**6**.

Cage	Corner type	$Zn^{II}\cdots Zn^{II}\cdots Zn^{II}$ angle (°)
**1**	I	85.1 ± 1.4
	II	94.6 ± 1.6
**2**	I	90.0 ± 6.6*
**3**	I	90.0 ± 0.8
**4**	I	90.0 ± 1.1
**5**	I	93.2
	II	93.2
	III	88.4
	IV	85.2
**6**	I	87.1 ± 1.1
	II	89.6 ± 0.9
	III	98.9 ± 1.1
	IV	84.4 ± 1.1

*There is a large standard deviation as the structure of **2** is distorted in the solid state. There are essentially two different corner types (Figure S81), one with an obtuse (96.5 ± 1.9°) angle and one with an acute (83.5°) angle. The ^{1}H (and ^{13}C) NMR spectrum indicates the presence of only one, as opposed to two, magnetically distinct ligand arms. In solution, we hypothesize there to be dynamic changes in $Zn^{II}\cdots Zn^{II}\cdots Zn^{II}$ angle on a timescale faster than the NMR timescale.

6 Volume calculations

The volumes of the interior cavities of Zn_8L_6 pseudo-cubic metal-organic cages **1**–**6** were calculated from the crystal structure of each architecture using MoloVol[19]. The “probe-occupied volume” (V_{occ}) was calculated in each case in “single-probe mode”, using the following parameters:

Small probe radius: 2.0 Å, except in the cases of **1** and **5**, probe radii of 2.2 Å and 2.5 Å were used, respectively.

Grid resolution: 0.1 Å

Optimisation depth: 4 (default value)

Element radii. Zn : 2.39 Å, N : 1.66 Å, H : 1.20 Å, C : 1.77 Å

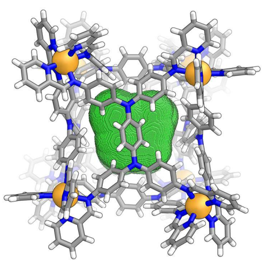

400 Å^3

1

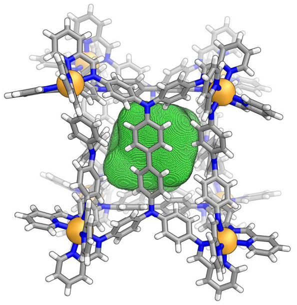

424 Å^3

2

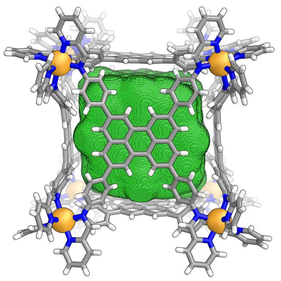

1770 Å^3

3

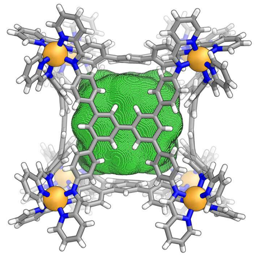

942 Å^3

4

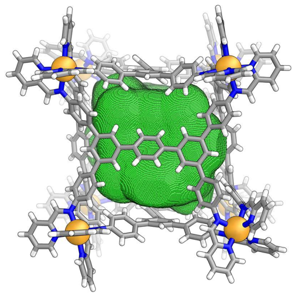

1600 Å^3

5

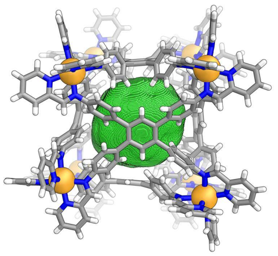

421 Å^3

6

Figure S82. MoloVol[19] calculated void space (green mesh) within the crystal structures of **1**–**6**. The MoloVol[19] volume of the interior cavity of **4** (942 Å^3) is similar to the previously reported value, 852 Å^3,[5] calculated using VOIDOO[20]. The small variation is attributed to the different probe sizes used.

The probe-occupied volumes of the interior cavities of previously reported $Co^{II}_4L_4$[21] and $Zn^{II}_4L_4$[22] tetrahedra were calculated using MoloVol[19] in single-probe mode.

Small probe radius: 1.2 Å (a smaller probe size is suitable in these cases due to the small and highly enclosed cavities of these two structures)

Grid resolution: 0.1 Å

Optimisation depth: 4 (default value)

Element radii. Zn : 2.39 Å, Co : 2.40 Å, N : 1.66 Å, H : 1.20 Å, C : 1.77 Å

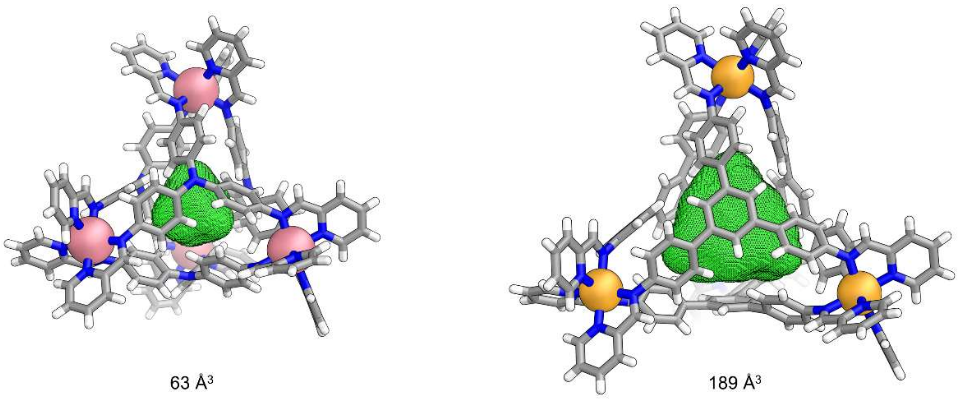

Figure S83. MoloVol[19] calculated void space (green mesh) within the crystal structures of previously reported $Co^{II}_4L_4$[21] and $Zn^{II}_4L_4$[22] tetrahedra. The volumes calculated using MoloVol[19] closely match the previously reported volumes calculated using VOIDOO[20]. The mean $Co^{II}\cdots Co^{II}$ and $Zn^{II}\cdots Zn^{II}$ separations in each structure are 12.0 ± 0.1 Å and 14.6 Å, respectively. These values are similar to the mean $Zn^{II}\cdots Zn^{II}$ distances in **1** (12.6 ± 0.2 Å) and **4** (14.2 ± 0.2 Å), respectively.

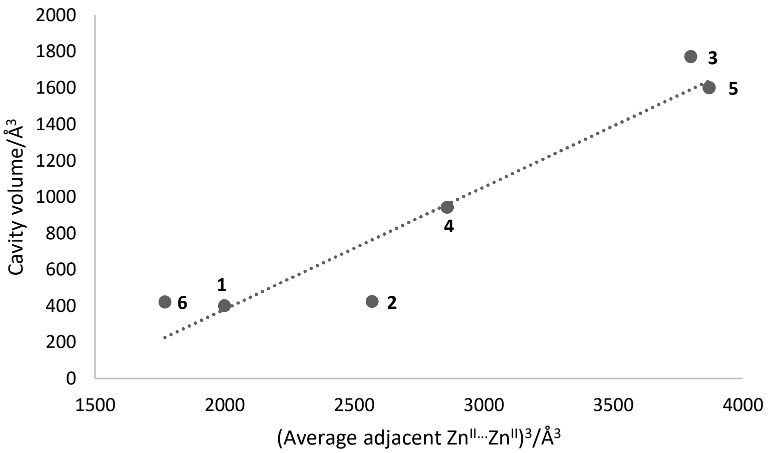

Figure S84. Cavity volume calculated using MoloVol[19] as a function of the cube of the average $Zn^{II}\cdots Zn^{II}$ distance between adjacent pseudo-cube vertices. We hypothesize that the lower than expected calculated cavity volume of pseudo-cube **2** is due to the orientation of the phenyl rings in the crystal structure; some phenyl rings of the **B** residue protrude into the cavity of **2**. Using a linear least-squares fit, cavity volume = $-964 + 0.672(Zn^{II}\cdots Zn^{II})^3$ with $R^2 = 0.90$.

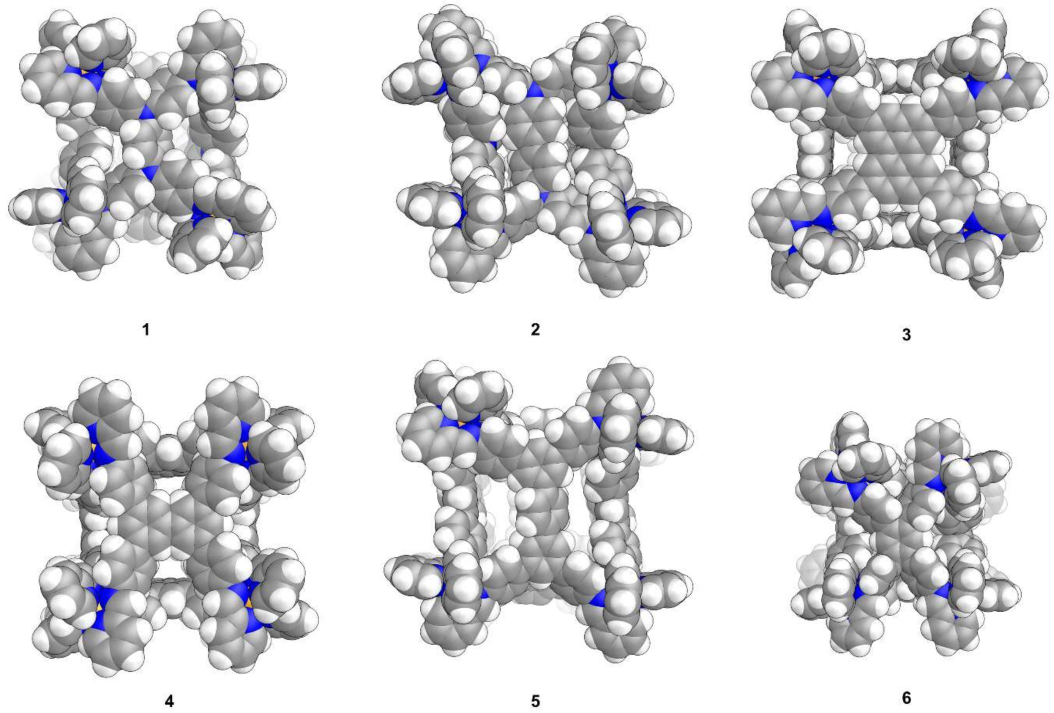

Figure S85. Crystal structures of **1**–**6**, with the cages in space-filling mode, illustrating the highly enclosed interior cavities created by the face-capping tetra-aniline residues.

7 Software versions used for computational studies

- GULP: 5.1[23,24]
- xTB[25]: 6.4.0=hf06ca72_0 on conda
- CREST[26]: version 2.9
- *stk*[27]: 2022.1.26.0
- *stko*[28]: 0.0.33
- SHAPE[29]: 2.1
- Python code for generating and analyzing cages and faces is available at: https://github.com/andrewtarzia/sca_cage_assembler

8 Diastereomers to be evaluated geometrically using computed models

In the manuscript, we describe four experimentally observed diastereomeric configurations, with T, T_h, S_6 and D_3 point symmetry. When discussing computational models here, we will use the labels $T1$, T_h2, S_62 and D_32, respectively, to specify these diastereomeric configurations. The number (i.e. 2 in T_h2) distinguishes the configuration from other diastereomeric configurations with the same point symmetry (see below). Although considering all possible pseudo-cube diastereomers that could potentially form was not reasonable, as well as the four diastereomers we observed experimentally in this work, denoted as $T1$, T_h2, S_62 and D_32 in Figure S86, we considered some additional diastereomers.

There is a second set of three diastereomers with T_h, S_6 or D_3 point symmetry, differing from T_h2, $S_6$2 and $D_3$2, which are here denoted as T_h1, $S_6$1 and $D_3$1, respectively. The difference stems from how the ligand arms wrap around the metal centers, particularly along edges formed by a pair of Zn^{II} centers with opposite handedness (Figure S86). In the observed diastereomers T_h2, $S_6$2 and $D_3$2, at edges formed by two Zn^{II} centers with opposing handedness where mismatching tetra-aniline residue axes meet, the pyridyl-imine moieties flip so that along the short tetra-aniline residue axis the imine bonds lie between the two Zn^{II} centers. The imine bonds thus lie on the outside of, or approximately in line with, the $Zn^{II}\cdots Zn^{II}$ axis along the long rectangular axis of the tetra-aniline residue. In the isomers T_h1, $S_6$1 and $D_3$1, at analogous edges to those discussed above, the opposite is observed; pyridyl-imine units flip so that imine bonds are placed in between the Zn^{II} centers along the long rectangular axis. The different relative placement of the imine bonds along the two different rectangular axes at edges formed by pairs of Zn^{II} centers with opposite handedness is illustrated in the facial configurations in Figure S86 (*ii* vs. *iv* and *iii* vs. *v*). We included diastereomers T_h1, $S_6$1 and $D_3$1 in our study to investigate the observed preference for T_h2, $S_6$2 and $D_3$2 diastereomeric configurations over T_h1, $S_6$1 and $D_3$1 (i.e., a preference for facial configurations *ii* and *iii* over *iv* and *v*, respectively).

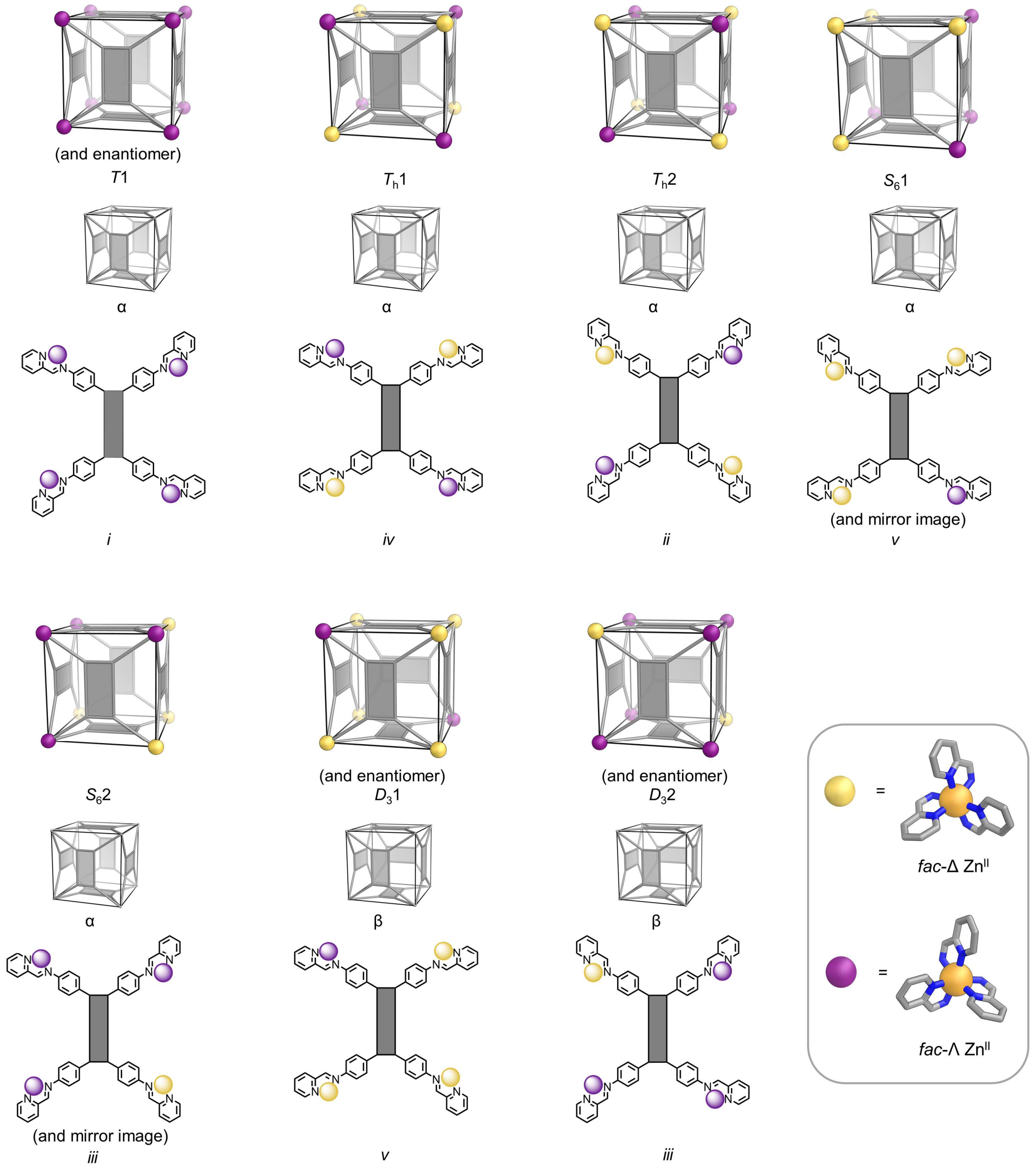

Figure S86. Schematic representations of the four diastereomers observed experimentally in this work (denoted as $T1$, T_h2, S_62 and D_32), along with diastereomers not observed experimentally in this work, but with the same idealized point symmetry as those that are (T_h1, S_61 and D_31). The difference between T_h2, S_62 and D_32 compared to T_h1, S_61 and D_31 is the relative position of the imine bonds along rectangular axes meeting at edges formed by Zn^{II} centers with opposing handedness. As in the manuscript (Figures 2−5), we illustrate the stereochemical elements of each isomer using the relative orientation of rectangular panels, and handedness of the Zn^{II} centers (highlighted using facial configurations).

In addition to the seven diastereomers in Figure S86, we considered another seven diastereomers (Figure S87) during the development of the strategy for the prediction of the preferred M_8L_6 pseudo-cube diastereomer for a given tetra-aniline subcomponent. The following were included from previously

reported crystal structures: $D_2$1,[30] $S_4$1,[31] $D_3$2n,[32] and C_{2h}1.[33] $S_4$2 and $D_3$1n were included for analogous reasons to T_h1, $S_6$1 and $D_3$1. C_{2v}1 was constructed during the attempted construction of another diastereomer and we included it as it seemed a plausible diastereomer and had the added feature that it was not included based on rationale; we were interested to see how C_{2v}1 performed against the diastereomers that were observed experimentally or rationally included.

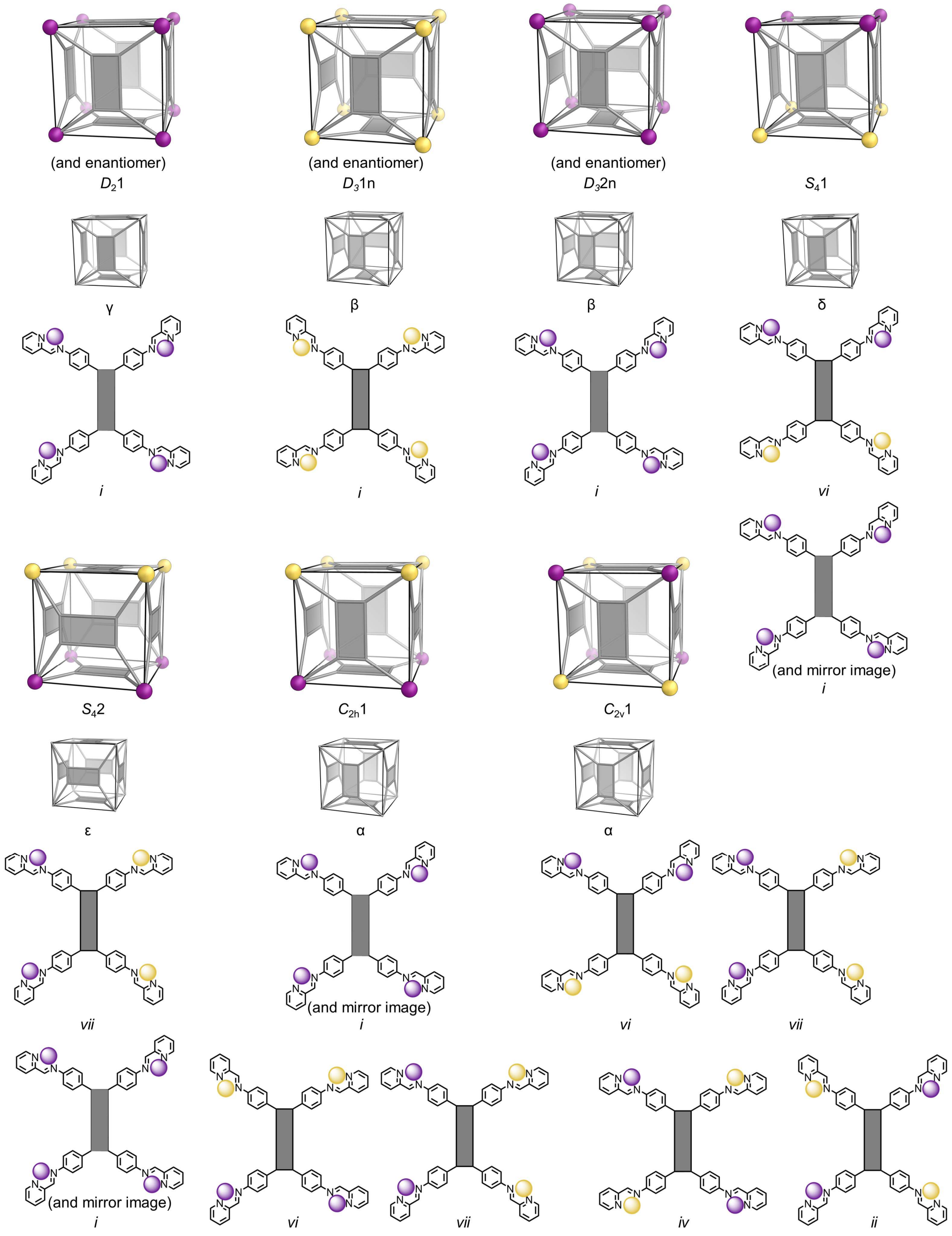

Figure S87. Schematic representations of the seven additional diastereomers to be included in the development of a method for the prediction of the preferred Zn_8L_6 pseudo-cube diastereomers for given tetra-aniline subcomponents.

9 Tetratopic panel model construction

In order to construct face (Supporting Information Section 11.1) and cage (Supporting Information Section 15.1) models, tetratopic organic subcomponent (see below) and metal complex (Supporting Information Section 10) building blocks were used. The tetratopic building blocks have bromine atoms at the points that connect to the metal complexes, these act as connection points in the construction algorithm, and are not retained in the final face and cage models. For each SMILES string in Table S3, we generated a conformer ensemble of 100 conformers using the ETKDG[34,35] algorithm in RDKit[36]. The most planar conformer was saved for cage and face model construction. The planarity of each conformer was calculated as the sum of the atom deviations (shortest distance to the plane) from the plane of best fit through the conformer. The long axis of the rectangular panel was defined based on the position of the bromine atoms in each molecule. Figure S88 shows the assigned long axis of each tetratopic building block.

Table S3. SMILES strings for the organic building blocks. The labels **A**–**F** are the names of tetra-aniline building blocks used in this work that the given “tetrabromo panel” is associated with. Bromine functional groups are used as the connection points for the tetratopic panels, which are tetra-anilines in the experimental parts of the work. The “Cap” building block is the bidentate binding motif used to construct the metal complex building blocks in Figure S89.

	SMILES
**Cap**	Br/N=C/c1ccccn1
**A**	Brc1ccc(N(c2ccc(Br)cc2)c2ccc(N(c3ccc(Br)cc3)c3ccc(Br)cc3)cc2)cc1
**B**	Brc1ccc(N(c2ccc(Br)cc2)c2ccc(-c3ccc(N(c4ccc(Br)cc4)c4ccc(Br)cc4)cc3)cc2)cc1
**C**	Brc1ccc(-c2cc3cc(-c4ccc(Br)cc4)cc4c5cc(-c6ccc(Br)cc6)cc6cc(-c7ccc(Br)cc7)cc(c(c2)c34)c65)cc1
**D**	Brc1ccc(-c2cc(-c3ccc(Br)cc3)cc(-c3cc(-c4ccc(Br)cc4)cc(-c4ccc(Br)cc4)c3)c2)cc1
**E**	Brc1ccc(-c2cc(-c3ccc(Br)cc3)cc(-c3ccc(-c4cc(-c5ccc(Br)cc5)cc(-c5ccc(Br)cc5)c4)cc3)c2)cc1
**F**	Brc1ccc(-c2cc(-c3ccc(Br)cc3)c(-c3ccc(Br)cc3)cc2-c2ccc(Br)cc2)cc1

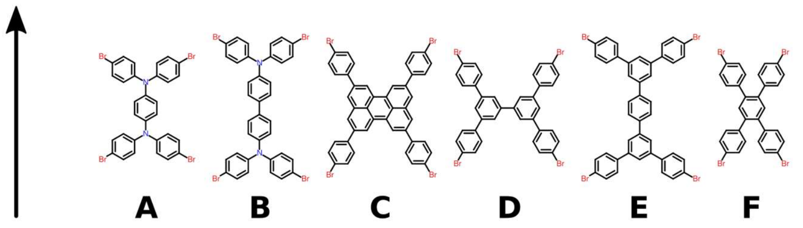

Figure S88. Tetratopic subcomponent panel building blocks used to construct face and cage models (names of associated tetra-anilines on bottom), with the long axis aligned vertically. Bromine atoms (colored red) reside at connection points, which connect to metal complex building blocks in face and cage models.

10 Metal complex model construction

The two Zn^{II} complexes used in this work were constructed with *stk*[27] and used for all face and cage model constructions. The *tris*-chelated Zn^{II} centers with Δ and Λ handedness were constructed from different topology graphs. Each complex was geometry optimized using UFF4MOF[37-39] in GULP[23,24] (the square planar atom type, "Zn4+2", was used for Zn, while other atom types were derived in *stko;* available online at https://github.com/JelfsMaterialsGroup/stko) followed by geometry optimization with GFN2-xTB[25,40] with a charge of +2. Because there is no octahedral Zn parameter in UFF4MOF, we have applied the square planar parameter as an approximate solution for intermediate geometry optimizations, as done by Addicoat co-workers.[38] We expect these two atom types to be mostly equivalent, preferring 90° angles between neighboring substituents. Figure S89 shows the optimization results, and the average $Zn^{II}\cdots N$ distance, for the metal complexes at the UFF and GFN2-xTB level, confirming that this approximation was sufficient.

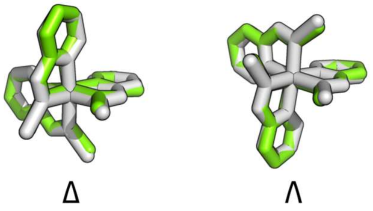

Avg. Zn-N distances [Å]

	Δ	Λ
UFF opt.:	2.14	2.14
xtb opt.:	2.13	2.13

Figure S89. Overlay of UFF (white) and GFN2-xTB (green) structures of the Δ and Λ *tris*-chelated Zn^{II} complexes. Average $Zn\cdots N$ distance at the two levels of optimization are shown underneath.

11 Ligand-based geometric approach for assessing relative stability of diastereomers

Experimental observations indicated that the difference in the distance between imine-condensed aniline N atoms along two tetra-aniline residue sides meeting at a pseudo-cube edge influenced whether that edge was formed by a pair of Zn^{II} centers with the same or opposite handedness (Figure 6 in the main text). We proposed that a deviation from the formation of edge types formed by Zn^{II} centers with the same handedness is driven by the inversion of the stereochemistry of one Zn^{II} center resulting in a smaller difference (or better match) between the ideal $Zn^{II}\cdots Zn^{II}$ distances (when not constrained in a pseudo-cube) along the two ligand lengths meeting at the pseudo-cube edge. In our pursuit to develop a strategy to predict the preferred Zn_8L_6 pseudo-cube diastereomer formed by a given tetra-aniline, we explored the viability of an approach which focused on comparing ideal $Zn^{II}\cdots Zn^{II}$ distances along different ligand lengths. To obtain estimates for these $Zn^{II}\cdots Zn^{II}$ distances along ligand lengths before being constrained in a pseudo-cube, we built models of facial configurations, denoted *i-vii,* for each of tetra-anilines **A**–**F**. For each tetra-aniline **A–F**, we construct a new *stk* "**Face**" topology graph that

connects one tetratopic subcomponent panel to four metal complexes. The complexes have either Δ or Λ handedness, the distribution of which defines the facial configuration.

11.1 Face model construction

The face model simplifies the computational method for assessing the relative (geometric) stability of diastereomers significantly. However, to adequately represent the planarity of tetra-anilines in the cage structures is difficult. In most of the crystal structures for pseudo-cubes **1**–**6**, we find nearly planar conformations for tetra-aniline residues. By modelling just the face, we ignore the restriction and strain on the ligand from connections to other neighboring faces, which could result in preferred geometries for the tetra-aniline residues that deviate from (near) planar. To avoid analyzing lower-in-energy conformers of tetra-aniline residues that do not match the planar form expected within the pseudo-cube, we performed a restricted geometry optimization of the face structures. In this process, we must balance representing the tetra-aniline residue conformation inside a cage with adequately geometry optimizing an isolated face structure. The predictions from the face model will depend on the features of the faces (bonds, angles, etc.) being reasonably representative of the conformation that would be observed in the putative pseudo-cube diastereomer. Here, the goal is to have a cheap representation of the strain in different pseudo-cube diastereomers arising from the rectangular nature of the subcomponents (i.e. ligand lengths with different ideal $Zn^{II}\cdots Zn^{II}$ separations meet at pseudo-cubes edges formed by two Zn^{II} centers), which may capture the qualitative trends that govern diastereomer preference. The optimization process is:

1. After *stk* construction, we performed an **MCHammer** geometry optimization (a rigid-body optimization of the building-block-to-building-block distance in an *stk* molecule).
2. A limited geometry optimization (50 cycles only) with UFF4MOF in GULP ("Zn4+2" force field type used for Zn, other atom types derived in *stko*) is performed. Structures from this point were saved for comparison.
3. A longer geometry optimization with UFF4MOF is performed until convergence is met (or 2000 steps are completed).
4. A geometry optimization with GFN2-xTB to the "crude" convergence criteria (energy converged within 5 x 10^{-4} Hartrees and gradient norm converged within 1 x 10^{-2} Hartree bohr^{-1}; hence, it remains somewhat restricted) is performed.

Comparing the geometries from steps 2 and 4 above, we found that most structures remained planar (Figure S90b shows slight deviation in face *ii* for tetra-aniline **D**). The most common change we see is in the angle between the center of the face and the metal center (pink lines in Figure S90a). Clearly, these distortions stem from differences in the potential energy surfaces of different methods. Throughout this analysis, we must also acknowledge how differing degrees of flexibility of the ligands will affect the representativeness of this analysis. For example, such a restrained optimization process on a very flexible ligand may be less meaningful than for a rigid ligand.

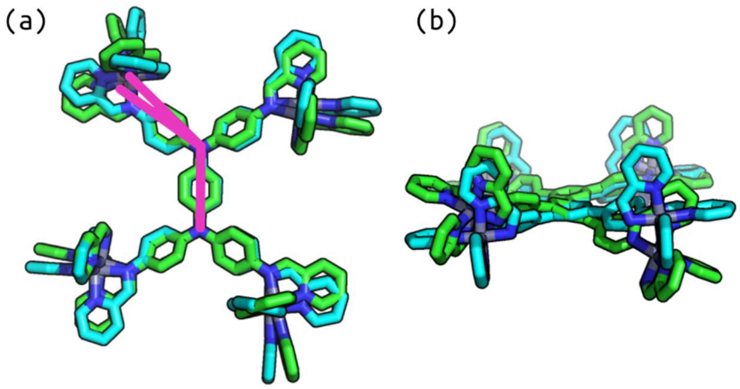

Figure S90. Comparison of face structures at steps 2 (blue) and 4 (green) of the optimization protocol. a) Tetra-aniline **A**, face configuration *i*. b) Tetra-aniline **D**, face configuration *ii* shows the slight loss of planarity following the additional optimization steps.

From here on, we focus on results from step 4 of the optimization process. After step 2, a very restricted optimization sequence, we obtain similar general trends for the following analysis.

11.2 Geometric feasibility of edge types

Upon inspection of the computationally constructed face models, it was apparent that in some cases there was a large disparity between the conformation of the tetra-aniline core in the face models compared to those in the corresponding crystal structure, illustrated in Figure S91. The cores of tetra-aniline **E** residues in **5** exhibit a noticeable bend (Figure S91a), which we propose to be a key feature driving the preference for the observed ($S_6$2) diastereomer. However, this bend is not replicated in the model of facial configuration *iii*. We inferred that the incorporation of the bend in **E** residues in pseudo-cube **5** reduced the strain incurred upon formation of pseudo-cube edges. It is thus intuitive that such a bend does not occur in the isolated face models due to the absence of pseudo-cube edge formation in this approximation. Instead of being perfectly planar, tetra-aniline **D** has a bowed shape in the crystal structure of pseudo-cube **4**, potentially to relieve strain associated with eclipsing the four central hydrogen atoms of the biphenyl unit. We predict this bowing of **D** residues acts to increase the difference in N···N separations (Δ(N···N)) along tetra-aniline sides meeting at the pseudo-cube edges, which results in a preference for pseudo-cube edges formed by pairs of Zn^{II} centers with opposing handedness. The absence of the bowing, and the deviation from planarity, of the **D** residues in the model would likely result in the inaccurate prediction of the preferred diastereomeric configuration of pseudo-cube **4** by a geometric approach that uses isolated face models only.

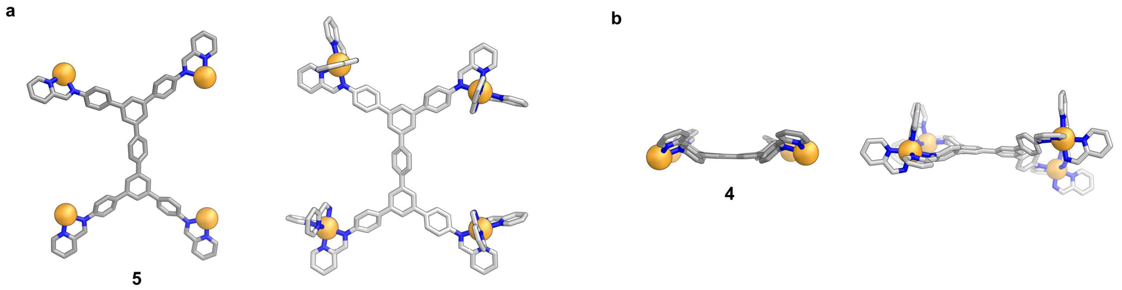

Figure S91. Disparity between the ligand conformations in the experimental crystal structures and face models. a) Face extracted from the crystal structure of **5** (left) and the model of facial configuration *iii* for **E** (right). b) Face extracted from the crystal structure of **4** (left) and the model of facial configuration *ii* for **D** (right).

Furthermore, the prediction of the preferred diastereomer using an approach based on the facial configuration models would require the preselection of the relative arrangement of rectangular ligand panels (α, β… *etc*) for a given subcomponent. Experimentally, high symmetry orientational configuration α is observed in the overwhelming majority of cases. Despite being able to post rationalize the only deviation from α (pseudo-cube **6**), it is challenging to predict this from the structure of the subcomponent only.

Therefore, we concluded that we were unlikely to be able to develop a strategy based on the facial configuration models for the prediction of the exact pseudo-cube diastereomer favored for each of tetra-anilines **A**–**F**. However, we recognized the potential for these face models to provide a platform for assessing the geometric feasibility of the fourteen diastereomers in more general terms. This was done by considering the edge types present in each diastereomer. The $Zn^{II}\cdots Zn^{II}$ separations from the face models were used to evaluate the geometric feasibility of forming each edge type. We hypothesized that by determining which edge types appear more or less favored may in turn help to identify which diastereomers may be more or less favored.

In the crystal structures of pseudo-cubes **1**–**6**, there are four different edge types (Figure S92), three formed by pairs of Zn^{II} centers with the same handedness (**E1**–**E3**), and one formed by two Zn^{II} centers with opposite handedness (**E4**). It is worth noting that **E1**–**E3** were encompassed by the description of edge types formed by "Zn^{II} centers with the same handedness" in Figure 6b in the main manuscript, while **E4** is equivalent to the edge type described as being formed by "Zn^{II} centers with opposing handedness" in the same Figure. Note that the nomenclature Edge type I, II, III in the main text (Figures 2–6) is used to simply distinguish between the different edge types within a given pseudo-cube, and does not specifically correspond to a particular edge type described using the nomenclature **E1**, **E2**…, which we here use to describe possible edge types in general terms. For example, in pseudo-cubes **2**–**4**, the edges labelled edge type I (Figures 3 and 6) are of the type **E4** (Figure S92), not **E1**.

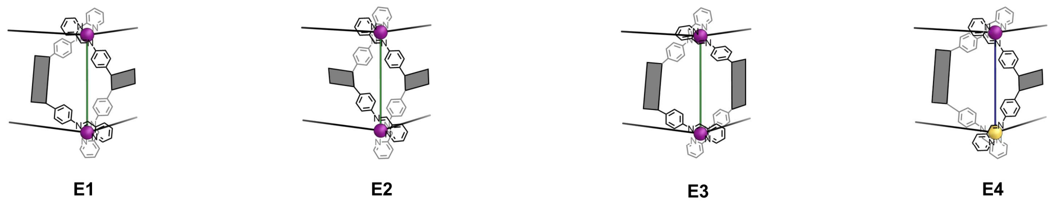

Figure S92. Edge types present in the experimentally observed diastereomers in this work ($T1$, T_h2, S_62 and D_32). The mirror images (all-Δ analogues) of **E1**–**E3** displayed are also present.

In some of the diastereomers not observed experimentally in this work, three edge types in addition to **E1**–**E4** are present (Figure S93). In **E4**, the short-axis nitrogen atoms are placed between the two Zn^{II} centers, and the long-axis nitrogen atoms are on the outside of, or approximately in line with, the $Zn^{II}\cdots Zn^{II}$ axis. In contrast, in **E5**, the long-axis nitrogen atoms are placed between the two Zn^{II} centers, and the short-axis nitrogen atoms would be placed at the wider positions. The final two edge types are also spanned by a pair of Zn^{II} centers with opposite handedness, with either two long (**E6**) or two short (**E7**) tetra-aniline residue axes meeting at the edge.

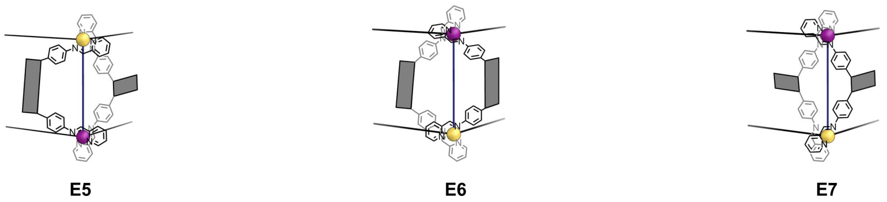

Figure S93. Edge types (**E5**–**E7**) that are, in addition to **E1**–**E4**, present in the diastereomers not observed experimentally in this work.

In **E2** and **E3**, like tetra-aniline axes meet at the edge, therefore, it is intuitive for the two Zn^{II} centers to have the same handedness; these edge types are analogous to the edges found in most metal-organic polyhedra assembled from high symmetry capping ligands.[41,42] Consequently, we did not include these in the screening, and assigned these as geometrically viable edge types straight away.

We assessed the geometric feasibility of edge types **E1** and **E4**–**E7** by considering the average difference in the ideal $Zn^{II}\cdots Zn^{II}$ distances ($\Delta(Zn^{II}\cdots Zn^{II})$) along the different ligand lengths meeting at that edge type. The $Zn^{II}\cdots Zn^{II}$ distances were obtained from constructed and optimized face models, i.e., the face is restrained to be near planar but with the absence of additional strain (bending/flexing of the tetra-aniline residue) that often arises from being constrained within a pseudo-cubic framework. Figure S94 illustrates the concept of this strategy, indicating which facial configurations and specific ligands lengths are used for the evaluation of each edge type.

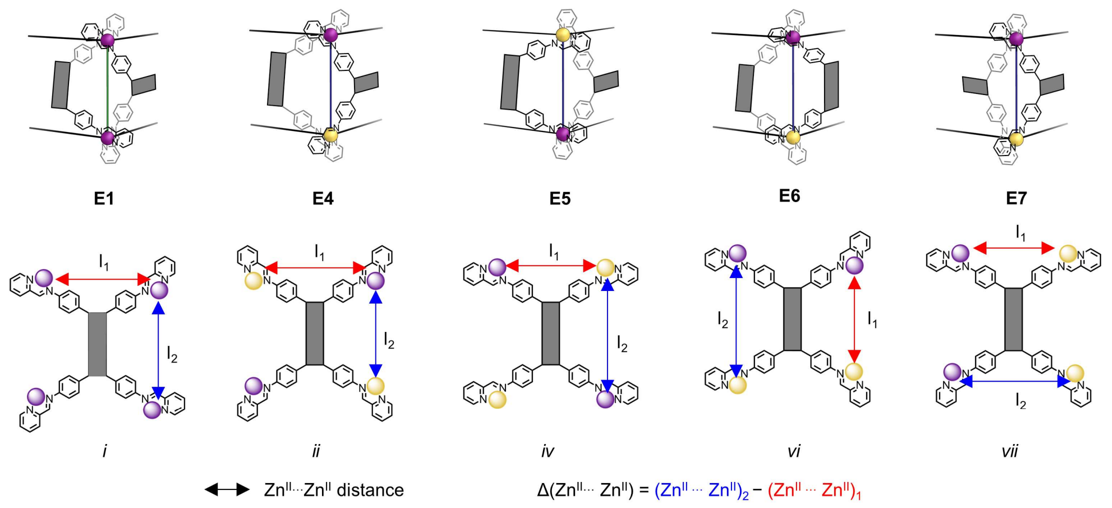

Figure S94. Pairs of $Zn^{II}\cdots Zn^{II}$ separations used to assess the geometric feasibility of particular edge types.

Figure S95a shows the calculated values for $\Delta(Zn^{II}\cdots Zn^{II})$ for the edge types **E1** and **E4**–**E7**, as a function of the average difference in N···N separations along the tetra-aniline sides meeting at the given edge, Δ(N···N). Values for average Δ(N···N) in each case were calculated using N···N separations measured from the same face models from which $Zn^{II}\cdots Zn^{II}$ distances were measured. For example, for assessment of edge type **E1**, both N···N and $Zn^{II}\cdots Zn^{II}$ separations used to calculate Δ(N···N) and $\Delta(Zn^{II}\cdots Zn^{II})$, respectively, were measured from the model for the facial configuration *i*. As alluded to previously, we opted to focus on a geometric parameter, in this case Δ(N···N), as opposed to the identity of the particular tetra-aniline, due to the inaccuracy of the tetra-aniline residue conformation in the face model at approximating the tetra-aniline residue conformation in the pseudo-cube. In the face models, the tetra-anilines approximate rigid rectangles with differing aspect ratios, providing a good platform for investigating the relationship between the $Zn^{II}\cdots Zn^{II}$ mismatch along ligand lengths meeting at the edge ($\Delta(Zn^{II}\cdots Zn^{II})$) and the average difference in N···N separations along the tetra-aniline sides meeting at that edge (Δ(N···N)), for the five different edge types considered. It should be noted that values for average Δ(N···N) calculated from the face models differ, in some cases considerably, from the mean Δ(N···N) calculated from N···N separations measured from the crystal structures of pseudo-cubes **1**–**6**. This difference in values of Δ(N···N) between the models and crystal structures arises from the already highlighted difference in the conformation of the subcomponent cores in the solid-state structures compared to in the models. Figure S95b shows the same data for **E1**, **E4** and **E5** as in Figure S95a but highlights that the values of Δ(N···N) change for particular tetra-aniline residues in different face models, which in theory should not occur in these models. From the models, for a given tetra-aniline, Δ(N···N) values would be expected to be the same for **E1**, **E4** and **E5** as the short axis of one residue meets the long axis of another residue at all three of these edge types. We suggest that this variation in values for Δ(N···N) arises from the restricted geometry optimization yielding slightly different conformations of the tetra-aniline residue cores in each case. This observation does not significantly impact this study, however. We target general trends for the relationship between $\Delta(Zn^{II}\cdots Zn^{II})$ and Δ(N···N) and thus

provided we use Δ(Zn^{II}···Zn^{II}) and Δ(N···N) values both measured from the same model, there will be very little effect upon the conclusions drawn.

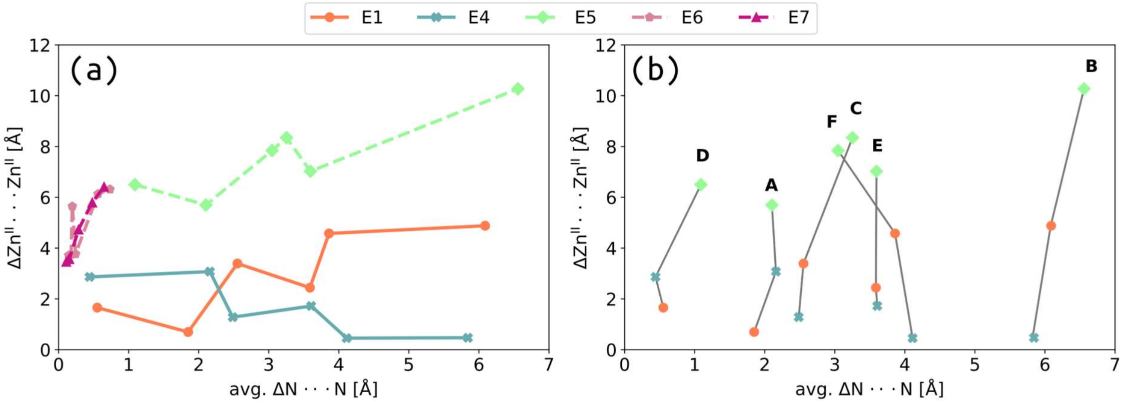

Figure S95. Mismatch in ideal Zn^{II}···Zn^{II} separations between ligand lengths meeting at a pseudo-cube edge as a function of the average difference in N···N distances along the tetra-aniline sides meeting at that edge, for the edge types **E1** and **E4**–**E7**. In a), lines connect mismatch values based on the same edge type, and **E1** and **E4** are in solid because they are observed experimentally. In b), vertical, grey lines connect mismatch values, for edge types at which the short rectangular axis of one tetra-aniline residue meets the long axis of another, based on the same subcomponent. **E6** and **E7** are not shown for clarity, tetra-aniline axes match at these edge types so the Δ(N···N) values for these edge types cannot be directly compared to those for **E1**, **E4** and **E5**.

If the strain associated with forming a pseudo-cube edge between two ligand units is minimized by minimizing Δ(Zn^{II}···Zn^{II}), we can predict the relative stability of the different edge types. The lowest value for Zn^{II}···Zn^{II} mismatch switches between **E1** and **E4** (discounting **E2** and **E3**), depending on the value of average Δ(N···N). These results are consistent with those determined from the experimental crystal structures (Figure 6, main manuscript). Importantly, this computational approach allowed us to assess the feasibility of edge types not observed experimentally. These edge types, **E5**–**E7**, can be deemed unlikely to form. As seen in Figure S95a, the mismatch in Zn^{II}···Zn^{II} separations for **E5** is always larger than for **E1** and **E4**. The mismatches for **E6** and **E7** are never approximately 0 Å, which would be required to be favored over **E3** and **E2**, respectively. The alignment of the rectangular axes of tetra-aniline residues at edge types **E2** and **E3**, coupled with the two Zn^{II} centers having the same handedness, means that values of Δ(Zn^{II}···Zn^{II}) ≈ 0 Å would be expected for each of **E2** and **E3**.

The value for Δ(N···N) at which there is a switch in preference from **E1** to **E4** matches very well with the empirically derived phase boundary of mean Δ(N···N) ≈ 2 Å (Figure 6, manuscript). However, the zigzag nature of the lines connecting the points in the plot in Figure S95a highlights the impact of the relative values for the angle between subcomponent arms (θ in Figure S96) along distinct rectangular axes of the tetra-aniline subcomponent upon the value of Δ(Zn^{II}···Zn^{II}) (and thus the relative geometric favorability of edge types). Although we recognized such a phenomenon would be present, clear evidence of this could not be observed from the experimental data, given the data points available.

Tetra-anilines **A**–**F** have an idealized angle between subcomponent arms of 60° along one rectangular axis, and an ideal angle of 120° between subcomponent arms which span the other distinct rectangular axis. Tetra-anilines **A**, **B**, and **E** have the 120° angle along the short axis and θ = 60° along the long axis, which is reversed for **C**, **D** and **F**.

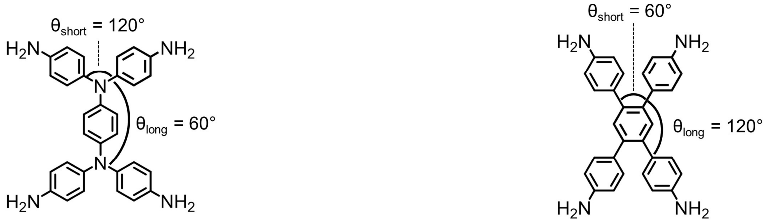

Figure S96. Idealized angles between subcomponent arms (θ) along the distinct rectangular axes of tetra-anilines **A** and **F**.

Figure S97 plots the same data for **E1** and **E4** as displayed in Figure S95a, but with the points grouped according to the relative angles between subcomponent arms along the distinct tetra-aniline axes. Such an analysis indicates that the value of Δ(N···N) at which there is a switch in the preference for **E4** vs. **E1** is dependent on the relative values of θ along the two tetra-aniline axes meeting at a pseudo-cube edge. Based on the point at which the lines for edge types **E1** and **E4** intersect, the "phase boundary" has a lower value for average Δ(N···N) when the angle between subcomponent arms is 60° along the short tetra-aniline axis and 120° along the long axis, compared to when this is reversed (Figure S97). When applying the new computationally determined boundaries (Figure S97) to the experimental data in this work, the experimental observations and the computational data remain complimentary (Figure S98).

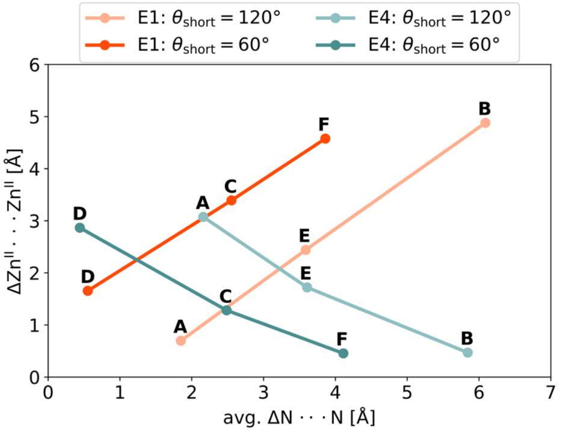

Figure S97. Mismatch in ideal $Zn^{II}\cdots Zn^{II}$ separations between ligand lengths meeting at a pseudo-cube edge as a function of the average difference in N···N distances along the sides of the tetra-anilines meeting at that edge, for the edge types **E1** and **E4,** grouped by the idealized value of θ_{short} in the tetra-aniline in each model. The name of the tetra-aniline used to form the face model, from which $Zn^{II}\cdots Zn^{II}$ and N···N distances are measured, is shown next to each point.

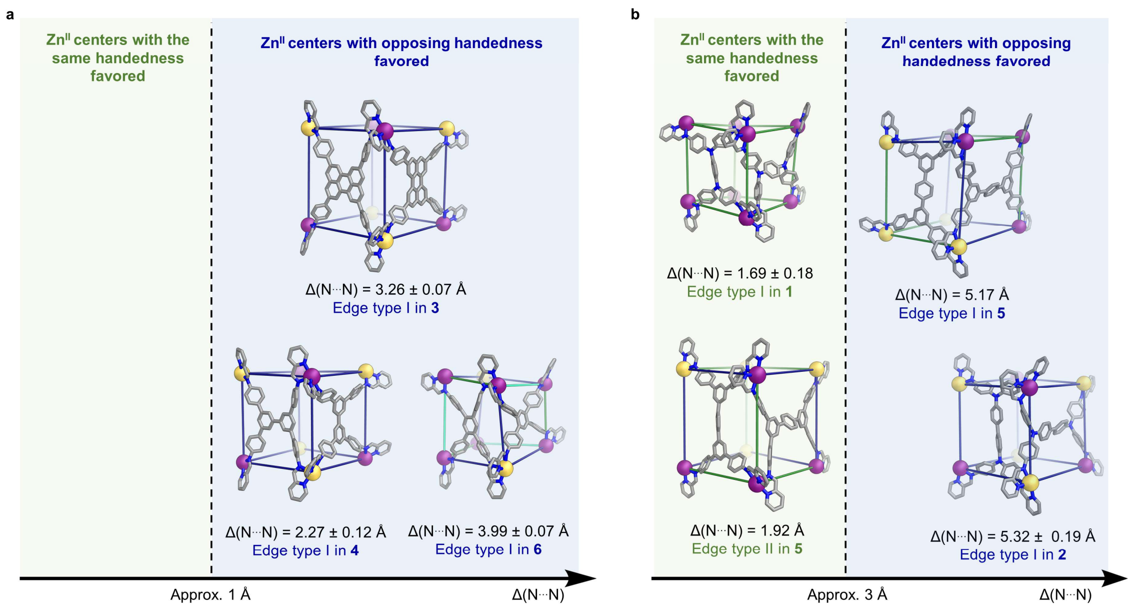

Figure S98. Data from an edge analysis conducted on the crystal structures of pseudo-cubes **1**–**6**, grouped according to the relative idealized angles between subcomponent arms (θ) along distinct tetra-aniline axes a) θ_{short} = 60°, θ_{long} = 120°. b) θ_{short} = 120°, θ_{long} = 60°. "Phase boundaries" are those determined from the analysis of computationally constructed and optimized face models (Figure S97). Edge types II and III in pseudo-cube **6** are omitted as they correspond to edge types **E2** and **E3**. This Figure focusses on the boundary for a preference for **E1** vs. **E4**.

Future work in which geometric rules are used to design both homoleptic and heteroleptic metal-organic architectures from multitopic bidentate paneling ligands should consider the angle between subcomponent arms (θ). Values of both θ and N···N separation along ligand lengths meeting at edges of a polyhedron described by pairs of metal centers impact upon the ideal M···M (where M = metal center) distances along those lengths and thus the value of Δ(M···M) (a more general analogue of Δ(Zn^{II}···Zn^{II})) which is used to assess the geometric feasibility of such edges. Similar considerations are already widely made for the assembly of metal-organic architectures from square planar metal centers and *bis*-monodentate ligands.[43,44]

Table S4 lists the fourteen diastereomers included in this study, along with the edge types present in each. From the above analysis we determined that **E1**–**E4** are feasible edge types. When like axes of the tetra-aniline residues meeting at an edge align, edge type **E2** or **E3** would be expected. When there is a mismatch of the axes of the tetra-aniline residues at the edge, of which there needs to at least be some to form these pseudo-cube structures, a preference for **E1** vs. **E4** depends on the difference in length along subcomponent sides meeting at the edge (quantified using Δ(N···N)). Furthermore, the values of the angle between aniline arms along the tetra-aniline sides meeting at the edge impacts upon the value of Δ(N···N) at which the preference for **E1** vs. **E4** switches. We infer that **E5**–**E7** are not favored for any pseudo-cube edges where two rectangular tetra-aniline residues meet. Making the assumption that pseudo-cube diastereomers containing disfavored edge types (**E5**–**E7**) are themselves disfavored

allows the prediction of which diastereomeric configurations are geometrically favored, and disfavored. The geometrically feasible diastereomers are predicted to be T1, T_h2, $S_6$2, $D_3$2, $D_2$1, $D_3$1n and $D_3$2n, while T_h1, $S_6$1, $D_3$1, $S_4$1, $S_4$2, C_{2h}1 and C_{2v}1 are not (Table S4).

Table S4. Diastereomers assessed for geometric feasibility, and the edge types they contain. Edge types, and thus diastereomers, determined to be disfavored are colored red.

Diastereomer	Edge types present
T1	**E1**
T_h1	**E5**
T_h2	**E4**
$S_6$1	**E1** + **E5**
$S_6$2	**E1** + **E4**
$D_3$1	**E2** + **E3** + **E5**
$D_3$2	**E2** + **E3** + **E4**
$D_2$1	**E1** + **E2** + **E3**
$D_3$1n	**E1** + **E2** + **E3**
$D_3$2n	**E1** + **E2** + **E3**
$S_4$1	**E1** + **E2** + **E6**
$S_4$2	**E1** + **E3** + **E7**
C_{2h}1	**E1** + **E4** + **E5**
C_{2v}1	**E1** + **E4** + **E5**

The diastereomers predicted to be geometrically feasible by this method align well with experimental observations. In this work, the experimentally prepared assemblies exist as the diastereomers T1, T_h2, $S_6$2 or $D_3$2. From the reaction of TPE-based tetra-anilines, 2-formylpyridine and $Zn(OTf)_2$ in a mixture of acetonitrile and dichloromethane, the Cui group obtained crystal structures that revealed Zn_8L_6 pseudo-cubes as $D_2$1 and $D_3$2 diastereomers (Figure S99).[30] Although we did not observe the $D_2$1 diastereomer in our work, our ligand-based edge type analysis above predicts this diastereomeric configuration to be geometrically feasible.

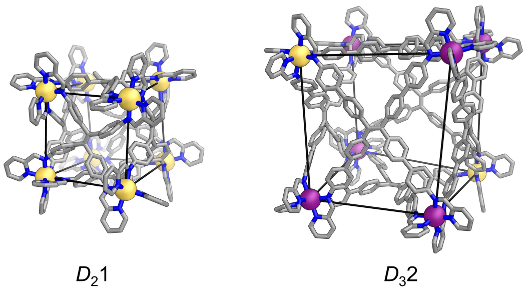

Figure S99. Crystal structures of Zn_8L_6 pseudo-cubes reported by Cui and co-workers. One is the $D_2$1 diastereomer and the other is $D_3$2 – both are "allowed" diastereomers according to our ligand-based geometric analysis of edge types **E1**–**E7**.[30]

M_8L_6 pseudo-cubes prepared by methods other than the subcomponent self-assembly of a rigid rectangular tetra-aniline subcomponent with 2-formylpyridine and Zn^{II} are not considered in our analysis at this stage.[31,32,45] However, it is envisaged that our methods might be extended to other metal cations, including Fe^{II} and Co^{II}. The differing propensity of these cations to distort from octahedral coordination geometries is likely to impact upon the self-assembly outcomes, which may be interestingly different from those observed for Zn^{II}.

12 Deconstruction of subcomponent self-assembly

For high-throughput structure generation using *stk*,[27] we have applied an alchemical disconnection of the ligands into "core" (organic tetratopic) and "coordinating" (metal complex) building blocks. We constructed cages using a hierarchical construction approach (Figure S100) from these components where the coordinating building blocks were placed on the Δ and Λ octahedral metal topologies, which are placed on the corners of the cage topology, and the core building blocks are placed on the faces of the cage topology.

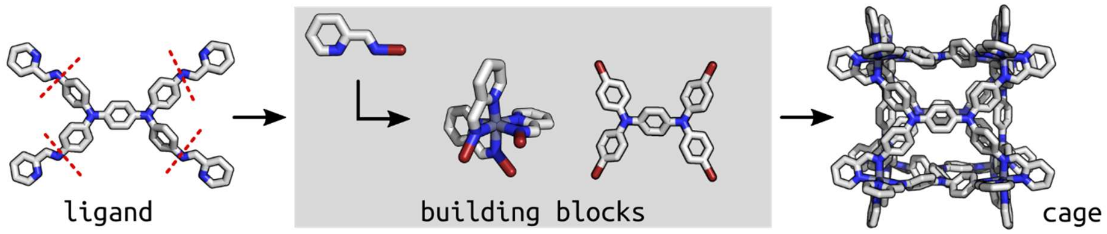

Figure S100. Deconstruction of the ligand formed by **A** into *stk* building blocks for cage construction. The Δ complex is shown, the Λ complex is also present in the cage constructed. Red dashed lines show the disconnection points.

13 Library definitions

To automate the computational process of cage construction, four library files are used: 1) tetratopic building block library (Table S3), 2) metal complex library, 3) cage library and 4) experimental library (Table S5). These are provided as JSON files with the source code. Only two complexes were used, Zn^{II} centers with an octahedral coordination geometry, *tris*-chelated by bidentate pyridyl-imine ligands, with either Δ or Λ handedness. In Supporting Information Section 8, we introduce the diastereomers studied in this work, which are defined with the available source code (in the file "symmetries.py"). We also include one pair of enantiomers (specifically the all-Λ and all-Δ enantiomers of the *T*1 diastereomer for all six tetra-anilines) to test the effect of enantiomer selection/initial conditions on the computational workflow (Supporting Information Section 15.3.4); the all-Δ *T*1 enantiomer is used in the analysis unless otherwise stated.

Table S5. Cage diastereomer, and the constituent facial configuration, of the experimentally observed crystal structures.

pseudo-cube	tetra-aniline	diastereomer	face-type in cage
**1**	**A**	$T1$	*i*
**2**	**B**	T_h2	*ii*
**3**	**C**	T_h2	*ii*
**4**	**D**	T_h2	*ii*
**5**	**E**	S_62	*iii*
**6**	**F**	D_32	*iii*

14 Density functional theory calculations

Due to the large size (more than 600 atoms) and number of the cages in this work, we have only performed preliminary density functional theory (DFT) optimizations on the set of diastereomers for one tetra-aniline, subcomponent **C** (Supporting Information Section 15.3.2). We used CP2K (version 8.1),[46] a plane-wave DFT code, for these calculations and the PBE[47] exchange-correlation function with Grimme-D3 dispersion corrections.[48] We used the molecularly optimized basis sets and potentials, with DZVP-MOLOPT-GTH atom basis sets for C, H and N and DZVP-MOLOPT-SR-GTH for Zn.[49] The periodic box was set for each cage structure to be 10 Å larger than the maximum cage diameter. The charge of all systems was set to 16+ and all calculations were in the gas phase. Using these settings, we tested grid variables to ensure convergence. Scripts to run the convergence tests, optimizations and analysis are in the available source code. In the first step, we selected three cage structures that are experimentally observed (formed from tetra-anilines **C** (T_h2), **D** (T_h2) and **E** (S_62)) and ran single-point energy evaluations of their GFN2-xTB optimized structures with a relative cutoff of 60 and varying the cutoff value. From these values, we selected a cutoff value of 700 Ry, where the relative DFT energy converges for all three systems. We then test the convergence of the DFT energy and atomic forces as a function of relative cutoff using a cutoff of 700 Ry. From this, we selected a relative cutoff of 60.

15 Cage-based modelling and geometric analysis

The ligand-based geometric approach in Supporting Information Section 11 was shown to be powerful for determining the relative stability of different Zn_8L_6 pseudo-cube diastereomers as a function of general geometric parameters. However, this approach is not sophisticated enough to accurately predict the preferred diastereomer for a given subcomponent. We thus targeted the development of a strategy which could circumvent the major pitfalls of the ligand-based approach — which required preselection of the relative arrangement of rectangular panels and it cannot accurately mimic the conformation of the ligand when constrained in the pseudo-cube geometry — in relation to the prediction of the single favored diastereomer for a given tetra-aniline. We did this by building and optimizing each pseudo-cube diastereomer, and then evaluating different properties to assess their relative stabilities.

15.1 Cage construction

Cages were constructed using our Python framework, *stk*,[27] by placing tetratopic panel building blocks (Figure S88) and preconstructed metal complex building blocks (Figure S89) on the "**M8L6Cube**" topology graph. The *stk* assembly process i) places building blocks on the vertices of a topology graph, and ii) aligns them, based on their functional groups, along the edges connecting that vertex to its neighbors. Vertices in *stk* correspond to nodes on a chemical graph and are not related to the shape of the eventual molecule (i.e., a vertex in *stk* does not describe exclusively pseudo-cube vertices). Building blocks were constructed and optimized as stated above (Supporting Information Section 9 for tetratopics, 10 for metal complexes); the most planar tetratopic panel conformer was used for cage construction. The cage assembly process places metal complex building blocks on eight three-connected vertices and the tetra-anilines on six four-connected vertices. In our software, *stko* (available at https://github.com/JelfsMaterialsGroup/stko), we have implemented methods to optimize metal-containing systems using the UFF4MOF forcefield[38,39] in the General Utility Lattice Program (GULP;[23,24] version 5.1), and the xtb[25,40] software. UFF4MOF is an extended version of the universal force field (UFF[37]) that handles metal environments common in metal-organic framework structures. The GFN*n*-xTB methods are recently developed and very efficient semiempirical quantum mechanical methods that were parameterized for large parts of the periodic table (up to Z=86)[25] and have been demonstrated to be reliable for the optimization of large transition metal-containing structures.[40]

We attempted to find the lowest energy cage conformer using the following sequence:

1. *stk* assembles structures based on predefined topology graphs with unphysical, long bonds between building blocks. The expanded structure is collapsed to a realistic size, while maintaining the shape of the assembled structure, by translating each building block toward the centroid of the assembled structure. The algorithm (**Collapser** in *stko*) stops when the inter-building block distance is less than 2.5 Å to avoid steric clashes.
2. The cage structure is geometry optimized using UFF4MOF in GULP. This optimization is performed in two steps: 1) with the conjugate gradient algorithm ("conj unit" option) and 2) with the second-order Newton-Raphson and BFGS hessian update (default options). The atom typing is handled by an *stko* implementation of the **ForceFieldHelpers** module in RDKit,[36] except for the metal atoms, which are manually typed to match the target types in UFF4MOF. Zinc(II) cations are assigned the square planar atom type, "Zn4+2".
3. A conformer search is performed starting from the UFF4MOF optimized cage structure using high-temperature molecular dynamics (MD). One MD run in the NVT ensemble, using the leapfrog verlet integrator, is performed using UFF4MOF and GULP at 400 K. A short equilibration is run for 0.1 ps and the production run is performed for 2.0 ps with a time step of 0.5 fs. From the production run, 10 conformers are extracted at 0.2 ps intervals.
4. The lowest energy cage conformer (based on UFF4MOF) is optimized using GFN2-xTB with the "normal" (energy converged within 5 x 10^{-6} Hartrees and gradient norm converged within 1 x 10^{-3} Hartree $bohr^{-1}$) convergence criteria and acetonitrile as the implicit solvent.

If the optimization failed in this automatic process, the structure was assumed unstable and no further optimization was attempted. Failure did not occur for any of the tetra-anilines and diastereomers included in this work.

15.2 Methods for cage analysis

15.2.1 Ligand strain

To analyze the ligand strain in a cage molecule, we first extract all of the individual organic ligands from the structure using the *NetworkX*[50] Python framework by deleting the metal cations and any associated bonds with them (Figure S101). We calculated the strain of each organic ligand compared to the lowest energy conformer of that ligand. The same lowest energy ligand conformer is used for all strain energy calculations for all cages containing that ligand. As alluded to above, in this case the ligand refers to the tetra-aniline with all four aniline groups condensed with 2-formylpyridine, but without chelation of metal centers (Figure S101, right).

The lowest energy conformer of the "free-ligand" for each tetra-aniline was calculated using the following procedure:

1. A CREST[26] conformer generation is run (version 2.9 used throughout) using GFN2-xTB in the gas phase and the "squick" mode and optimization level "3".
2. The lowest energy conformer is optimized at the "extreme" level (energy converged within 5 x 10^{-8} Hartrees and gradient norm converged within 5 x 10^{-5} Hartree $bohr^{-1}$) using GFN2-xTB in the gas phase.
3. That conformer is further optimized with implicit solvation (acetonitrile) in xTB at the "extreme" level.

Of the six lowest-energy ligand conformers (one for each of the six tetra-anilines) that this automated procedure produces, one lowest-energy conformer has one of the imines in the *cis*, not *trans* configuration. However, this is expected only to produce a systematic shift in strain energies when comparing the strain among the different pseudo-cube diastereomers formed by tetra-aniline **C**, and have little impact upon the discussion in Supporting Information Section 15.3.3.

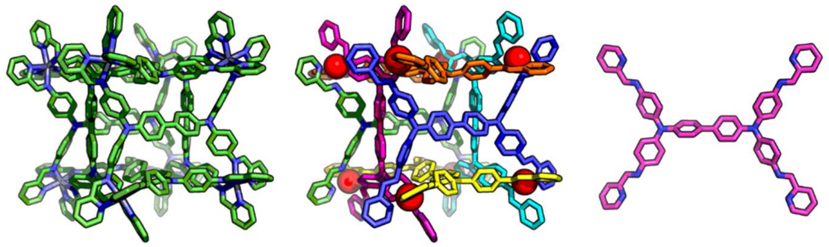

Figure S101. a) Cage structure. b) With each of the paneling ligands colored differently. (c) Extracted ligand.

We calculated the strain energy for each extracted ligand conformation (E_{strain}) as $E_{strain} = E_{extracted} - E_{free\text{-}ligand}$, where $E_{extracted}$ is the energy of the ligand extracted (without further optimization) from the

cage structure and $E_{free\text{-}ligand}$ is the energy of the lowest energy conformer. All ligand energies are calculated with acetonitrile implicit solvation.

15.2.2 Deviation from a perfect cube

We implemented a geometrical measure to investigate the deviation of the cage structures from a perfect cube, based on the positions of the Zn^{II} centers. We calculated the 8-node "CU-8" shape value from the positions of the metal cations in the cage structures using the SHAPE[29,51] software. The SHAPE software calculates continuous shape measures that quantify the distance of a set of points from the positions of the vertices of an ideal reference shape; it is based on the root-mean-squared deviation of the "distorted" structure from the ideal structure. Figure S102 shows examples of the effect of cube shape on the CU-8 value, where structures more closely resembling perfect cubes have lower scores. Scaled cubes score zero (structures 1, 2) and changes in one dimension (i.e., toward tetragonal prisms, structures 1, 3-6 in Figure S102) increase the measure monotonically. Random coordinates provide unclear changes in the shape measure (structures 7, 8), but are clearly non-zero.

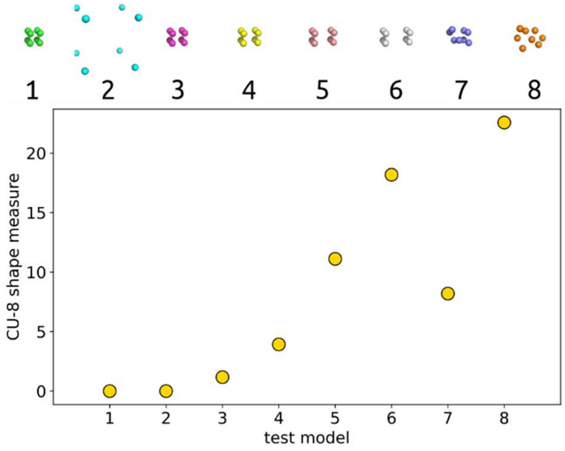

Figure S102. Test models showing the CU-8 shape measure of exemplar sets of eight spheres of the same atom type. Different colors are arbitrarily used for the different structures.

15.2.3 Porosity

The internal pore volume and diameter of each cage are calculated with our Python software, pyWindow.[52] This analysis assumes a spherical probe and a single pore, whose position is optimized to maximize the sphere fit in the pore. The pore diameter is calculated by determining the distance between the optimized center and the nearest atom, and multiplying by two; i.e., the largest sphere that can fit in the pore.

15.3 Cage analysis

For each cage structure, we use the above-defined methods to analyze their properties and relative stability.

15.3.1 X-ray structure analysis

We performed the same analysis on single-molecule cages extracted from the experimental crystal structures as we did on the *stk*-constructed cages ("calculated" models). We removed the disorder and solvent/counter-ions from the crystal structures and the structures were translated to the origin. Figure S103 compares the calculated structures and the X-ray structures. Unsurprisingly, the overlap between the structures is quite poor in most cases in terms of ring orientation in the ligands and the twist of some faces. However, the overall structures align well in terms of geometrical properties and pore sizes/shapes.

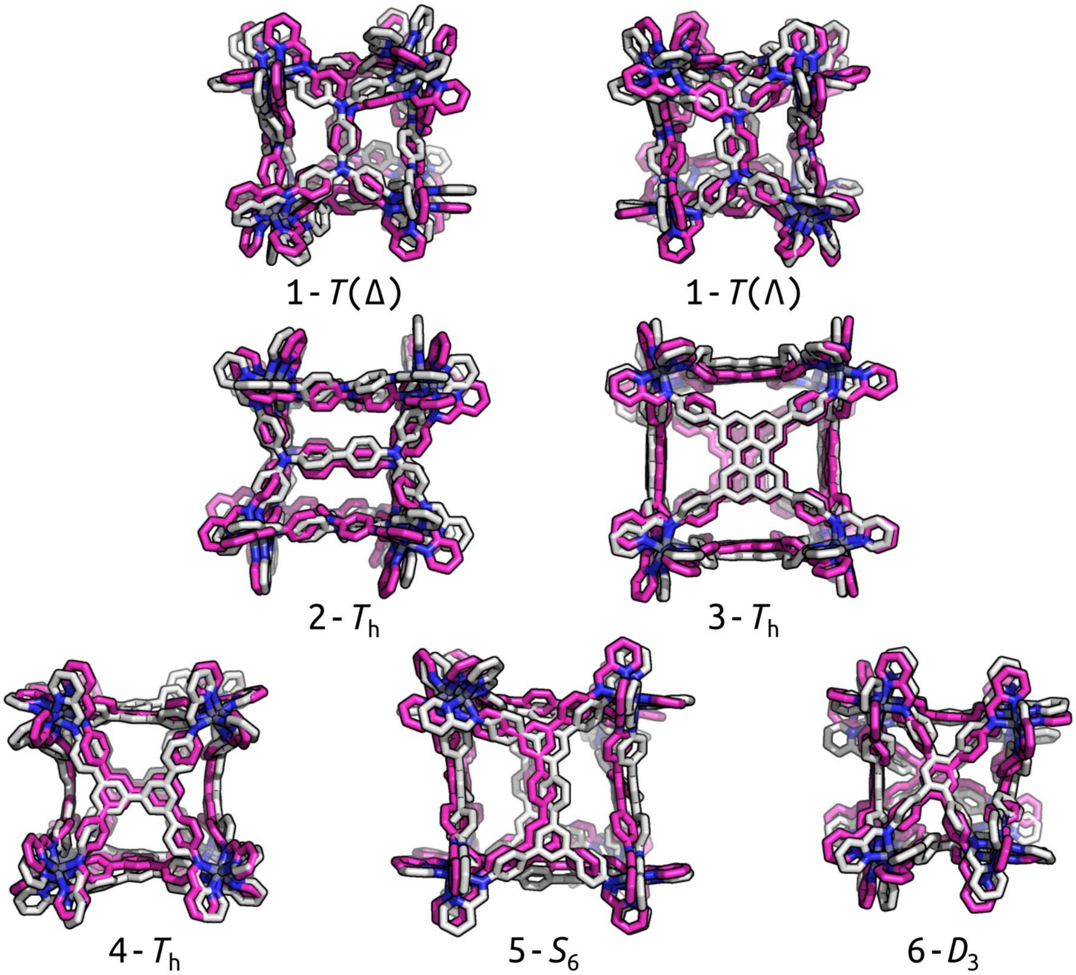

Figure S103. Overlap of computational and crystal structures. Computational structures are grey, X-ray structures are pink. Each structure is labeled with its pseudo-cube number and idealized point symmetry. Both of the enantiomers of *T*-symmetric pseudo-cube **1**, with metal centers with either all Λ or all Δ handedness, are shown, which we discuss further in Supporting Information Section 15.3.4 as validation of our approach. Hydrogens are omitted from both the models and crystal structures, with the added omission of disorder, anions and solvent molecules from the crystal structures, for clarity.

Figure S104 compares properties of the X-ray and calculated structures. We see that the measure of the shapes of the pseudo-cubes (Figure S104a) generally agree between the experimental and calculated structures. Importantly, the structures mostly do not significantly deviate from resembling regular cubes; structures **2** and **6**, formed by tetra-anilines **B** and **F**, respectively, deviate from a perfect cube by a greater amount than pseudo-cubes formed by the other tetra-anilines, however. It should be noted that as the CU-8 measure increases, the difference between the X-ray and calculated structure values is higher (Figure S104a). Figure S104b shows that the pore sizes of the X-ray structures are also reproduced by the calculated structure, which is crucial for property prediction, if that was the goal of the computational modelling.

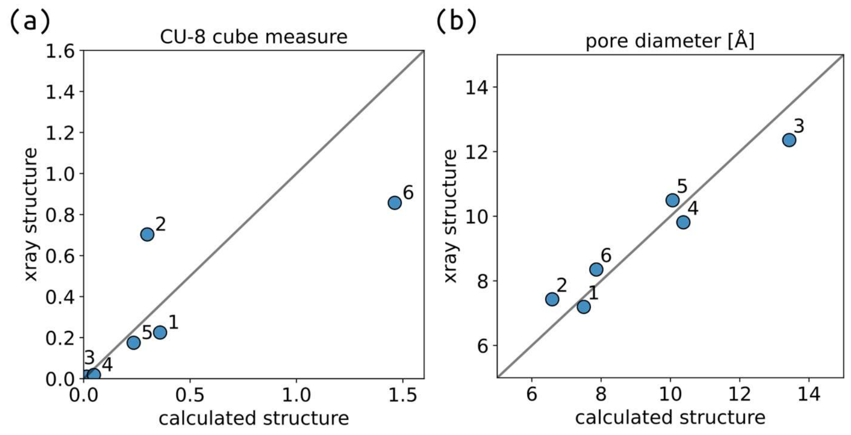

Figure S104. Parities of structural properties of X-ray and calculated structures. (a) Cube shape measure (Supporting information Section 15.2.2). (b) Pore diameter from pyWindow (section 15.2.3).

Figure S105 shows the comparison of all $Zn^{II}\cdots Zn^{II}$ distances in the X-ray and calculated structures; the first sets of peaks (less than, or around 16 Å) in each distribution represent the adjacent $Zn^{II}\cdots Zn^{II}$ distances. This data shows agreement, overall, between the experimental and calculated structures. There are small deviations, as expected, but agreement in the number of distinct adjacent $Zn^{II}\cdots Zn^{II}$ separation types in pseudo-cube **6** (three distinct types of adjacent $Zn^{II}\cdots Zn^{II}$ separation) is reassuring. The small deviations are expected due to the potential error in geometries from the semiempirical GFN2-xTB method, the lack of solvent/ions in the calculated structures and the contribution from crystal packing effects.

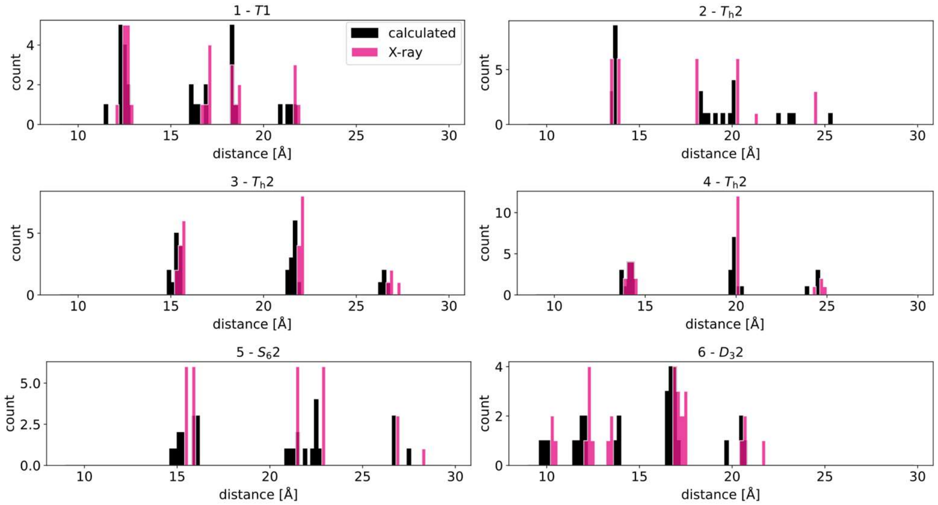

Figure S105. All $Zn^{II}\cdots Zn^{II}$ distances (the first sets of peaks correspond to distances between Zn^{II} centers connected by pseudo-cube edges) for pseudo-cubes **1**-**6** assembled from tetra-anilines **A**-**F**. Black distributions are for the computational structures, pink are for the X-ray structures.

15.3.2 Evaluating semiempirical and DFT relative energetics for diastereomer prediction

For the set of cages analyzed at the DFT-level (fourteen diastereomers, including both enantiomers for *T*1*,* formed from tetra-aniline **C**), we show that there are only small changes in cage structures from the GFN2-xTB optimized structures (Figure S106). However, more crucially, the relative energies show dramatic changes (Table S6). At the DFT-level, the lowest energy structure is the T_h2 diastereomer, which is experimentally observed, while this structure is 20 kJ mol^{-1} less stable than the $D_3$2 diastereomer at the GFN2-xTB level. At the xTB level, the relative energies are close, which suggests that we cannot discern between these structures using this method (considering the error in the method and the size/complexity of these cages). The most significant change is that the T_h2 structure is at least 200 kJ mol^{-1} more stable than any other structure at the DFT-level, which clearly supports its preferred formation. However, such large changes in the relative energies between methods suggests further optimization with higher levels of theory/larger basis sets would ideally be conducted in future prediction work. The compute time for these cages, using 10 nodes and 128 cores ranges from 4-10 hours. Therefore, expanding the number of cages evaluated is tractable but improving the method (larger basis set, hybrid functionals, solvation) may dramatically increase the cost. Overall, this is a set of promising results for future explorations of diastereomer prediction in large systems using DFT and highlights the importance of using DFT for making purely energetic arguments.

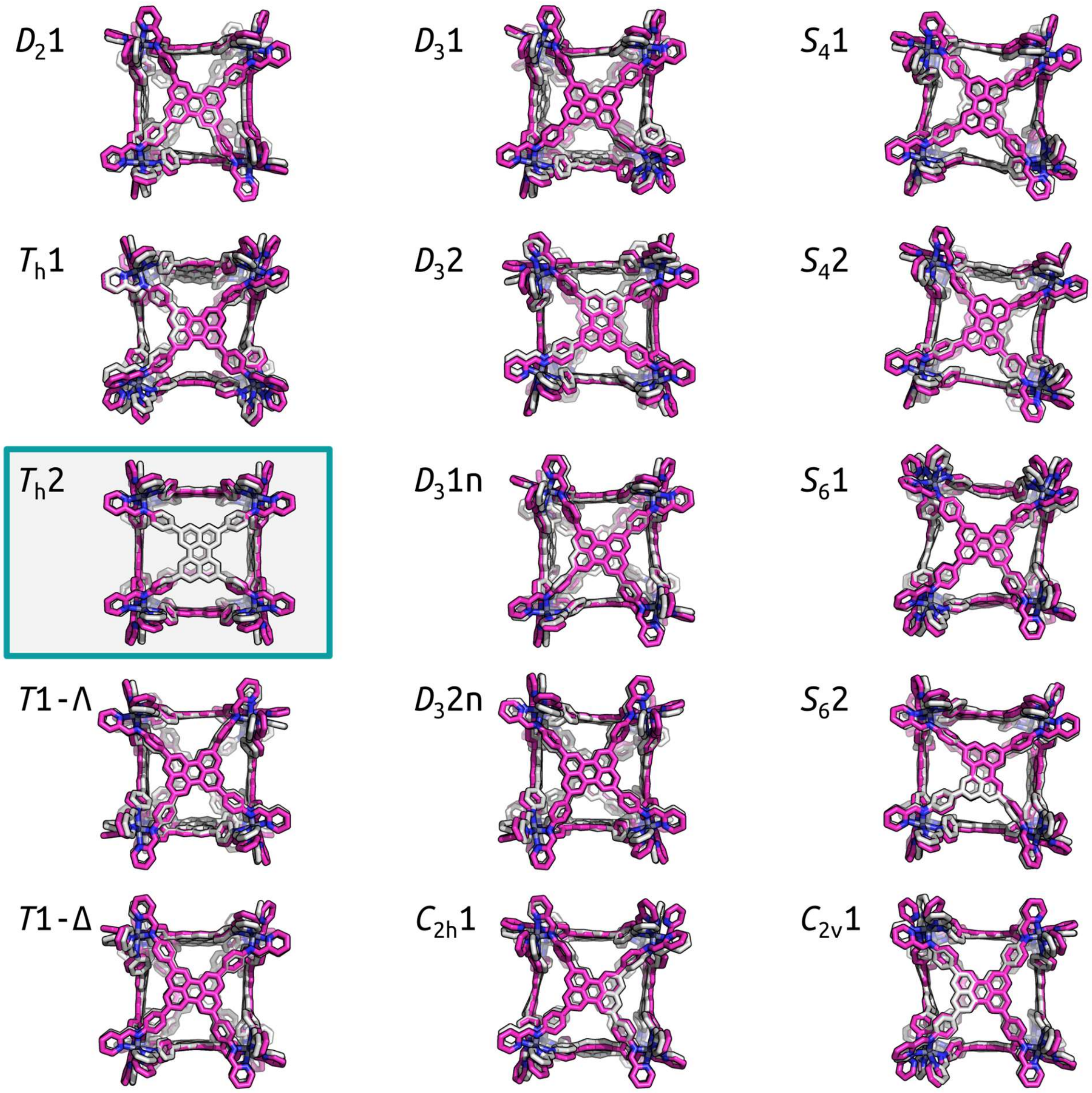

Figure S106. Comparison of GFN2-xTB (grey) and PBE/DZVP (pink) optimized pseudo-cube diastereomers formed from tetra-aniline **C**. Lowest energy diastereomer (by DFT) is highlighted. Models for both enantiomers of *T*1, with metal centers with either all Λ or all Δ handedness, are included (discussed in Supporting Information Section 15.3.4).

Table S6. Comparison of relative energies of cage structures, formed by tetra-aniline **C**, geometry optimized and energy-evaluated at the GFN2-xTB and PBE/DZVP (using CP2K) levels of theory. In each column, the shaded cell corresponds to the observed or lowest energy diastereomer. Models for both enantiomers of *T*1, with metal centers with either all Λ or all Δ handedness, are included (discussed in Supporting Information Section 15.3.4).

diastereomer	GFN2-xTB (kJ mol^{-1})	PBE/DZVP (kJ mol^{-1})
$D_2$1	66	215
T_h1	554	498
T_h2	20	0
*T*1-Δ	87	217
*T*1-Λ	86	241
$S_6$1	360	464
$S_6$2	112	310
$D_3$1	307	451
$D_3$2	0	290
$D_3$1n	47	254
$D_3$2n	27	363
$S_4$1	136	300
$S_4$2	186	455
C_{2v}1	230	337
C_{2h}1	250	279

As indicated for pseudo-cube **3** above, Figure S107 shows that the total cage energy from GFN2-xTB cannot conclusively predict the diastereomer selection. For example, three of the systems that are observed to form experimentally are not the lowest energy, by the GFN2-xTB method, of all examined diastereomers of the pseudo-cubes formed by the given tetra-aniline subcomponents. The largest deviation from zero relative energy occurs for pseudo-cube **4**, which is a kinetic product, with tetra-aniline **D** preferring to form an $M_{16}L_{12}$ structure.[5]

Therefore, the data presented in Table S6 and Figure S107 suggest that low-cost energy evaluation may not be sufficient for comparing the different pseudo-cube diastereomers. Because all cages have the same stoichiometry within a set of diastereomers, we compare the total energy, in this case the GFN2-xTB total energy, of the optimized cage structures in that set.

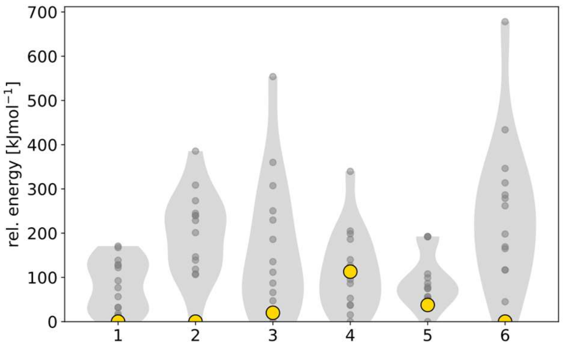

Figure S107. Relative GFN2-xTB energy of all diastereomers of pseudo-cubes **1**–**6**. Yellow points are the diastereomers that are observed experimentally to form selectively, grey points are all other diastereomers. Grey shaded regions approximate the density of points using the kernel density estimation with a bandwidth factor of 0.5.

15.3.3 Evaluating structure and strain across different diastereomers

Figure S108 shows a distribution of a measure describing the degree of deviation of the pseudo-cube structures from perfect cubes. Interestingly, only some diastereomers result in cage structures that deviate significantly from a perfect cube; these structures do still resemble cubes, however. It should be noted that such information regarding which diastereomers allow anisotropic structures cannot be obtained from the ligand-based approach described in Supporting Information Section 11.

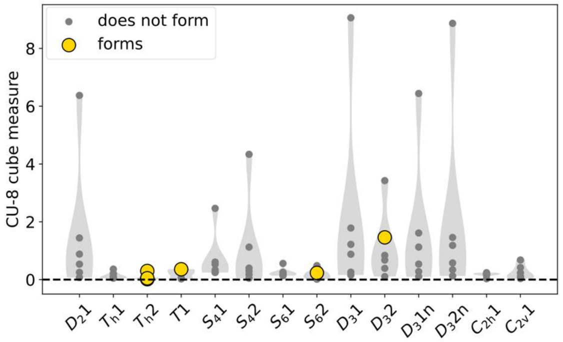

Figure S108. Distributions of the calculated cage CU-8 shape measure separated by diastereomer. Yellow markers are the values for structures that form experimentally, grey points are all other cages. The horizontal dashed line corresponds to a perfect cube measure of zero. Grey shaded regions approximate the density of points using the kernel density estimation with a bandwidth factor of 0.5.

Figure S109 shows the relative strain energy of each calculated cage structure constructed from each tetra-aniline **A**–**F**. We highlight the structures that were experimentally observed and we also color code points based on whether the diastereomeric configuration was identified to be either geometrically favored or unfavored using the ligand-based strategy described in Supporting Information Section 11.2.

This grouping of diastereomers indicates that the diastereomers predicted to be unfavored based on the ligand-based approach are often not among the structures with the least calculated ligand strain. However, there are outliers, for example, for the tetra-aniline **A** the $S_6$1 pseudo-cube diastereomer is more stable than $S_6$2 based on the evaluation of the total ligand strain in the diastereomers, highlighting the limitation of this approach. The high strain energy in an experimentally observed T_h2 diastereomer (for pseudo-cube **4**, formed from tetra-aniline **D**) also highlights the need for caution in over-interpreting these results. It should be noted, however, that the Zn_8L_6 pseudo-cube in this case is an isolable kinetic product, as opposed to the thermodynamic product which is a larger $Zn_{16}L_{12}$ capsule.[5]

Referring back to Supporting Information Section 11, edge types are assigned to be geometrically feasible if they provide a minimal $\Delta(Zn^{II}\cdots Zn^{II})$ value for ligand lengths meeting at an edge in at least one case (i.e., for at least one value of average $\Delta(N\cdots N)$). Assignment of an edge type as geometrically feasible does not suggest that the edge type would be favorable for all possible parings of tetra-aniline sides at a pseudo-cube edge. As illustrated in Figures S95 and S97, for a pair of tetra-aniline sides, with similar $N\cdots N$ separations (between imine-condensed N atoms), meeting at a pseudo-cube edge, an edge formed by a pair Zn^{II} centers with the same handedness would be anticipated to be geometrically favored, and the formation of an edge by two Zn^{II} centers with opposite handedness would be predicted to be geometrically disfavored. When the difference in length along tetra-aniline sides ($\Delta(N\cdots N)$) meeting at the pseudo-cube edge is larger, the opposite would be expected. This trend is at least in part replicated in the results in Figure S109. For example, at edges where tetra-aniline axes mismatch, the pair of Zn^{II} centers spanning the edge have the same handedness (i.e., edge type **E1** forms) in the *T*-symmetric diastereomer, which is predicted to have low relative ligand strain energy for pseudo-cube **1** assembled from low aspect ratio tetra-aniline **A**, and high strain for pseudo-cubes assembled from tetra-anilines **B** (**2**) and **F** (**6**) with higher aspect ratios. In contrast, Zn^{II} centers forming edges, where mismatching tetra-aniline axes meet, have solely opposite handedness (i.e., edge type **E4** forms) in diastereomers T_h2 and $D_3$2, which have high calculated relative ligand strain for pseudo-cube **1** and low calculated strain for pseudo-cubes **2** and **6**. Potential driving forces for the formation of the $D_3$2 diastereomer (over T_h2) are discussed in the main text; however, distinguishing between the energetics for the T_h2 and $D_3$2 diastereomeric configurations appears to be beyond the scope of the current ligand-based and cage-based computational analyses.

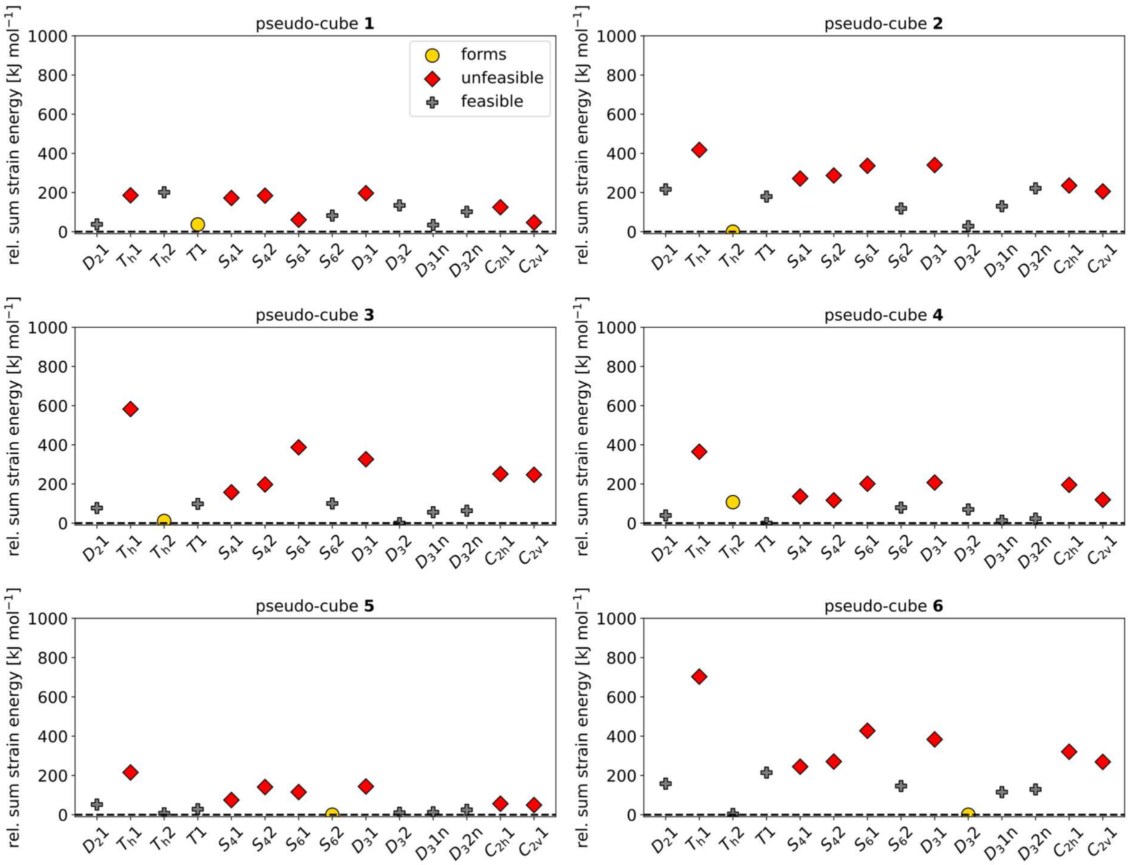

Figure S109. Calculated cage ligand strain energy separated by diastereomer for models of each pseudo-cube. Yellow markers are the values for structures that form experimentally, red points are cages with diastereomeric configurations predicted to be unfavored using the ligand-based approach, grey points are all other cages with diastereomeric configurations predicted to be favored using the ligand-based approach. All relative values are relative to the minimum value within a set of cages defined by a single tetra-aniline. Note that the minimum strain energy diastereomer of pseudo-cube **1** is the all-Λ model of the *T*1 diastereomer, not shown here, but discussed in Supporting Information Section 15.3.4.

15.3.4 Structural output and limitations

Ultimately, a high-throughput screening workflow must make concessions to avoid computationally expensive calculations. Considering the size of the cages studied in this work (more than 600 atoms), we have put together an optimization sequence that removes human intervention; however, such a process may result in poorly optimized structures. We check for failed optimizations or non-convergence during the optimization sequence; however, we do not manually fix/modify or solve issues with structures. For the most part, the optimization sequence, which includes a high temperature conformer search and multiple optimization steps, leads to reasonable and consistent structures. We expect that failures in this automated process suggest that the structures themselves are too strained and are not likely to form experimentally.

The concessions made to avoid computationally expensive calculations can give rise to limitations. The main example we look into that illustrates potential limitations are the all-Δ or all-Λ enantiomers of the *T*1 diastereomer (T1-Δ and *T*1-Λ, respectively), which should have identical values for sum strain energy, the CU-8 parameter and pore diameter. Figure S110 shows that the geometrical properties of *T*1-Δ and *T*1-Λ structures are mostly correlated. Picking an example, pseudo-cube **6** formed by tetra-aniline **F** (expected to have reasonably high strain given the high aspect ratio of the tetra-aniline), the *T*1-Λ structure looks more expanded than *T*1-Δ (Figure S110g), which is also supported by the greater pore diameter of **6**-*T*1-Λ compared to **6**-*T*1-Δ. The difference in the optimized structures of *T*1-Λ and *T*1-Δ may not only occur due to this being a high energy/disfavored structure though; comparing models of the all-Δ and all-Λ enantiomers for the pseudo-cube formed by tetra-aniline **A** (**1**) shows a variation in CU-8 measure (although these CU-8 values are small compared to some shown in Figure S108). Note that **1** does form the *T*-symmetric diastereomer experimentally (both the all-Δ and all-Λ enantiomers are present in the X-ray structure). This suggests that our optimization procedure can get stuck in different local energy minima along the optimization sequence, and thus implies that our calculated structures are not necessarily the global minimum on their potential energy surface, which is not surprising considering the low-cost sequence and the complexity of the cages. Both models of *T*1-Δ and *T*1-Λ structures successfully converge at the xTB level based on the checks in our workflow. We suggest the main difference occurs in the first step, i.e., *stk* construction, where the slight angle in the faces, which overlap in the two structures, results in the starting structures of the all-Δ and all-Λ enantiomers not being perfect mirror images; they will be at differing positions on the potential energy surface. This change in initial geometry (i.e., starting position on the potential energy surface) has a knock-on effect through the other optimization steps. Ultimately, the input structure has an impact on the outcome: the properties of *T*1-Δ and *T*1-Λ displayed below show close similarities but not perfect overlap for each model constructed from tetra-anilines **A**–**F**. Although a significant impact on the overall conclusions would not be expected, depending on which enantiomer model is used for the *T*1 diastereomer, the discussion in Supporting information Section S15.3.3 may have slightly changed.

Unfortunately, it is not possible to know *a priori* which of the optimized structures is most representative of the experimentally observed *T*-symmetric diastereomer. Importantly, we would expect the same outcome from this workflow if it were to be rerun, as the starting points (initial structures) would be the same each time. The above example thus emphasizes the importance of the structure generation/exploration process used when dealing with complex self-sorting, isomer possibilities.

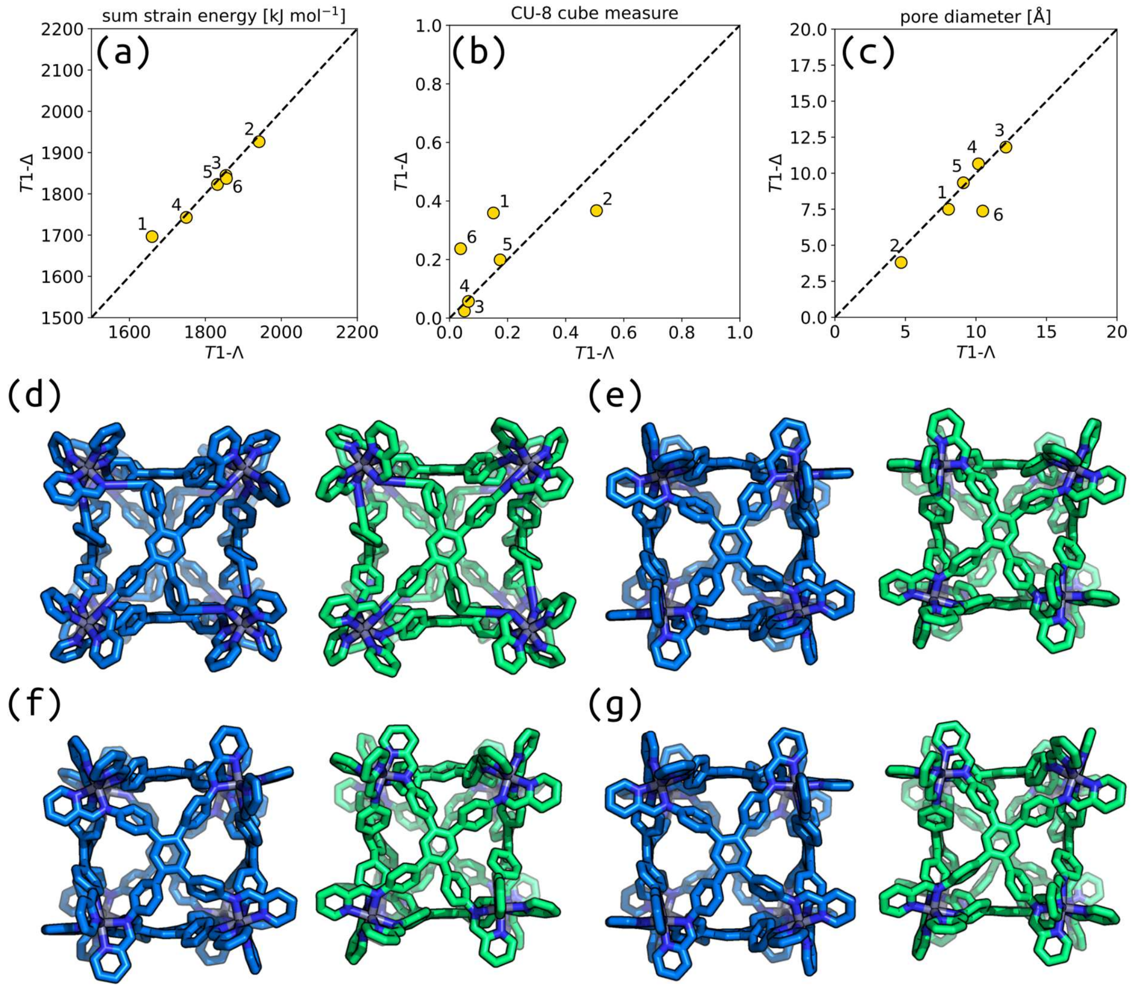

Figure S110. (a, b, c) Parity plots of cage properties of the two enantiomeric forms of the *T*1 diastereomer for pseudo-cubes **1**–**6**. (d-g) Comparing structures (all Δ: green, all Λ: blue) formed from tetra-aniline **F** during the optimization sequence. (d) Models output from *stk* after the **Collapser** algorithm is completed, note the near perfect overlap of the ligands, which is by definition. However, the connections with metal centers show distinct differences. (e and f) Models after the UFF optimization and UFF MD run, respectively. (g) Models after GFN2-xTB optimization, which are those analyzed throughout this work.

16 References

(1) Rosenau, C. P.; Jelier, B. J.; Gossert, A. D.; Togni, A. Exposing the Origins of Irreproducibility in Fluorine NMR Spectroscopy. *Angew. Chem. Int. Ed.* **2018**, *57*, 9528–9533.

(2) Wu, D. H.; Chen, A.; Johnson, C. S. An Improved Diffusion-Ordered Spectroscopy Experiment Incorporating Bipolar-Gradient Pulses. *J. Magn. Reson., Ser. A* **1995**, *115*, 260–264.

(3) Ascherl, L.; Evans, E. W.; Gorman, J.; Orsborne, S.; Bessinger, D.; Bein, T.; Friend, R. H.; Auras, F. Perylene-Based Covalent Organic Frameworks for Acid Vapor Sensing. *J. Am. Chem. Soc.* **2019**, *141*, 15693–15699.

(4) Galanti, A.; Santoro, J.; Mannancherry, R.; Duez, Q.; Diez-Cabanes, V.; Valášek, M.; De Winter, J.; Cornil, J.; Gerbaux, P.; Mayor, M.; Samorì, P. A New Class of Rigid Multi(azobenzene) Switches Featuring Electronic Decoupling: Unravelling the Isomerization in Individual Photochromes. *J. Am. Chem. Soc.* **2019**, *141*, 9273–9283.

(5) Davies, J. A.; Ronson, T. K.; Nitschke, J. R. Twisted Rectangular Subunits Self-Assemble into a Ferritin-Like Capsule. *Chem* **2022**, *8*, 1099–1106.

(6) Allan, D. R.; Nowell, H.; Barnett, S. A.; Warren, M. R.; Wilcox, A.; Christensen, J.; Saunders, L. K.; Peach, A.; Hooper, M. T.; Zaja, L.; Patel, S.; Cahill, L.; Marshall, R.; Trimnell, S.; Foster, A. J.; Bates, T.; Lay, S.; Williams, M. A.; Hathaway, P. V.; Winter, G.; Gerstel, M.; Wooley, R. W. A Novel Dual Air-Bearing Fixed-χ Diffractometer for Small-Molecule Single-Crystal X-ray Diffraction on Beamline I19 at Diamond Light Source. *Crystals* **2017**, *7*, 336.

(7) Evans, P. Scaling and Assessment of Data Quality. *Acta Cryst.* **2006**, *D62*, 72–82.

(8) Winter, G. *xia2*: An Expert System for Macromolecular Crystallography Data Reduction. *J. Appl. Crystallogr.* **2010**, *43*, 186–190.

(9) Winter, G.; Waterman, D. G.; Parkhurst, J. M.; Brewster, A. S.; Gildea, R. J.; Gerstel, M.; Fuentes-Montero, L.; Vollmar, M.; Michels-Clark, T.; Young, I. D.; Sauter, N. K.; Evans, G. DIALS: Implementation and Evaluation of a New Integration Package. *Acta Cryst.* **2018**, *D74*, 85–97.

(10) Farrugia, L. J. *WinGX* and *ORTEP* for Windows: An Update. *J. Appl. Crystallogr.* **2012**, *45*, 849–854.

(11) Evans, P. R.; Murshudov, G. N. How Good Are My Data and What is the Resolution? *Acta Cryst.* **2013**, *D69*, 1204–1214.

(12) Winn, M. D.; Ballard, C. C.; Cowtan, K. D.; Dodson, E. J.; Emsley, P.; Evans, P. R.; Keegan, R. M.; Krissinel, E. B.; Leslie, A. G. W.; McCoy, A.; McNicholas, S. J.; Murshudov, G. N.; Pannu, N. S.; Potterton, E. A.; Powell, H. R.; Read, R. J.; Vagin, A.; Wilson, K. S. Overview of the *CCP4* Suite and Current Developments. *Acta Cryst.* **2011**, *D67*, 235–242.

(13) Sheldrick, G. M. *SHELXT* - Integrated Space-Group and Crystal-Structure Determination. *Acta. Cryst.* **2015**, *A71*, 3–8.

(14) Sheldrick, G. M. Crystal Structure Refinement with *SHELXL*. *Acta. Cryst.* **2015**, *C71*, 3–8.

(15) Smart, O. S.; Sharff, A.; Holstein, J.; Womack, T. O.; Flensburg, C.; Keller, P.; Paciorek, W.; Vonrhein, C.; Bricogne G. *Grade2 version 1.3.0*. Global Phasing Ltd.: Cambridge, United Kingdom, 2021.

(16) Smart, O. S.; Womack, T. O., *Grade Web Server*. Global Phasing Ltd.: 2014.

(17) van der Sluis, P.; Spek, A. L. BYPASS: An Effective Method for the Refinement of Crystal Structures Containing Disordered Solvent Regions. *Acta Cryst.* **1990**, *A46*, 194–201.

(18) Spek, A. L., *PLATON: A Multipurpose Crystallographic Tool*. Utrecht University: Utrecht, The Netherlands, 2008.

(19) Maglic, J. B.; Lavendomme, R. *MoloVol*: An Easy-to-Use Program for Analyzing Cavities, Volumes and Surface Areas of Chemical Structures. *J. Appl. Crystallogr.* **2022**, *55*, 1033–1044.

(20) Kleywegt, G. J.; Jones, T. A. Detection, Delineation, Measurement and Display of Cavities in Macromolecular Structures. *Acta Cryst.* **1994**, *D50*, 178–185.

(21) Kieffer, M.; Bilbeisi, R. A.; Thoburn, J. D.; Clegg, J. K.; Nitschke, J. R. Guest Binding Drives Host Redistribution in Libraries of $Co^{II}_4L_4$ Cages. *Angew. Chem. Int. Ed.* **2020**, *59*, 11369–11373.

(22) Jiménez, A.; Bilbeisi, R. A.; Ronson, T. K.; Zarra, S.; Woodhead, C.; Nitschke, J. R. Selective Encapsulation and Sequential Release of Guests within a Self-Sorting Mixture of Three Tetrahedral Cages. *Angew. Chem. Int. Ed.* **2014**, *53*, 4556–4560.

(23) Gale, J. D. GULP: A Computer Program for the Symmetry-Adapted Simulation of Solids. *J. Chem. Soc., Faraday Trans.* **1997**, *93*, 629–637.

(24) Gale, J. D.; Rohl, A. L. The General Utility Lattice Program (GULP). *Mol. Simul.* **2003**, *29*, 291–341.

(25) Bannwarth, C.; Ehlert, S.; Grimme, S. GFN2-xTB—An Accurate and Broadly Parametrized Self-Consistent Tight-Binding Quantum Chemical Method with Multipole Electrostatics and Density-Dependent Dispersion Contributions. *J. Chem. Theory Comput.* **2019**, *15*, 1652–1671.

(26) Pracht, P.; Bohle, F.; Grimme, S. Automated Exploration of the Low-Energy Chemical Space with Fast Quantum Chemical Methods. *Phys. Chem. Chem. Phys.* **2020**, *22*, 7169–7192.

(27) Turcani, L.; Tarzia, A.; Szczypiński, F. T.; Jelfs, K. E. *stk*: An Extendable Python Framework for Automated Molecular and Supramolecular Structure Assembly and Discovery. *J. Chem. Phys.* **2021**, *154*, 214102.

(28) Bennett, S.; Tarzia, A.; Turcani, L. *stko,* **2022***,* https://github.com/JelfsMaterialsGroup/stko

(29) Casanova, D.; Llunell, M.; Alemany, P.; Alvarez, S. The Rich Stereochemistry of Eight-Vertex Polyhedra: A Continuous Shape Measures Study. *Chem. Eur. J.* **2005**, *11*, 1479–1494.

(30) Jiao, J.; Li, Z.; Qiao, Z.; Li, X.; Liu, Y.; Dong, J.; Jiang, J.; Cui, Y. Design and Self-Assembly of Hexahedral Coordination Cages for Cascade Reactions. *Nat. Commun.* **2018**, *9*, 4423.

(31) Liu, Y.; Lin, Z.; He, C.; Zhao, L.; Duan, C. A Symmetry-Controlled and Face-Driven Approach for the Assembly of Cerium-Based Molecular Polyhedra. *Dalton Trans.* **2010**, *39*, 11122–11125.

(32) Yang, L.; Jing, X.; He, C.; Chang, Z.; Duan, C. Redox-Active M_8L_6 Cubic Hosts with Tetraphenylethylene Faces Encapsulate Organic Dyes for Light-Driven H_2 Production. *Chem. Eur. J.* **2016**, *22*, 18107–18114.

(33) Ramsay, W. J.; Szczypiński, F. T.; Weissman, H.; Ronson, T. K.; Smulders, M. M. J.; Rybtchinski, B.; Nitschke, J. R. Designed Enclosure Enables Guest Binding Within the 4200 $Å^3$ Cavity of a Self-Assembled Cube. *Angew. Chem. Int. Ed.* **2015**, *54*, 5636–5640.

(34) Riniker, S.; Landrum, G. A. Better Informed Distance Geometry: Using What We Know To Improve Conformation Generation. *J. Chem. Inf. Model.* **2015**, *55*, 2562–2574.

(35) Wang, S.; Witek, J.; Landrum, G. A.; Riniker, S. Improving Conformer Generation for Small Rings and Macrocycles Based on Distance Geometry and Experimental Torsional-Angle Preferences. *J. Chem. Inf. Model.* **2020**, *60*, 2044–2058.

(36) Landrum, G. A. RDKit: Open-Source Cheminformatics., http://www.rdkit.org/, (accessed March 1, 2021).

(37) Rappe, A. K.; Casewit, C. J.; Colwell, K. S.; Goddard, W. A.; Skiff, W. M. UFF, a Full Periodic Table Force Field for Molecular Mechanics and Molecular Dynamics Simulations. *J. Am. Chem. Soc.* **1992**, *114*, 10024–10035.

(38) Addicoat, M. A.; Vankova, N.; Akter, I. F.; Heine, T. Extension of the Universal Force Field to Metal–Organic Frameworks. *J. Chem. Theory Comput.* **2014**, *10*, 880–891.

(39) Coupry, D. E.; Addicoat, M. A.; Heine, T. Extension of the Universal Force Field for Metal–Organic Frameworks. *J. Chem. Theory Comput.* **2016**, *12*, 5215–5225.

(40) Bursch, M.; Neugebauer, H.; Grimme, S. Structure Optimisation of Large Transition-Metal Complexes with Extended Tight-Binding Methods. *Angew. Chem. Int. Ed.* **2019**, *58*, 11078–11087.

(41) Meng, W.; Breiner, B.; Rissanen, K.; Thoburn, J. D.; Clegg, J. K.; Nitschke, J. R. A Self-Assembled M_8L_6 Cubic Cage that Selectively Encapsulates Large Aromatic Guests. *Angew. Chem. Int. Ed.* **2011**, *50*, 3479–3483.

(42) Bilbeisi, R. A.; Clegg, J. K.; Elgrishi, N.; de Hatten, X.; Devillard, M.; Breiner, B.; Mal, P.; Nitschke, J. R. Subcomponent Self-Assembly and Guest-Binding Properties of Face-Capped $Fe_4L_4^{8+}$ Capsules. *J. Am. Chem. Soc.* **2012**, *134*, 5110–5119.

(43) Sun, Q.-F.; Iwasa, J.; Ogawa, D.; Ishido, Y.; Sato, S.; Ozeki, T.; Sei, Y.; Yamaguchi, K.; Fujita, M. Self-Assembled $M_{24}L_{48}$ Polyhedra and Their Sharp Structural Switch upon Subtle Ligand Variation. *Science* **2010**, *328*, 1144–1147.

(44) Bloch, W. M.; Abe, Y.; Holstein, J. J.; Wandtke, C. M.; Dittrich, B.; Clever, G. H. Geometric Complementarity in Assembly and Guest Recognition of a Bent Heteroleptic *cis*-$[Pd_2L^A{}_2L^B{}_2]$ Coordination Cage. *J. Am. Chem. Soc.* **2016**, *138*, 13750–13755.

(45) Zhao, L.; Qu, S.; He, C.; Zhang, R.; Duan, C. Face-Driven Octanuclear Cerium(IV) Luminescence Polyhedra: Synthesis and Luminescent Sensing Natural Saccharides. *Chem. Commun.* **2011**, *47*, 9387–9389.

(46) Kühne, T. D.; Iannuzzi, M.; Del Ben, M.; Rybkin, V. V.; Seewald, P.; Stein, F.; Laino, T.; Khaliullin, R. Z.; Schütt, O.; Schiffmann, F.; Golze, D.; Wilhelm, J.; Chulkov, S.; Bani-Hashemian, M. H.; Weber, V.; Borštnik, U.; Taillefumier, M.; Jakobovits, A. S.; Lazzaro, A.; Pabst, H.; Müller, T.; Schade, R.; Guidon, M.; Andermatt, S.; Holmberg, N.; Schenter, G. K.; Hehn, A.; Bussy, A.; Belleflamme, F.; Tabacchi, G.; Glöß, A.; Lass, M.; Bethune, I.; Mundy, C. J.; Plessl, C.; Watkins, M.; VandeVondele, J.; Krack, M.; Hutter, J. CP2K: An Electronic Structure and Molecular Dynamics Software Package - Quickstep: Efficient and Accurate Electronic Structure Calculations. *J. Chem. Phys.* **2020**, *152*, 194103.

(47) Perdew, J. P.; Burke, K.; Ernzerhof, M. Generalized Gradient Approximation Made Simple. *Phys. Rev. Lett.* **1996**, *77*, 3865–3868.

(48) Grimme, S.; Antony, J.; Ehrlich, S.; Krieg, H. A Consistent and Accurate *ab initio* Parametrization of Density Functional Dispersion Correction (DFT-D) for the 94 Elements H-Pu. *J. Chem. Phys.* **2010**, *132*, 154104.

(49) VandeVondele, J.; Hutter, J. Gaussian Basis Sets for Accurate Calculations on Molecular Systems in Gas and Condensed Phases. *J. Chem. Phys.* **2007**, *127*, 114105.

(50) Hagberg, A. A.; Schult, D. A.; Swart, P. J. Exploring Network Structure, Dynamics, and Function Using NetworkX. *In Proceedings of the 7th Python in Science Conference* (SciPy 2008), Pasadena, CA, 2008; Varoquaux, G., Vaught, T., Millman, J., Eds.; pp 11–15.

(51) Pinsky, M.; Avnir, D. Continuous Symmetry Measures. 5. The Classical Polyhedra. *Inorg. Chem.* **1998**, *37*, 5575–5582.

(52) Miklitz, M.; Jelfs, K. E. pywindow: Automated Structural Analysis of Molecular Pores. *J. Chem. Inf. Model.* **2018**, *58*, 2387–2391.
