# Supplementary material for: Tetramine Aspect Ratio and Flexibility Determine Framework Symmetry for Zn8L6 Self‐Assembled Structures
Source: Angew Chem Int Ed Engl. 2023 Feb 1;62(10):e202217987. doi: 10.1002/anie.202217987 (PMC10946785; doi:10.1002/anie.202217987)

checkCIF/PLATON report

Structure factors have been supplied for datablock(s) jd370_sq

THIS REPORT IS FOR GUIDANCE ONLY. IF USED AS PART OF A REVIEW PROCEDURE FOR PUBLICATION, IT SHOULD NOT REPLACE THE EXPERTISE OF AN EXPERIENCED CRYSTALLOGRAPHIC REFEREE.

No syntax errors found.   CIF dictionary   Interpreting this report

Datablock: jd370_sq

```
Bond precision:   C-C = 0.0166 A          Wavelength=0.68890

Cell:           a=20.8181(6)       b=22.8600(8)      c=42.6867(14)
                alpha=90.355(3)    beta=97.922(3)    gamma=95.178(3)
Temperature:    100 K
```

	Calculated	Reported
Volume	20035.1(11)	20035.1(11)
Space group	P -1	P -1
Hall group	-P 1	-P 1
Moiety formula		
Sum formula	C337 H251.25 B13.73 F54.92 N66.50 Zn8 [+ solvent]	C337 H259.50 B16 F64 N66.50 Zn8
Mr	6947.17	7152.52
Dx,g cm-3	1.152	1.186
Z	2	2
Mu (mm-1)	0.506	0.510
F000	7083.3	7286.0
F000’	7091.10	
h,k,lmax	20,22,42	20,22,42
Nref	41976	41924
Tmin,Tmax	0.941,0.975	0.993,1.000
Tmin’	0.903	

```
Correction method= # Reported T Limits: Tmin=0.993 Tmax=1.000
AbsCorr = EMPIRICAL

Data completeness= 0.999          Theta(max)= 20.148

R(reflections)= 0.1278( 17539)          wR2(reflections)= 0.3788( 41924)

S = 1.124                 Npar= 4539
```

The following ALERTS were generated. Each ALERT has the format
test-name_ALERT_alert-type_alert-level.
Click on the hyperlinks for more details of the test.

Alert level A

```
THETM01_ALERT_3_A  The value of sine(theta_max)/wavelength is less than 0.550
            Calculated sin(theta_max)/wavelength =    0.5000
```

Author Response: The crystals were weakly diffracting and few reflections at greater than 1.0 A resolution were observed hence the data was trimmed accordingly.

Alert level B

```
PLAT084_ALERT_3_B High wR2 Value (i.e. > 0.25) ..................       0.38 Report
```

Author Response: The quality of the integration was lower than ideal due to the poor diffraction properties of the crystals and weak high angle data. As a consequence the values of the R1, wR and wR2 factors are larger than typical small molecule structures.

```
PLAT341_ALERT_3_B Low Bond Precision on  C-C Bonds ..............    0.01661 Ang.
```

Author Response: The low bond precision arises from the limited resolution of the data and thermal motion in some areas of the structure.

Alert level C

```
PLAT026_ALERT_3_C Ratio Observed / Unique Reflections (too) Low ..        42% Check
PLAT077_ALERT_4_C Unitcell Contains Non-integer Number of Atoms ..     Please Check
PLAT082_ALERT_2_C High R1 Value ..................................       0.13 Report
PLAT088_ALERT_3_C Poor Data / Parameter Ratio ....................       9.24 Note
PLAT094_ALERT_2_C Ratio of Maximum / Minimum Residual Density ....       3.21 Report
PLAT202_ALERT_3_C Isotropic non-H Atoms in Anion/Solvent .........         16 Check
              F33     F34     F35     F36     B9      F65              etc.
PLAT220_ALERT_2_C NonSolvent   Resd 1  C   Ueq(max)/Ueq(min) Range        3.3 Ratio
PLAT241_ALERT_2_C High   'MainMol' Ueq as Compared to Neighbors of       N10F Check
PLAT241_ALERT_2_C High   'MainMol' Ueq as Compared to Neighbors of        C2A Check
PLAT241_ALERT_2_C High   'MainMol' Ueq as Compared to Neighbors of        C3B Check
PLAT241_ALERT_2_C High   'MainMol' Ueq as Compared to Neighbors of        C3D Check
PLAT241_ALERT_2_C High   'MainMol' Ueq as Compared to Neighbors of        C3F Check
PLAT241_ALERT_2_C High   'MainMol' Ueq as Compared to Neighbors of        C4B Check
PLAT241_ALERT_2_C High   'MainMol' Ueq as Compared to Neighbors of        C4F Check
PLAT241_ALERT_2_C High   'MainMol' Ueq as Compared to Neighbors of        C8C Check
PLAT241_ALERT_2_C High   'MainMol' Ueq as Compared to Neighbors of        C8D Check
PLAT241_ALERT_2_C High   'MainMol' Ueq as Compared to Neighbors of       C11B Check
PLAT241_ALERT_2_C High   'MainMol' Ueq as Compared to Neighbors of       C11C Check
```

```
PLAT241_ALERT_2_C High   'MainMol' Ueq as Compared to Neighbors of       C12B Check
PLAT241_ALERT_2_C High   'MainMol' Ueq as Compared to Neighbors of       C12C Check
PLAT241_ALERT_2_C High   'MainMol' Ueq as Compared to Neighbors of       C20B Check
PLAT241_ALERT_2_C High   'MainMol' Ueq as Compared to Neighbors of       C21C Check
PLAT241_ALERT_2_C High   'MainMol' Ueq as Compared to Neighbors of       C23A Check
PLAT241_ALERT_2_C High   'MainMol' Ueq as Compared to Neighbors of       C23B Check
PLAT241_ALERT_2_C High   'MainMol' Ueq as Compared to Neighbors of       C23D Check
PLAT241_ALERT_2_C High   'MainMol' Ueq as Compared to Neighbors of       C23F Check
PLAT241_ALERT_2_C High   'MainMol' Ueq as Compared to Neighbors of       C24C Check
PLAT241_ALERT_2_C High   'MainMol' Ueq as Compared to Neighbors of       C27A Check
PLAT241_ALERT_2_C High   'MainMol' Ueq as Compared to Neighbors of       C27C Check
PLAT241_ALERT_2_C High   'MainMol' Ueq as Compared to Neighbors of       C30A Check
PLAT241_ALERT_2_C High   'MainMol' Ueq as Compared to Neighbors of       C32A Check
PLAT241_ALERT_2_C High   'MainMol' Ueq as Compared to Neighbors of       C32E Check
PLAT241_ALERT_2_C High   'MainMol' Ueq as Compared to Neighbors of       C33C Check
PLAT241_ALERT_2_C High   'MainMol' Ueq as Compared to Neighbors of       C35B Check
PLAT241_ALERT_2_C High   'MainMol' Ueq as Compared to Neighbors of       C35C Check
PLAT241_ALERT_2_C High   'MainMol' Ueq as Compared to Neighbors of       C35E Check
PLAT241_ALERT_2_C High   'MainMol' Ueq as Compared to Neighbors of       C35F Check
PLAT241_ALERT_2_C High   'MainMol' Ueq as Compared to Neighbors of       C36C Check
PLAT241_ALERT_2_C High   'MainMol' Ueq as Compared to Neighbors of       C38D Check
PLAT241_ALERT_2_C High   'MainMol' Ueq as Compared to Neighbors of       C38F Check
PLAT241_ALERT_2_C High   'MainMol' Ueq as Compared to Neighbors of       C39F Check
PLAT241_ALERT_2_C High   'MainMol' Ueq as Compared to Neighbors of       C40D Check
PLAT241_ALERT_2_C High   'MainMol' Ueq as Compared to Neighbors of       C40F Check
PLAT241_ALERT_2_C High   'MainMol' Ueq as Compared to Neighbors of       C44F Check
PLAT241_ALERT_2_C High   'MainMol' Ueq as Compared to Neighbors of       C45D Check
PLAT241_ALERT_2_C High   'MainMol' Ueq as Compared to Neighbors of       C50B Check
PLAT241_ALERT_2_C High   'MainMol' Ueq as Compared to Neighbors of       C51B Check
PLAT241_ALERT_2_C High   'MainMol' Ueq as Compared to Neighbors of       C51C Check
PLAT241_ALERT_2_C High   'MainMol' Ueq as Compared to Neighbors of       C53B Check
PLAT241_ALERT_2_C High   'MainMol' Ueq as Compared to Neighbors of       C53C Check
PLAT241_ALERT_2_C High   'MainMol' Ueq as Compared to Neighbors of       C53D Check
PLAT241_ALERT_2_C High   'MainMol' Ueq as Compared to Neighbors of       C54B Check
PLAT241_ALERT_2_C High   'MainMol' Ueq as Compared to Neighbors of       C54C Check
PLAT242_ALERT_2_C Low    'MainMol' Ueq as Compared to Neighbors of        Zn4 Check
PLAT242_ALERT_2_C Low    'MainMol' Ueq as Compared to Neighbors of        N6A Check
PLAT242_ALERT_2_C Low    'MainMol' Ueq as Compared to Neighbors of        C7A Check
PLAT242_ALERT_2_C Low    'MainMol' Ueq as Compared to Neighbors of        C7B Check
PLAT242_ALERT_2_C Low    'MainMol' Ueq as Compared to Neighbors of        C7C Check
PLAT242_ALERT_2_C Low    'MainMol' Ueq as Compared to Neighbors of        C7D Check
PLAT242_ALERT_2_C Low    'MainMol' Ueq as Compared to Neighbors of        C9C Check
PLAT242_ALERT_2_C Low    'MainMol' Ueq as Compared to Neighbors of       C10C Check
PLAT242_ALERT_2_C Low    'MainMol' Ueq as Compared to Neighbors of       C10F Check
PLAT242_ALERT_2_C Low    'MainMol' Ueq as Compared to Neighbors of       C19B Check
PLAT242_ALERT_2_C Low    'MainMol' Ueq as Compared to Neighbors of       C19C Check
PLAT242_ALERT_2_C Low    'MainMol' Ueq as Compared to Neighbors of       C31A Check
PLAT242_ALERT_2_C Low    'MainMol' Ueq as Compared to Neighbors of       C31C Check
PLAT242_ALERT_2_C Low    'MainMol' Ueq as Compared to Neighbors of       C31E Check
PLAT242_ALERT_2_C Low    'MainMol' Ueq as Compared to Neighbors of       C34B Check
PLAT242_ALERT_2_C Low    'MainMol' Ueq as Compared to Neighbors of       C34E Check
PLAT242_ALERT_2_C Low    'MainMol' Ueq as Compared to Neighbors of       C34F Check
PLAT242_ALERT_2_C Low    'MainMol' Ueq as Compared to Neighbors of       C41D Check
PLAT242_ALERT_2_C Low    'MainMol' Ueq as Compared to Neighbors of       C43F Check
PLAT242_ALERT_2_C Low    'MainMol' Ueq as Compared to Neighbors of       C46D Check
PLAT242_ALERT_2_C Low    'MainMol' Ueq as Compared to Neighbors of       C49B Check
PLAT242_ALERT_2_C Low    'MainMol' Ueq as Compared to Neighbors of       C49C Check
```

```
PLAT242_ALERT_2_C Low    'MainMol' Ueq as Compared to Neighbors of       C52B Check
PLAT242_ALERT_2_C Low    'MainMol' Ueq as Compared to Neighbors of       C52C Check
PLAT260_ALERT_2_C Large Average Ueq of Residue Including       Zn1      0.132 Check
PLAT260_ALERT_2_C Large Average Ueq of Residue Including        F1      0.148 Check
PLAT260_ALERT_2_C Large Average Ueq of Residue Including        F5      0.177 Check
PLAT260_ALERT_2_C Large Average Ueq of Residue Including        F9      0.157 Check
PLAT260_ALERT_2_C Large Average Ueq of Residue Including       F17      0.242 Check
PLAT260_ALERT_2_C Large Average Ueq of Residue Including       F21      0.184 Check
PLAT260_ALERT_2_C Large Average Ueq of Residue Including       F25      0.176 Check
PLAT260_ALERT_2_C Large Average Ueq of Residue Including       F41      0.204 Check
PLAT260_ALERT_2_C Large Average Ueq of Residue Including       N3S      0.131 Check
PLAT410_ALERT_2_C Short Intra H...H Contact  H6E      ..H8E      .       1.94 Ang.
                                                  x,y,z  =      1_555 Check
PLAT410_ALERT_2_C Short Intra H...H Contact  H42A     ..H48A     .       1.92 Ang.
                                                  x,y,z  =      1_555 Check
PLAT906_ALERT_3_C Large K Value in the Analysis of Variance ......      2.112 Check
PLAT911_ALERT_3_C Missing FCF Refl Between Thmin & STh/L=    0.500         53 Report
PLAT918_ALERT_3_C Reflection(s) with I(obs) much Smaller I(calc) .         10 Check
PLAT971_ALERT_2_C Check Calcd Resid. Dens.  1.36Ang From Zn7            2.11 eA-3
PLAT971_ALERT_2_C Check Calcd Resid. Dens.  1.27Ang From Zn8            1.93 eA-3
```

Alert level G

```
FORMU01_ALERT_1_G  There is a discrepancy between the atom counts in the
            _chemical_formula_sum and _chemical_formula_moiety. This is
            usually due to the moiety formula being in the wrong format.
            Atom count from _chemical_formula_sum:   C337 H259.5 B16 F64 N66.5 Zn8
            Atom count from _chemical_formula_moiety:
FORMU01_ALERT_2_G  There is a discrepancy between the atom counts in the
            _chemical_formula_sum and the formula from the _atom_site* data.
            Atom count from _chemical_formula_sum:C337 H259.5 B16 F64 N66.5 Zn8
            Atom count from the _atom_site data:  C337 H251.25 B13.72899 F54.91599
ABSMU01_ALERT_1_G  Calculation of _exptl_absorpt_correction_mu
                not performed for this radiation type.
CELLZ01_ALERT_1_G Difference between formula and atom_site contents detected.
CELLZ01_ALERT_1_G ALERT: Large difference may be due to a
            symmetry error - see SYMMG tests
           From the CIF: _cell_formula_units_Z    2
           From the CIF: _chemical_formula_sum  C337 H259.50 B16 F64 N66.50 Zn8
           TEST: Compare cell contents of formula and atom_site data

           atom    Z*formula  cif sites diff
           C        674.00    674.00    0.00
           H        519.00    502.50   16.50
           B         32.00     27.46    4.54
           F        128.00    109.83   18.17
           N        133.00    133.00    0.00
           Zn        16.00     16.00    0.00
PLAT002_ALERT_2_G Number of Distance or Angle Restraints on AtSite        561 Note
PLAT003_ALERT_2_G Number of Uiso or Uij Restrained non-H Atoms ...        573 Report
PLAT041_ALERT_1_G Calc. and Reported SumFormula    Strings  Differ     Please Check
PLAT042_ALERT_1_G Calc. and Reported MoietyFormula Strings  Differ     Please Check
PLAT092_ALERT_4_G Check: Wavelength Given is not Cu,Ga,Mo,Ag,In Ka    0.68890 Ang.
PLAT154_ALERT_1_G The s.u.'s on the Cell Angles are Equal ..(Note)      0.003 Degree
PLAT172_ALERT_4_G The CIF-Embedded .res File Contains DFIX Records        491 Report
PLAT173_ALERT_4_G The CIF-Embedded .res File Contains DANG Records        576 Report
PLAT174_ALERT_4_G The CIF-Embedded .res File Contains FLAT Records        498 Report
```

```
PLAT175_ALERT_4_G The CIF-Embedded .res File Contains SAME Records          2 Report
PLAT178_ALERT_4_G The CIF-Embedded .res File Contains SIMU Records         18 Report
PLAT186_ALERT_4_G The CIF-Embedded .res File Contains ISOR Records          6 Report
PLAT187_ALERT_4_G The CIF-Embedded .res File Contains RIGU Records          1 Report
PLAT244_ALERT_4_G Low    'Solvent' Ueq as Compared to Neighbors of         B1 Check
PLAT244_ALERT_4_G Low    'Solvent' Ueq as Compared to Neighbors of         B2 Check
PLAT244_ALERT_4_G Low    'Solvent' Ueq as Compared to Neighbors of         B3 Check
PLAT300_ALERT_4_G Atom Site Occupancy of N2S       Constrained at         0.5 Check
PLAT300_ALERT_4_G Atom Site Occupancy of C3S       Constrained at         0.5 Check
PLAT300_ALERT_4_G Atom Site Occupancy of C4S       Constrained at         0.5 Check
PLAT300_ALERT_4_G Atom Site Occupancy of H4S1      Constrained at         0.5 Check
PLAT300_ALERT_4_G Atom Site Occupancy of H4S2      Constrained at         0.5 Check
PLAT300_ALERT_4_G Atom Site Occupancy of H4S3      Constrained at         0.5 Check
PLAT300_ALERT_4_G Atom Site Occupancy of N3S       Constrained at        0.75 Check
PLAT300_ALERT_4_G Atom Site Occupancy of C5S       Constrained at        0.75 Check
PLAT300_ALERT_4_G Atom Site Occupancy of C6S       Constrained at        0.75 Check
PLAT300_ALERT_4_G Atom Site Occupancy of H6SA      Constrained at        0.75 Check
PLAT300_ALERT_4_G Atom Site Occupancy of H6SB      Constrained at        0.75 Check
PLAT300_ALERT_4_G Atom Site Occupancy of H6SC      Constrained at        0.75 Check
PLAT300_ALERT_4_G Atom Site Occupancy of N4S       Constrained at         0.5 Check
PLAT300_ALERT_4_G Atom Site Occupancy of C7S       Constrained at         0.5 Check
PLAT300_ALERT_4_G Atom Site Occupancy of C8S       Constrained at         0.5 Check
PLAT300_ALERT_4_G Atom Site Occupancy of N5S       Constrained at         0.5 Check
PLAT300_ALERT_4_G Atom Site Occupancy of C9S       Constrained at         0.5 Check
PLAT300_ALERT_4_G Atom Site Occupancy of C10S      Constrained at         0.5 Check
PLAT300_ALERT_4_G Atom Site Occupancy of H10A      Constrained at         0.5 Check
PLAT300_ALERT_4_G Atom Site Occupancy of H10B      Constrained at         0.5 Check
PLAT300_ALERT_4_G Atom Site Occupancy of H10C      Constrained at         0.5 Check
PLAT300_ALERT_4_G Atom Site Occupancy of N6S       Constrained at         0.5 Check
PLAT300_ALERT_4_G Atom Site Occupancy of C11S      Constrained at         0.5 Check
PLAT300_ALERT_4_G Atom Site Occupancy of C12S      Constrained at         0.5 Check
PLAT300_ALERT_4_G Atom Site Occupancy of H12G      Constrained at         0.5 Check
PLAT300_ALERT_4_G Atom Site Occupancy of H12H      Constrained at         0.5 Check
PLAT300_ALERT_4_G Atom Site Occupancy of H12I      Constrained at         0.5 Check
PLAT300_ALERT_4_G Atom Site Occupancy of N8S       Constrained at         0.5 Check
PLAT300_ALERT_4_G Atom Site Occupancy of C15S      Constrained at         0.5 Check
PLAT300_ALERT_4_G Atom Site Occupancy of C16S      Constrained at         0.5 Check
PLAT300_ALERT_4_G Atom Site Occupancy of H16G      Constrained at         0.5 Check
PLAT300_ALERT_4_G Atom Site Occupancy of H16H      Constrained at         0.5 Check
PLAT300_ALERT_4_G Atom Site Occupancy of H16J      Constrained at         0.5 Check
PLAT300_ALERT_4_G Atom Site Occupancy of N9S       Constrained at        0.25 Check
PLAT300_ALERT_4_G Atom Site Occupancy of C17S      Constrained at        0.25 Check
PLAT300_ALERT_4_G Atom Site Occupancy of C18S      Constrained at        0.25 Check
PLAT300_ALERT_4_G Atom Site Occupancy of N9Z       Constrained at        0.25 Check
PLAT300_ALERT_4_G Atom Site Occupancy of C17Z      Constrained at        0.25 Check
PLAT300_ALERT_4_G Atom Site Occupancy of C18Z      Constrained at        0.25 Check
PLAT300_ALERT_4_G Atom Site Occupancy of N10S      Constrained at        0.25 Check
PLAT300_ALERT_4_G Atom Site Occupancy of C19S      Constrained at        0.25 Check
PLAT300_ALERT_4_G Atom Site Occupancy of C20S      Constrained at        0.25 Check
PLAT300_ALERT_4_G Atom Site Occupancy of N11S      Constrained at        0.25 Check
PLAT300_ALERT_4_G Atom Site Occupancy of C21S      Constrained at        0.25 Check
PLAT300_ALERT_4_G Atom Site Occupancy of C22S      Constrained at        0.25 Check
PLAT300_ALERT_4_G Atom Site Occupancy of N12S      Constrained at        0.25 Check
PLAT300_ALERT_4_G Atom Site Occupancy of C23S      Constrained at        0.25 Check
PLAT300_ALERT_4_G Atom Site Occupancy of C24S      Constrained at        0.25 Check
PLAT301_ALERT_3_G Main Residue  Disorder .............(Resd  1  )          5% Note
PLAT302_ALERT_4_G Anion/Solvent/Minor-Residue Disorder (Resd  5  )        100% Note
```

```
PLAT302_ALERT_4_G Anion/Solvent/Minor-Residue Disorder (Resd  6  )        100% Note
PLAT302_ALERT_4_G Anion/Solvent/Minor-Residue Disorder (Resd  7  )        100% Note
PLAT302_ALERT_4_G Anion/Solvent/Minor-Residue Disorder (Resd  8  )        100% Note
PLAT302_ALERT_4_G Anion/Solvent/Minor-Residue Disorder (Resd  9  )        100% Note
PLAT302_ALERT_4_G Anion/Solvent/Minor-Residue Disorder (Resd 10  )        100% Note
PLAT302_ALERT_4_G Anion/Solvent/Minor-Residue Disorder (Resd 11  )        100% Note
PLAT302_ALERT_4_G Anion/Solvent/Minor-Residue Disorder (Resd 12  )        100% Note
PLAT302_ALERT_4_G Anion/Solvent/Minor-Residue Disorder (Resd 13  )        100% Note
PLAT302_ALERT_4_G Anion/Solvent/Minor-Residue Disorder (Resd 14  )        100% Note
PLAT302_ALERT_4_G Anion/Solvent/Minor-Residue Disorder (Resd 15  )        100% Note
PLAT302_ALERT_4_G Anion/Solvent/Minor-Residue Disorder (Resd 16  )        100% Note
PLAT302_ALERT_4_G Anion/Solvent/Minor-Residue Disorder (Resd 17  )        100% Note
PLAT302_ALERT_4_G Anion/Solvent/Minor-Residue Disorder (Resd 18  )        100% Note
PLAT302_ALERT_4_G Anion/Solvent/Minor-Residue Disorder (Resd 19  )        100% Note
PLAT302_ALERT_4_G Anion/Solvent/Minor-Residue Disorder (Resd 20  )        100% Note
PLAT302_ALERT_4_G Anion/Solvent/Minor-Residue Disorder (Resd 21  )        100% Note
PLAT302_ALERT_4_G Anion/Solvent/Minor-Residue Disorder (Resd 22  )        100% Note
PLAT302_ALERT_4_G Anion/Solvent/Minor-Residue Disorder (Resd 23  )        100% Note
PLAT302_ALERT_4_G Anion/Solvent/Minor-Residue Disorder (Resd 24  )        100% Note
PLAT302_ALERT_4_G Anion/Solvent/Minor-Residue Disorder (Resd 25  )        100% Note
PLAT302_ALERT_4_G Anion/Solvent/Minor-Residue Disorder (Resd 26  )        100% Note
PLAT302_ALERT_4_G Anion/Solvent/Minor-Residue Disorder (Resd 27  )        100% Note
PLAT302_ALERT_4_G Anion/Solvent/Minor-Residue Disorder (Resd 28  )        100% Note
PLAT302_ALERT_4_G Anion/Solvent/Minor-Residue Disorder (Resd 29  )        100% Note
PLAT302_ALERT_4_G Anion/Solvent/Minor-Residue Disorder (Resd 30  )        100% Note
PLAT302_ALERT_4_G Anion/Solvent/Minor-Residue Disorder (Resd 31  )        100% Note
PLAT302_ALERT_4_G Anion/Solvent/Minor-Residue Disorder (Resd 32  )        100% Note
PLAT302_ALERT_4_G Anion/Solvent/Minor-Residue Disorder (Resd 33  )        100% Note
PLAT302_ALERT_4_G Anion/Solvent/Minor-Residue Disorder (Resd 34  )        100% Note
PLAT302_ALERT_4_G Anion/Solvent/Minor-Residue Disorder (Resd 35  )        100% Note
PLAT302_ALERT_4_G Anion/Solvent/Minor-Residue Disorder (Resd 36  )        100% Note
PLAT302_ALERT_4_G Anion/Solvent/Minor-Residue Disorder (Resd 37  )        100% Note
PLAT302_ALERT_4_G Anion/Solvent/Minor-Residue Disorder (Resd 38  )        100% Note
PLAT302_ALERT_4_G Anion/Solvent/Minor-Residue Disorder (Resd 39  )        100% Note
PLAT302_ALERT_4_G Anion/Solvent/Minor-Residue Disorder (Resd 40  )        100% Note
PLAT302_ALERT_4_G Anion/Solvent/Minor-Residue Disorder (Resd 41  )        100% Note
PLAT304_ALERT_4_G Non-Integer Number of Atoms in ..... (Resd  5  )        3.59 Check
PLAT304_ALERT_4_G Non-Integer Number of Atoms in ..... (Resd  6  )        4.20 Check
PLAT304_ALERT_4_G Non-Integer Number of Atoms in ..... (Resd  7  )        4.20 Check
PLAT304_ALERT_4_G Non-Integer Number of Atoms in ..... (Resd  8  )        2.52 Check
PLAT304_ALERT_4_G Non-Integer Number of Atoms in ..... (Resd  9  )        3.31 Check
PLAT304_ALERT_4_G Non-Integer Number of Atoms in ..... (Resd 10  )        2.50 Check
PLAT304_ALERT_4_G Non-Integer Number of Atoms in ..... (Resd 11  )        3.42 Check
PLAT304_ALERT_4_G Non-Integer Number of Atoms in ..... (Resd 12  )        2.34 Check
PLAT304_ALERT_4_G Non-Integer Number of Atoms in ..... (Resd 13  )        1.10 Check
PLAT304_ALERT_4_G Non-Integer Number of Atoms in ..... (Resd 14  )        2.50 Check
PLAT304_ALERT_4_G Non-Integer Number of Atoms in ..... (Resd 15  )        2.49 Check
PLAT304_ALERT_4_G Non-Integer Number of Atoms in ..... (Resd 16  )        2.12 Check
PLAT304_ALERT_4_G Non-Integer Number of Atoms in ..... (Resd 17  )        1.88 Check
PLAT304_ALERT_4_G Non-Integer Number of Atoms in ..... (Resd 18  )        1.63 Check
PLAT304_ALERT_4_G Non-Integer Number of Atoms in ..... (Resd 19  )        1.79 Check
PLAT304_ALERT_4_G Non-Integer Number of Atoms in ..... (Resd 20  )        1.56 Check
PLAT304_ALERT_4_G Non-Integer Number of Atoms in ..... (Resd 21  )        2.03 Check
PLAT304_ALERT_4_G Non-Integer Number of Atoms in ..... (Resd 22  )        2.25 Check
PLAT304_ALERT_4_G Non-Integer Number of Atoms in ..... (Resd 23  )        2.40 Check
PLAT304_ALERT_4_G Non-Integer Number of Atoms in ..... (Resd 24  )        2.23 Check
PLAT304_ALERT_4_G Non-Integer Number of Atoms in ..... (Resd 25  )        2.03 Check
```

```
PLAT304_ALERT_4_G Non-Integer Number of Atoms in ..... (Resd 26  )       1.59 Check
PLAT304_ALERT_4_G Non-Integer Number of Atoms in ..... (Resd 27  )       1.99 Check
PLAT304_ALERT_4_G Non-Integer Number of Atoms in ..... (Resd 29  )       4.50 Check
PLAT304_ALERT_4_G Non-Integer Number of Atoms in ..... (Resd 30  )       1.50 Check
PLAT304_ALERT_4_G Non-Integer Number of Atoms in ..... (Resd 33  )       3.46 Check
PLAT304_ALERT_4_G Non-Integer Number of Atoms in ..... (Resd 35  )       1.01 Check
PLAT304_ALERT_4_G Non-Integer Number of Atoms in ..... (Resd 36  )       2.54 Check
PLAT304_ALERT_4_G Non-Integer Number of Atoms in ..... (Resd 37  )       0.75 Check
PLAT304_ALERT_4_G Non-Integer Number of Atoms in ..... (Resd 38  )       0.75 Check
PLAT304_ALERT_4_G Non-Integer Number of Atoms in ..... (Resd 39  )       0.75 Check
PLAT304_ALERT_4_G Non-Integer Number of Atoms in ..... (Resd 40  )       0.75 Check
PLAT304_ALERT_4_G Non-Integer Number of Atoms in ..... (Resd 41  )       0.75 Check
PLAT315_ALERT_2_G Singly Bonded Carbon Detected (H-atoms Missing).        C2S Check
PLAT315_ALERT_2_G Singly Bonded Carbon Detected (H-atoms Missing).        C8S Check
PLAT315_ALERT_2_G Singly Bonded Carbon Detected (H-atoms Missing).        C2Z Check
PLAT315_ALERT_2_G Singly Bonded Carbon Detected (H-atoms Missing).       C18S Check
PLAT315_ALERT_2_G Singly Bonded Carbon Detected (H-atoms Missing).       C18Z Check
PLAT315_ALERT_2_G Singly Bonded Carbon Detected (H-atoms Missing).       C20S Check
PLAT315_ALERT_2_G Singly Bonded Carbon Detected (H-atoms Missing).       C22S Check
PLAT315_ALERT_2_G Singly Bonded Carbon Detected (H-atoms Missing).       C24S Check
PLAT410_ALERT_2_G Short Intra H...H Contact  H18I     ..H24F     .       1.90 Ang.
                                                      x,y,z  =     1_555 Check
PLAT410_ALERT_2_G Short Intra H...H Contact  H20E     ..H44G     .       2.10 Ang.
                                                      x,y,z  =     1_555 Check
PLAT410_ALERT_2_G Short Intra H...H Contact  H33B     ..H45H     .       1.65 Ang.
                                                      x,y,z  =     1_555 Check
PLAT410_ALERT_2_G Short Intra H...H Contact  H44D     ..H48H     .       1.75 Ang.
                                                      x,y,z  =     1_555 Check
PLAT411_ALERT_2_G Short Inter H...H Contact  H40F     ..H16F     .       2.10 Ang.
                                                   x,-1+y,z  =     1_545 Check
PLAT413_ALERT_2_G Short Inter XH3 .. XHn     H26E     ..H14K     .       2.14 Ang.
                                                 1-x,1-y,-z  =     2_665 Check
PLAT413_ALERT_2_G Short Inter XH3 .. XHn     H40A     ..H14L     .       1.63 Ang.
                                                      x,y,z  =     1_555 Check
PLAT413_ALERT_2_G Short Inter XH3 .. XHn     H4S1     ..H24E     .       2.14 Ang.
                                                  1-x,-y,-z  =     2_655 Check
PLAT413_ALERT_2_G Short Inter XH3 .. XHn     H4S3     ..H23E     .       2.07 Ang.
                                                      x,y,z  =     1_555 Check
PLAT413_ALERT_2_G Short Inter XH3 .. XHn     H10B     ..H38D     .       2.00 Ang.
                                                2-x,1-y,1-z  =     2_766 Check
PLAT413_ALERT_2_G Short Inter XH3 .. XHn     H14G     ..H25E     .       1.71 Ang.
                                                 1-x,1-y,-z  =     2_665 Check
PLAT432_ALERT_2_G Short Inter X...Y Contact  F1       ..C42A     .       2.91 Ang.
                                                   -x,-y,-z  =     2_555 Check
PLAT432_ALERT_2_G Short Inter X...Y Contact  F4       ..C5E      .       2.95 Ang.
                                                      x,y,z  =     1_555 Check
PLAT432_ALERT_2_G Short Inter X...Y Contact  F9       ..C30D     .       2.95 Ang.
                                                      x,y,z  =     1_555 Check
PLAT432_ALERT_2_G Short Inter X...Y Contact  F11      ..C9S      .       2.86 Ang.
                                                      x,y,z  =     1_555 Check
PLAT432_ALERT_2_G Short Inter X...Y Contact  F17      ..C18F     .       2.88 Ang.
                                                1-x,1-y,1-z  =     2_666 Check
PLAT432_ALERT_2_G Short Inter X...Y Contact  F13A     ..C30C     .       2.70 Ang.
                                                      x,y,z  =     1_555 Check
PLAT432_ALERT_2_G Short Inter X...Y Contact  F14      ..C28C     .       2.97 Ang.
                                                      x,y,z  =     1_555 Check
PLAT432_ALERT_2_G Short Inter X...Y Contact  F31      ..C38A     .       2.87 Ang.
```

```
                                                -x,1-y,-z  =      2_565 Check
PLAT432_ALERT_2_G Short Inter X...Y Contact  F37      ..C12S     .       2.78 Ang.
                                                 -1+x,y,z  =      1_455 Check
PLAT432_ALERT_2_G Short Inter X...Y Contact  F45A     ..C2F      .       2.90 Ang.
                                                -x,-y,1-z  =      2_556 Check
PLAT432_ALERT_2_G Short Inter X...Y Contact  F55      ..C16S     .       2.82 Ang.
                                                    x,y,z  =      1_555 Check
PLAT432_ALERT_2_G Short Inter X...Y Contact  F55      ..C4C      .       2.89 Ang.
                                                    x,y,z  =      1_555 Check
PLAT432_ALERT_2_G Short Inter X...Y Contact  F55      ..C6C      .       2.89 Ang.
                                                    x,y,z  =      1_555 Check
PLAT432_ALERT_2_G Short Inter X...Y Contact  F56      ..C15S     .       2.12 Ang.
                                                    x,y,z  =      1_555 Check
PLAT432_ALERT_2_G Short Inter X...Y Contact  F56      ..C16S     .       2.71 Ang.
                                                    x,y,z  =      1_555 Check
PLAT432_ALERT_2_G Short Inter X...Y Contact  F66A     ..C25A     .       2.97 Ang.
                                                 x,1+y,z  =      1_565 Check
PLAT432_ALERT_2_G Short Inter X...Y Contact  C28C     ..C24S     .       3.17 Ang.
                                                x,-1+y,z  =      1_545 Check
PLAT432_ALERT_2_G Short Inter X...Y Contact  C30C     ..N12S     .       3.03 Ang.
                                                x,-1+y,z  =      1_545 Check
PLAT432_ALERT_2_G Short Inter X...Y Contact  C36C     ..N12S     .       2.69 Ang.
                                                x,-1+y,z  =      1_545 Check
PLAT432_ALERT_2_G Short Inter X...Y Contact  C40A     ..C14Z     .       3.05 Ang.
                                                    x,y,z  =      1_555 Check
PLAT606_ALERT_4_G Solvent Accessible VOID(S) in Structure .......          ! Info
PLAT720_ALERT_4_G Number of Unusual/Non-Standard Labels .........          6 Note
PLAT722_ALERT_1_G Angle   Calc      108.00, Rep      109.50 Dev...       1.50 Degree
              H14L    -C14Z    -H14M       1_555  1_555  1_555     # 1539 Check
PLAT790_ALERT_4_G Centre of Gravity not Within Unit Cell: Resd.  #         2 Note
              B F4
PLAT790_ALERT_4_G Centre of Gravity not Within Unit Cell: Resd.  #         3 Note
              B F4
PLAT790_ALERT_4_G Centre of Gravity not Within Unit Cell: Resd.  #         4 Note
              B F4
PLAT790_ALERT_4_G Centre of Gravity not Within Unit Cell: Resd.  #        10 Note
              B F4
PLAT790_ALERT_4_G Centre of Gravity not Within Unit Cell: Resd.  #        12 Note
              B F4
PLAT790_ALERT_4_G Centre of Gravity not Within Unit Cell: Resd.  #        13 Note
              B F4
PLAT790_ALERT_4_G Centre of Gravity not Within Unit Cell: Resd.  #        19 Note
              B F4
PLAT790_ALERT_4_G Centre of Gravity not Within Unit Cell: Resd.  #        20 Note
              B F4
PLAT790_ALERT_4_G Centre of Gravity not Within Unit Cell: Resd.  #        22 Note
              B F4
PLAT790_ALERT_4_G Centre of Gravity not Within Unit Cell: Resd.  #        23 Note
              B F4
PLAT790_ALERT_4_G Centre of Gravity not Within Unit Cell: Resd.  #        24 Note
              B F4
PLAT790_ALERT_4_G Centre of Gravity not Within Unit Cell: Resd.  #        25 Note
              B F4
PLAT790_ALERT_4_G Centre of Gravity not Within Unit Cell: Resd.  #        31 Note
              C2 H3 N
PLAT790_ALERT_4_G Centre of Gravity not Within Unit Cell: Resd.  #        34 Note
              C2 H3 N
```

```
PLAT790_ALERT_4_G Centre of Gravity not Within Unit Cell: Resd.  #         40 Note
              C2 N
PLAT794_ALERT_5_G Tentative Bond Valency for Zn1       (II)      .       1.86 Info
PLAT794_ALERT_5_G Tentative Bond Valency for Zn2       (II)      .       1.69 Info
PLAT794_ALERT_5_G Tentative Bond Valency for Zn4       (II)      .       1.79 Info
PLAT794_ALERT_5_G Tentative Bond Valency for Zn5       (II)      .       1.76 Info
PLAT794_ALERT_5_G Tentative Bond Valency for Zn6       (II)      .       1.67 Info
PLAT794_ALERT_5_G Tentative Bond Valency for Zn7       (II)      .       1.68 Info
PLAT802_ALERT_4_G CIF Input Record(s) with more than 80 Characters          1 Info
PLAT860_ALERT_3_G Number of Least-Squares Restraints .............       9093 Note
PLAT869_ALERT_4_G ALERTS Related to the Use of SQUEEZE  Suppressed          ! Info
PLAT883_ALERT_1_G No Info/Value for _atom_sites_solution_primary .     Please Do !
PLAT910_ALERT_3_G Missing # of FCF Reflection(s) Below Theta(Min).          2 Note
PLAT933_ALERT_2_G Number of HKL-OMIT Records in Embedded .res File         28 Note
PLAT941_ALERT_3_G Average HKL Measurement Multiplicity ...........        4.1 Low
PLAT978_ALERT_2_G Number C-C Bonds with Positive Residual Density.          0 Info
PLAT984_ALERT_1_G The Zn-f'=    0.3032 Deviates from the B&C-Value     0.3063 Check
PLAT985_ALERT_1_G The Zn-f"=    1.3627 Deviates from the B&C-Value     1.3615 Check
```

---

1 **ALERT level A** = Most likely a serious problem - resolve or explain
2 **ALERT level B** = A potentially serious problem, consider carefully
93 **ALERT level C** = Check. Ensure it is not caused by an omission or oversight
213 **ALERT level G** = General information/check it is not something unexpected

11 ALERT type 1 CIF construction/syntax error, inconsistent or missing data
130 ALERT type 2 Indicator that the structure model may be wrong or deficient
13 ALERT type 3 Indicator that the structure quality may be low
149 ALERT type 4 Improvement, methodology, query or suggestion
6 ALERT type 5 Informative message, check

It is advisable to attempt to resolve as many as possible of the alerts in all categories. Often the minor alerts point to easily fixed oversights, errors and omissions in your CIF or refinement strategy, so attention to these fine details can be worthwhile. In order to resolve some of the more serious problems it may be necessary to carry out additional measurements or structure refinements. However, the purpose of your study may justify the reported deviations and the more serious of these should normally be commented upon in the discussion or experimental section of a paper or in the "special_details" fields of the CIF. checkCIF was carefully designed to identify outliers and unusual parameters, but every test has its limitations and alerts that are not important in a particular case may appear. Conversely, the absence of alerts does not guarantee there are no aspects of the results needing attention. It is up to the individual to critically assess their own results and, if necessary, seek expert advice.

Publication of your CIF in IUCr journals

A basic structural check has been run on your CIF. These basic checks will be run on all CIFs submitted for publication in IUCr journals (*Acta Crystallographica*, *Journal of Applied Crystallography*, *Journal of Synchrotron Radiation*); however, if you intend to submit to *Acta Crystallographica Section C* or *E* or *IUCrData*, you should make sure that full publication checks are run on the final version of your CIF prior to submission.

Publication of your CIF in other journals

Please refer to the *Notes for Authors* of the relevant journal for any special instructions relating to CIF submission.

PLATON version of 18/05/2022; check.def file version of 17/05/2022

Datablock jd370_sq - ellipsoid plot

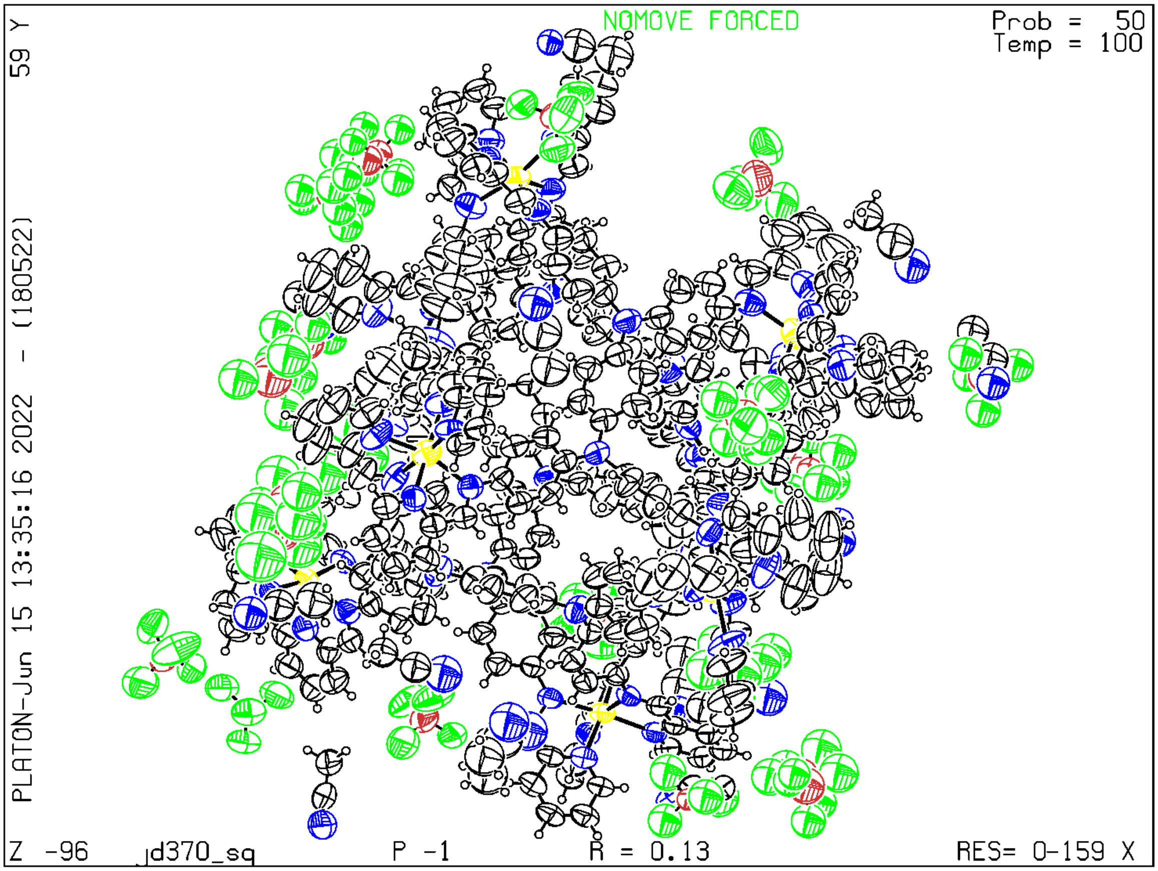

Supplement: Supplementary file 3 — Supporting Information [file ANIE-62-0-s003.pdf]
