# Supplementary material for: Tetramine Aspect Ratio and Flexibility Determine Framework Symmetry for Zn8L6 Self‐Assembled Structures
Source: Angew Chem Int Ed Engl. 2023 Feb 1;62(10):e202217987. doi: 10.1002/anie.202217987 (PMC10946785; doi:10.1002/anie.202217987)

checkCIF/PLATON report

Structure factors have been supplied for datablock(s) jd326_sq

THIS REPORT IS FOR GUIDANCE ONLY. IF USED AS PART OF A REVIEW PROCEDURE FOR PUBLICATION, IT SHOULD NOT REPLACE THE EXPERTISE OF AN EXPERIENCED CRYSTALLOGRAPHIC REFEREE.

No syntax errors found. CIF dictionary Interpreting this report

Datablock: jd326_sq

Bond precision: C-C = 0.0059 A Wavelength=0.68890

Cell:	a=33.94390(6)	b=33.94390(6)	c=32.52560(12)
	alpha=90	beta=90	gamma=120
Temperature:	100 K		

	Calculated	Reported
Volume	32454.84(16)	32454.83(16)
Space group	R -3	R -3 :H
Hall group	-R 3	-R 3
Moiety formula		
Sum formula	C454 H365 N67 O60 Re15 Zn8 [+ solvent]	C454 H365 N67 O64 Re16 Zn8
Mr	11035.26	11285.27
Dx,g cm-3	1.694	1.732
Z	3	3
Mu (mm-1)	4.294	4.550
F000	16208.8	16530.0
F000’	16185.84	
h,k,lmax	52,52,50	52,51,50
Nref	27525	27468
Tmin,Tmax	0.989,0.991	0.978,1.000
Tmin’	0.986	

Correction method= # Reported T Limits: Tmin=0.978 Tmax=1.000
AbsCorr = EMPIRICAL

Data completeness= 0.998 Theta(max)= 32.000

R(reflections)= 0.0536(17741) wR2(reflections)= 0.1794(27468)

S = 1.017 Npar= 1006

The following ALERTS were generated. Each ALERT has the format
test-name_ALERT_alert-type_alert-level.
Click on the hyperlinks for more details of the test.

Alert level A

```
PLAT973_ALERT_2_A Check Calcd Positive Resid. Density on       Re2       2.20 eA-3
```

Author Response: Peaks close to Re atoms due to absorption effects or minor unresolved disorder.

Alert level B

```
PLAT971_ALERT_2_B Check Calcd Resid. Dens.  0.79Ang From Re2             2.54 eA-3
```

Author Response: Peaks close to Re atoms due to absorption effects or minor unresolved disorder.

Alert level C

```
PLAT094_ALERT_2_C Ratio of Maximum / Minimum Residual Density ....       2.76 Report
PLAT244_ALERT_4_C Low    'Solvent' Ueq as Compared to Neighbors of        C1S Check
PLAT260_ALERT_2_C Large Average Ueq of Residue Including      Re1B      0.106 Check
PLAT260_ALERT_2_C Large Average Ueq of Residue Including      Re3'      0.212 Check
PLAT260_ALERT_2_C Large Average Ueq of Residue Including       N1S      0.104 Check
PLAT911_ALERT_3_C Missing FCF Refl Between Thmin & STh/L=    0.600         29 Report
PLAT918_ALERT_3_C Reflection(s) with I(obs) much Smaller I(calc) .          1 Check
PLAT934_ALERT_3_C Number of (Iobs-Icalc)/Sigma(W) > 10 Outliers ..          1 Check
PLAT971_ALERT_2_C Check Calcd Resid. Dens.  0.75Ang From Re2'            2.42 eA-3
```

Author Response: Peaks close to Re atoms due to absorption effects or minor unresolved disorder.

```
PLAT971_ALERT_2_C Check Calcd Resid. Dens.  0.80Ang From Re2'            2.42 eA-3
```

Author Response: Peaks close to Re atoms due to absorption effects or minor unresolved disorder.

```
PLAT971_ALERT_2_C Check Calcd Resid. Dens.  0.73Ang From Zn1             2.19 eA-3
```

Author Response: Peaks close to Re atoms due to absorption effects or minor unresolved disorder.

PLAT971_ALERT_2_C Check Calcd Resid. Dens. 1.00Ang From O12' 2.08 eA-3

Author Response: Peaks close to Re atoms due to absorption effects or minor unresolved disorder.

PLAT971_ALERT_2_C Check Calcd Resid. Dens. 1.01Ang From Re2 1.92 eA-3

Author Response: Peaks close to Re atoms due to absorption effects or minor unresolved disorder.

PLAT971_ALERT_2_C Check Calcd Resid. Dens. 1.21Ang From Re1A 1.91 eA-3

Author Response: Peaks close to Re atoms due to absorption effects or minor unresolved disorder.

PLAT971_ALERT_2_C Check Calcd Resid. Dens. 0.66Ang From Zn2 1.72 eA-3

Author Response: Peaks close to Re atoms due to absorption effects or minor unresolved disorder.

PLAT971_ALERT_2_C Check Calcd Resid. Dens. 0.23Ang From Zn2 1.57 eA-3

Author Response: Peaks close to Re atoms due to absorption effects or minor unresolved disorder.

PLAT971_ALERT_2_C Check Calcd Resid. Dens. 0.42Ang From O9' 1.54 eA-3

Author Response: Peaks close to Re atoms due to absorption effects or minor unresolved disorder.

PLAT971_ALERT_2_C Check Calcd Resid. Dens. 0.50Ang From Re2' 1.51 eA-3

Author Response: Peaks close to Re atoms due to absorption effects or minor unresolved disorder.

PLAT973_ALERT_2_C Check Calcd Positive Resid. Density on Zn1 1.40 eA-3

Author Response: Peaks close to Re atoms due to absorption effects or minor unresolved disorder.

Alert level G

FORMU01_ALERT_1_G There is a discrepancy between the atom counts in the

```
            _chemical_formula_sum and _chemical_formula_moiety. This is
            usually due to the moiety formula being in the wrong format.
            Atom count from _chemical_formula_sum:   C454 H365 N67 O64 Re16 Zn8
            Atom count from _chemical_formula_moiety:
FORMU01_ALERT_2_G  There is a discrepancy between the atom counts in the
            _chemical_formula_sum and the formula from the _atom_site* data.
            Atom count from _chemical_formula_sum:C454 H365 N67 O64 Re16 Zn8
            Atom count from the _atom_site data:  C454. H365. N67. O60.00261 Re15.
CELLZ01_ALERT_1_G Difference between formula and atom_site contents detected.
CELLZ01_ALERT_1_G ALERT: Large difference may be due to a
            symmetry error - see SYMMG tests
           From the CIF: _cell_formula_units_Z    3
           From the CIF: _chemical_formula_sum  C454 H365 N67 O64 Re16 Zn8
           TEST: Compare cell contents of formula and atom_site data

           atom    Z*formula  cif sites diff
           C       1362.00   1362.00   -0.00
           H       1095.00   1095.00   -0.00
           N        201.00    201.00   -0.00
           O        192.00    179.99   12.01
           Re        48.00     45.00    3.00
           Zn        24.00     24.00    0.00
PLAT002_ALERT_2_G Number of Distance or Angle Restraints on AtSite         33 Note
PLAT003_ALERT_2_G Number of Uiso or Uij Restrained non-H Atoms ...        122 Report
PLAT041_ALERT_1_G Calc. and Reported SumFormula    Strings  Differ     Please Check
PLAT042_ALERT_1_G Calc. and Reported MoietyFormula Strings  Differ     Please Check
PLAT051_ALERT_1_G Mu(calc) and Mu(CIF) Ratio Differs from 1.0 by .       5.63 %
PLAT092_ALERT_4_G Check: Wavelength Given is not Cu,Ga,Mo,Ag,In Ka    0.68890 Ang.
PLAT143_ALERT_4_G s.u. on c - Axis Small or Missing .............    0.00012 Ang.
PLAT172_ALERT_4_G The CIF-Embedded .res File Contains DFIX Records          2 Report
PLAT175_ALERT_4_G The CIF-Embedded .res File Contains SAME Records          5 Report
PLAT178_ALERT_4_G The CIF-Embedded .res File Contains SIMU Records          6 Report
PLAT180_ALERT_4_G Check Cell Rounding: # of Values Ending with 0 =          3 Note
PLAT300_ALERT_4_G Atom Site Occupancy of C113       Constrained at     0.6667 Check
PLAT300_ALERT_4_G Atom Site Occupancy of C114       Constrained at     0.6667 Check
PLAT300_ALERT_4_G Atom Site Occupancy of H113       Constrained at     0.6667 Check
PLAT300_ALERT_4_G Atom Site Occupancy of H114       Constrained at     0.6667 Check
PLAT300_ALERT_4_G Atom Site Occupancy of N2S        Constrained at     0.1667 Check
PLAT300_ALERT_4_G Atom Site Occupancy of C3S        Constrained at     0.1667 Check
PLAT300_ALERT_4_G Atom Site Occupancy of C4S        Constrained at     0.1667 Check
PLAT300_ALERT_4_G Atom Site Occupancy of H4S1       Constrained at     0.1667 Check
PLAT300_ALERT_4_G Atom Site Occupancy of H4S2       Constrained at     0.1667 Check
PLAT300_ALERT_4_G Atom Site Occupancy of H4S3       Constrained at     0.1667 Check
PLAT302_ALERT_4_G Anion/Solvent/Minor-Residue Disorder (Resd  4  )       100% Note
PLAT302_ALERT_4_G Anion/Solvent/Minor-Residue Disorder (Resd  5  )       100% Note
PLAT302_ALERT_4_G Anion/Solvent/Minor-Residue Disorder (Resd  6  )       100% Note
PLAT302_ALERT_4_G Anion/Solvent/Minor-Residue Disorder (Resd  7  )       100% Note
PLAT302_ALERT_4_G Anion/Solvent/Minor-Residue Disorder (Resd  8  )       100% Note
PLAT302_ALERT_4_G Anion/Solvent/Minor-Residue Disorder (Resd  9  )       100% Note
PLAT302_ALERT_4_G Anion/Solvent/Minor-Residue Disorder (Resd 10  )       100% Note
PLAT302_ALERT_4_G Anion/Solvent/Minor-Residue Disorder (Resd 11  )       100% Note
PLAT302_ALERT_4_G Anion/Solvent/Minor-Residue Disorder (Resd 13  )       100% Note
PLAT304_ALERT_4_G Non-Integer Number of Atoms in ..... (Resd  5  )       4.77 Check
PLAT304_ALERT_4_G Non-Integer Number of Atoms in ..... (Resd  6  )       2.08 Check
PLAT304_ALERT_4_G Non-Integer Number of Atoms in ..... (Resd  7  )       2.10 Check
PLAT304_ALERT_4_G Non-Integer Number of Atoms in ..... (Resd  8  )       0.83 Check
PLAT304_ALERT_4_G Non-Integer Number of Atoms in ..... (Resd  9  )       0.23 Check
```

```
PLAT304_ALERT_4_G Non-Integer Number of Atoms in ..... (Resd 10  )       1.94 Check
PLAT304_ALERT_4_G Non-Integer Number of Atoms in ..... (Resd 11  )       0.56 Check
PLAT432_ALERT_2_G Short Inter X...Y Contact  O1       ..C42      .       2.91 Ang.
                                            x-y,x,1-z  =     12_556 Check
PLAT432_ALERT_2_G Short Inter X...Y Contact  O1       ..C40      .       2.93 Ang.
                                            x-y,x,1-z  =     12_556 Check
PLAT432_ALERT_2_G Short Inter X...Y Contact  O1       ..C41      .       2.94 Ang.
                                            x-y,x,1-z  =     12_556 Check
PLAT432_ALERT_2_G Short Inter X...Y Contact  O1A      ..C42      .       2.97 Ang.
                                            x-y,x,1-z  =     12_556 Check
PLAT432_ALERT_2_G Short Inter X...Y Contact  O1A      ..C25      .       2.99 Ang.
                                 -2/3+x,-1/3+y,-1/3+z  =      7_444 Check
PLAT432_ALERT_2_G Short Inter X...Y Contact  O3       ..C18      .       2.84 Ang.
                                                x,y,z  =      1_555 Check
PLAT432_ALERT_2_G Short Inter X...Y Contact  O3B      ..C25      .       2.73 Ang.
                                 -2/3+x,-1/3+y,-1/3+z  =      7_444 Check
PLAT432_ALERT_2_G Short Inter X...Y Contact  O4B      ..C18      .       2.63 Ang.
                                                x,y,z  =      1_555 Check
PLAT432_ALERT_2_G Short Inter X...Y Contact  O8'      ..C12      .       2.81 Ang.
                                  2/3+x-y,1/3+x,4/3-z  =     15_556 Check
PLAT432_ALERT_2_G Short Inter X...Y Contact  O8'      ..C11      .       2.85 Ang.
                                  2/3+x-y,1/3+x,4/3-z  =     15_556 Check
PLAT432_ALERT_2_G Short Inter X...Y Contact  O9'      ..C1S      .       2.72 Ang.
                                           -x,1-y,1-z  =     10_566 Check
PLAT432_ALERT_2_G Short Inter X...Y Contact  O9'      ..C2S      .       2.97 Ang.
                                           -x,1-y,1-z  =     10_566 Check
PLAT432_ALERT_2_G Short Inter X...Y Contact  O11      ..C8       .       2.91 Ang.
                                           -x,1-y,1-z  =     10_566 Check
PLAT432_ALERT_2_G Short Inter X...Y Contact  O12'     ..C2S      .       2.74 Ang.
                                           -x,1-y,1-z  =     10_566 Check
PLAT432_ALERT_2_G Short Inter X...Y Contact  C6       ..C106     .       3.18 Ang.
                                                x,y,z  =      1_555 Check
PLAT605_ALERT_4_G Largest Solvent Accessible VOID in the Structure        259 A**3
PLAT720_ALERT_4_G Number of Unusual/Non-Standard Labels .........           6 Note
PLAT789_ALERT_4_G Atoms with Negative _atom_site_disorder_group  #         10 Check
PLAT794_ALERT_5_G Tentative Bond Valency for Zn1       (II)      .       1.71 Info
PLAT794_ALERT_5_G Tentative Bond Valency for Zn2       (II)      .       1.84 Info
PLAT802_ALERT_4_G CIF Input Record(s) with more than 80 Characters          1 Info
PLAT860_ALERT_3_G Number of Least-Squares Restraints ............         785 Note
PLAT869_ALERT_4_G ALERTS Related to the Use of SQUEEZE  Suppressed          ! Info
PLAT883_ALERT_1_G No Info/Value for _atom_sites_solution_primary .     Please Do !
PLAT910_ALERT_3_G Missing # of FCF Reflection(s) Below Theta(Min).          2 Note
PLAT912_ALERT_4_G Missing # of FCF Reflections Above STh/L=  0.600         28 Note
PLAT933_ALERT_2_G Number of HKL-OMIT Records in Embedded .res File         22 Note
PLAT978_ALERT_2_G Number C-C Bonds with Positive Residual Density.          0 Info
PLAT984_ALERT_1_G The Re-f'=   -0.8234 Deviates from the B&C-Value    -0.8001 Check
PLAT984_ALERT_1_G The Zn-f'=    0.3032 Deviates from the B&C-Value     0.3063 Check
PLAT985_ALERT_1_G The Re-f"=    6.9493 Deviates from the B&C-Value     6.9013 Check
PLAT985_ALERT_1_G The Zn-f"=    1.3627 Deviates from the B&C-Value     1.3615 Check
```

---

 1 **ALERT level A** = Most likely a serious problem - resolve or explain
 1 **ALERT level B** = A potentially serious problem, consider carefully
19 **ALERT level C** = Check. Ensure it is not caused by an omission or oversight
73 **ALERT level G** = General information/check it is not something unexpected

11 ALERT type 1 CIF construction/syntax error, inconsistent or missing data

```
37 ALERT type 2 Indicator that the structure model may be wrong or deficient
 5 ALERT type 3 Indicator that the structure quality may be low
39 ALERT type 4 Improvement, methodology, query or suggestion
 2 ALERT type 5 Informative message, check
```

It is advisable to attempt to resolve as many as possible of the alerts in all categories. Often the minor alerts point to easily fixed oversights, errors and omissions in your CIF or refinement strategy, so attention to these fine details can be worthwhile. In order to resolve some of the more serious problems it may be necessary to carry out additional measurements or structure refinements. However, the purpose of your study may justify the reported deviations and the more serious of these should normally be commented upon in the discussion or experimental section of a paper or in the "special_details" fields of the CIF. checkCIF was carefully designed to identify outliers and unusual parameters, but every test has its limitations and alerts that are not important in a particular case may appear. Conversely, the absence of alerts does not guarantee there are no aspects of the results needing attention. It is up to the individual to critically assess their own results and, if necessary, seek expert advice.

Publication of your CIF in IUCr journals

A basic structural check has been run on your CIF. These basic checks will be run on all CIFs submitted for publication in IUCr journals (*Acta Crystallographica*, *Journal of Applied Crystallography*, *Journal of Synchrotron Radiation*); however, if you intend to submit to *Acta Crystallographica Section C* or *E* or *IUCrData*, you should make sure that full publication checks are run on the final version of your CIF prior to submission.

Publication of your CIF in other journals

Please refer to the *Notes for Authors* of the relevant journal for any special instructions relating to CIF submission.

PLATON version of 12/09/2022; check.def file version of 09/08/2022

Datablock jd326_sq - ellipsoid plot

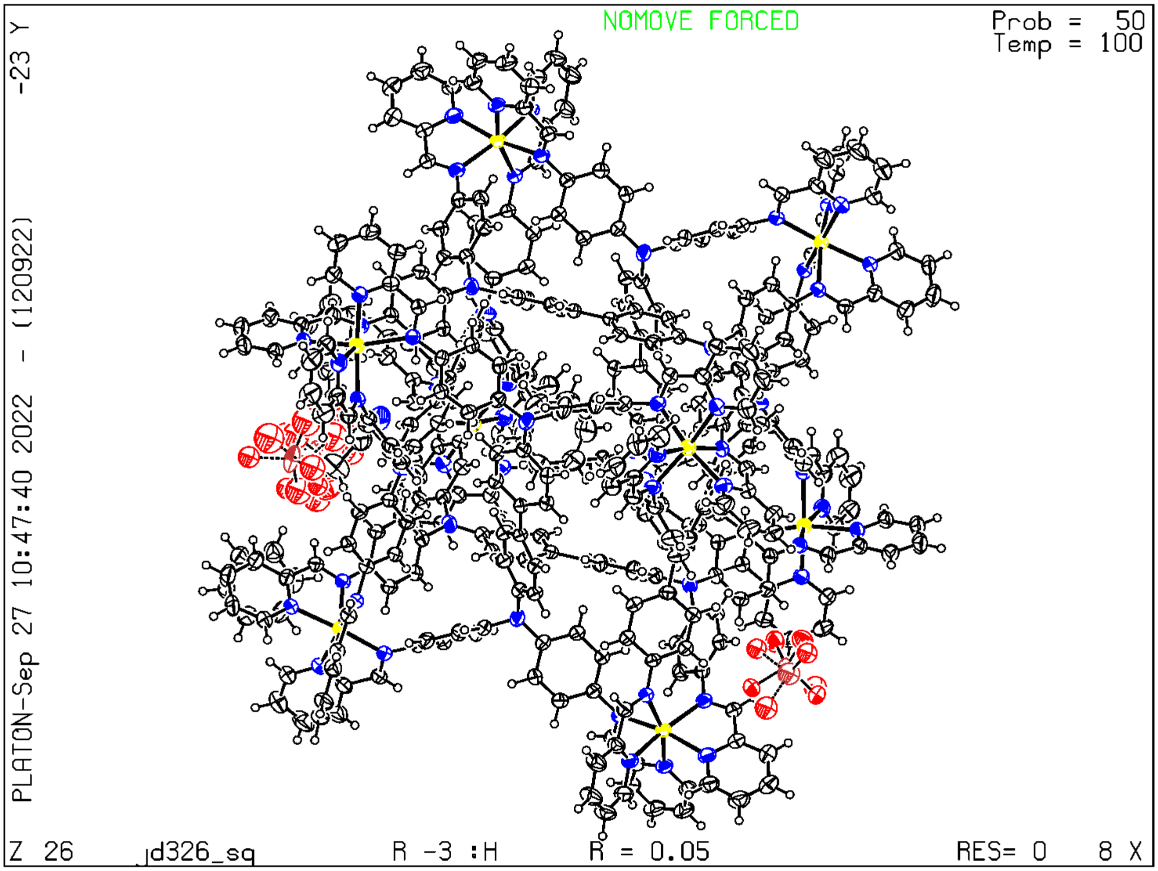

Supplement: Supplementary file 5 — Supporting Information [file ANIE-62-0-s009.pdf]
