# Supplementary material for: Tetramine Aspect Ratio and Flexibility Determine Framework Symmetry for Zn8L6 Self‐Assembled Structures
Source: Angew Chem Int Ed Engl. 2023 Feb 1;62(10):e202217987. doi: 10.1002/anie.202217987 (PMC10946785; doi:10.1002/anie.202217987)

checkCIF/PLATON report

Structure factors have been supplied for datablock(s) jd235_sq

THIS REPORT IS FOR GUIDANCE ONLY. IF USED AS PART OF A REVIEW PROCEDURE FOR PUBLICATION, IT SHOULD NOT REPLACE THE EXPERTISE OF AN EXPERIENCED CRYSTALLOGRAPHIC REFEREE.

No syntax errors found. CIF dictionary Interpreting this report

Datablock: jd235_sq

Bond precision:	C-C = 0.0131 A		Wavelength=0.68890
Cell:	a=24.8925(4)	b=25.7465(4)	c=29.1725(5)
	alpha=70.125(1)	beta=70.055(1)	gamma=79.220(1)
Temperature:	100 K		

	Calculated	Reported
Volume	16474.8(5)	16474.8(5)
Space group	P -1	P -1
Hall group	-P 1	-P 1
Moiety formula		
Sum formula	C422 H285 As10.21 F61.27 N55 Zn8 [+ solvent]	C422 H285 As16 F96 N55 Zn8
Mr	8578.44	9671.71
Dx,g cm-3	0.865	0.975
Z	1	1
Mu (mm-1)	0.781	1.056
F000	4330.4	4834.0
F000'	4335.61	
h,k,lmax	27,28,32	27,28,32
Nref	47343	46290
Tmin,Tmax	0.951,0.979	0.988,1.000
Tmin'	0.949	

Correction method= # Reported T Limits: Tmin=0.988 Tmax=1.000
AbsCorr = EMPIRICAL

Data completeness= 0.978 Theta(max)= 22.502

R(reflections)= 0.1065(26460) wR2(reflections)= 0.3446(46290)

S = 1.158 Npar= 2724

The following ALERTS were generated. Each ALERT has the format
test-name_ALERT_alert-type_alert-level.
Click on the hyperlinks for more details of the test.

Alert level B

```
THETM01_ALERT_3_B  The value of sine(theta_max)/wavelength is less than 0.575
            Calculated sin(theta_max)/wavelength =    0.5555
```

Author Response: Despite the use of a synchrotron radiation and rapid sample handling, few reflections at greater than 0.9 A resolution were observed and the data was trimmed accordingly.

```
PLAT934_ALERT_3_B Number of (Iobs-Icalc)/Sigma(W) > 10 Outliers ..          4 Check
```

Alert level C

```
PLAT029_ALERT_3_C _diffrn_measured_fraction_theta_full value Low .      0.978 Why?
PLAT077_ALERT_4_C Unitcell Contains Non-integer Number of Atoms ..     Please Check
PLAT082_ALERT_2_C High R1 Value ................................       0.11 Report
PLAT084_ALERT_3_C High wR2 Value (i.e. > 0.25) ..................       0.34 Report
PLAT220_ALERT_2_C NonSolvent   Resd 1  C   Ueq(max)/Ueq(min) Range       4.1 Ratio
PLAT241_ALERT_2_C High   'MainMol' Ueq as Compared to Neighbors of       C1B Check
PLAT241_ALERT_2_C High   'MainMol' Ueq as Compared to Neighbors of      C14A Check
PLAT241_ALERT_2_C High   'MainMol' Ueq as Compared to Neighbors of      C14B Check
PLAT241_ALERT_2_C High   'MainMol' Ueq as Compared to Neighbors of      C15B Check
PLAT241_ALERT_2_C High   'MainMol' Ueq as Compared to Neighbors of      C15C Check
PLAT241_ALERT_2_C High   'MainMol' Ueq as Compared to Neighbors of      C16A Check
PLAT241_ALERT_2_C High   'MainMol' Ueq as Compared to Neighbors of      C20A Check
PLAT241_ALERT_2_C High   'MainMol' Ueq as Compared to Neighbors of      C21A Check
PLAT241_ALERT_2_C High   'MainMol' Ueq as Compared to Neighbors of      C23A Check
PLAT241_ALERT_2_C High   'MainMol' Ueq as Compared to Neighbors of      C23C Check
PLAT241_ALERT_2_C High   'MainMol' Ueq as Compared to Neighbors of      C24A Check
PLAT241_ALERT_2_C High   'MainMol' Ueq as Compared to Neighbors of      C27C Check
PLAT241_ALERT_2_C High   'MainMol' Ueq as Compared to Neighbors of      C30A Check
PLAT241_ALERT_2_C High   'MainMol' Ueq as Compared to Neighbors of      C39B Check
PLAT241_ALERT_2_C High   'MainMol' Ueq as Compared to Neighbors of      C44A Check
PLAT241_ALERT_2_C High   'MainMol' Ueq as Compared to Neighbors of      C44C Check
PLAT241_ALERT_2_C High   'MainMol' Ueq as Compared to Neighbors of      C45A Check
PLAT242_ALERT_2_C Low    'MainMol' Ueq as Compared to Neighbors of       N4A Check
PLAT242_ALERT_2_C Low    'MainMol' Ueq as Compared to Neighbors of       N8C Check
PLAT242_ALERT_2_C Low    'MainMol' Ueq as Compared to Neighbors of       C7B Check
PLAT242_ALERT_2_C Low    'MainMol' Ueq as Compared to Neighbors of       C7C Check
PLAT242_ALERT_2_C Low    'MainMol' Ueq as Compared to Neighbors of      C19A Check
PLAT242_ALERT_2_C Low    'MainMol' Ueq as Compared to Neighbors of      C22A Check
PLAT242_ALERT_2_C Low    'MainMol' Ueq as Compared to Neighbors of      C22C Check
PLAT242_ALERT_2_C Low    'MainMol' Ueq as Compared to Neighbors of      C29A Check
PLAT242_ALERT_2_C Low    'MainMol' Ueq as Compared to Neighbors of      C43A Check
PLAT242_ALERT_2_C Low    'MainMol' Ueq as Compared to Neighbors of      C46A Check
PLAT244_ALERT_4_C Low    'Solvent' Ueq as Compared to Neighbors of       As1 Check
PLAT244_ALERT_4_C Low    'Solvent' Ueq as Compared to Neighbors of       As2 Check
PLAT244_ALERT_4_C Low    'Solvent' Ueq as Compared to Neighbors of       C9S Check
PLAT250_ALERT_2_C Large U3/U1 Ratio for Average U(i,j) Tensor ....       2.2 Note
PLAT250_ALERT_2_C Large U3/U1 Ratio for Average U(i,j) Tensor ....       2.8 Note
```

```
PLAT260_ALERT_2_C Large Average Ueq of Residue Including       Zn1      0.116 Check
PLAT260_ALERT_2_C Large Average Ueq of Residue Including       As4      0.111 Check
PLAT260_ALERT_2_C Large Average Ueq of Residue Including       As1      0.181 Check
PLAT260_ALERT_2_C Large Average Ueq of Residue Including       As2      0.123 Check
PLAT260_ALERT_2_C Large Average Ueq of Residue Including       As5      0.240 Check
PLAT260_ALERT_2_C Large Average Ueq of Residue Including      As3'      0.131 Check
PLAT260_ALERT_2_C Large Average Ueq of Residue Including       As6      0.126 Check
PLAT260_ALERT_2_C Large Average Ueq of Residue Including      As6'      0.249 Check
PLAT260_ALERT_2_C Large Average Ueq of Residue Including       As7      0.166 Check
PLAT260_ALERT_2_C Large Average Ueq of Residue Including       N1S      0.109 Check
PLAT260_ALERT_2_C Large Average Ueq of Residue Including       N2S      0.215 Check
PLAT260_ALERT_2_C Large Average Ueq of Residue Including       N3S      0.158 Check
PLAT260_ALERT_2_C Large Average Ueq of Residue Including       N4S      0.184 Check
PLAT260_ALERT_2_C Large Average Ueq of Residue Including       N5S      0.212 Check
PLAT341_ALERT_3_C Low Bond Precision on  C-C Bonds ..............    0.01307 Ang.
PLAT905_ALERT_3_C Negative K value in the Analysis of Variance ...     -2.691 Report
PLAT911_ALERT_3_C Missing FCF Refl Between Thmin & STh/L=    0.556       1054 Report
PLAT918_ALERT_3_C Reflection(s) with I(obs) much Smaller I(calc) .          7 Check
```

Alert level G

```
FORMU01_ALERT_1_G  There is a discrepancy between the atom counts in the
            _chemical_formula_sum and _chemical_formula_moiety. This is
            usually due to the moiety formula being in the wrong format.
            Atom count from _chemical_formula_sum:   C422 H285 As16 F96 N55 Zn8
            Atom count from _chemical_formula_moiety:
FORMU01_ALERT_2_G  There is a discrepancy between the atom counts in the
            _chemical_formula_sum and the formula from the _atom_site* data.
            Atom count from _chemical_formula_sum:C422 H285 As16 F96 N55 Zn8
            Atom count from the _atom_site data:  C422. H285 As10.21200 F61.27199
ABSMU01_ALERT_1_G  Calculation of _exptl_absorpt_correction_mu
                not performed for this radiation type.
CELLZ01_ALERT_1_G Difference between formula and atom_site contents detected.
CELLZ01_ALERT_1_G ALERT: Large difference may be due to a
            symmetry error - see SYMMG tests
           From the CIF: _cell_formula_units_Z    1
           From the CIF: _chemical_formula_sum  C422 H285 As16 F96 N55 Zn8
           TEST: Compare cell contents of formula and atom_site data

           atom    Z*formula  cif sites diff
           C        422.00    422.00   -0.00
           H        285.00    285.00    0.00
           As        16.00     10.21    5.79
           F         96.00     61.27   34.73
           N         55.00     55.00   -0.00
           Zn         8.00      8.00    0.00
PLAT002_ALERT_2_G Number of Distance or Angle Restraints on AtSite        306 Note
PLAT003_ALERT_2_G Number of Uiso or Uij Restrained non-H Atoms ...        302 Report
PLAT041_ALERT_1_G Calc. and Reported SumFormula    Strings  Differ     Please Check
PLAT042_ALERT_1_G Calc. and Reported MoietyFormula Strings  Differ     Please Check
PLAT051_ALERT_1_G Mu(calc) and Mu(CIF) Ratio Differs from 1.0 by .      26.05 %
PLAT092_ALERT_4_G Check: Wavelength Given is not Cu,Ga,Mo,Ag,In Ka    0.68890 Ang.
PLAT154_ALERT_1_G The s.u.'s on the Cell Angles are Equal ..(Note)      0.001 Degree
PLAT171_ALERT_4_G The CIF-Embedded .res File Contains EADP Records          1 Report
PLAT172_ALERT_4_G The CIF-Embedded .res File Contains DFIX Records         20 Report
PLAT174_ALERT_4_G The CIF-Embedded .res File Contains FLAT Records          3 Report
PLAT175_ALERT_4_G The CIF-Embedded .res File Contains SAME Records          4 Report
```

PLAT176_ALERT_4_G The CIF-Embedded .res File Contains SADI Records 12 Report
PLAT178_ALERT_4_G The CIF-Embedded .res File Contains SIMU Records 5 Report
PLAT186_ALERT_4_G The CIF-Embedded .res File Contains ISOR Records 4 Report
PLAT187_ALERT_4_G The CIF-Embedded .res File Contains RIGU Records 1 Report
PLAT300_ALERT_4_G Atom Site Occupancy of As4 Constrained at 0.85 Check
PLAT300_ALERT_4_G Atom Site Occupancy of F19 Constrained at 0.45 Check
PLAT300_ALERT_4_G Atom Site Occupancy of F19' Constrained at 0.4 Check
PLAT300_ALERT_4_G Atom Site Occupancy of F20 Constrained at 0.45 Check
PLAT300_ALERT_4_G Atom Site Occupancy of F20' Constrained at 0.4 Check
PLAT300_ALERT_4_G Atom Site Occupancy of F21 Constrained at 0.45 Check
PLAT300_ALERT_4_G Atom Site Occupancy of F21' Constrained at 0.4 Check
PLAT300_ALERT_4_G Atom Site Occupancy of F22 Constrained at 0.45 Check
PLAT300_ALERT_4_G Atom Site Occupancy of F22' Constrained at 0.4 Check
PLAT300_ALERT_4_G Atom Site Occupancy of F23 Constrained at 0.45 Check
PLAT300_ALERT_4_G Atom Site Occupancy of F23' Constrained at 0.4 Check
PLAT300_ALERT_4_G Atom Site Occupancy of F24 Constrained at 0.45 Check
PLAT300_ALERT_4_G Atom Site Occupancy of F24' Constrained at 0.4 Check
PLAT300_ALERT_4_G Atom Site Occupancy of N2S Constrained at 0.5 Check
PLAT300_ALERT_4_G Atom Site Occupancy of C3S Constrained at 0.5 Check
PLAT300_ALERT_4_G Atom Site Occupancy of C4S Constrained at 0.5 Check
PLAT300_ALERT_4_G Atom Site Occupancy of H4S1 Constrained at 0.5 Check
PLAT300_ALERT_4_G Atom Site Occupancy of H4S2 Constrained at 0.5 Check
PLAT300_ALERT_4_G Atom Site Occupancy of H4S3 Constrained at 0.5 Check
PLAT300_ALERT_4_G Atom Site Occupancy of N3S Constrained at 0.5 Check
PLAT300_ALERT_4_G Atom Site Occupancy of C5S Constrained at 0.5 Check
PLAT300_ALERT_4_G Atom Site Occupancy of C6S Constrained at 0.5 Check
PLAT300_ALERT_4_G Atom Site Occupancy of H6SA Constrained at 0.5 Check
PLAT300_ALERT_4_G Atom Site Occupancy of H6SB Constrained at 0.5 Check
PLAT300_ALERT_4_G Atom Site Occupancy of H6SC Constrained at 0.5 Check
PLAT300_ALERT_4_G Atom Site Occupancy of N4S Constrained at 0.5 Check
PLAT300_ALERT_4_G Atom Site Occupancy of C7S Constrained at 0.5 Check
PLAT300_ALERT_4_G Atom Site Occupancy of C8S Constrained at 0.5 Check
PLAT300_ALERT_4_G Atom Site Occupancy of H8SA Constrained at 0.5 Check
PLAT300_ALERT_4_G Atom Site Occupancy of H8SB Constrained at 0.5 Check
PLAT300_ALERT_4_G Atom Site Occupancy of H8SC Constrained at 0.5 Check
PLAT301_ALERT_3_G Main Residue Disorder ....(Resd 1) 7% Note
PLAT302_ALERT_4_G Anion/Solvent/Minor-Residue Disorder (Resd 2) 100% Note
PLAT302_ALERT_4_G Anion/Solvent/Minor-Residue Disorder (Resd 5) 100% Note
PLAT302_ALERT_4_G Anion/Solvent/Minor-Residue Disorder (Resd 6) 100% Note
PLAT302_ALERT_4_G Anion/Solvent/Minor-Residue Disorder (Resd 7) 100% Note
PLAT302_ALERT_4_G Anion/Solvent/Minor-Residue Disorder (Resd 8) 100% Note
PLAT302_ALERT_4_G Anion/Solvent/Minor-Residue Disorder (Resd 9) 100% Note
PLAT302_ALERT_4_G Anion/Solvent/Minor-Residue Disorder (Resd 10) 100% Note
PLAT302_ALERT_4_G Anion/Solvent/Minor-Residue Disorder (Resd 11) 100% Note
PLAT302_ALERT_4_G Anion/Solvent/Minor-Residue Disorder (Resd 12) 100% Note
PLAT302_ALERT_4_G Anion/Solvent/Minor-Residue Disorder (Resd 13) 100% Note
PLAT302_ALERT_4_G Anion/Solvent/Minor-Residue Disorder (Resd 14) 100% Note
PLAT302_ALERT_4_G Anion/Solvent/Minor-Residue Disorder (Resd 16) 100% Note
PLAT304_ALERT_4_G Non-Integer Number of Atoms in ..... (Resd 2) 5.95 Check
PLAT304_ALERT_4_G Non-Integer Number of Atoms in ..... (Resd 5) 4.17 Check
PLAT304_ALERT_4_G Non-Integer Number of Atoms in ..... (Resd 6) 2.92 Check
PLAT304_ALERT_4_G Non-Integer Number of Atoms in ..... (Resd 7) 1.20 Check
PLAT304_ALERT_4_G Non-Integer Number of Atoms in ..... (Resd 8) 2.63 Check
PLAT304_ALERT_4_G Non-Integer Number of Atoms in ..... (Resd 9) 1.51 Check
PLAT304_ALERT_4_G Non-Integer Number of Atoms in ..... (Resd 10) 3.35 Check
PLAT304_ALERT_4_G Non-Integer Number of Atoms in ..... (Resd 11) 4.66 Check
PLAT304_ALERT_4_G Non-Integer Number of Atoms in ..... (Resd 16) 1.34 Check

```
PLAT413_ALERT_2_G Short Inter XH3 .. XHn     H37C     ..H2XA     .       1.80 Ang.
                                                x,1+y,z  =      1_565 Check
PLAT432_ALERT_2_G Short Inter X...Y Contact  As3'     ..C26B     .       3.28 Ang.
                                            1-x,1-y,1-z  =      2_666 Check
PLAT432_ALERT_2_G Short Inter X...Y Contact  F4       ..C25B     .       2.91 Ang.
                                                  x,y,z  =      1_555 Check
PLAT432_ALERT_2_G Short Inter X...Y Contact  F5       ..C2X      .       2.61 Ang.
                                             1-x,-y,1-z  =      2_656 Check
PLAT432_ALERT_2_G Short Inter X...Y Contact  F13      ..C15B     .       2.84 Ang.
                                             -x,1-y,1-z  =      2_566 Check
PLAT432_ALERT_2_G Short Inter X...Y Contact  F13      ..C14B     .       2.96 Ang.
                                             -x,1-y,1-z  =      2_566 Check
PLAT432_ALERT_2_G Short Inter X...Y Contact  F13      ..C16B     .       2.96 Ang.
                                             -x,1-y,1-z  =      2_566 Check
PLAT432_ALERT_2_G Short Inter X...Y Contact  F13'     ..C25B     .       2.36 Ang.
                                            1-x,1-y,1-z  =      2_666 Check
PLAT432_ALERT_2_G Short Inter X...Y Contact  F13'     ..C26B     .       2.60 Ang.
                                            1-x,1-y,1-z  =      2_666 Check
PLAT432_ALERT_2_G Short Inter X...Y Contact  F14      ..C41C     .       2.77 Ang.
                                                  x,y,z  =      1_555 Check
PLAT432_ALERT_2_G Short Inter X...Y Contact  F14'     ..C13A     .       2.45 Ang.
                                             -x,1-y,1-z  =      2_566 Check
PLAT432_ALERT_2_G Short Inter X...Y Contact  F14'     ..C14A     .       2.96 Ang.
                                             -x,1-y,1-z  =      2_566 Check
PLAT432_ALERT_2_G Short Inter X...Y Contact  F15      ..C13A     .       2.88 Ang.
                                             -x,1-y,1-z  =      2_566 Check
PLAT432_ALERT_2_G Short Inter X...Y Contact  F15'     ..C14A     .       2.60 Ang.
                                             -x,1-y,1-z  =      2_566 Check
PLAT432_ALERT_2_G Short Inter X...Y Contact  F15'     ..C27C     .       2.85 Ang.
                                             -x,2-y,1-z  =      2_576 Check
PLAT432_ALERT_2_G Short Inter X...Y Contact  F15'     ..C13A     .       2.85 Ang.
                                             -x,1-y,1-z  =      2_566 Check
PLAT432_ALERT_2_G Short Inter X...Y Contact  F15'     ..C26C     .       2.90 Ang.
                                             -x,2-y,1-z  =      2_576 Check
PLAT432_ALERT_2_G Short Inter X...Y Contact  F16      ..C25B     .       2.74 Ang.
                                            1-x,1-y,1-z  =      2_666 Check
PLAT432_ALERT_2_G Short Inter X...Y Contact  F16'     ..C25B     .       2.88 Ang.
                                            1-x,1-y,1-z  =      2_666 Check
PLAT432_ALERT_2_G Short Inter X...Y Contact  F16'     ..C26B     .       2.89 Ang.
                                            1-x,1-y,1-z  =      2_666 Check
PLAT432_ALERT_2_G Short Inter X...Y Contact  F18      ..C14B     .       2.69 Ang.
                                             -x,1-y,1-z  =      2_566 Check
PLAT432_ALERT_2_G Short Inter X...Y Contact  F18      ..C42C     .       2.73 Ang.
                                                  x,y,z  =      1_555 Check
PLAT432_ALERT_2_G Short Inter X...Y Contact  F18'     ..C26B     .       2.69 Ang.
                                            1-x,1-y,1-z  =      2_666 Check
PLAT432_ALERT_2_G Short Inter X...Y Contact  F21'     ..C6S      .       2.69 Ang.
                                                  x,y,z  =      1_555 Check
PLAT432_ALERT_2_G Short Inter X...Y Contact  F31      ..C26A     .       2.86 Ang.
                                                  x,y,z  =      1_555 Check
PLAT434_ALERT_2_G Short Inter HL..HL Contact F3       ..F7       .       2.75 Ang.
                                                  x,y,z  =      1_555 Check
PLAT606_ALERT_4_G Solvent Accessible VOID(S) in Structure ........         ! Info
PLAT720_ALERT_4_G Number of Unusual/Non-Standard Labels ..........        15 Note
PLAT790_ALERT_4_G Centre of Gravity not Within Unit Cell: Resd.  #         2 Note
              As0.85 F5.10
PLAT790_ALERT_4_G Centre of Gravity not Within Unit Cell: Resd.  #         4 Note
```

```
              As F6
PLAT790_ALERT_4_G Centre of Gravity not Within Unit Cell: Resd.  #         10 Note
              As F6
PLAT790_ALERT_4_G Centre of Gravity not Within Unit Cell: Resd.  #         12 Note
              C2 H3 N
PLAT790_ALERT_4_G Centre of Gravity not Within Unit Cell: Resd.  #         15 Note
              C2 H3 N
PLAT802_ALERT_4_G CIF Input Record(s) with more than 80 Characters          1 Info
PLAT860_ALERT_3_G Number of Least-Squares Restraints .............       5033 Note
PLAT869_ALERT_4_G ALERTS Related to the Use of SQUEEZE  Suppressed          ! Info
PLAT883_ALERT_1_G No Info/Value for _atom_sites_solution_primary .     Please Do !
PLAT933_ALERT_2_G Number of HKL-OMIT Records in Embedded .res File         18 Note
PLAT941_ALERT_3_G Average HKL Measurement Multiplicity ...........        2.9 Low
PLAT978_ALERT_2_G Number C-C Bonds with Positive Residual Density.          0 Info
PLAT984_ALERT_1_G The As-f'=    0.1095 Deviates from the B&C-Value     0.1041 Check
PLAT984_ALERT_1_G The Zn-f'=    0.3032 Deviates from the B&C-Value     0.3063 Check
PLAT985_ALERT_1_G The Zn-f"=    1.3627 Deviates from the B&C-Value     1.3615 Check
```

```
   0 ALERT level A = Most likely a serious problem - resolve or explain
   2 ALERT level B = A potentially serious problem, consider carefully
  55 ALERT level C = Check. Ensure it is not caused by an omission or oversight
 116 ALERT level G = General information/check it is not something unexpected

  12 ALERT type 1 CIF construction/syntax error, inconsistent or missing data
  76 ALERT type 2 Indicator that the structure model may be wrong or deficient
  11 ALERT type 3 Indicator that the structure quality may be low
  74 ALERT type 4 Improvement, methodology, query or suggestion
   0 ALERT type 5 Informative message, check
```

Validation response form

Please find below a validation response form (VRF) that can be filled in and pasted into your CIF.

```
# start Validation Reply Form
_vrf_PLAT934_jd235_sq
;
PROBLEM: Number of (Iobs-Icalc)/Sigma(W) > 10 Outliers ..          4 Check
RESPONSE: ...
;
# end Validation Reply Form
```

It is advisable to attempt to resolve as many as possible of the alerts in all categories. Often the minor alerts point to easily fixed oversights, errors and omissions in your CIF or refinement strategy, so attention to these fine details can be worthwhile. In order to resolve some of the more serious problems it may be necessary to carry out additional measurements or structure refinements. However, the purpose of your study may justify the reported deviations and the more serious of these should normally be commented upon in the discussion or experimental section of a paper or in the "special_details" fields of the CIF. checkCIF was carefully designed to identify outliers and unusual parameters, but every test has its limitations and alerts that are not important in a particular case may appear. Conversely, the absence of alerts does not guarantee there are no aspects of the results needing attention. It is up to the individual to critically assess their own results and, if necessary, seek expert advice.

Publication of your CIF in IUCr journals

A basic structural check has been run on your CIF. These basic checks will be run on all CIFs submitted for publication in IUCr journals (*Acta Crystallographica*, *Journal of Applied Crystallography*, *Journal of Synchrotron Radiation*); however, if you intend to submit to *Acta Crystallographica Section C* or *E* or *IUCrData*, you should make sure that full publication checks are run on the final version of your CIF prior to submission.

Publication of your CIF in other journals

Please refer to the *Notes for Authors* of the relevant journal for any special instructions relating to CIF submission.

PLATON version of 18/05/2022; check.def file version of 17/05/2022

Datablock jd235_sq - ellipsoid plot

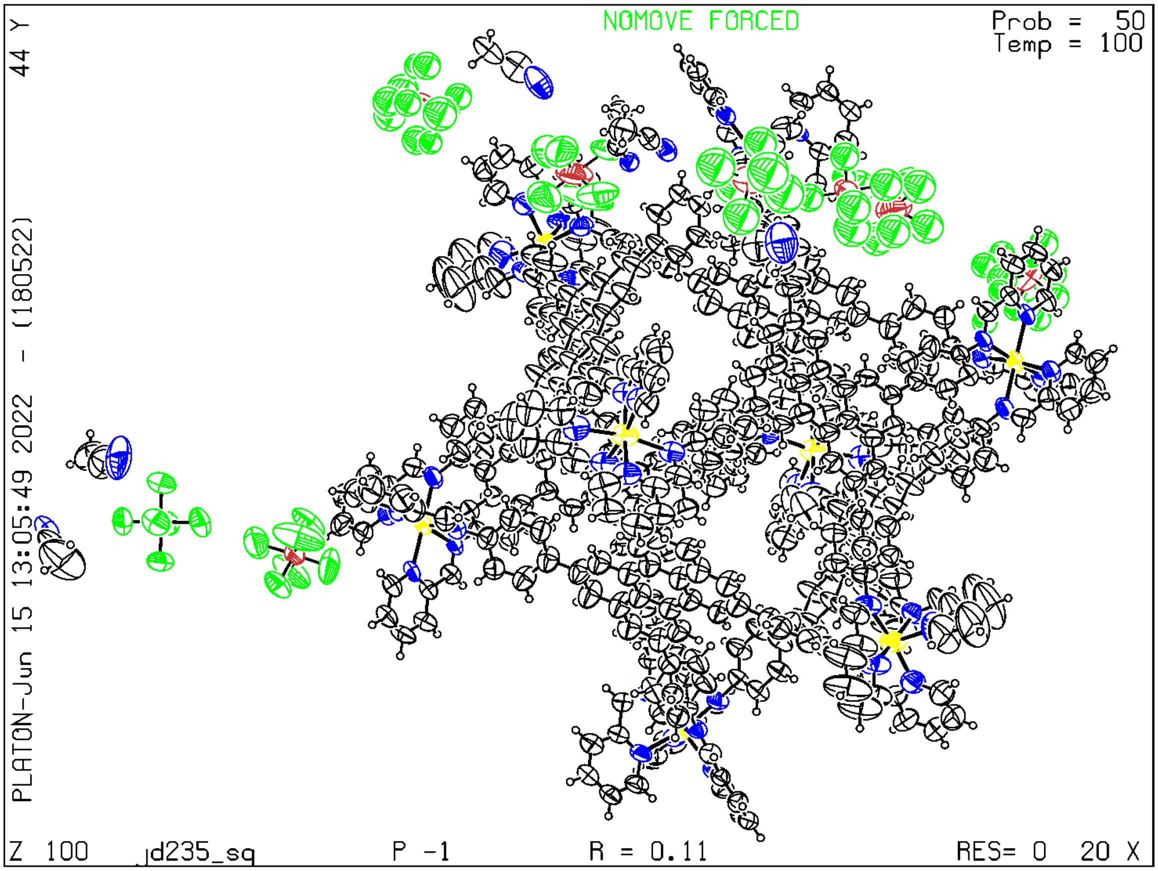

Supplement: Supplementary file 7 — Supporting Information [file ANIE-62-0-s002.pdf]
