# Supplementary material for: Tetramine Aspect Ratio and Flexibility Determine Framework Symmetry for Zn8L6 Self‐Assembled Structures
Source: Angew Chem Int Ed Engl. 2023 Feb 1;62(10):e202217987. doi: 10.1002/anie.202217987 (PMC10946785; doi:10.1002/anie.202217987)

checkCIF/PLATON report

Structure factors have been supplied for datablock(s) jd354_sq

THIS REPORT IS FOR GUIDANCE ONLY. IF USED AS PART OF A REVIEW PROCEDURE FOR PUBLICATION, IT SHOULD NOT REPLACE THE EXPERTISE OF AN EXPERIENCED CRYSTALLOGRAPHIC REFEREE.

No syntax errors found. CIF dictionary Interpreting this report

Datablock: jd354_sq

```
Bond precision:   C-C = 0.0196 A          Wavelength=0.68890

Cell:             a=25.9759(4)     b=48.2777(6)        c=36.8773(4)
                  alpha=90         beta=110.331(1)     gamma=90
Temperature:      100 K
```

	Calculated	Reported
Volume	43365.1(10)	43365.1(10)
Space group	P 21/c	P 21/c
Hall group	-P 2ybc	-P 2ybc
Moiety formula		
Sum formula	C363.45 H939 F106.36 N208 O106.36 S35.45 Zn32 [+ solvent]	C348 H240 F48 N52 O48 S16 Zn8
Mr	27186.95	7865.83
Dx,g cm-3	1.041	1.205
Z	1	4
Mu (mm-1)	0.498	0.546
F000	13911.0	16016.0
F000'	13930.10	
h,k,lmax	23,43,33	23,43,33
Nref	34132	33662
Tmin,Tmax	0.974,0.978	0.991,1.000
Tmin'	0.973	

```
Correction method= # Reported T Limits: Tmin=0.991 Tmax=1.000
AbsCorr = EMPIRICAL

Data completeness= 0.986          Theta(max)= 18.248
```

R(reflections)= 0.1208(10495)   wR2(reflections)= 0.3444(33662)

S = 0.999   Npar= 4230

The following ALERTS were generated. Each ALERT has the format
test-name_ALERT_alert-type_alert-level.
Click on the hyperlinks for more details of the test.

Alert level A

THETM01_ALERT_3_A The value of sine(theta_max)/wavelength is less than 0.550
Calculated sin(theta_max)/wavelength = 0.4545

Author Response: The crystals were very weakly diffracting and few reflections at greater than 1.1 A resolution were observed hence the data was trimmed accordingly.

Alert level B

PLAT026_ALERT_3_B Ratio Observed / Unique Reflections (too) Low .. 31% Check

Author Response: There was a significant drop-off in diffraction intensity after around 1.5 A resolution resulting in a low ratio of observed/unique reflections.

PLAT088_ALERT_3_B Poor Data / Parameter Ratio .... 7.96 Note

Author Response: The low data to parameter ratio results from the limited resolution of the data. Modelling of disorder within the structure increased the number of paramters, exacerbating the already poor data to parameter ratio.

PLAT341_ALERT_3_B Low Bond Precision on C-C Bonds .... 0.01964 Ang.

Author Response: The low bond precision arises from the limited resolution of the data and thermal motion in some areas of the structure.

PLAT411_ALERT_2_B Short Inter H...H Contact H28B ..H39C . 1.90 Ang.
-1+x,1/2-y,-1/2+z = 4_465 Check

Author Response: Short contact between pyridyl rings with a high degree of thermal motion.

PLAT934_ALERT_3_B Number of (Iobs-Icalc)/Sigma(W) > 10 Outliers .. 4 Check

---

Alert level C

PLAT077_ALERT_4_C Unitcell Contains Non-integer Number of Atoms .. Please Check
PLAT082_ALERT_2_C High R1 Value .... 0.12 Report

```
PLAT084_ALERT_3_C High wR2 Value (i.e. > 0.25) ...................       0.34 Report
PLAT094_ALERT_2_C Ratio of Maximum / Minimum Residual Density ....       2.01 Report
PLAT202_ALERT_3_C Isotropic non-H Atoms in Anion/Solvent .........         34 Check
              F1H     F2H     F3H     O1H     O2H     O3H                 etc.
PLAT241_ALERT_2_C High   'MainMol' Ueq as Compared to Neighbors of        C2E Check
PLAT241_ALERT_2_C High   'MainMol' Ueq as Compared to Neighbors of        C4B Check
PLAT241_ALERT_2_C High   'MainMol' Ueq as Compared to Neighbors of        C4C Check
PLAT241_ALERT_2_C High   'MainMol' Ueq as Compared to Neighbors of       C11E Check
PLAT241_ALERT_2_C High   'MainMol' Ueq as Compared to Neighbors of       C14A Check
PLAT241_ALERT_2_C High   'MainMol' Ueq as Compared to Neighbors of       C15A Check
PLAT241_ALERT_2_C High   'MainMol' Ueq as Compared to Neighbors of       C15F Check
PLAT241_ALERT_2_C High   'MainMol' Ueq as Compared to Neighbors of       C16C Check
PLAT241_ALERT_2_C High   'MainMol' Ueq as Compared to Neighbors of       C16E Check
PLAT241_ALERT_2_C High   'MainMol' Ueq as Compared to Neighbors of       C26E Check
PLAT241_ALERT_2_C High   'MainMol' Ueq as Compared to Neighbors of       C26F Check
PLAT241_ALERT_2_C High   'MainMol' Ueq as Compared to Neighbors of       C27C Check
PLAT241_ALERT_2_C High   'MainMol' Ueq as Compared to Neighbors of       C28D Check
PLAT241_ALERT_2_C High   'MainMol' Ueq as Compared to Neighbors of       C28E Check
PLAT241_ALERT_2_C High   'MainMol' Ueq as Compared to Neighbors of       C30B Check
PLAT241_ALERT_2_C High   'MainMol' Ueq as Compared to Neighbors of       C30E Check
PLAT241_ALERT_2_C High   'MainMol' Ueq as Compared to Neighbors of       C32A Check
PLAT241_ALERT_2_C High   'MainMol' Ueq as Compared to Neighbors of       C32B Check
PLAT241_ALERT_2_C High   'MainMol' Ueq as Compared to Neighbors of       C33E Check
PLAT241_ALERT_2_C High   'MainMol' Ueq as Compared to Neighbors of       C33F Check
PLAT241_ALERT_2_C High   'MainMol' Ueq as Compared to Neighbors of       C35A Check
PLAT241_ALERT_2_C High   'MainMol' Ueq as Compared to Neighbors of       C35E Check
PLAT241_ALERT_2_C High   'MainMol' Ueq as Compared to Neighbors of       C36A Check
PLAT241_ALERT_2_C High   'MainMol' Ueq as Compared to Neighbors of       C36D Check
PLAT241_ALERT_2_C High   'MainMol' Ueq as Compared to Neighbors of       C38A Check
PLAT241_ALERT_2_C High   'MainMol' Ueq as Compared to Neighbors of       C38B Check
PLAT241_ALERT_2_C High   'MainMol' Ueq as Compared to Neighbors of       C39B Check
PLAT241_ALERT_2_C High   'MainMol' Ueq as Compared to Neighbors of       C39C Check
PLAT241_ALERT_2_C High   'MainMol' Ueq as Compared to Neighbors of       C39E Check
PLAT241_ALERT_2_C High   'MainMol' Ueq as Compared to Neighbors of       C39F Check
PLAT241_ALERT_2_C High   'MainMol' Ueq as Compared to Neighbors of       C40E Check
PLAT241_ALERT_2_C High   'MainMol' Ueq as Compared to Neighbors of       C42C Check
PLAT241_ALERT_2_C High   'MainMol' Ueq as Compared to Neighbors of       C44C Check
PLAT241_ALERT_2_C High   'MainMol' Ueq as Compared to Neighbors of       C44E Check
PLAT241_ALERT_2_C High   'MainMol' Ueq as Compared to Neighbors of       C44F Check
PLAT241_ALERT_2_C High   'MainMol' Ueq as Compared to Neighbors of       C45A Check
PLAT241_ALERT_2_C High   'MainMol' Ueq as Compared to Neighbors of       C45C Check
PLAT241_ALERT_2_C High   'MainMol' Ueq as Compared to Neighbors of       C48E Check
PLAT242_ALERT_2_C Low    'MainMol' Ueq as Compared to Neighbors of        Zn3 Check
PLAT242_ALERT_2_C Low    'MainMol' Ueq as Compared to Neighbors of        N4F Check
PLAT242_ALERT_2_C Low    'MainMol' Ueq as Compared to Neighbors of        N5B Check
PLAT242_ALERT_2_C Low    'MainMol' Ueq as Compared to Neighbors of        N6A Check
PLAT242_ALERT_2_C Low    'MainMol' Ueq as Compared to Neighbors of        N6B Check
PLAT242_ALERT_2_C Low    'MainMol' Ueq as Compared to Neighbors of        N6E Check
PLAT242_ALERT_2_C Low    'MainMol' Ueq as Compared to Neighbors of        C1C Check
PLAT242_ALERT_2_C Low    'MainMol' Ueq as Compared to Neighbors of        C1E Check
PLAT242_ALERT_2_C Low    'MainMol' Ueq as Compared to Neighbors of       C29E Check
PLAT242_ALERT_2_C Low    'MainMol' Ueq as Compared to Neighbors of       C31A Check
PLAT242_ALERT_2_C Low    'MainMol' Ueq as Compared to Neighbors of       C31B Check
PLAT242_ALERT_2_C Low    'MainMol' Ueq as Compared to Neighbors of       C31D Check
PLAT242_ALERT_2_C Low    'MainMol' Ueq as Compared to Neighbors of       C34E Check
PLAT242_ALERT_2_C Low    'MainMol' Ueq as Compared to Neighbors of       C37C Check
PLAT242_ALERT_2_C Low    'MainMol' Ueq as Compared to Neighbors of       C41E Check
```

```
PLAT242_ALERT_2_C Low    'MainMol' Ueq as Compared to Neighbors of       C43F Check
PLAT242_ALERT_2_C Low    'MainMol' Ueq as Compared to Neighbors of       C46C Check
PLAT242_ALERT_2_C Low    'MainMol' Ueq as Compared to Neighbors of       C53E Check
PLAT260_ALERT_2_C Large Average Ueq of Residue Including       Zn1      0.165 Check
PLAT260_ALERT_2_C Large Average Ueq of Residue Including       S1G      0.224 Check
PLAT260_ALERT_2_C Large Average Ueq of Residue Including       S1H      0.189 Check
PLAT260_ALERT_2_C Large Average Ueq of Residue Including       S1L      0.205 Check
PLAT260_ALERT_2_C Large Average Ueq of Residue Including       S1M      0.148 Check
PLAT260_ALERT_2_C Large Average Ueq of Residue Including       S1N      0.279 Check
PLAT260_ALERT_2_C Large Average Ueq of Residue Including       S1P      0.238 Check
PLAT260_ALERT_2_C Large Average Ueq of Residue Including       S1U      0.283 Check
PLAT260_ALERT_2_C Large Average Ueq of Residue Including      S1HH      0.180 Check
PLAT260_ALERT_2_C Large Average Ueq of Residue Including       S1I      0.275 Check
PLAT260_ALERT_2_C Large Average Ueq of Residue Including       S1K      0.252 Check
PLAT260_ALERT_2_C Large Average Ueq of Residue Including       S1O      0.187 Check
PLAT260_ALERT_2_C Large Average Ueq of Residue Including       S1Q      0.254 Check
PLAT260_ALERT_2_C Large Average Ueq of Residue Including       S1T      0.177 Check
PLAT260_ALERT_2_C Large Average Ueq of Residue Including      S1II      0.253 Check
PLAT260_ALERT_2_C Large Average Ueq of Residue Including      S1KK      0.251 Check
PLAT260_ALERT_2_C Large Average Ueq of Residue Including      S1OO      0.183 Check
PLAT260_ALERT_2_C Large Average Ueq of Residue Including      S1UU      0.297 Check
PLAT411_ALERT_2_C Short Inter H...H Contact  H38A     ..H40E     .        2.01 Ang.
                                    1-x,-1/2+y,3/2-z  =      2_646 Check
```

Author Response: Short contact between pyridyl rings with a high degree of thermal motion.

```
PLAT733_ALERT_1_C Torsion Calc     0.3(19), Rep      0.1(4) ......       4.75 s.u.-R
             C25B-C26B-C27B-C28B  1_555  1_555  1_555  1_555      #  210 Check
PLAT733_ALERT_1_C Torsion Calc     0.2(18), Rep     -0.1(4) ......       4.50 s.u.-R
             C25B-N5B -C29B-C28B  1_555  1_555  1_555  1_555      #  212 Check
PLAT733_ALERT_1_C Torsion Calc  -179.7(13), Rep   -179.9(3) ......       4.33 s.u.-R
             C27B-C28B-C29B-C30B  1_555  1_555  1_555  1_555      #  217 Check
PLAT733_ALERT_1_C Torsion Calc     0.2(19), Rep     -0.1(4) ......       4.75 s.u.-R
             C37B-N7B -C41B-C40B  1_555  1_555  1_555  1_555      #  241 Check
PLAT733_ALERT_1_C Torsion Calc  -179.8(12), Rep    180.0(2) ......       6.00 s.u.-R
             C37B-N7B -C41B-C42B  1_555  1_555  1_555  1_555      #  243 Check
PLAT733_ALERT_1_C Torsion Calc   179.9(13), Rep   -179.9(3) ......       4.33 s.u.-R
             C39B-C40B-C41B-C42B  1_555  1_555  1_555  1_555      #  246 Check
PLAT733_ALERT_1_C Torsion Calc    -0.2(18), Rep     -0.1(4) ......       4.50 s.u.-R
             C25C-N5C -C29C-C28C  1_555  1_555  1_555  1_555      #  360 Check
PLAT733_ALERT_1_C Torsion Calc   179.8(11), Rep   -180.0(2) ......       5.50 s.u.-R
             C25C-N5C -C29C-C30C  1_555  1_555  1_555  1_555      #  362 Check
PLAT733_ALERT_1_C Torsion Calc  -179.9(13), Rep   -180.0(3) ......       4.33 s.u.-R
             C27C-C28C-C29C-C30C  1_555  1_555  1_555  1_555      #  365 Check
PLAT733_ALERT_1_C Torsion Calc    -0.4(18), Rep     -0.1(4) ......       4.50 s.u.-R
             C37C-N7C -C41C-C40C  1_555  1_555  1_555  1_555      #  393 Check
PLAT733_ALERT_1_C Torsion Calc   179.9(15), Rep    179.9(3) ......       5.00 s.u.-R
             C39C-C40C-C41C-C42C  1_555  1_555  1_555  1_555      #  398 Check
PLAT733_ALERT_1_C Torsion Calc   179.9(17), Rep    179.9(3) ......       5.67 s.u.-R
             C27D-C28D-C29D-C30D  1_555  1_555  1_555  1_555      #  531 Check
PLAT733_ALERT_1_C Torsion Calc    -0.5(17), Rep      0.0(4) ......       4.25 s.u.-R
             C25E-N5E -C29E-C28E  1_555  1_555  1_555  1_555      #  674 Check
PLAT733_ALERT_1_C Torsion Calc   179.9(12), Rep   -180.0(2) ......       6.00 s.u.-R
             C25E-N5E -C29E-C30E  1_555  1_555  1_555  1_555      #  676 Check
PLAT733_ALERT_1_C Torsion Calc  -179.9(15), Rep   -180.0(3) ......       5.00 s.u.-R
             C27E-C28E-C29E-C30E  1_555  1_555  1_555  1_555      #  679 Check
```

```
PLAT733_ALERT_1_C Torsion Calc    -0.2(17), Rep     -0.1(4) ......        4.25 s.u.-R
              C37F-C38F-C39F-C40F  1_555  1_555  1_555  1_555      #  849 Check
PLAT733_ALERT_1_C Torsion Calc  -179.9(11), Rep   -179.9(2) ......        5.50 s.u.-R
              C37F-N7F -C41F-C42F  1_555  1_555  1_555  1_555      #  853 Check
PLAT911_ALERT_3_C Missing FCF Refl Between Thmin & STh/L=    0.455        467 Report
PLAT918_ALERT_3_C Reflection(s) with I(obs) much Smaller I(calc) .          7 Check
```

Alert level G

```
FORMU01_ALERT_1_G  There is a discrepancy between the atom counts in the
            _chemical_formula_sum and _chemical_formula_moiety. This is
            usually due to the moiety formula being in the wrong format.
            Atom count from _chemical_formula_sum:   C348 H240 F48 N52 O48 S16 Zn8
            Atom count from _chemical_formula_moiety:
FORMU01_ALERT_2_G  There is a discrepancy between the atom counts in the
            _chemical_formula_sum and the formula from the _atom_site* data.
            Atom count from _chemical_formula_sum:C348 H240 F48 N52 O48 S16 Zn8
            Atom count from the _atom_site data:  C340.8630 H234.75 F26.589 N52 O2
ABSMU01_ALERT_1_G  Calculation of _exptl_absorpt_correction_mu
                not performed for this radiation type.
CELLZ01_ALERT_1_G Difference between formula and atom_site contents detected.
CELLZ01_ALERT_1_G ALERT: Large difference may be due to a
            symmetry error - see SYMMG tests
           From the CIF: _cell_formula_units_Z    4
           From the CIF: _chemical_formula_sum  C348 H240 F48 N52 O48 S16 Zn8
           TEST: Compare cell contents of formula and atom_site data

           atom    Z*formula  cif sites diff
           C       1392.00   1363.45   28.55
           H        960.00    939.00   21.00
           F        192.00    106.36   85.64
           N        208.00    208.00    0.00
           O        192.00    106.36   85.64
           S         64.00     35.45   28.55
           Zn        32.00     32.00    0.00
PLAT002_ALERT_2_G Number of Distance or Angle Restraints on AtSite        541 Note
PLAT003_ALERT_2_G Number of Uiso or Uij Restrained non-H Atoms ...        541 Report
PLAT041_ALERT_1_G Calc. and Reported SumFormula    Strings  Differ     Please Check
PLAT042_ALERT_1_G Calc. and Reported MoietyFormula Strings  Differ     Please Check
PLAT045_ALERT_1_G Calculated and Reported Z Differ by a Factor ...      0.250 Check
PLAT051_ALERT_1_G Mu(calc) and Mu(CIF) Ratio Differs from 1.0 by .       8.71 %
PLAT092_ALERT_4_G Check: Wavelength Given is not Cu,Ga,Mo,Ag,In Ka    0.68890 Ang.
PLAT171_ALERT_4_G The CIF-Embedded .res File Contains EADP Records          1 Report
PLAT172_ALERT_4_G The CIF-Embedded .res File Contains DFIX Records        217 Report
PLAT173_ALERT_4_G The CIF-Embedded .res File Contains DANG Records        300 Report
PLAT174_ALERT_4_G The CIF-Embedded .res File Contains FLAT Records         15 Report
PLAT175_ALERT_4_G The CIF-Embedded .res File Contains SAME Records          6 Report
PLAT178_ALERT_4_G The CIF-Embedded .res File Contains SIMU Records          6 Report
PLAT186_ALERT_4_G The CIF-Embedded .res File Contains ISOR Records          5 Report
PLAT187_ALERT_4_G The CIF-Embedded .res File Contains RIGU Records          1 Report
PLAT300_ALERT_4_G Atom Site Occupancy of S1J        Constrained at        0.5 Check
PLAT300_ALERT_4_G Atom Site Occupancy of F1J        Constrained at        0.5 Check
PLAT300_ALERT_4_G Atom Site Occupancy of F2J        Constrained at        0.5 Check
PLAT300_ALERT_4_G Atom Site Occupancy of F3J        Constrained at        0.5 Check
PLAT300_ALERT_4_G Atom Site Occupancy of O1J        Constrained at        0.5 Check
PLAT300_ALERT_4_G Atom Site Occupancy of O2J        Constrained at        0.5 Check
PLAT300_ALERT_4_G Atom Site Occupancy of O3J        Constrained at        0.5 Check
```

```
PLAT300_ALERT_4_G Atom Site Occupancy of C1J        Constrained at        0.5 Check
PLAT300_ALERT_4_G Atom Site Occupancy of S1M        Constrained at        0.5 Check
PLAT300_ALERT_4_G Atom Site Occupancy of F1M        Constrained at        0.5 Check
PLAT300_ALERT_4_G Atom Site Occupancy of F2M        Constrained at        0.5 Check
PLAT300_ALERT_4_G Atom Site Occupancy of F3M        Constrained at        0.5 Check
PLAT300_ALERT_4_G Atom Site Occupancy of O1M        Constrained at        0.5 Check
PLAT300_ALERT_4_G Atom Site Occupancy of O2M        Constrained at        0.5 Check
PLAT300_ALERT_4_G Atom Site Occupancy of O3M        Constrained at        0.5 Check
PLAT300_ALERT_4_G Atom Site Occupancy of C1M        Constrained at        0.5 Check
PLAT300_ALERT_4_G Atom Site Occupancy of N1S        Constrained at        0.5 Check
PLAT300_ALERT_4_G Atom Site Occupancy of C1S        Constrained at        0.5 Check
PLAT300_ALERT_4_G Atom Site Occupancy of C2S        Constrained at        0.5 Check
PLAT300_ALERT_4_G Atom Site Occupancy of H2SA       Constrained at        0.5 Check
PLAT300_ALERT_4_G Atom Site Occupancy of H2SB       Constrained at        0.5 Check
PLAT300_ALERT_4_G Atom Site Occupancy of H2SC       Constrained at        0.5 Check
PLAT300_ALERT_4_G Atom Site Occupancy of N2S        Constrained at        0.5 Check
PLAT300_ALERT_4_G Atom Site Occupancy of C3S        Constrained at        0.5 Check
PLAT300_ALERT_4_G Atom Site Occupancy of C4S        Constrained at        0.5 Check
PLAT300_ALERT_4_G Atom Site Occupancy of N3S        Constrained at       0.75 Check
PLAT300_ALERT_4_G Atom Site Occupancy of C5S        Constrained at       0.75 Check
PLAT300_ALERT_4_G Atom Site Occupancy of C6S        Constrained at       0.75 Check
PLAT300_ALERT_4_G Atom Site Occupancy of N4S        Constrained at        0.5 Check
PLAT300_ALERT_4_G Atom Site Occupancy of C7S        Constrained at        0.5 Check
PLAT300_ALERT_4_G Atom Site Occupancy of C8S        Constrained at        0.5 Check
PLAT300_ALERT_4_G Atom Site Occupancy of H8SA       Constrained at        0.5 Check
PLAT300_ALERT_4_G Atom Site Occupancy of H8SB       Constrained at        0.5 Check
PLAT300_ALERT_4_G Atom Site Occupancy of H8SC       Constrained at        0.5 Check
PLAT300_ALERT_4_G Atom Site Occupancy of N5S        Constrained at        0.5 Check
PLAT300_ALERT_4_G Atom Site Occupancy of C9S        Constrained at        0.5 Check
PLAT300_ALERT_4_G Atom Site Occupancy of C10S       Constrained at        0.5 Check
PLAT300_ALERT_4_G Atom Site Occupancy of H10A       Constrained at        0.5 Check
PLAT300_ALERT_4_G Atom Site Occupancy of H10B       Constrained at        0.5 Check
PLAT300_ALERT_4_G Atom Site Occupancy of H10C       Constrained at        0.5 Check
PLAT300_ALERT_4_G Atom Site Occupancy of N6S        Constrained at       0.75 Check
PLAT300_ALERT_4_G Atom Site Occupancy of C11S       Constrained at       0.75 Check
PLAT300_ALERT_4_G Atom Site Occupancy of C12S       Constrained at       0.75 Check
PLAT300_ALERT_4_G Atom Site Occupancy of H12G       Constrained at       0.75 Check
PLAT300_ALERT_4_G Atom Site Occupancy of H12H       Constrained at       0.75 Check
PLAT300_ALERT_4_G Atom Site Occupancy of H12I       Constrained at       0.75 Check
PLAT300_ALERT_4_G Atom Site Occupancy of N7S        Constrained at        0.5 Check
PLAT300_ALERT_4_G Atom Site Occupancy of C13S       Constrained at        0.5 Check
PLAT300_ALERT_4_G Atom Site Occupancy of C14S       Constrained at        0.5 Check
PLAT301_ALERT_3_G Main Residue  Disorder .............(Resd  1  )         1% Note
PLAT302_ALERT_4_G Anion/Solvent/Minor-Residue Disorder (Resd  2  )       100% Note
PLAT302_ALERT_4_G Anion/Solvent/Minor-Residue Disorder (Resd  3  )       100% Note
PLAT302_ALERT_4_G Anion/Solvent/Minor-Residue Disorder (Resd  4  )       100% Note
PLAT302_ALERT_4_G Anion/Solvent/Minor-Residue Disorder (Resd  5  )       100% Note
PLAT302_ALERT_4_G Anion/Solvent/Minor-Residue Disorder (Resd  6  )       100% Note
PLAT302_ALERT_4_G Anion/Solvent/Minor-Residue Disorder (Resd  7  )       100% Note
PLAT302_ALERT_4_G Anion/Solvent/Minor-Residue Disorder (Resd  8  )       100% Note
PLAT302_ALERT_4_G Anion/Solvent/Minor-Residue Disorder (Resd  9  )       100% Note
PLAT302_ALERT_4_G Anion/Solvent/Minor-Residue Disorder (Resd 10  )       100% Note
PLAT302_ALERT_4_G Anion/Solvent/Minor-Residue Disorder (Resd 11  )       100% Note
PLAT302_ALERT_4_G Anion/Solvent/Minor-Residue Disorder (Resd 12  )       100% Note
PLAT302_ALERT_4_G Anion/Solvent/Minor-Residue Disorder (Resd 13  )       100% Note
PLAT302_ALERT_4_G Anion/Solvent/Minor-Residue Disorder (Resd 14  )       100% Note
PLAT302_ALERT_4_G Anion/Solvent/Minor-Residue Disorder (Resd 15  )       100% Note
```

```
PLAT302_ALERT_4_G Anion/Solvent/Minor-Residue Disorder (Resd 16  )       100% Note
PLAT302_ALERT_4_G Anion/Solvent/Minor-Residue Disorder (Resd 17  )       100% Note
PLAT302_ALERT_4_G Anion/Solvent/Minor-Residue Disorder (Resd 18  )       100% Note
PLAT302_ALERT_4_G Anion/Solvent/Minor-Residue Disorder (Resd 19  )       100% Note
PLAT302_ALERT_4_G Anion/Solvent/Minor-Residue Disorder (Resd 20  )       100% Note
PLAT302_ALERT_4_G Anion/Solvent/Minor-Residue Disorder (Resd 21  )       100% Note
PLAT302_ALERT_4_G Anion/Solvent/Minor-Residue Disorder (Resd 22  )       100% Note
PLAT302_ALERT_4_G Anion/Solvent/Minor-Residue Disorder (Resd 23  )       100% Note
PLAT302_ALERT_4_G Anion/Solvent/Minor-Residue Disorder (Resd 24  )       100% Note
PLAT302_ALERT_4_G Anion/Solvent/Minor-Residue Disorder (Resd 25  )       100% Note
PLAT302_ALERT_4_G Anion/Solvent/Minor-Residue Disorder (Resd 26  )       100% Note
PLAT304_ALERT_4_G Non-Integer Number of Atoms in ..... (Resd  2  )       7.05 Check
PLAT304_ALERT_4_G Non-Integer Number of Atoms in ..... (Resd  3  )       4.41 Check
PLAT304_ALERT_4_G Non-Integer Number of Atoms in ..... (Resd  5  )       6.29 Check
PLAT304_ALERT_4_G Non-Integer Number of Atoms in ..... (Resd  7  )       7.52 Check
PLAT304_ALERT_4_G Non-Integer Number of Atoms in ..... (Resd  8  )       6.26 Check
PLAT304_ALERT_4_G Non-Integer Number of Atoms in ..... (Resd  9  )       5.02 Check
PLAT304_ALERT_4_G Non-Integer Number of Atoms in ..... (Resd 10  )       2.84 Check
PLAT304_ALERT_4_G Non-Integer Number of Atoms in ..... (Resd 11  )       3.06 Check
PLAT304_ALERT_4_G Non-Integer Number of Atoms in ..... (Resd 12  )       3.53 Check
PLAT304_ALERT_4_G Non-Integer Number of Atoms in ..... (Resd 13  )       3.70 Check
PLAT304_ALERT_4_G Non-Integer Number of Atoms in ..... (Resd 14  )       2.16 Check
PLAT304_ALERT_4_G Non-Integer Number of Atoms in ..... (Resd 15  )       3.08 Check
PLAT304_ALERT_4_G Non-Integer Number of Atoms in ..... (Resd 16  )       2.78 Check
PLAT304_ALERT_4_G Non-Integer Number of Atoms in ..... (Resd 17  )       1.51 Check
PLAT304_ALERT_4_G Non-Integer Number of Atoms in ..... (Resd 18  )       0.87 Check
PLAT304_ALERT_4_G Non-Integer Number of Atoms in ..... (Resd 19  )       2.84 Check
PLAT304_ALERT_4_G Non-Integer Number of Atoms in ..... (Resd 21  )       1.50 Check
PLAT304_ALERT_4_G Non-Integer Number of Atoms in ..... (Resd 22  )       2.25 Check
PLAT304_ALERT_4_G Non-Integer Number of Atoms in ..... (Resd 25  )       4.50 Check
PLAT304_ALERT_4_G Non-Integer Number of Atoms in ..... (Resd 26  )       1.50 Check
PLAT315_ALERT_2_G Singly Bonded Carbon Detected (H-atoms Missing).        C4S Check
PLAT315_ALERT_2_G Singly Bonded Carbon Detected (H-atoms Missing).        C6S Check
PLAT315_ALERT_2_G Singly Bonded Carbon Detected (H-atoms Missing).       C14S Check
PLAT410_ALERT_2_G Short Intra H...H Contact  H36E     ..H32G     .       2.10 Ang.
                                                        x,y,z  =     1_555 Check
PLAT410_ALERT_2_G Short Intra H...H Contact  H36E     ..H33G     .       2.12 Ang.
                                                        x,y,z  =     1_555 Check
PLAT413_ALERT_2_G Short Inter XH3 .. XHn     H8SA     ..H42F     .       1.99 Ang.
                                                        x,y,z  =     1_555 Check
PLAT432_ALERT_2_G Short Inter X...Y Contact  F2L      ..C18C     .       2.91 Ang.
                                                        x,y,z  =     1_555 Check
PLAT432_ALERT_2_G Short Inter X...Y Contact  F2M      ..C16F     .       2.89 Ang.
                                                        x,y,z  =     1_555 Check
PLAT432_ALERT_2_G Short Inter X...Y Contact  F3M      ..C10S     .       2.97 Ang.
                                                        x,y,z  =     1_555 Check
PLAT432_ALERT_2_G Short Inter X...Y Contact  F3M      ..C9S      .       2.97 Ang.
                                                        x,y,z  =     1_555 Check
PLAT432_ALERT_2_G Short Inter X...Y Contact  O1L      ..C2F      .       3.02 Ang.
                                                x,1/2-y,1/2+z  =     4_566 Check
PLAT432_ALERT_2_G Short Inter X...Y Contact  O1M      ..C18D     .       3.00 Ang.
                                                        x,y,z  =     1_555 Check
PLAT432_ALERT_2_G Short Inter X...Y Contact  O1P      ..C42E     .       2.92 Ang.
                                                        x,y,z  =     1_555 Check
PLAT432_ALERT_2_G Short Inter X...Y Contact  O2H      ..C30D     .       2.92 Ang.
                                                        x,y,z  =     1_555 Check
PLAT432_ALERT_2_G Short Inter X...Y Contact  O2U      ..C30C     .       3.02 Ang.
```

```
                                                x,y,z  =      1_555 Check
PLAT432_ALERT_2_G Short Inter X...Y Contact  F2Q      ..C24A     .       2.82 Ang.
                                                x,y,z  =      1_555 Check
PLAT432_ALERT_2_G Short Inter X...Y Contact  F3Q      ..C18A     .       2.59 Ang.
                                                x,y,z  =      1_555 Check
PLAT432_ALERT_2_G Short Inter X...Y Contact  F1OO     ..C42F     .       2.77 Ang.
                                                x,y,z  =      1_555 Check
PLAT432_ALERT_2_G Short Inter X...Y Contact  F1OO     ..C40F     .       2.96 Ang.
                                                x,y,z  =      1_555 Check
PLAT432_ALERT_2_G Short Inter X...Y Contact  N1S      ..C42C     .       2.91 Ang.
                                       x,1/2-y,-1/2+z  =      4_565 Check
PLAT432_ALERT_2_G Short Inter X...Y Contact  N6S      ..C6A      .       3.04 Ang.
                                                x,y,z  =      1_555 Check
PLAT432_ALERT_2_G Short Inter X...Y Contact  O1Q      ..C16E     .       2.94 Ang.
                                                x,y,z  =      1_555 Check
PLAT432_ALERT_2_G Short Inter X...Y Contact  O3HH     ..C33B     .       2.84 Ang.
                                                x,y,z  =      1_555 Check
PLAT432_ALERT_2_G Short Inter X...Y Contact  O2O      ..C18E     .       2.95 Ang.
                                          1-x,1-y,1-z  =      3_666 Check
PLAT432_ALERT_2_G Short Inter X...Y Contact  O3T      ..C28F     .       2.78 Ang.
                                                x,y,z  =      1_555 Check
PLAT432_ALERT_2_G Short Inter X...Y Contact  O3T      ..C16A     .       2.99 Ang.
                                          1-x,1-y,1-z  =      3_666 Check
PLAT432_ALERT_2_G Short Inter X...Y Contact  O2KK     ..C24A     .       2.89 Ang.
                                                x,y,z  =      1_555 Check
PLAT432_ALERT_2_G Short Inter X...Y Contact  O3KK     ..C18A     .       2.95 Ang.
                                                x,y,z  =      1_555 Check
PLAT432_ALERT_2_G Short Inter X...Y Contact  O1OO     ..C28F     .       2.76 Ang.
                                                x,y,z  =      1_555 Check
PLAT432_ALERT_2_G Short Inter X...Y Contact  O3OO     ..C18E     .       2.70 Ang.
                                          1-x,1-y,1-z  =      3_666 Check
PLAT432_ALERT_2_G Short Inter X...Y Contact  O3OO     ..C37F     .       3.02 Ang.
                                          1-x,1-y,1-z  =      3_666 Check
PLAT432_ALERT_2_G Short Inter X...Y Contact  O1UU     ..C2S      .       2.92 Ang.
                                        x,1/2-y,1/2+z  =      4_566 Check
PLAT432_ALERT_2_G Short Inter X...Y Contact  O2UU     ..C42C     .       2.65 Ang.
                                                x,y,z  =      1_555 Check
PLAT432_ALERT_2_G Short Inter X...Y Contact  O2UU     ..C41C     .       2.94 Ang.
                                                x,y,z  =      1_555 Check
PLAT432_ALERT_2_G Short Inter X...Y Contact  O2UU     ..C40C     .       2.95 Ang.
                                                x,y,z  =      1_555 Check
PLAT432_ALERT_2_G Short Inter X...Y Contact  C28B     ..C40C     .       3.13 Ang.
                                    -1+x,1/2-y,-1/2+z  =      4_465 Check
PLAT606_ALERT_4_G Solvent Accessible VOID(S) in Structure ........          ! Info
PLAT720_ALERT_4_G Number of Unusual/Non-Standard Labels ..........         46 Note
PLAT722_ALERT_1_G Angle   Calc      123.00, Rep      121.50 Dev...       1.50 Degree
              C31C    -C36G    -H36G       1_555  1_555  1_555     #  695 Check
PLAT794_ALERT_5_G Tentative Bond Valency for Zn1       (II)      .       1.87 Info
PLAT794_ALERT_5_G Tentative Bond Valency for Zn2       (II)      .       1.84 Info
PLAT794_ALERT_5_G Tentative Bond Valency for Zn3       (II)      .       1.82 Info
PLAT794_ALERT_5_G Tentative Bond Valency for Zn4       (II)      .       1.92 Info
PLAT794_ALERT_5_G Tentative Bond Valency for Zn5       (II)      .       1.86 Info
PLAT794_ALERT_5_G Tentative Bond Valency for Zn6       (II)      .       1.94 Info
PLAT794_ALERT_5_G Tentative Bond Valency for Zn7       (II)      .       1.82 Info
PLAT802_ALERT_4_G CIF Input Record(s) with more than 80 Characters          1 Info
PLAT860_ALERT_3_G Number of Least-Squares Restraints ............        9973 Note
PLAT869_ALERT_4_G ALERTS Related to the Use of SQUEEZE  Suppressed          ! Info
```

```
PLAT883_ALERT_1_G No Info/Value for _atom_sites_solution_primary .      Please Do !
PLAT908_ALERT_2_G Max. Perc. Data with I > 2*s(I) per Res.Shell .       72.14% Note
PLAT910_ALERT_3_G Missing # of FCF Reflection(s) Below Theta(Min).           4 Note
PLAT913_ALERT_3_G Missing # of Very Strong Reflections in FCF ....           2 Note
PLAT933_ALERT_2_G Number of HKL-OMIT Records in Embedded .res File          30 Note
PLAT941_ALERT_3_G Average HKL Measurement Multiplicity ..........          3.3 Low
PLAT965_ALERT_2_G The SHELXL WEIGHT Optimisation has not Converged     Please Check
PLAT978_ALERT_2_G Number C-C Bonds with Positive Residual Density.           1 Info
PLAT984_ALERT_1_G The  S-f'=    0.1203 Deviates from the B&C-Value     0.1187 Check
PLAT984_ALERT_1_G The Zn-f'=    0.3032 Deviates from the B&C-Value     0.3063 Check
PLAT985_ALERT_1_G The Zn-f"=    1.3627 Deviates from the B&C-Value     1.3615 Check
```

```
   1 ALERT level A = Most likely a serious problem - resolve or explain
   5 ALERT level B = A potentially serious problem, consider carefully
  99 ALERT level C = Check. Ensure it is not caused by an omission or oversight
 175 ALERT level G = General information/check it is not something unexpected

  30 ALERT type 1 CIF construction/syntax error, inconsistent or missing data
 121 ALERT type 2 Indicator that the structure model may be wrong or deficient
  14 ALERT type 3 Indicator that the structure quality may be low
 108 ALERT type 4 Improvement, methodology, query or suggestion
   7 ALERT type 5 Informative message, check
```

It is advisable to attempt to resolve as many as possible of the alerts in all categories. Often the minor alerts point to easily fixed oversights, errors and omissions in your CIF or refinement strategy, so attention to these fine details can be worthwhile. In order to resolve some of the more serious problems it may be necessary to carry out additional measurements or structure refinements. However, the purpose of your study may justify the reported deviations and the more serious of these should normally be commented upon in the discussion or experimental section of a paper or in the "special_details" fields of the CIF. checkCIF was carefully designed to identify outliers and unusual parameters, but every test has its limitations and alerts that are not important in a particular case may appear. Conversely, the absence of alerts does not guarantee there are no aspects of the results needing attention. It is up to the individual to critically assess their own results and, if necessary, seek expert advice.

Publication of your CIF in IUCr journals

A basic structural check has been run on your CIF. These basic checks will be run on all CIFs submitted for publication in IUCr journals (*Acta Crystallographica*, *Journal of Applied Crystallography*, *Journal of Synchrotron Radiation*); however, if you intend to submit to *Acta Crystallographica Section C* or *E* or *IUCrData*, you should make sure that full publication checks are run on the final version of your CIF prior to submission.

Publication of your CIF in other journals

Please refer to the *Notes for Authors* of the relevant journal for any special instructions relating to CIF submission.

Validation response form

Please find below a validation response form (VRF) that can be filled in and pasted into your CIF.

```
# start Validation Reply Form
_vrf_PLAT934_jd354_sq
;
PROBLEM: Number of (Iobs-Icalc)/Sigma(W) > 10 Outliers ..          4 Check
RESPONSE: ...
;
# end Validation Reply Form
```

PLATON version of 18/05/2022; check.def file version of 17/05/2022

Datablock jd354_sq - ellipsoid plot

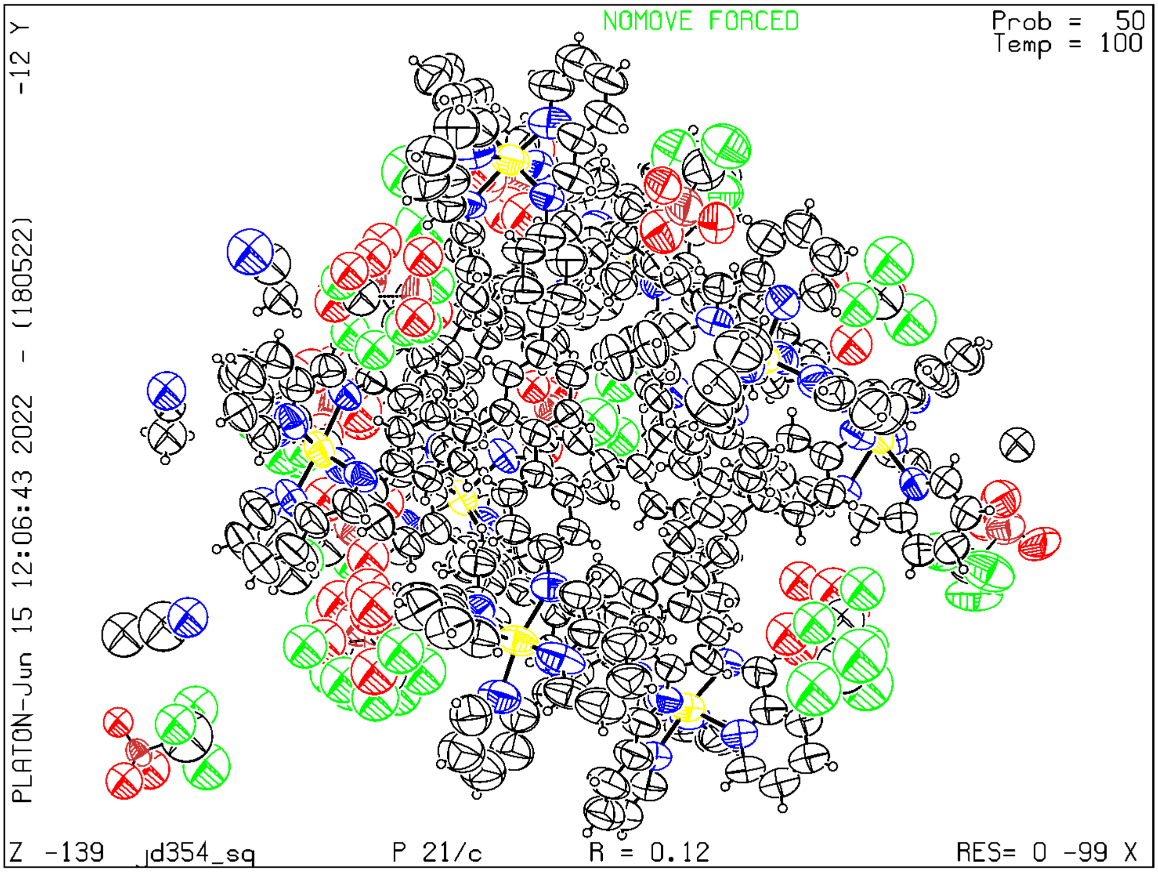

Supplement: Supplementary file 11 — Supporting Information [file ANIE-62-0-s007.pdf]
