# Supplementary material for: Tetramine Aspect Ratio and Flexibility Determine Framework Symmetry for Zn8L6 Self‐Assembled Structures
Source: Angew Chem Int Ed Engl. 2023 Feb 1;62(10):e202217987. doi: 10.1002/anie.202217987 (PMC10946785; doi:10.1002/anie.202217987)

checkCIF/PLATON report

Structure factors have been supplied for datablock(s) jd257_sq

THIS REPORT IS FOR GUIDANCE ONLY. IF USED AS PART OF A REVIEW PROCEDURE FOR PUBLICATION, IT SHOULD NOT REPLACE THE EXPERTISE OF AN EXPERIENCED CRYSTALLOGRAPHIC REFEREE.

No syntax errors found. CIF dictionary Interpreting this report

Datablock: jd257_sq

Bond precision: C-C = 0.0148 A Wavelength=0.68890

Cell:	a=27.3930(3)	b=27.3930(3)	c=125.954(3)
	alpha=90	beta=90	gamma=120
Temperature:	100 K		

	Calculated	Reported
Volume	81851(3)	81851(3)
Space group	R -3 c	R -3 c :H
Hall group	-R 3 2"c	-R 3 2"c
Moiety formula		
Sum formula	C402 H285 Cl16 N51 O66 Zn8 [+ solvent]	C268 H192.67 Cl10.67 N34 O44 Zn5.33
Mr	7975.93	5319.99
Dx,g cm-3	0.971	0.971
Z	6	9
Mu (mm-1)	0.441	0.441
F000	24563.4	24588.0
F000'	24599.92	
h,k,lmax	27,27,125	27,27,125
Nref	9556	9546
Tmin,Tmax	0.979,0.987	0.984,1.000
Tmin'	0.978	

Correction method= # Reported T Limits: Tmin=0.984 Tmax=1.000
AbsCorr = EMPIRICAL

Data completeness= 0.999 Theta(max)= 20.148

R(reflections)= 0.1182(5368) wR2(reflections)= 0.3740(9546)

S = 1.191 Npar= 1010

The following ALERTS were generated. Each ALERT has the format
test-name_ALERT_alert-type_alert-level.
Click on the hyperlinks for more details of the test.

Alert level A

```
THETM01_ALERT_3_A  The value of sine(theta_max)/wavelength is less than 0.550
            Calculated sin(theta_max)/wavelength =    0.5000
```

Author Response: Despite the use of a synchrotron radiation and rapid sample handling, few reflections at greater than 1.0 A resolution were observed and the data was trimmed accordingly.

Alert level B

```
PLAT084_ALERT_3_B High wR2 Value (i.e. > 0.25) ...................       0.37 Report
```

Author Response: The quality of the integration was lower than ideal due to the poor diffraction properties of the crystals. As a consequence the values of the R1, wR and wR2 factors are larger than typical small molecule

```
PLAT260_ALERT_2_B Large Average Ueq of Residue Including       Cl2      0.330 Check
```

Author Response: These alerts result from thermal motion and/or minor unresolved disorder of some anions.

```
PLAT260_ALERT_2_B Large Average Ueq of Residue Including       Cl3      0.302 Check
```

Author Response: These alerts result from thermal motion and/or minor unresolved disorder of some anions.

```
PLAT260_ALERT_2_B Large Average Ueq of Residue Including       Cl4      0.337 Check
```

Author Response: These alerts result from thermal motion and/or minor unresolved disorder of some anions.

Alert level C

```
PLAT082_ALERT_2_C High R1 Value ..................................       0.12 Report
PLAT088_ALERT_3_C Poor Data / Parameter Ratio ....................       9.45 Note
PLAT241_ALERT_2_C High   'MainMol' Ueq as Compared to Neighbors of         C3 Check
PLAT241_ALERT_2_C High   'MainMol' Ueq as Compared to Neighbors of        C13 Check
PLAT241_ALERT_2_C High   'MainMol' Ueq as Compared to Neighbors of        C15 Check
PLAT241_ALERT_2_C High   'MainMol' Ueq as Compared to Neighbors of        C26 Check
PLAT241_ALERT_2_C High   'MainMol' Ueq as Compared to Neighbors of        C28 Check
```

```
PLAT241_ALERT_2_C High   'MainMol' Ueq as Compared to Neighbors of        C30 Check
PLAT241_ALERT_2_C High   'MainMol' Ueq as Compared to Neighbors of        C38 Check
PLAT241_ALERT_2_C High   'MainMol' Ueq as Compared to Neighbors of        C39 Check
PLAT242_ALERT_2_C Low    'MainMol' Ueq as Compared to Neighbors of         N3 Check
PLAT242_ALERT_2_C Low    'MainMol' Ueq as Compared to Neighbors of         N5 Check
PLAT242_ALERT_2_C Low    'MainMol' Ueq as Compared to Neighbors of         N7 Check
PLAT260_ALERT_2_C Large Average Ueq of Residue Including       Zn1      0.149 Check
```

Author Response: These alerts result from thermal motion and/or minor unresolved disorder of some anions.

```
PLAT260_ALERT_2_C Large Average Ueq of Residue Including       Cl1      0.199 Check
```

Author Response: These alerts result from thermal motion and/or minor unresolved disorder of some anions.

```
PLAT260_ALERT_2_C Large Average Ueq of Residue Including      Cl1'      0.170 Check
```

Author Response: These alerts result from thermal motion and/or minor unresolved disorder of some anions.

```
PLAT260_ALERT_2_C Large Average Ueq of Residue Including       N2S      0.180 Check
```

Author Response: These alerts result from thermal motion and/or minor unresolved disorder of some anions.

```
PLAT341_ALERT_3_C Low Bond Precision on  C-C Bonds ...............    0.01476 Ang.
PLAT911_ALERT_3_C Missing FCF Refl Between Thmin & STh/L=    0.500         11 Report
PLAT918_ALERT_3_C Reflection(s) with I(obs) much Smaller I(calc) .          1 Check
PLAT975_ALERT_2_C Check Calcd Resid. Dens.  0.65Ang From O1W     .       0.44 eA-3
```

Alert level G

```
FORMU01_ALERT_1_G  There is a discrepancy between the atom counts in the
            _chemical_formula_sum and _chemical_formula_moiety. This is
            usually due to the moiety formula being in the wrong format.
            Atom count from _chemical_formula_sum:   C268 H192.6700 Cl10.67 N34 O4
            Atom count from _chemical_formula_moiety:
FORMU01_ALERT_2_G  There is a discrepancy between the atom counts in the
            _chemical_formula_sum and the formula from the _atom_site* data.
            Atom count from _chemical_formula_sum:C268 H192.6700 Cl10.67 N34 O44 Z
            Atom count from the _atom_site data:  C268. H190 Cl10.66533 N34 O43.99
ABSMU01_ALERT_1_G  Calculation of _exptl_absorpt_correction_mu
                not performed for this radiation type.
CELLZ01_ALERT_1_G Difference between formula and atom_site contents detected.
CELLZ01_ALERT_1_G WARNING: H atoms missing from atom site list. Is this intentional?
           From the CIF: _cell_formula_units_Z    9
           From the CIF: _chemical_formula_sum  C268 H192.67 Cl10.67 N34 O44 Zn5.3
           TEST: Compare cell contents of formula and atom_site data
```

```
          atom    Z*formula  cif sites diff
          C       2412.00   2412.00   -0.00
          H       1734.03   1710.00   24.03
          Cl        96.03     95.99    0.04
          N        306.00    306.00    0.00
          O        396.00    395.95    0.05
          Zn        47.97     48.00   -0.03
PLAT002_ALERT_2_G Number of Distance or Angle Restraints on AtSite        128 Note
PLAT003_ALERT_2_G Number of Uiso or Uij Restrained non-H Atoms ...        128 Report
PLAT041_ALERT_1_G Calc. and Reported SumFormula    Strings  Differ     Please Check
PLAT042_ALERT_1_G Calc. and Reported MoietyFormula Strings  Differ     Please Check
PLAT045_ALERT_1_G Calculated and Reported Z Differ by a Factor ...      0.667 Check
PLAT083_ALERT_2_G SHELXL Second Parameter in WGHT  Unusually Large     150.00 Why ?
PLAT092_ALERT_4_G Check: Wavelength Given is not Cu,Ga,Mo,Ag,In Ka    0.68890 Ang.
PLAT172_ALERT_4_G The CIF-Embedded .res File Contains DFIX Records        111 Report
PLAT173_ALERT_4_G The CIF-Embedded .res File Contains DANG Records        133 Report
PLAT174_ALERT_4_G The CIF-Embedded .res File Contains FLAT Records        105 Report
PLAT175_ALERT_4_G The CIF-Embedded .res File Contains SAME Records          2 Report
PLAT178_ALERT_4_G The CIF-Embedded .res File Contains SIMU Records          6 Report
PLAT186_ALERT_4_G The CIF-Embedded .res File Contains ISOR Records          2 Report
PLAT187_ALERT_4_G The CIF-Embedded .res File Contains RIGU Records          1 Report
PLAT244_ALERT_4_G Low    'Solvent' Ueq as Compared to Neighbors of        Cl3 Check
PLAT300_ALERT_4_G Atom Site Occupancy of N2S       Constrained at         0.5 Check
PLAT300_ALERT_4_G Atom Site Occupancy of C3S       Constrained at         0.5 Check
PLAT300_ALERT_4_G Atom Site Occupancy of C4S       Constrained at         0.5 Check
PLAT300_ALERT_4_G Atom Site Occupancy of H4SA      Constrained at         0.5 Check
PLAT300_ALERT_4_G Atom Site Occupancy of H4SB      Constrained at         0.5 Check
PLAT300_ALERT_4_G Atom Site Occupancy of H4SC      Constrained at         0.5 Check
PLAT300_ALERT_4_G Atom Site Occupancy of O1W       Constrained at         0.5 Check
PLAT300_ALERT_4_G Atom Site Occupancy of O2W       Constrained at        0.25 Check
PLAT301_ALERT_3_G Main Residue  Disorder .............(Resd  1  )         37% Note
PLAT302_ALERT_4_G Anion/Solvent/Minor-Residue Disorder (Resd  2  )       100% Note
PLAT302_ALERT_4_G Anion/Solvent/Minor-Residue Disorder (Resd  3  )       100% Note
PLAT302_ALERT_4_G Anion/Solvent/Minor-Residue Disorder (Resd  5  )       100% Note
PLAT302_ALERT_4_G Anion/Solvent/Minor-Residue Disorder (Resd  6  )       100% Note
PLAT302_ALERT_4_G Anion/Solvent/Minor-Residue Disorder (Resd  7  )       100% Note
PLAT302_ALERT_4_G Anion/Solvent/Minor-Residue Disorder (Resd  8  )       100% Note
PLAT302_ALERT_4_G Anion/Solvent/Minor-Residue Disorder (Resd  9  )       100% Note
PLAT304_ALERT_4_G Non-Integer Number of Atoms in ..... (Resd  2  )       3.52 Check
PLAT304_ALERT_4_G Non-Integer Number of Atoms in ..... (Resd  3  )       3.64 Check
PLAT304_ALERT_4_G Non-Integer Number of Atoms in ..... (Resd  5  )       3.03 Check
PLAT304_ALERT_4_G Non-Integer Number of Atoms in ..... (Resd  6  )       1.48 Check
PLAT304_ALERT_4_G Non-Integer Number of Atoms in ..... (Resd  8  )       0.08 Check
PLAT304_ALERT_4_G Non-Integer Number of Atoms in ..... (Resd  9  )       0.25 Check
PLAT311_ALERT_2_G Isolated Disordered Oxygen Atom (No H's ?) .....        O1W Check
PLAT311_ALERT_2_G Isolated Disordered Oxygen Atom (No H's ?) .....        O2W Check
PLAT410_ALERT_2_G Short Intra H...H Contact  H42      ..H44'     .       1.94 Ang.
                                                      x,y,z  =      1_555 Check
PLAT432_ALERT_2_G Short Inter X...Y Contact  O1       ..C42      .       2.99 Ang.
                                                1-y,1+x-y,z  =      2_665 Check
PLAT432_ALERT_2_G Short Inter X...Y Contact  O7       ..C37      .       2.60 Ang.
                                                1-y,1+x-y,z  =      2_665 Check
PLAT432_ALERT_2_G Short Inter X...Y Contact  O9       ..C30      .       2.92 Ang.
                                                      x,y,z  =      1_555 Check
PLAT606_ALERT_4_G Solvent Accessible VOID(S) in Structure ........          ! Info
PLAT720_ALERT_4_G Number of Unusual/Non-Standard Labels ..........          3 Note
PLAT790_ALERT_4_G Centre of Gravity not Within Unit Cell: Resd.  #          2 Note
```

```
              Cl O4
PLAT790_ALERT_4_G Centre of Gravity not Within Unit Cell: Resd.  #          3 Note
              Cl O4
PLAT790_ALERT_4_G Centre of Gravity not Within Unit Cell: Resd.  #          6 Note
              Cl O4
PLAT790_ALERT_4_G Centre of Gravity not Within Unit Cell: Resd.  #          7 Note
              C2 H3 N
PLAT794_ALERT_5_G Tentative Bond Valency for Zn1       (II)      .       1.84 Info
PLAT802_ALERT_4_G CIF Input Record(s) with more than 80 Characters          1 Info
PLAT811_ALERT_5_G No ADDSYM Analysis: Too Many Excluded Atoms ....          ! Info
PLAT860_ALERT_3_G Number of Least-Squares Restraints ............        2487 Note
PLAT869_ALERT_4_G ALERTS Related to the Use of SQUEEZE  Suppressed          ! Info
PLAT883_ALERT_1_G No Info/Value for _atom_sites_solution_primary .     Please Do !
PLAT933_ALERT_2_G Number of HKL-OMIT Records in Embedded .res File          9 Note
PLAT965_ALERT_2_G The SHELXL WEIGHT Optimisation has not Converged     Please Check
PLAT978_ALERT_2_G Number C-C Bonds with Positive Residual Density.          0 Info
PLAT984_ALERT_1_G The Cl-f'=    0.1435 Deviates from the B&C-Value     0.1420 Check
PLAT984_ALERT_1_G The Zn-f'=    0.3032 Deviates from the B&C-Value     0.3063 Check
PLAT985_ALERT_1_G The Cl-f"=    0.1514 Deviates from the B&C-Value     0.1501 Check
PLAT985_ALERT_1_G The Zn-f"=    1.3627 Deviates from the B&C-Value     1.3615 Check
```

```
   1 ALERT level A = Most likely a serious problem - resolve or explain
   4 ALERT level B = A potentially serious problem, consider carefully
  21 ALERT level C = Check. Ensure it is not caused by an omission or oversight
  67 ALERT level G = General information/check it is not something unexpected

  12 ALERT type 1 CIF construction/syntax error, inconsistent or missing data
  33 ALERT type 2 Indicator that the structure model may be wrong or deficient
   8 ALERT type 3 Indicator that the structure quality may be low
  38 ALERT type 4 Improvement, methodology, query or suggestion
   2 ALERT type 5 Informative message, check
```

It is advisable to attempt to resolve as many as possible of the alerts in all categories. Often the minor alerts point to easily fixed oversights, errors and omissions in your CIF or refinement strategy, so attention to these fine details can be worthwhile. In order to resolve some of the more serious problems it may be necessary to carry out additional measurements or structure refinements. However, the purpose of your study may justify the reported deviations and the more serious of these should normally be commented upon in the discussion or experimental section of a paper or in the "special_details" fields of the CIF. checkCIF was carefully designed to identify outliers and unusual parameters, but every test has its limitations and alerts that are not important in a particular case may appear. Conversely, the absence of alerts does not guarantee there are no aspects of the results needing attention. It is up to the individual to critically assess their own results and, if necessary, seek expert advice.

Publication of your CIF in IUCr journals

A basic structural check has been run on your CIF. These basic checks will be run on all CIFs submitted for publication in IUCr journals (*Acta Crystallographica*, *Journal of Applied Crystallography*, *Journal of Synchrotron Radiation*); however, if you intend to submit to *Acta Crystallographica Section C* or *E* or *IUCrData*, you should make sure that full publication checks are run on the final version of your CIF prior to submission.

Publication of your CIF in other journals

Please refer to the *Notes for Authors* of the relevant journal for any special instructions relating to CIF submission.

PLATON version of 18/05/2022; check.def file version of 17/05/2022

Datablock jd257_sq - ellipsoid plot

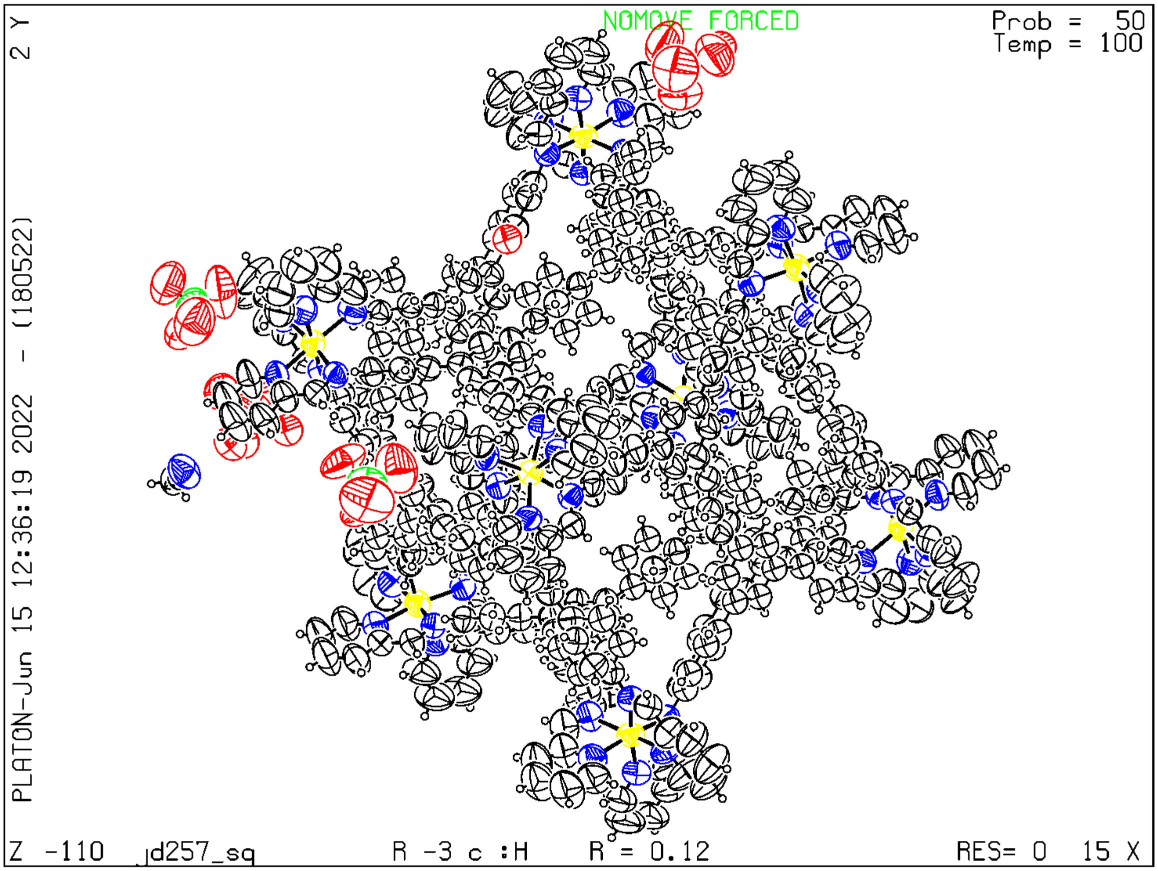

Supplement: Supplementary file 9 — Supporting Information [file ANIE-62-0-s004.pdf]
